# Dearomative Spirocyclization of Tryptamine-Derived Isocyanides via Iron-Catalyzed Carbene Transfer

**DOI:** 10.1021/acs.joc.3c02160

**Published:** 2023-12-04

**Authors:** Thomas R. Roose, Finn McSorley, Bryan Groenhuijzen, Jordy M. Saya, Bert U. W. Maes, Romano V. A. Orrù, Eelco Ruijter

**Affiliations:** † Department of Chemistry & Pharmaceutical Sciences and Amsterdam Institute for Molecular & Life Science (AIMMS), Vrije Universiteit Amsterdam, De Boelelaan 1108, 1081 HZ Amsterdam, The Netherlands; ‡ Organic Synthesis Division, Department of Chemistry, University of Antwerp, Groenenborgerlaan 171, B-2020 Antwerp, Belgium.s; § Organic Chemistry, Aachen-Maastricht Institute for Biobased Materials (AMIBM), Maastricht University, Urmonderbaan 22, 6167 KD Geleen, Netherlands

## Abstract

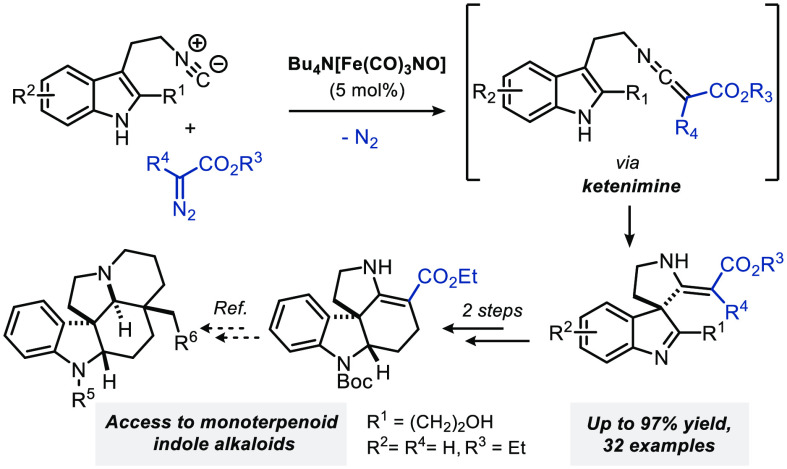

Tryptamine-derived isocyanides are valuable building blocks in the construction of spirocyclic indolenines and indolines via dearomatization of the indole moiety. We report the Bu_4_N[Fe(CO)_3_NO]-catalyzed carbene transfer of α-diazo esters to 3-(2-isocyanoethyl)indoles, leading to ketenimine intermediates that undergo spontaneous dearomative spirocyclization. The utility of this iron-catalyzed carbene transfer/spirocyclization cascade was demonstrated by its use as a key step in the formal total synthesis of monoterpenoid indole alkaloids (±)-aspidofractinine, (±)-limaspermidine, (±)-aspidospermidine, and (±)-17-demethoxy-*N*-acetylcylindrocarine.

## Introduction

**Scheme 1:**
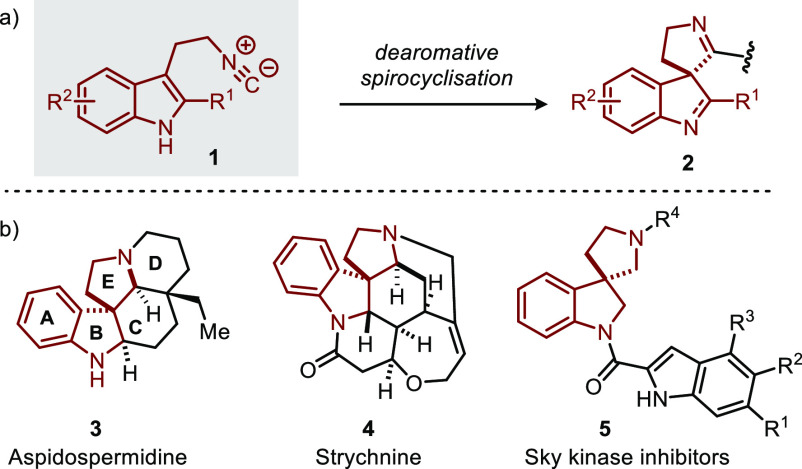
Dearomative Spirocyclization of 3-(2-Isocyanoethyl)indoles

**Scheme 2:**
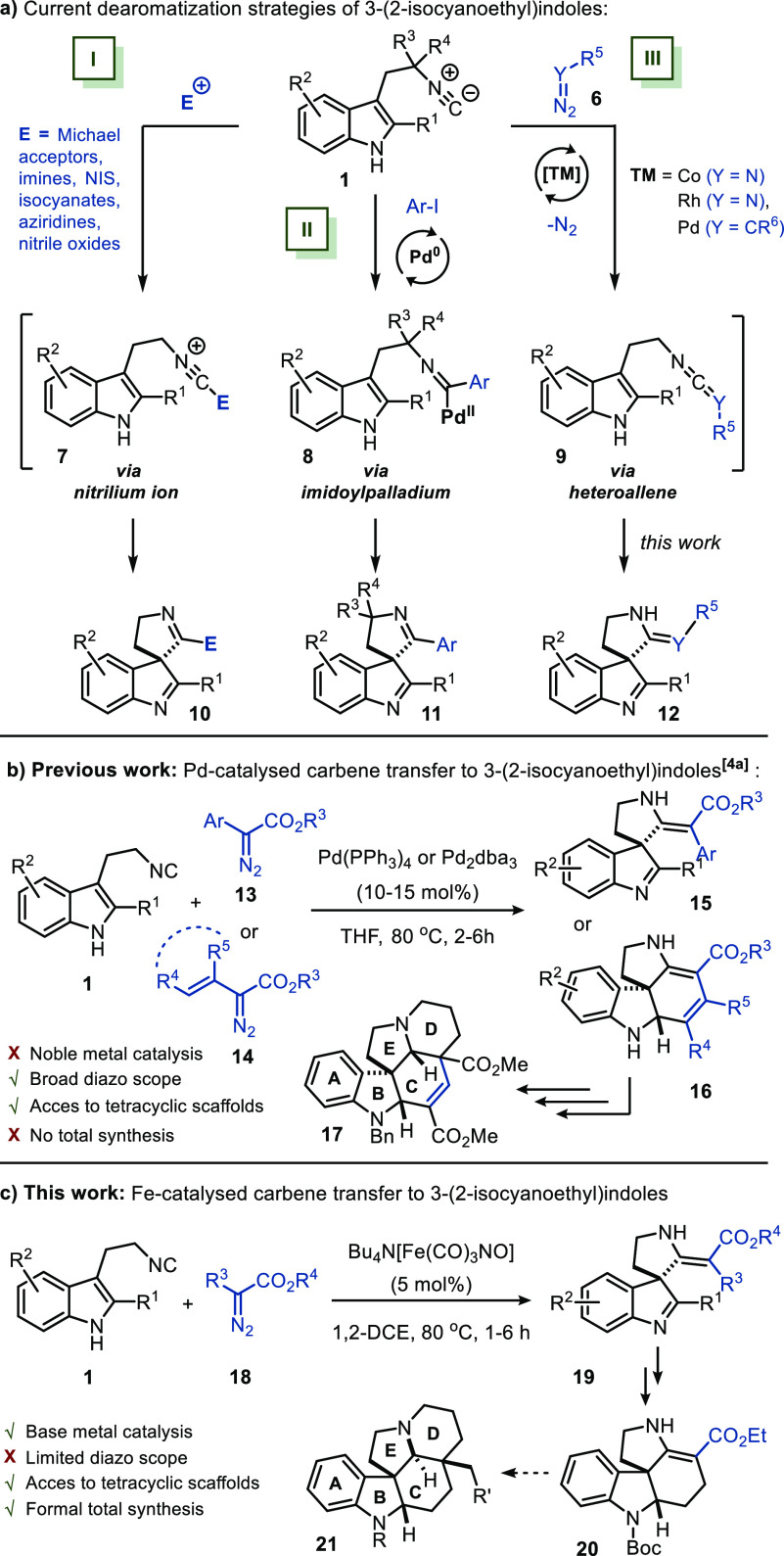
Strategies for Dearomatization of 3-(2-Isocyanoethyl)indoles

Functionalized isocyanides have proven valuable building blocks in organic chemistry. Tethering the isocyanide moiety to other reactive functionalities provides great opportunities for the development of novel cascade and multicomponent processes.^1^ For example, 3-(2-isocyanoethyl)indoles (**1**, Scheme 1a) have recently attracted considerable interest, as they allow for the facile construction of (polycyclic) spiroindol(en)ines **2**(2−5) through dearomatization of the indole moiety.^6^ These spiroindolenines/indolines (**2**) are of considerable relevance as these motifs occur in, e.g., medicinally relevant compounds,^7^ such as Sky kinase inhibitor **5**(7a) and monoterpenoid indole alkaloids of the *Aspidosperma* and *Strychnos* types (Scheme 1b).^8^ Notably, strategies toward construction of these natural products often involve dearomatization of the indole moiety.^9^ Several strategies for the dearomative spirocyclization of 3-(2-isocyanoethyl)indoles **1** have been reported,^3−5^ which differ in the transformation of the isocyano moiety providing different functionalities allowing spirocyclization (Scheme 2a). The first strategy (I) relies on trapping the isocyano moiety by an electrophile, resulting in nitrilium ion **7**. Subsequently, this intermediate is trapped in an intramolecular fashion by the indole C3 position.

Multiple electrophiles have been applied in the formation of spirocyclic indolenines and indolines.^3^ Moreover, our group has demonstrated that using NIS as electrophile, 3-(2-isocyanoethyl) indoles **1** could be applied in the formal total synthesis of (±)-aspidofractinine.^3g^ A less explored strategy (II) involves transition-metal-catalyzed imidoylative cross-coupling,^10^ which proceeds via imidoylpalladium intermediate **8** (Scheme 2a).^5^ The third strategy (III) proceeds via heteroallene **9**, which can be accessed via selective transition-metal-catalyzed carbene (Y = CR^6^) or nitrene transfer (Y = N) to the isocyanide moiety,^11^ followed by nucleophilic addition of the C3-position of the indole to the heteroallene (Scheme 2a).^4^ Although one base-metal-catalyzed example is reported for the nitrene transfer to isocyanide **1**,^4b^ no base-metal-catalyzed carbene transfers to 3-(2-isocyanoethyl)indoles (**1**) have been reported.

In 2020, Chen and co-workers reported the dearomative spirocyclization of isocyanides **1** using strategy III, proceeding via ketenimine intermediate **9** (Y = CR^6^, Scheme 2a).^4a^ They described the Pd-catalyzed carbene transfer to isocyanide **1** in the construction of spiroindolenine **15** and polycyclic spiroindolines **16** (Scheme 2b). Although this method displays a broad scope, a high loading of the precious palladium catalyst (10–15%) is required. In addition, despite obtaining pentacyclic scaffold **17** (resembling the core of monoterpenoid indole alkaloids), the authors could not obtain the correct relative stereochemistry at the C–E ring junction, which should be *cis*-fused as in, e.g., aspidospermidine (**3**, Scheme 1b).

**Scheme 3:**
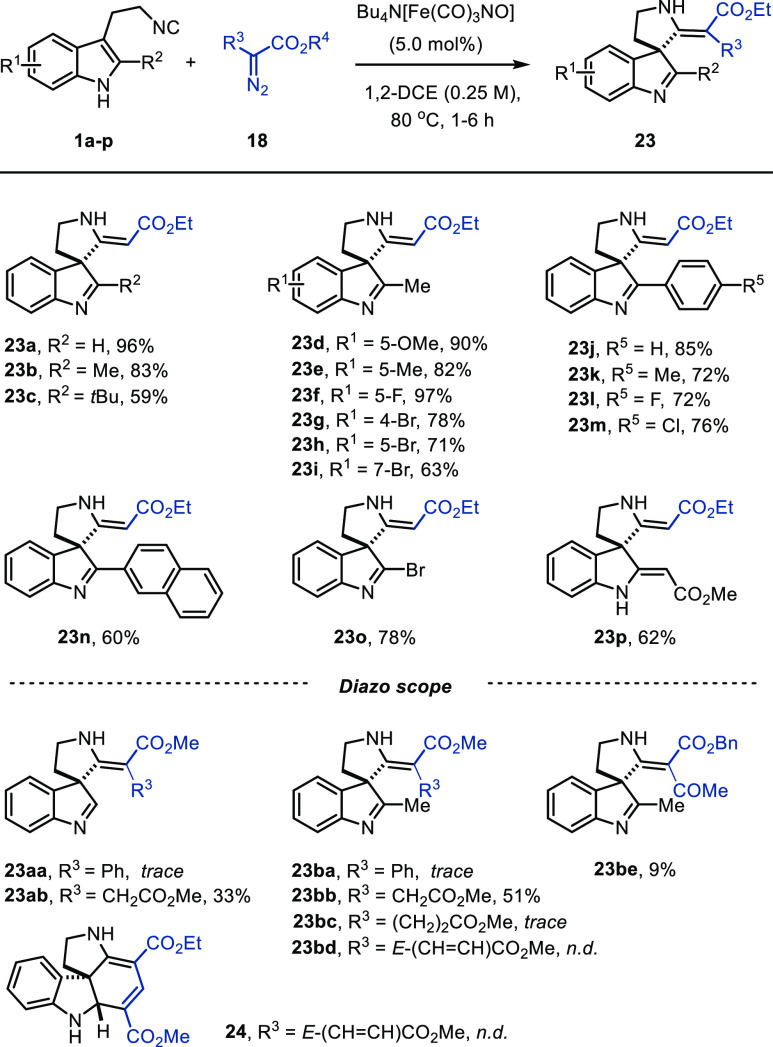
Scope for C2-Substituted 3-(2-Isocyanoethyl)indoles and Substituted α-Diazo Esters Reaction conditions: Bu_4_N[Fe(CO)_3_NO] (0.025 mmol), **1** (0.5 mmol), and **18** (0.6 mmol) in DCE (2 mL) at 80 °C under N_2_.

Shifting from Pd-catalyzed processes to base metals, such as iron, is highly desired, due to their high abundancy on Earth and low cost. Recently, our group developed an iron-catalyzed carbene transfer reaction to isocyanides for the construction of multiple heterocycles.^12^ The ferrate complex, Bu_4_N[Fe(CO)_3_NO] (also known as the Hieber anion),^13^ was demonstrated to effectively catalyze the transfer of carbenes^14^ to isocyanides to give a ketenimine intermediate. In this work, we demonstrate for the first time that the Hieber anion can be employed to catalyze a dearomative spirocyclization of 3-(2-isocyanoethyl)indoles (**1**). The process proceeds via carbene transfer to the isocyanide moiety (Scheme 3b) to afford spiroindolenines **19** as potential synthetic intermediates in the total synthesis of indole alkaloids.

## Results and Discussion

We started our investigation using isocyanide **1a** and ethyl diazoacetate (**22**) as model reactants for optimization (Table 1). Various iron-based catalysts (entries 1–6) were found to be inferior to the Bu_4_N[Fe(CO)_3_NO] as a catalyst of the reaction (entry 7). The addition of phosphine ligands negatively affected the reaction (entries 8 and 9).

**Table 1:**
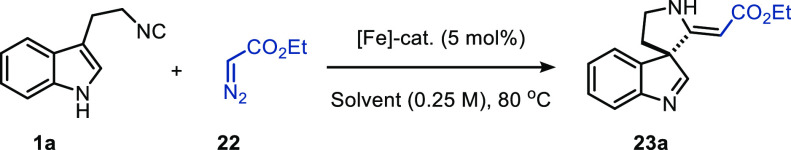
Optimization of the Fe-Catalyzed Carbene Transfer to 3-(2-Isocyanoethyl)indole **1a**

Entry	[Fe]-cat.	Additive (mol %)	Solvent	Yield of **23a** (%)^a^
1	Fe(CO)_5_		DCE	92
2	Fe (*Pc*)		DCE	21^c^
3	Fe(TPP)Cl		DCE	18^c^
4	Fe(TPP)Cl	Zn (50)	DCE	trace^c^
5	Fe(ClO_4_)_2_·4H_2_O	TMEDA (6) NaBarF (6)	DCE	trace^d^
6	Fe(ClO_4_)_2_·4H_2_O	DPPE (6) NaBarF (6)	DCE	trace^d^
7	Bu_4_N[Fe(CO)_3_NO]		DCE	98 (96^b^)
8	Bu_4_N[Fe(CO)_3_NO]	PPh_3_ (6)	DCE	89
9	Bu_4_N[Fe(CO)_3_NO]	P(2-Fur)_3_ (6)	DCE	86
10	Bu_4_N[Fe(CO)_3_NO]		DCE	22^d^,^e^
11			DCE	0
12	Bu_4_N[Fe(CO)_3_NO]		dioxane	70
13	Bu_4_N[Fe(CO)_3_NO]		CH_3_CN	89
14	Bu_4_N[Fe(CO)_3_NO]		PhMe	66
15	Bu_4_N[Fe(CO)_3_NO]		DMF	85
16	Bu_4_N[Fe(CO)_3_NO]		*i*-PrOH	56

a Reactions performed on a 0.5 mmol scale of **1a** and 0.6 mmol of **22**. Yields are determined by ^1^H NMR analysis using 2,5-dimethylfuran as internal standard.

b Isolated yield.

c Full conversion of ethyl diazoacetate (**22**) prior to full conversion of isocyanide **1a**.

d No full conversion of isocyanide **1a** observed by TLC analysis after 22–24 h at 80 °C.

e Reaction performed at 60 °C.

Furthermore, performing the reaction at lower temperature afforded the product in low yield with slow conversion (entry 10). In addition, the reaction was found to proceed in several solvents (entries 12–16), albeit not as efficiently as in DCE. Thus, we opted to continue with the conditions in entry 7, affording spiroindolenine **23a** in 96% isolated yield.

With the optimal conditions in hand, we started to investigate the scope of the Fe-catalyzed carbene transfer/spirocyclization cascade with regard to C2-substituted indole isocyanides **1b**–**p** (Scheme 3). Aliphatic substituents were generally well tolerated, affording indolenine **23b** (R^2^ = Me) in good yield. A slower conversion and lower yields were observed with increasing bulk of the aliphatic substituent (**23c**, R^2^ = *t-*Bu, Scheme 3). Decoration of the indole benzene ring with several substituents at different positions afforded spiroindolenines **23d**–**i** in good to excellent yield. In addition, the reaction allowed the presence of aromatic indole substituents (R^2^) including a 2-naphthyl group, and the corresponding indolenines (**23j**–**n**) were obtained in good yield. To our delight, even the use of a 2-bromoindole isocyanide **1o** (R^2^ = Br) afforded **23o** in good yield, providing an imidoyl halide as a functional handle at the C2-position.^15^ In addition, the tautomerized bis-β-enamino ester **23p** was obtained in moderate yield starting from 2-(2-methoxy-2-oxoethyl)indole isocyanide **1p** (R^2^ = CH_2_CO_2_Me).

After investigation of the isocyanide scope, the scope of diazo compounds was briefly explored (Scheme 3). We started with the use of diazo precursors for donor–acceptor carbenes (**18a**, R^3^ = Ph, R^4^ = Me), which afforded the products **23aa** and **23ba** in only trace amounts. In contrast, in the analogous Pd-catalyzed reaction, these carbenes were converted to indolenines **23aa** and **23ba** in good to excellent yield.^4a^ A similar limitation in scope of α-diazo esters was observed in the recently reported iron-catalyzed intermolecular carbene transfer to isocyanides, where we used amidines to trap the ketenimine intermediate.^12^ Next, we employed diethyl 2-diazosuccinate (**18b**) of the acceptor-type carbene class, which was reacted with isocyanides **1a** and **1b** to give the corresponding spiroindolenines **23ab** and **23bb** in moderate yield. Extending the carbon chain to diethyl 2-diazoglutarate (**18c**) afforded only a trace amount of product **23bc** as judged by ^1^H NMR analysis of the crude product. In addition, the use of α-diazo ester **18e** (R^3^**=** COMe, R^4^ = Me) from the acceptor–acceptor class did afford spiroindolenine **23be**, albeit in low yield. Finally, we employed α-diazo ester **18d** (R^3^**=***E*-CH = CHCO_2_Me, R^4^ = Me) in combination with isocyanide **1a**, which would allow for a carbene transfer/spirocyclization/Mannich cascade affording tetracyclic spiroindoline **24** as described by Chen et al. (Scheme 1b).^4a^ Unfortunately, with [Fe(CO)_3_NO]Bu_4_N as catalyst this cascade did not occur.

**Scheme 4:**
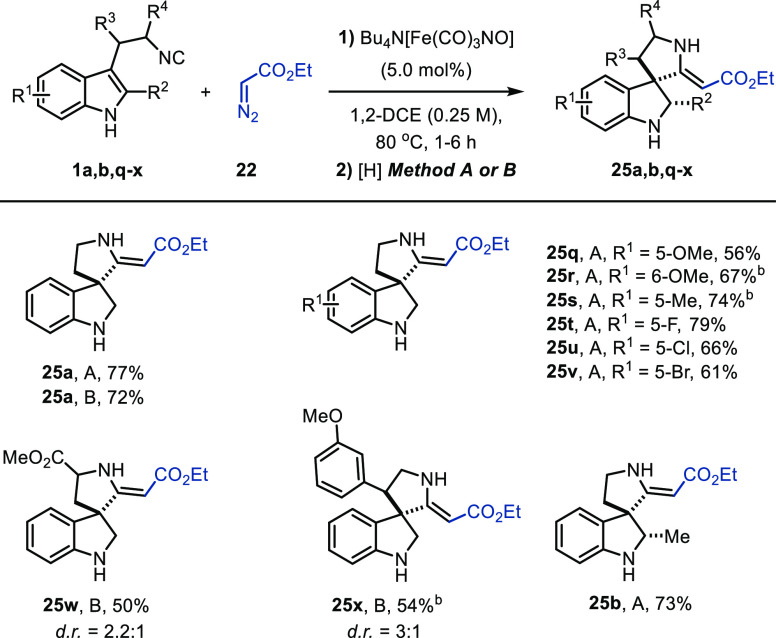
Scope for C2-Unsubstituted 3-(2-Isocyanoethyl)indoles Reaction conditions: Bu_4_N[Fe(CO)_3_NO] (0.025 mmol), **1** (0.5 mmol), and **22** (0.6 mmol) in 1,2-DCE (2 mL) at 80 °C under N_2_ until full conversion of **1**. Method A: Solution was cooled to 0 °C and diluted with MeOH (2 mL), and NaBH_4_ (0.525 mmol) was added. Method B: Solution was cooled to 0 °C, and MeOH (2 mL). NaBH_3_CN (0.525 mmol) and a few drops of AcOH were added. Extra portion(s) of reducing agent (NaBH_4_/NaBH_3_CN) added to reach full conversion of indolenine intermediate **23** observed on TLC.

In addition to the isocyanide scope bearing a C2 indole substituent, we explored the scope of the C2-unsubstituted isocyanides, where R^2^ = H (Scheme 4). Based on previous work,^3g,16^ we envisioned that the corresponding spiroindolines, containing an imine functionality, are relatively less stable compared to their corresponding C2-substituted counterparts. Fortunately, the obtained spiroindolenine **25a** with the benchmark substrate (**1a**) is relatively stable upon isolation and column chromatography. However, the stability of the spiroindolenine derived from isocyanides **1q**–**x** differs significantly depending on the substitution pattern on the indole moiety (R^1^). For example, the spiroindolenine derived from isocyanide **1q** (R^1^ = 5-OMe) could not be isolated and fully degraded upon isolation. Therefore, we decided to *in situ* transform all C2-unsubstituted spiroindolenines **23** to the more stable spiroindolines **25q**–**x** via a one-pot spirocyclization/reduction sequence (Scheme 4). After a brief optimization (Table S3) we were able to isolate benchmark spiroindoline **25a** in 77% yield using NaBH_4_ as the reducing agent (method A). Various C5-substituted indole isocyanides (**1q**–**v**) were converted to the corresponding spiroindolines **25q**–**v** in moderate to good yield (Scheme 4). Next, we investigated tryptamine-derived isocyanides bearing a substituent on the ethylene linker (**1w**, **1x**). Initially, low yields were observed for spiroindolines **25w** and **25x** employing NaBH_4_ reductant (method A). Gratifyingly, changing to slightly different conditions (method B) using NaBH_3_CN as the hydride source, spiroindolenines **25w** and **25x** could be isolated in reasonable yield.

Conversion of **1w** to **25w** proceeded with moderate diastereoselectivity (2.2:1 dr). A slightly higher stereoinduction (3:1 dr) was observed for **25x**. Advantageously, when C2-methyl-substituted isocyanide **1b** was employed in the one-pot sequence, spiroindoline **25b** was obtained as a single diastereomer. Based on literature precedent,^3g^ the relative stereochemistry was assumed to proceed with the hydride approaching from the least hindered face.

**Scheme 5:**
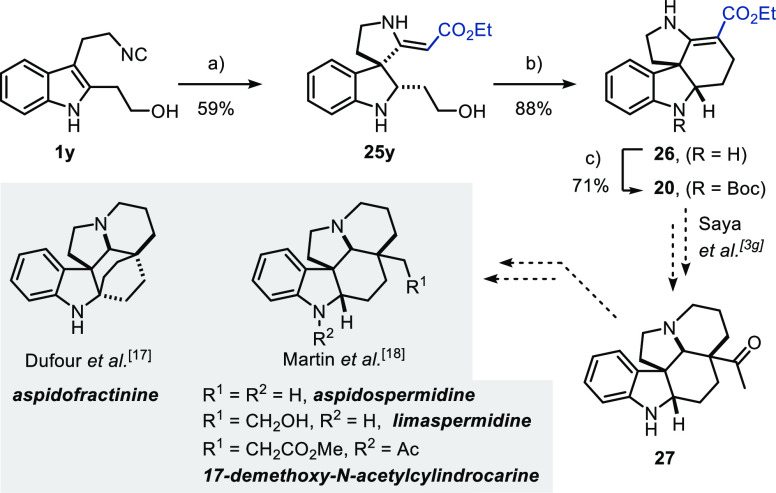
Application of Fe-Catalyzed Carbene Transfer/Spirocyclization Cascade in Formal Total Synthesis Reaction conditions: (a) Bu_4_N[Fe(CO)_3_NO] (0.63 mmol), **1y** (6.3 mmol), and **22** (7.6 mmol) in DCE (25 mL), 80 °C; then NaBH_4_, MeOH, 0 °C; (b) imidazole (1.35 equiv), **25y** (1.0 equiv), PPh_3_ (1.30 equiv), I_2_ (1.30 equiv), rt, CH_2_Cl_2_, 1 h; (c) **26** (1.0 equiv), Boc_2_O (10.5 equiv), DMAP (0.4 equiv), 72 h.

In order to show the utility of the Fe-catalyzed carbene transfer/spirocyclization cascade methodology, we investigated the conversion of a suitably functionalized isocyanide to the core scaffold of monoterpene indole alkaloids (Scheme 5). To our delight, isocyanide **1y** could be subjected to the one-pot spirocyclization/reduction sequence as the free alcohol, affording spiroindoline **25y** in 66% yield as a single diastereomer on a 6.3 mmol scale. Next, the alcohol in **25y** was converted to the corresponding iodide, which under the reactions conditions immediately cyclized to afford tetracycle **26** in excellent yield. Subsequent Boc-protection results in the desired scaffold **20**, which can be transformed into pentacyclic 19-oxoaspidospermidine (**27**) as demonstrated by Saya et al.^3g^ Further, Dufour et al. demonstrated that scaffold **27** can be transformed into (±)-aspidofractinine,^17^ while more recently, Martin et al. also reported the conversion of indoline **27** to (±)-limaspermidine, (±)-aspidospermidine, and (±)-17-demethoxy-*N*-acetylcylindrocarine.^18^

In conclusion, we report the use of Bu_4_N[Fe(CO)_3_NO] as catalyst in the carbene transfer/dearomative spirocyclization cascade toward spiroindolenines. In addition, the corresponding spiroindolines could be obtained via a one-pot reduction sequence. In general, the reaction displays a high functional group tolerance for the isocyanide **1**. However, the Bu_4_N[Fe(CO)_3_NO]-catalyzed reaction is less tolerant of α-diazo ester input compared to the Pd-catalyzed reaction developed by Chen and co-workers.^4a^ Nonetheless, using a carefully chosen C2-prefunctionalized 3-(2-isocyanoethyl)indole, we were able to apply the Bu_4_N[Fe(CO)_3_NO]-catalyzed carbene transfer/dearomative spirocyclization/reduction sequence in the formal total synthesis of the monoterpene indole alkaloids (±)-aspidofractinine, (±)-limaspermidine, (±)-aspidospermidine, and (±)-17-demethoxy-*N*-acetylcylindrocarine.

## Experimental Section

### General Information

Unless stated otherwise, all solvents and commercially available reagents were used as purchased. Anhydrous dichloromethane, THF, DMF, and toluene were obtained via the PureSolv MD 5 Solvent Purification System. All other solvents were used as purchased from the corresponding supplier. Diazo compounds used in this work were either obtained commercially or synthesized according to the corresponding literature procedures. ***Caution!*** It should be noted that diazo compounds can be potentially explosive. Correct safety measures, such as the scale of the reaction, and careful handling are required. Use of appropriate safety gear, including a blast shield, is strongly recommended. Nuclear magnetic resonance (NMR) spectra were recorded on a Bruker Avance 600 MHz (150 MHz for ^13^C), Bruker Avance 500 MHz (126 MHz for ^13^C), and (470 MHz for ^19^F) or Bruker Avance 300 MHz (75.4 MHz for ^13^C) using the residual solvent as internal standard (^1^H: δ 7.26 ppm, ^13^C {^1^H}: δ 77.16 ppm for CDCl_3_, ^1^H: δ 2.50 ppm, ^13^C{^1^H}: δ 39.52 ppm for DMSO-*d*_6_). Chemical shifts (δ) are given in ppm, and coupling constants (*J*) are quoted in hertz (Hz). Resonances are described as s (singlet), d (doublet), t (triplet), q (quartet), quint (quintet), sex (sextet), sep (septet), br (broad singlet), and m (multiplet) or combinations thereof. Electrospray ionization (ESI) high-resolution mass spectrometry was carried out using a Bruker QTOF impact II instrument in positive-ion mode (capillary potential of 4500 V). Flash chromatography was performed on Silicycle Silia-P flash silica gel (particle size 40–63 μm, pore diameter 60 Å) using the indicated eluent. Thin-layer chromatography (TLC) was performed using TLC plates from Merck (SiO_2_, Kieselgel 60 F254 neutral, on aluminum with fluorescence indicator), and compounds were visualized by UV detection (254 nm) and KMnO_4_ stain. SFC-MS analysis was conducted using a Shimadzu Nexera SFC-MS equipped with a Nexera X2 SIL-30AC autosampler, Nexera UC LC-30AD SF CO_2_ pump, Nexera X2 LC-30AD liquid chromatograph, Nexera UC SFC-30A back pressure regulator, prominence SPD-M20A diode array detector, prominence CTO-20AC column oven, and CBM-20A system controller. A gradient of supercritical CO_2_ (A) and methanol (B) was used. Method: 2% B/98% A → 100% B/0% A over the course of 7 min. The flow was maintained at 2.0 mL/min, and the sample injection volume was 5 μL. Mass spectrometry analyses were performed using a Shimadzu LCMS-2020 mass spectrometer. The data were acquired in full-scan APCI mode (MS) from *m*/*z* 100 to 800 in positive ionization mode. Data was processed using Shimadzu Labsolutions 5.82.

### General Procedure A: Synthesis of Spiroindolenines **23**

To a flame-dried Schlenk flask under N_2_ atmosphere, charged with a stirring bean, was added Bu_4_N[Fe(CO)_3_NO] (10.3 mg, 0.025 mmol, 0.05 equiv). Subsequently, 1,2-DCE was added (2 mL), and the mixture was stirred until the catalyst was dissolved. This was followed by the addition of tryptamine-derived isocyanide (0.5 mmol, 1.0 equiv) and ethyl diazoacetate (**22**) (0.6 mmol, 1.2 equiv). The solution was placed in a preheated oil bath and stirred at 80 °C until full conversion of the isocyanide was observed on TLC. Subsequently, the reaction mixture cooled to room temperature and directly subjected to purification by flash column chromatography, using a mixture of EtOAc:*c*Hex as eluent.

### General Procedure B: Synthesis of Spiroindolines **25**

To a flame-dried Schlenk flask under N_2_ atmosphere, charged with a stirring bean, was added Bu_4_N[Fe(CO)_3_NO] (0.05 equiv). Subsequently, 1,2-DCE was added (0.25 M), and the mixture was stirred until the catalyst was dissolved. This was followed by the addition of tryptamine-derived isocyanide (0.5 mmol, 1.0 equiv) and ethyl diazoacetate (**22**) (0.6 mmol, 1.2 equiv). The solution was placed in a preheated oil bath and stirred at 80 °C until full conversion of the isocyanide was observed on TLC. Subsequently, the reaction mixture was cooled to 0 °C and diluted with MeOH to a concentration of 0.125 M, after which NaBH_4_ (1.05 equiv) was added. The reaction was stirred at 0 °C until full conversion of indolenine intermediate **23** was observed on TLC. Afterward, the reaction mixture was quenched with saturated aqueous NH_4_Cl solution and stirred vigorously for 15 min. The aqueous layer was extracted with CH_2_Cl_2_ (3×), and the organic layers were collected, washed with brine, dried over Na_2_SO_4_, and filtered. The filtrate was collected and concentrated in vacuo. Subsequently, the crude product was subjected to flash column chromatography, using a mixture of EtOAc:*c*Hex as eluent, to obtain the pure title compound.

### General Procedure C: Synthesis of Spiroindolines **23** for Diazo Scope

To a flame-dried Schlenk flask under N_2_ atmosphere, charged with a stirring bean, was added Bu_4_N[Fe(CO)_3_NO] (20.6 mg, 0.025 mmol, 0.05 equiv). Subsequently, 1,2-DCE was added (2 mL), and the mixture was stirred until the catalyst was dissolved. This was followed by the addition of tryptamine-derived isocyanide (0.5 mmol, 1.0 equiv) and α-diazoacetate (**18**) (0.6 mmol, 1.2 equiv). The solution was placed in a preheated oil bath and stirred at 80 °C for 22–24 h. Subsequently, the reaction mixture was cooled to room temperature and directly purified via flash column chromatography using a mixture of EtOAc:*c*Hex as eluent to provide the title compound.

#### Ethyl (*Z*)-2-(Spiro[indole-3,3′-pyrrolidin]-2′-ylidene)acetate (**23a**)

Ethyl (*Z*)-2-(spiro[indole-3,3′-pyrrolidin]-2′-ylidene)acetate was prepared according to general procedure A starting from 3-(2-isocyanoethyl)-1*H*-indole (85.3 mg, 0.50 mmol, 1.0 equiv). The title compound was isolated via FCC using EtOAc:*c*Hex +5% Et_3_N as eluent to obtain the title compound as a light-yellow oil (124 mg, 0.48 mmol, 96%). *R*_*f*_ = 0.30 (EtOAc:*c*Hex = 1:9 + 5% Et_3_N); ^1^H NMR (600 MHz, CDCl_3_) δ 8.11 (s, 1H), 7.99 (s, 1H), 7.63 (d, *J* = 7.7 Hz, 1H), 7.37 (td, *J* = 7.5, 1.5 Hz, 1H), 7.31–7.24 (m, 2H), 4.06–3.97 (m, 2H), 3.94 (s, 1H), 3.88 (dddd, *J* = 10.2, 7.8, 5.0, 1.0 Hz, 1H), 3.85–3.79 (m, 1H), 2.43 (ddd, *J* = 12.5, 7.4, 4.9 Hz, 1H), 2.31 (ddd, *J* = 12.9, 7.8, 6.7 Hz, 1H), 1.15 (t, *J* = 7.1 Hz, 3H) ppm; ^13^C{^1^H} NMR (150 MHz, CDCl_3_): δ 171.9 (CH), 170.5 (C_q_), 162.0 (C_q_), 155.6 (C_q_), 140.1 (C_q_), 128.9 (CH), 127.3 (CH), 122.3 (CH), 121.5 (CH), 77.4 (CH), 67.2 (C_q_), 58.9 (CH_2_), 46.0 (CH_2_), 30.5 (CH_2_), 14.5 (CH_3_) ppm; HRMS (ESI): *m*/*z* calculated for C_15_H_17_N_2_O_2_ [M+H^+^] = 257.1285, found = 257.1281.

#### Ethyl (*Z*)-2-(2-Methylspiro[indole-3,3′-pyrrolidin]-2′-ylidene)acetate (**23b**)

Ethyl (*Z*)-2-(2-methylspiro[indole-3,3′-pyrrolidin]-2′-ylidene)acetate was prepared according to general procedure A starting from 3-(2-isocyanoethyl)-2-methyl-1*H*-indole (91.4 mg, 0.50 mmol, 1.0 equiv). The title compound was isolated as a yellow solid (112 mg, 0.41 mmol, 83%). *R*_*f*_ = 0.30 (cyclohexane:EtOAc 3:2); ^1^H NMR (500 MHz, CDCl_3_): δ 8.14 (s, 1H), 7.51 (d, *J* = 7.7 Hz, 1H), 7.32 (td, *J* = 7.5, 1.3 Hz, 1H), 7.23 (d, *J* = 6.9 Hz, 1H), 7.17 (t, *J* = 7.2 Hz, 1H), 4.01 (q, *J* = 7.1 Hz, 2H), 3.94–3.79 (m, 2H), 3.88 (s, 1H), 2.39–2.29 (m, 2H), 2.27 (s, 3H), 1.16 (t, *J* = 7.1 Hz, 3H) ppm; ^13^C{^1^H} NMR (126 MHz, CDCl_3_): δ 181.8 (C_q_), 170.8 (C_q_), 164.2 (C_q_), 155.2 (C_q_), 142.1 (C_q_), 128.8 (CH), 126.2 (CH), 122.1 (CH), 120.2 (CH), 77.1 (CH), 67.7 (C_q_), 58.9 (CH_2_), 46.0 (CH_2_), 31.6 (CH_2_), 16.4 (CH_3_), 14.5 (CH_3_) ppm. HRMS (ESI): *m*/*z* calculated for C_16_H_19_N_2_O_2_ [M+H^+^] = 271.1441, found = 271.1446.

#### Ethyl (*Z*)-2-(2-(*tert*-Butyl)spiro[indole-3,3′-pyrrolidin]-2′-ylidene)acetate (**23c**)

Ethyl (Z)-2-(2-(*tert*-butyl)spiro[indole-3,3′-pyrrolidin]-2′-ylidene)acetate was prepared according to general procedure A starting from 2-(*tert*-butyl)-3-(2-isocyanoethyl)-1*H*-indole (113.2 mg, 0.50 mmol, 1.0 equiv). The title compound was isolated as a white solid (92.0 mg, 0.294 mmol, 59%). *R*_*f*_ = 0.35 (cyclohexane:EtOAc 4:1); ^1^H NMR (500 MHz, CDCl_3_): δ 8.22 (s, 1H), 7.54 (dz, *J* = 7.7 Hz, 1H), 7.30 (ddd, *J* = 7.8, 5.1, 3.7 Hz, 1H), 7.18–7.14 (m, 2H), 4.06–3.91 (m, 5H), 2.89 (ddd, *J* = 13.5, 9.3, 7.7 Hz, 1H), 2.23 (ddd, *J* = 13.5, 8.0, 3.6 Hz, 1H).1.41 (s, 9H), 1.16 (t, *J* = 7.1 Hz, 3H) ppm; ^13^C{^1^H} NMR (126 MHz, CDCl_3_): δ 190.3 (C_q_), 170.8 (C_q_), 164.7 (C_q_), 153.6 (C_q_), 144.6 (C_q_), 128.5 (CH), 126.4 (CH), 120.9 (CH), 120.3 (CH), 76.8 (CH), 68.1 (C_q_), 58.8 (CH_2_), 46.3 (CH_2_), 38.1 (C_q_), 30.3 (CH_3_), 30.3 (CH_2_), 14.6 (CH_3_) ppm. HRMS (ESI): *m*/*z* calculated for C_19_H_25_N_2_O_2_ [M+H^+^] = 313.1911, found = 313.1914.

#### Ethyl (*Z*)-2-(5-Methoxy-2-methylspiro[indole-3,3′-pyrrolidin]-2′-ylidene)acetate (**23d**)

Ethyl (*Z*)-2-(5-methoxy-2-methylspiro[indole-3,3′-pyrrolidin]-2′-ylidene)acetate was prepared according to general procedure A starting from 3-(2-isocyanoethyl)-5-methoxy-2-methyl-1*H*-indole (107.3 mg, 0.50 mmol, 1.0 equiv). The title compound was isolated as a yellow waxy solid (136 mg, 0.45 mmol, 90%). *R*_*f*_ = 0.19 (cyclohexane:EtOAc 2:1); ^1^H NMR (500 MHz, CDCl_3_): δ 8.12 (s, 1H), 7.41 (d, *J* = 8.5 Hz, 1H), 6.83 (dd, *J* = 8.4, 2.5 Hz, 1H), 6.79 (d, *J* = 2.5 Hz, 1H), 4.03 (q, *J* = 7.1 Hz, 2H), 3.93–3.80 (m, 2H), 3.91 (s, 1H), 3.79 (s, 3H), 2.41–2.25 (m, 2H), 2.24 (s, 3H), 1.17 (t, *J* = 7.1 Hz, 3H) ppm; ^13^C{^1^H} NMR (126 MHz, CDCl_3_): δ 179.7 (C_q_), 170.9 (C_q_), 164.3 (C_q_), 158.6 (C_q_), 148.7 (C_q_), 143.6 (C_q_), 120.5 (CH), 113.3 (CH), 108.9 (CH), 77.3 (CH), 68.0 (C_q_), 59.0 (CH_2_), 55.8 (CH_3_), 46.0 (CH_2_), 31.8 (CH_2_) 16.3 (CH_3_), 14.6 (CH_3_) ppm. HRMS (ESI): *m*/*z* calculated for C_17_H_21_N_2_O_3_ [M+H^+^] = 301.1547, found = 301.1552.

#### Ethyl (*Z*)-2-(2,5-Dimethylspiro[indole-3,3′-pyrrolidin]-2′-ylidene)acetate (**23e**)

Ethyl (*Z*)-2-(2,5-dimethylspiro[indole-3,3′-pyrrolidin]-2′-ylidene)acetate was prepared according to general procedure A starting from 3-(2-isocyanoethyl)-2,5-dimethyl-1*H*-indole (99.5 mg, 0.50 mmol, 1.0 equiv). The title compound was isolated as a yellow waxy solid (116 mg, 0.41 mmol, 82%). *R*_*f*_ = 0.24 (*c*Hex:EtOAc 2:1); ^1^H NMR (500 MHz, CDCl_3_): δ 8.14 (s, 1H), 7.39 (d, *J* = 7.8 Hz, 1H), 7.12 (d, *J* = 7.8 Hz, 1H), 7.05 (s, 1H), 4.03 (q, *J* = 7.1 Hz, 2H), 3.94–3.81 (m, 2H), 3.90 (s, 1H), 2.39–2.27 (m, 2H), 2.35 (s, 3H), 2.26 (s, 3H), 1.17 (t, *J* = 7.1 Hz, 3H) ppm; ^13^C{^1^H} NMR (126 MHz, CDCl_3_): δ 180.8 (C_q_), 170.8 (C_q_), 164.5 (C_q_), 152.9 (C_q_), 142.3 (C_q_), 136.1 (C_q_), 129.3 (CH), 122.9 (CH), 119.8 (CH), 77.1 (CH), 67.7 (C_q_), 58.9 (CH_2_), 46.0 (CH_2_), 31.7 (CH_2_), 21.5 (CH_3_), 16.3 (CH_3_), 14.6 (CH_3_) ppm. HRMS (ESI): *m*/*z* calculated for C_17_H_21_N_2_O_2_ [M+H^+^] = 285.1598, found = 285.1603.

#### Ethyl (*Z*)-2-(5-Fluoro-2-methylspiro[indole-3,3′-pyrrolidin]-2′-ylidene)acetate (**23f**)

Ethyl (*Z*)-2-(5-fluoro-2-methylspiro[indole-3,3′-pyrrolidin]-2′-ylidene)acetate was prepared according to general procedure A starting from 5-fluoro-3-(2-isocyanoethyl)-2-methyl-1*H*-indole (101.3 mg, 0.5 mmol, 1.0 equiv). The title compound was isolated as a light-yellow solid (140 mg, 0.49 mmol, 97%). *R*_*f*_ = 0.23 (cyclohexane:EtOAc 3:2); ^1^H NMR (500 MHz, CDCl_3_): δ 8.13 (s, 1H), 7.44 (dd, *J* = 8.4, 4.6 Hz, 1H), 7.01 (td, *J* = 8.6, 2.5 Hz, 1H), 6.95 (dd, *J* = 7.8, 2.6 Hz, 1H), 4.03 (q, *J* = 7.1 Hz, 2H), 3.94–3.80 (m, 2H), 3.89 (s, 1H), 2.41–2.27 (m, 2H), 2.26 (s, 3H), 1.17 (t, *J* = 7.1 Hz, 3H) ppm; ^13^C{^1^H} NMR (126 MHz, CDCl_3_): δ 181.7 (C_q_, d, *J* = 3.6 Hz), 170.7 (C_q_), 163.4 (C_q_), 161.5 (d, *J* = 245.2 Hz, C_q_), 151.2 (C_q_, d, *J* = 2.3 Hz), 143.9 (C_q_, d, *J* = 8.8 Hz), 120.9 (CH, d, *J* = 8.8 Hz), 115.4 (CH, d, *J* = 23.6 Hz), 110.1 (CH, d, *J* = 25.1 Hz), 77.3 (CH), 68.2 (C_q_, d, *J* = 2.3 Hz), 59.0 (CH_2_), 45.9 (CH_2_), 31.6 (CH_2_), 16.3 (CH_3_), 14.5 (CH_3_) ppm; ^**19**^F{^1^H} NMR (470.4 MHz, CDCl_3_): δ −115.90 ppm; HRMS (ESI): *m*/*z* calculated for C_16_H_18_FN_2_O_2_ [M+H^+^] = 289.1347, found = 289.1356.

#### Ethyl (*Z*)-2-(4-**b**romo-2-methylspiro[indole-3,3′-pyrrolidin]-2′-ylidene)acetate (**23g**)

Ethyl (*Z*)-2-(4-bromo-2-methylspiro[indole-3,3′-pyrrolidin]-2′-ylidene)acetate was prepared according to general procedure A starting from 4-bromo-3-(2-isocyanoethyl)-2-methyl-1*H*-indole (132.0 mg, 0.5 mmol, 1.0 equiv). The title compound was isolated as light brown solid (136 mg, 0.39 mmol, 78%). *R*_*f*_ = 0.30 (EtOAc:*c*Hex = 1:5); ^1^H NMR (500 MHz, CDCl_3_): δ 8.19 (s, 1H), 7.43 (d, *J* = 7.6 Hz, 1H), 7.28 (d, *J* = 8.1 Hz, 1H), 7.18 (t, *J* = 7.8 Hz, 1H), 4.08–3.95 (m, 3H), 3.91–3.81 (m, 2H), 2.86 (ddd, *J* = 13.8, 9.7, 7.5 Hz, 1H), 2.23 (s, 3H), 2.12 (ddd, *J* = 13.8, 8.4, 3.4 Hz, 1H), 1.15 (t, *J* = 7.1 Hz, 3H) ppm. ^13^C{^1^H} NMR (126 MHz, CDCl_3_): δ 183.3 (C_q_), 170.8 (C_q_), 161.4 (C_q_), 157.4 (C_q_), 140.1 (C_q_), 130.5 (CH), 129.8 (CH), 119.3 (CH), 118.0 (C_q_), 76.6 (CH), 69.8 (C_q_), 59.0 (CH_2_), 46.4 (CH_2_), 26.7 (CH_2_), 16.2 (CH_3_), 14.6 (CH_3_) ppm. HRMS (ESI): *m*/*z* calculated for C_16_H_18_BrN_2_O_2_ [M+H^+^] = 349.0546, found = 349.0555.

#### Ethyl (*Z*)-2-(5-Bromo-2-methylspiro[indole-3,3′-pyrrolidin]-2′-ylidene)acetate (**23h**)

Ethyl (*Z*)-2-(5-bromo-2-methylspiro[indole-3,3′-pyrrolidin]-2′-ylidene)acetate was prepared according to general procedure A starting from 5-bromo-3-(2-isocyanoethyl)-2-methyl-1*H*-indole (132.0 mg, 0.5 mmol, 1.0 equiv). The title compound was isolated as a yellow solid (124 mg, 0.35 mmol, 71%). *R*_*f*_ = 0.23 (cyclohexane:EtOAc 2:1); ^1^H NMR (500 MHz, CDCl_3_): δ 8.12 (s, 1H), 7.45 (dd, *J* = 8.2, 1.9 Hz, 1H), 7.37 (d, *J* = 8.3 Hz, 1H), 7.36 (d, *J* = 1.9 Hz, 1H), 4.03 (qd, *J* = 7.1, 1.4 Hz, 2H), 3.93–3.79 (m, 2H), 3.88 (s, 1H), 2.40–2.27 (m, 2H), 2.26 (s, 3H), 1.17 (t, *J* = 7.1 Hz, 3H) ppm; ^13^C{^1^H} NMR (126 MHz, CDCl_3_): δ 182.4 (C_q_), 170.6 (C_q_), 163.1 (C_q_), 154.2 (C_q_), 144.2 (C_q_), 131.9 (CH), 125.6 (CH), 121.6 (CH), 119.7 (C_q_), 77.4 (CH), 66.1 (C_q_), 59.1 (CH_2_), 45.9 (CH_2_), 31.5 (CH_2_), 16.4 (CH_3_), 14.5 (CH_3_) ppm; HRMS (ESI): *m*/*z* calculated for C_16_H_18_BrN_2_O_2_ [M+H^+^] = 349.0546, found = 349.0553.

#### Ethyl (*Z*)-2-(7-Bromo-2-methylspiro[indole-3,3′-pyrrolidin]-2′-ylidene)acetate (**23i**)

Ethyl (*Z*)-2-(7-bromo-2-methylspiro[indole-3,3′-pyrrolidin]-2′-ylidene)acetate was prepared according to general procedure A starting from 7-bromo-3-(2-isocyanoethyl)-2-methyl-1*H*-indole (132.0 mg, 0.5 mmol, 1.0 equiv). The title compound was isolated as brown solid (110 mg, 0.31 mmol, 63%). *R*_*f*_ = 0.23 (cyclohexane:EtOAc 2:1); ^1^H NMR (500 MHz, CDCl_3_): δ 8.12 (s, 1H), 7.47 (dd, *J* = 7.9, 1.1 Hz, 1H), 7.16 (dd, *J* = 7.4, 1.0 Hz, 1H), 7.05 (t, *J* = 7.7 Hz, 1H), 4.02 (q, *J* = 7.1 Hz, 2H), 3.93–3.81 (m, 3H), 2.43–2.25 (m, 5H), 1.16 (t, *J* = 7.1 Hz, 3H) ppm; ^13^C{^1^H} NMR (126 MHz, CDCl_3_): δ 183.4 (C_q_), 170.7 (C_q_), 163.2 (C_q_), 153.6 (C_q_), 143.8 (C_q_), 132.2 (CH), 127.6 (CH), 121.2 (CH), 113.9 (C_q_), 77.5 (CH), 69.4 (C_q_), 59.0 (CH_2_), 45.9 (CH_2_), 31.6 (CH_2_), 16.6 (CH_3_), 14.5 (CH_3_) ppm; HRMS (ESI): *m*/*z* calculated for C_16_H_18_BrN_2_O_2_ [M+H^+^] = 349.0546, found = 349.0550.

#### Ethyl (*Z*)-2-(2-Phenylspiro[indole-3,3′-pyrrolidin]-2′-ylidene)acetate (**23j**)

Ethyl (*Z*)-2-(2-phenylspiro[indole-3,3′-pyrrolidin]-2′-ylidene)acetate was prepared according to general procedure A starting from 3-(2-isocyanoethyl)-2-phenyl-1*H*-indole (123.2 mg, 0.5 mmol, 1.0 equiv). The title compound was isolated as an off-white solid (141 mg, 0.424 mmol, 85%). *R*_*f*_ = 0.27 (EtOAc:*c*Hex = 1:5); ^1^H NMR (500 MHz, CDCl_3_): δ 8.30 (s, 1H), 7.97 (dd, *J* = 8.0, 1.7 Hz, 2H), 7.71 (d, *J* = 7.8 Hz, 1H), 7.53–7.42 (m, 3H), 7.40 (td, *J* = 7.6, 1.3 Hz, 1H), 7.30 (d, *J* = 7.2 Hz, 1H), 7.24 (td, *J* = 7.4, 1.1 Hz, 1H), 4.08 (s, 1H), 4.07–3.93 (m, 4H), 2.68 (dt, *J* = 13.1, 9.0 Hz, 1H), 2.19 (ddd, *J* = 13.1, 7.3, 2.7 Hz, 1H), 1.14 (t, *J* = 7.1 Hz, 3H) ppm. ^13^C{^1^H} NMR (126 MHz, CDCl_3_): δ 178.0 (C_q_), 171.0 (C_q_), 165.4 (C_q_), 154.0 (C_q_), 144.5 (C_q_), 131.8 (C_q_), 131.2 (CH), 128.9 (2 x CH), 128.8 (CH), 126.8 (CH), 121.4 (CH), 121.3 (CH), 77.7 (CH), 66.6 (C_q_), 59.0 (CH_2_), 46.3 (CH_2_), 33.1 (CH_2_), 14.5 (CH_3_) ppm. HRMS (ESI): *m*/*z* calculated for C_21_H_21_N_2_O_2_ [M+H^+^] 333.1598, found = 333.1604.

#### Ethyl (*Z*)-2-(2-(*p*-Tolyl)spiro[indole-3,3′-pyrrolidin]-2′-ylidene)acetate (**23k**)

Ethyl (*Z*)-2-(2-(*p*-tolyl)spiro[indole-3,3′-pyrrolidin]-2′-ylidene)acetate was prepared according to general procedure A starting from 3-(2-isocyanoethyl)-2-(*p*-tolyl)-1*H*-indole (130.7 mg, 0.5 mmol, 1.0 equiv). The product was purified by flash column chromatography using EtOAc:*c*Hex = (1:4) as eluent to obtain the product as a white solid (125 mg, 0.36 mmol, 72%). *R*_*f*_ = 0.25 (EtOAc:*c*Hex = 1:4); ^1^H NMR (500 MHz, CDCl_3_): δ 8.29 (s, 1H), 7.88 (d, *J* = 8.1 Hz, 2H), 7.70 (d, *J* = 7.7 Hz, 1H), 7.38 (t, *J* = 7.6 Hz, 1H), 7.29 (d, *J* = 7.2 Hz, 1H), 7.26 (d, *J* = 8.0 Hz, 2H), 7.22 (t, *J* = 7.3 Hz, 1H), 4.07 (s, 1H), 4.05–3.94 (m, 4H), 2.67 (dt, *J* = 13.2, 9.0 Hz, 1H), 2.40 (s, 3H), 2.22–2.12 (m, 1H), 1.13 (t, *J* = 7.2 Hz, 3H) ppm; ^13^C{^1^H} NMR (126 MHz, CDCl_3_): δ 178.0 (C_q_), 171.0 (C_q_), 165.5 (C_q_), 154.1 (C_q_), 144.5 (C_q_), 141.7 (C_q_), 129.6 (CH), 129.0 (C_q_), 128.8 (CH), 128.7 (CH), 126.5 (CH), 121.2 (CH), 121.1 (CH), 77.6 (CH), 66.5 (C_q_), 58.9 (CH_2_), 46.2 (CH_2_), 33.3 (CH_2_), 21.7 (CH_3_), 14.5 (CH_3_) ppm.; HRMS (ESI): *m*/*z* calculated for C_22_H_23_N_2_O_2_ [M+H^+^] = 347.1754, found = 347.1758.

#### Ethyl (*Z*)-2-(2-(4-Fluorophenyl)spiro[indole-3,3′-pyrrolidin]-2′-ylidene)acetate (**23l**)

Ethyl (*Z*)-2-(2-(4-fluorophenyl)spiro[indole-3,3′-pyrrolidin]-2′-ylidene)acetate was prepared according to general procedure A starting from 2-(4-fluorophenyl)-3-(2-isocyanoethyl)-1*H*-indole (132.3 mg, 0.5 mmol, 1.0 equiv). The title compound was isolated as a light-yellow solid (125 mg, 0.36 mmol, 72%). *R*_*f*_ = 0.25 (EtOAc:*c*Hex = 1:4); ^1^H NMR (500 MHz, CDCl_3_): δ 8.29 (s, 1H), 7.98 (dd, *J* = 8.8, 5.4 Hz, 2H), 7.69 (d, *J* = 7.7 Hz, 1H), 7.39 (td, *J* = 7.7, 1.0 Hz, 1H), 7.29 (d, *J* = 7.1 Hz, 1H), 7.23 (t, *J* = 7.4 Hz, 1H), 7.13 (t, *J* = 8.6 Hz, 2H), 4.06 (s, 1H), 4.05–3.93 (m, 4H), 2.62 (dt, *J* = 13.2, 9.0 Hz, 1H), 2.22–2.13 (m, 1H), 1.14 (t, *J* = 7.1 Hz, 3H) ppm; ^13^C{^1^H} NMR (126 MHz, CDCl_3_): δ 176.8 (C_q_), 170.9 (C_q_), 165.1 (C_q_), 164.8 (d, *J* = 253.2 Hz, C_q_), 153.8 (C_q_), 144.4 (C_q_), 130.9 (d, *J* = 8.6 Hz, CH), 128.9 (CH), 128.0 (C_q_, d, *J* = 3.3 Hz), 126.7 (CH), 121.3 (2 x CH), 116.0 (d, *J* = 21.8 Hz, CH), 77.8 (CH), 66.5 (C_q_), 59.0 (CH_2_), 46.2 (CH_2_), 33.2 (CH_2_), 14.5 (CH_3_) ppm; ^**19**^F{^1^H} NMR (470 MHz, CDCl_3_): δ −108.27 ppm; HRMS (ESI): *m*/*z* calculated for C_21_H_20_FN_2_O_2_ [M+H^+^] = 351.1503, found = 351.1515.

#### Ethyl (*Z*)-2-(2-(4-Chlorophenyl)spiro[indole-3,3′-pyrrolidin]-2′-ylidene)acetate (**23m**)

Ethyl (*Z*)-2-(2-(4-chlorophenyl)spiro[indole-3,3′-pyrrolidin]-2′-ylidene)acetate was prepared according to general procedure A starting from 2-(4-chlorophenyl)-3-(2-isocyanoethyl)-1*H*-indole (140.3 mg, 0.5 mmol, 1.0 equiv). The title compound was isolated as a light-yellow solid (138 mg, 0.38 mmol, 76%). *R*_*f*_ = 0.21 (EtOAc:*c*Hex = 1:4); ^1^H NMR (500 MHz, CDCl_3_): δ 8.28 (s, 1H), 7.92 (d, *J* = 8.6 Hz, 2H), 7.92 (d, *J* = 7.7 Hz, 1H), 7.46–7.37 (m, 3H), 7.30 (d, *J* = 7.1 Hz, 1H), 7.24 (t, *J* = 7.3 Hz, 1H), 4.05 (s, 1H), 4.06–3.93 (m, 4H), 2.62 (dt, *J* = 13.0, 8.9 Hz, 1H), 2.17 (dd, *J* = 12.9, 6.1 Hz, 2H)1.14 (t, *J* = 7.1 Hz, 1H) ppm; ^13^C{^1^H} NMR (126 MHz, CDCl_3_): δ 176.8 (C_q_), 170.9 (C_q_), 164.9 (C_q_), 153.8 (C_q_), 144.5 (C_q_), 137.4 (C_q_), 130.1 (C_q_), 130.0 (CH), 129.2 (CH), 129.0 (CH), 126.9 (CH) 121.4 (CH), 121.3 (CH), 77.8 (CH), 66.4 (C_q_), 59.0 (CH_2_), 46.2 (CH_2_), 33.1 (CH_2_), 14.5 (CH_3_) ppm. HRMS (ESI): *m*/*z* calculated for C_21_H_20_ClN_2_O_2_ [M+H^+^] = 367.1208, found = 367.1215.

#### Ethyl (*Z*)-2-(2-(Naphthalen-2-yl)spiro[indole-3,3′-pyrrolidin]-2′-ylidene)acetate (**23n**)

Ethyl (*Z*)-2-(2-(naphthalen-2-yl)spiro[indole-3,3′-pyrrolidin]-2′-ylidene)acetate was prepared according to general procedure A starting from 3-(2-isocyanoethyl)-2-(naphthalen-2-yl)-1*H*-indole (148.1 mg, 0.5 mmol, 1.0 equiv). The product was purified by flash column chromatography using EtOAc:*c*Hex = 1:4 as eluent to obtain the product as a white solid (115 mg, 0.30 mmol, 60%). *R*_*f*_ = 0.30 (1% Et_3_N in EtOAc:*c*Hex = 1:4); ^1^H NMR (500 MHz, CDCl_3_) δ (ppm): 8.44–8.30 (m, 2H), 8.18 (dd, *J* = 8.7, 1.8 Hz, 1H), 7.95–7.89 (m, 2H), 7.87 (d, *J* = 7.9, 1H), 7.75 (d, *J* = 7.8 Hz, 1H), 7.59–7.50 (m, 2H), 7.45 (td, *J* = 7.5, 1.3 Hz, 1H), 7.34 (dd, *J* = 7.5, 1.2 Hz, 1H), 7.26 (td, *J* = 7.4, 1.0 Hz, 1H), 4.12 (s, 1H), 4.11–3.94 (m, 4H), 2.78 (dt, *J* = 13.2, 9.0 Hz, 1H), 2.24 (ddd, *J* = 13.1, 6.7, 3.1 Hz, 1H), 1.12 (t, *J* = 7.2 Hz, 3H) ppm; ^13^C{^1^H} NMR (126 MHz, CDCl_3_): δ 177.9 (C_q_), 170.9 (C_q_), 165.4 (C_q_), 154.0 (C_q_), 144.7 (C_q_), 134.6 (C_q_), 133.0 (C_q_), 129.3 (CH), 129.3 (CH), 129.2 (C_q_), 128.9 (CH), 128.6 (CH), 127.8 (CH), 127.8 (CH), 126.8 (CH), 126.6 (CH), 125.3 (CH), 121.4 (CH), 121.3 (CH), 77.8 (CH), 66.6 (CH_q_), 58.9 (CH_2_), 46.3 (CH_2_), 33.4 (CH_2_), 14.5 (CH_3_) ppm. HRMS (ESI): *m*/*z* calculated for C_25_H_22_N_2_O_2_ [M+H^+^] = 383.1754, found = 383.1750.

#### Ethyl (*Z*)-2-(2-Bromospiro[indole-3,3′-pyrrolidin]-2′-ylidene)acetate (**23o**)

Ethyl (*Z*)-2-(2-bromospiro[indole-3,3′-pyrrolidin]-2′-ylidene)acetate was prepared according to general procedure A starting from 2-bromo-3-(2-isocyanoethyl)-1*H*-indole (129.9 mg, 0.52 mmol, 1.0 equiv). The title compound was isolated as a light-yellow solid (136 mg, 0.40 mmol, 78%). *R*_*f*_ = 0.43 (EtOAc:*c*Hex = 1:3); ^1^H NMR (500 MHz, CDCl_3_): δ 8.12 (s, 1H), 7.55 (d, *J* = 7.7 Hz, 1H), 7.35 (td, *J* = 7.3, 2.0 Hz, 1H), 7.30–7.22 (m, 2H), 4.10–3.94 (m, 5H), 2.53 (ddd, *J* = 13.1, 7.9, 5.0 Hz, 1H), 2.34 (ddd, *J* = 13.7, 8.1, 6.2 Hz, 1H), 1.17 (t, *J* = 7.2 Hz, 3H) ppm; ^13^C{^1^H} NMR (126 MHz, CDCl_3_): δ 170.6 (C_q_), 164.1 (C_q_), 161.9 (C_q_), 154.0 (C_q_), 141.8 (C_q_), 129.2 (CH), 127.2 (CH) 122.3 (CH), 120.7 (CH), 78.0 (CH), 71.0 (C_q_), 59.1 (CH_2_), 45.9 (CH_2_), 31.9 (CH_2_), 14.5 (CH_3_) ppm. HRMS (ESI): *m*/*z* calculated for C_15_H_16_BrN_2_O_2_ [M+H^+^] = 335.0390, found = 335.0388.

#### Ethyl (*Z*)-2-((*Z*)-2-(2-Methoxy-2-oxoethylidene)spiro[indoline-3,3′-pyrrolidin]-2′-ylidene)acetate (**23p**)

Ethyl (*Z*)-2-((*Z*)-2-(2-methoxy-2-oxoethylidene)spiro[indoline-3,3′-pyrrolidin]-2′-ylidene)acetate was prepared according to general procedure A starting from methyl 2-(3-(2-isocyanoethyl)-1*H*-indol-2-yl)acetate (121.6 mg, 0.5 mmol, 1.0 equiv). The title compound was isolated as a white solid (102 mg, 0.31 mmol, 62%). *R*_*f*_ = 0.28 (EtOAc:*c*Hex = 1:4); ^1^H NMR (500 MHz, CDCl_3_): δ 9.66 (s, 1H), 8.06 (s, 1H), 7.20 (t, *J* = 7.7 Hz, 1H), 7.12 (d, *J* = 7.4 Hz, 1H), 6.92 (t, *J* = 7.4 Hz, 1H), 6.85 (d, *J* = 7.8 Hz, 1H), 4.88 (s, 1H), 4.11 (s, 1H), 4.04 (qd, *J* = 7.1, 1.1 Hz, 2H), 3.82 (t, *J* = 6.8 Hz, 2H), 3.70 (s, 3H), 2.46–2.30 (m, 2H), 1.18 (t, *J* = 7.1 Hz, 3H) ppm; ^13^C{^1^H} NMR (126 MHz, CDCl_3_): δ 171.0 (C_q_), 170.5 (C_q_), 167.2 (C_q_), 167.0 (C_q_), 143.8 (C_q_), 132.6 (C_q_), 129.2 (CH), 123.4 (CH), 122.0 (CH), 109.4 (CH), 82.3 (CH), 78.8 (CH), 61.1 (C_q_), 59.0 (CH_2_), 50.9 (CH_3_), 45.4 (CH_2_), 38.5 (CH_2_), 14.6 (CH_3_) ppm. HRMS (ESI): *m*/*z* calculated for C_18_H_21_N_2_O_4_ [M+H^+^] = 329.1496, found = 329.1497.

#### Ethyl (*Z*)-2-(Spiro[indoline-3,3′-pyrrolidin]-2′-ylidene)acetate (**25a**)

Ethyl (*Z*)-2-(spiro[indoline-3,3′-pyrrolidin]-2′-ylidene)acetate was prepared according to general procedure B starting from 3-(2-isocyanoethyl)-1H-indole (85.2 mg, 0.5 mmol, 1.0 equiv), ethyl diazoacetate (0.6 mmol, 1.2 equiv), Bu_4_N[Fe(CO)_3_NO] (10.3 mg, 0.025 mmol, 0.05 equiv) and NaBH_4_ (20 mg, 0.53 mmol). The title compound was isolated as a yellow solid (99 mg, 0.38 mmol, 77%). *R*_*f*_ = 0.25 (EtOAc:*c*Hex 1:2); ^1^H NMR (500 MHz, CDCl_3_) δ (ppm): 7.96 (s, 1H), 7.08 (td, *J* = 7.6, 1.3 Hz, 1H), 7.01 (dd, *J* = 7.4, 1.3 Hz, 1H), 6.74 (td, *J* = 7.4, 1.0 Hz, 1H), 6.68 (d, *J* = 7.9, 1H), 4.44 (s, 1H), 4.07 (qd, *J* = 7.1, 3.1 Hz, 2H), 3.79 (br, 1H), 3.72–3.48 (m, 4H), 2.29–2.12 (m, 2H), 1.21 (t, *J* = 7.1 Hz, 3H) ppm; ^13^C{^1^H} NMR (126 MHz, CDCl_3_): δ 171.3 (C_q_), 170.7 (C_q_), 151.2 (C_q_), 132.5 (C_q_), 128.6 (CH), 123.7 (CH), 119.5 (CH), 110.1 (CH), 77.5 (CH), 59.4 (CH_2_), 58.7 (CH_2_), 57.3 (C_q_), 45.0 (CH_2_), 37.2 (CH_2_), 14.7 (CH_3_) ppm; HRMS (ESI): *m*/*z* calculated for C_15_H_19_N_2_O_2_ [M+H^+^] = 259.1441, 259.1441.

#### Ethyl (Z)-2-(2-methylspiro[indoline-3,3′-pyrrolidin]-2′-ylidene)acetate (**25b**)

To a flame-dried Schlenk flask under N_2_ atmosphere, charged with a stirring bean, was added Bu_4_N[Fe(CO)_3_NO] (10.3 mg, 0.025 mmol, 0.05 equiv). Subsequently, 1,2-DCE was added (0.25 M), and the mixture was stirred until the catalyst was dissolved. This was followed by the addition of 3-(2-isocyanoethyl)-2-methyl-1H-indole (92.4 mg, 0.5 mmol, 1.0 equiv), ethyl diazoacetate (0.6 mmol, 1.2 equiv). The solution was placed in a preheated oil bath and stirred at 80 °C until full conversion of the isocyanide was observed on TLC. Subsequently, the reaction mixture was cooled to 0 °C and diluted with MeOH to a concentration of 0.125 M, after which NaBH_3_CN (32 mg, 0.51 mmol, 1.02 equiv) and a few drops of AcOH were added. The resulting mixture was stirred at 0 °C until full conversion of the spiroindolenine intermediate was observed on TLC. Subsequently, the mixture was neutralized with Na_2_CO_3_ and diluted with CH_2_Cl_2_. The aqueous layer was extracted with CH_2_Cl_2_ (3 x). The combined organic layers were washed with brine, dried over Na_2_SO_4_, filtered, and concentrated *in vacuo*. This was followed by purification via FCC using a gradient of *c*Hex:EtOAc to obtain the title compound as a light-yellow solid (99 mg, 0.36 mmol, 73%). *R*_*f*_ = 0.29 (*c*Hex:EtOAc = 2:1); ^1^H NMR (500 MHz, CDCl_3_): δ 7.96 (s, 1H), 7.08 (td, *J* = 7.6, 1.3 Hz, 1H), 7.03 (dd, *J* = 7.4, 1.2 Hz, 1H), 6.76 (td, *J* = 7.4, 0.8 Hz, 1H), 6.66 (d, *J* = 7.7 Hz, 1H), 4.25 (s, 1H), 4.04 (q, *J* = 7.1 Hz, 2H), 3.86 (q, *J* = 6.5 Hz, 1H), 3.65–3.51 (m, 2H), 2.53–2.41 (m, 1H), 2.13 (ddd, *J* = 13.0, 6.6, 2.2 Hz, 1H).zf), 1.23 (d, *J* = 6.4 Hz, 3H), 1.20 (t, *J* = 7.1 Hz, 3H) ppm; ^13^C{^1^H} NMR (126 MHz, CDCl_3_): δ 171.1 (C_q_), 166.6 (C_q_), 151.0 (C_q_), 132.3 (C_q_), 128.6 (CH), 124.0 (CH), 119.6 (CH), 110.1 (CH), 79.5 (CH), 65.5 (CH), 60.0 (C_q_), 58.6 (CH_2_), 44.7 (CH_2_), 36.8 (CH_2_), 17.1 (CH_3_), 14.6 (CH_3_) ppm; HRMS (ESI): *m*/*z* calculated for C_16_H_21_N_2_O_2_ [M+H^+^] = 273.1598, found = 273.1603.

#### Ethyl (Z)-2-(5-methoxyspiro[indoline-3,3′-pyrrolidin]-2′-ylidene)acetate (**25q**)

Ethyl (Z)-2-(5-methoxyspiro[indoline-3,3′-pyrrolidin]-2′-ylidene)acetate was prepared according to general procedure B starting from 3-(2-isocyanoethyl)-5-methoxy-1H-indole (100.2 mg, 0.5 mmol, 1.0 equiv), ethyl diazoacetate (0.6 mmol, 1.2 equiv), Bu_4_N[Fe(CO)_3_NO] (10.3 mg, 0.025 mmol, 0.05 equiv) and NaBH_4_ (20 mg, 0.53 mmol). The title compound was isolated as a light-yellow solid (81 mg, 0.28 mmol, 56%). **Rf**: indoline = 0.16 (*c*Hex:EtOAc = 6:4); ^1^H NMR (500 MHz, CDCl_3_): δ 7.94 (s, 1H), 6.69–6.59 (m, 3H), 4.43 (s, 1H), 4.07 (qd, *J* = 7.1, 1.2 Hz, 2H), 3.72 (s, 3H), 3.68–3.48 (m, 4H), 3.26 (br, 1H), 2.29–2.11 (m, 2H), 1.21 (t, *J* = 7.1 Hz, 3H) ppm; ^13^C{^1^H} NMR (126 MHz, CDCl_3_): δ 171.3 (C_q_), 170.4 (C_q_), 154.3 (C_q_), 144.9 (C_q_), 134.2 (C_q_), 114.1 (CH), 111.2 (CH), 110.1 (CH), 77.7 (CH), 59.9 (CH_2_), 58.8 (CH_2_), 58.0 (C_q_), 56.0 (CH_3_), 45.0 (CH_2_), 36.9 (CH_2_), 14.7 (CH_3_) ppm; HRMS (ESI): *m*/*z* calculated for C_16_H_21_N_2_O_3_ [M+H^+^] = 289.1547, found = 289.1553.

#### Ethyl (Z)-2-(6-methoxyspiro[indoline-3,3′-pyrrolidin]-2′-ylidene)acetate (**25r**)

Ethyl (Z)-2-(6-methoxyspiro[indoline-3,3′-pyrrolidin]-2′-ylidene)acetate was prepared according to general procedure B starting from 3-(2-isocyanoethyl)-6-methoxy-1H-indole (100.1 mg, 0.5 mmol, 1.0 equiv), ethyl diazoacetate (0.6 mmol, 1.2 equiv), Bu_4_N[Fe(CO)_3_NO] (10.3 mg, 0.025 mmol, 0.05 equiv) and NaBH_4_ (20 mg, 0.53 mmol). Extra portions of NaBH_4_ were added over time until full conversion of the indolenine intermediate was observed. The title compound was isolated as a light-yellow solid (97 mg, 0.34 mmol, 67%). *R*_*f*_ = 0.24 (*c*Hex:EtOAc = 6:4); ^1^H NMR (500 MHz, CDCl_3_): δ 7.93 (s, 1H), 6.89 (d, *J* = 8.1 Hz, 1H), 6.29 (dd, *J* = 8.2, 2.3 Hz, 1H), 6.25 (d, *J* = 2.3 Hz, 1H), 4.43 (s, 1H), 4.07 (qd, *J* = 7.2, 2.2 Hz, 2H), 3.78 (s, 1H), 3.75 (s, 3H), 3.67–3.50 (m, 4H), 2.26–2.10 (m, 2H), 1.21 (t, *J* = 7.1 Hz, 3H) ppm; ^13^C{^1^H} NMR (126 MHz, CDCl_3_): δ 171.4 (C_q_), 171.0 (C_q_), 160.9 (C_q_), 152.6 (C_q_), 124.9 (C_q_), 124.2 (CH), 104.7 (CH), 96.5 (CH), 77.3 (CH), 59.9 (CH_2_), 58.8 (CH_2_), 56.7 (C_q_), 55.5 (CH_3_), 45.0 (CH_2_), 37.3 (CH_2_), 14.7 (CH_3_) ppm. HRMS (ESI): *m*/*z* calculated for C_16_H_21_N_2_O_3_ [M+H^+^] = 289.1547, found = 289.1554.

#### Ethyl (Z)-2-(5-methylspiro[indoline-3,3′-pyrrolidin]-2′-ylidene)acetate (**25s**)

Ethyl (Z)-2-(5-methylspiro[indoline-3,3′-pyrrolidin]-2′-ylidene)acetate was prepared according to general procedure B starting from 3-(2-isocyanoethyl)-5-methyl-1H-indole (92.2 mg, 0.5 mmol, 1.0 equiv), ethyl diazoacetate (0.6 mmol, 1.2 equiv), Bu_4_N[Fe(CO)_3_NO] (10.3 mg, 0.025 mmol, 0.05 equiv) and NaBH_4_ (20 mg, 0.53 mmol). Extra portions of NaBH_4_ were added over time until full conversion of the indolenine intermediate was observed. The title compound was isolated as a white solid (100 mg, 0.37 mmol, 74%). *R*_*f*_ = 0.64 (*c*Hex:EtOAc = 1:1); ^1^H NMR (500 MHz, CDCl_3_): δ 7.97 (s, 1H), 6.89 (d, *J* = 7.9 Hz, 1H), 6.83 (s, 1H), 6.61 (d, *J* = 7.9 Hz, 1H), 4.44 (s, 1H), 4.08 (qd, *J* = 7.1, 4.0 Hz, 2H), 3.72–3.50 (m, 4H), 3.47 (br, 1H), 2.27–2.12 (m, 5H), 1.22 (t, *J* = 7.1 Hz, 3H) ppm; ^13^C{^1^H} NMR (126 MHz, CDCl_3_): δ 171.4 (C_q_), 170.8 (C_q_), 148.7 (C_q_), 132.9 (C_q_), 129.2 (C_q_), 129.1 (CH), 124.3 (CH), 110.3 (CH), 77.5 (CH), 59.6 (CH_2_), 58.7 (CH_2_), 57.5 (C_q_), 45.0 (CH_2_), 37.1 (CH_2_), 21.0 (CH_3_), 14.7 (CH_3_) ppm; HRMS (ESI): *m*/*z* calculated for C_16_H_21_N_2_O_2_ [M+H^+^] = 273.1598, found = 273.1597.

#### Ethyl (Z)-2-(5-fluorospiro[indoline-3,3′-pyrrolidin]-2′-ylidene)acetate (**25t**)

Ethyl (Z)-2-(5-fluorospiro[indoline-3,3′-pyrrolidin]-2′-ylidene)acetate was prepared according to general procedure B starting from 5-fluoro-3-(2-isocyanoethyl)-1H-indole (94.4 mg, 0.5 mmol, 1.0 equiv), ethyl diazoacetate (0.6 mmol, 1.2 equiv), Bu_4_N[Fe(CO)_3_NO] (10.3 mg, 0.025 mmol, 0.05 equiv) and NaBH_4_ (20 mg, 0.53 mmol). The title compound was isolated as a light-brown solid (110 mg, 0.70 mmol, 79%). *R*_*f*_ = 0.25 (*c*Hex:EtOAc = 2:1); ^1^**H NMR** (500 MHz, CDCl_3_): δ 7.94 (s, 1H), 6.78 (td, *J* = 8.8, 2.7 Hz, 1H), 6.73 (dd, *J* = 8.3, 2.6 Hz, 1H), 6.60 (dd, *J* = 8.5, 4.3 Hz, 1H), 4.42 (s, 1H), 4.08 (q, *J* = 7.0 Hz, 2H), 3.68–3.52 (m, 4H), 3.31 (br, 1H), 2.31–2.10 (m, 2H), 1.22 (t, *J* = 7.2 Hz, 3H) ppm; ^13^C{^1^H} NMR (126 MHz, CDCl_3_): δ 171.3 (C_q_), 169.9 (C_q_), 157.5 (C_q_, d, *J* = 236.6 Hz), 147.1 (C_q_, d, *J* = 1.6 Hz), 134.2 (C_q_, d, *J* = 7.7 Hz), 115.0 (CH, d, *J* = 23.5 Hz), 111.1 (CH, d, *J* = 24.2 Hz), 110.7 (CH, d, *J* = 8.2 Hz), 77.8 (CH), 59.9 (CH_2_), 58.9 (CH_2_), 57.7 (C_q_), 45.0 (CH_2_), 37.0 (CH_2_), 14.7 (CH_3_) ppm; ^**19**^**F{**^1^**H} NMR** (470.4 MHz, CDCl_3_): δ −124.9 ppm; HRMS (ESI): *m*/*z* calculated for C_15_H_18_FN_2_O_2_ [M+H^+^] = 277.1347, found = 277.1346.

#### Ethyl (Z)-2-(5-chlorospiro[indoline-3,3′-pyrrolidin]-2′-ylidene)acetate (**25u**)

Ethyl (Z)-2-(5-chlorospiro[indoline-3,3′-pyrrolidin]-2′-ylidene)acetate was prepared according to general procedure B starting from 5-chloro-3-(2-isocyanoethyl)-1H-indole (102.8 mg, 0.5 mmol, 1.0 equiv), ethyl diazoacetate (0.6 mmol, 1.2 equiv), Bu_4_N[Fe(CO)_3_NO] (10.3 mg, 0.025 mmol, 0.05 equiv) and NaBH_4_ (20 mg, 0.53 mmol). The title compound was isolated as a white solid (96 mg, 0.33 mmol, 66%). *R*_*f*_ = 0.48 (*c*Hex:EtOAc = 1:1); ^1^H NMR (500 MHz, CDCl_3_): δ 7.94 (s, 1H), 7.02 (dd, *J* = 8.4, 2.1 Hz, 1H), 6.95 (d, *J* = 2.1 Hz, 1H), 6.57 (d, *J* = 8.3 Hz, 1H), 4.42 (s, 1H), 4.08 (q, *J* = 7.1 Hz, 2H), 3.79 (br, 1H), 3.69–3.50 (m, 4H), 2.28–2.09 (m, 2H), 1.22 (t, *J* = 7.1 Hz, 3H) ppm; ^13^C{^1^H} NMR (126 MHz, CDCl_3_): δ 171.2 (C_q_), 169.9 (C_q_), 149.8 (C_q_), 134.3 (C_q_), 128.5 (CH), 124.0 (CH), 123.9 (C_q_), 110.8 (CH), 77.8 (CH), 59.7 (CH_2_), 58.9 (CH_2_), 57.3 (C_q_), 45.0 (CH_2_), 37.2 (CH_2_), 14.7 (CH_3_) ppm. HRMS (ESI): *m*/*z* calculated for C_15_H_18_ClN_2_O_2_ [M+H^+^] = 293.1051, found = 293.1058.

#### Ethyl (Z)-2-(5-bromospiro[indoline-3,3′-pyrrolidin]-2′-ylidene)acetate (**25v**)

Ethyl (Z)-2-(5-bromospiro[indoline-3,3′-pyrrolidin]-2′-ylidene)acetate was prepared according to general procedure B starting from 5-bromo-3-(2-isocyanoethyl)-1*H*-indole (124.4 mg, 0.5 mmol, 1.0 equiv), ethyl diazoacetate (0.6 mmol, 1.2 equiv), Bu_4_N[Fe(CO)_3_NO] (10.3 mg, 0.025 mmol, 0.05 equiv), and NaBH_4_ (20 mg, 0.53 mmol). The title compound was isolated as a light-brown solid (102 mg, 0.30 mmol, 61%). *R*_*f*_ = 0.27 (*c*Hex:EtOAc = 2:1); ^1^H NMR (500 MHz, CDCl_3_): δ 7.94 (s, 1H), 7.15 (dd, *J* = 8.3, 2.0 Hz, 1H), 7.08 (d, *J* = 2.1 Hz, 1H), 6.54 (d, *J* = 8.3 Hz, 1H), 4.42 (s, 1H), 4.08 (q, *J* = 7.1 Hz, 2H), 3.80 (br, 1H), 3.68–3.50 (m, 4H), 2.24 (ddd, *J* = 12.8, 7.1, 4.1 Hz, 1H), 2.14 (dt, *J* = 12.7, 7.7 Hz, 1H), 1.23 (t, *J* = 7.1 Hz, 3H) ppm; ^13^C{^1^H} NMR (126 MHz, CDCl_3_): δ 171.2 (C_q_), 169.9 (C_q_), 150.2 (C_q_), 134.8 (C_q_), 133.4 (CH), 126.8 (CH), 111.4 (CH), 110.8 (C_q_), 77.8 (CH), 59.6 (CH_2_), 58.9 (CH_2_), 57.3 (C_q_), 45.0 (CH_2_), 37.2 (CH_2_), 14.7 (CH_3_) ppm; HRMS (ESI): *m*/*z* calculated for C_15_H_18_BrN_2_O_2_ [M+H^+^] = 337.0546, found = 337.0552.

#### Methyl (3*S*,*Z*)-2′-(2-Ethoxy-2-oxoethylidene)spiro[indoline-3,3′-pyrrolidine]-5′-carboxylate (**25w**)

To a flame-dried Schlenk flask under N_2_ atmosphere, charged with a stirring bean, was added Bu_4_N[Fe(CO)_3_NO] (10.3 mg, 0.025 mmol, 0.05 equiv). Subsequently, 1,2-DCE was added (0.25 M), and the mixture was stirred until the catalyst was dissolved. This was followed by the addition of 3-(1*H*-indol-3-yl)-2-isocyanopropanoate (114, mg, 0.50 mmol, 1.0 equiv) and ethyl diazoacetate (0.6 mmol, 1.2 equiv). The solution was placed in a preheated oil bath and stirred at 80 °C until full conversion of the isocyanide was observed on TLC. Subsequently, the reaction mixture was cooled to 0 °C and diluted with MeOH to a concentration of 0.125 M, after which NaBH_3_CN (33 mg, 0.53 mmol, 1.05 equiv) and a few drops of AcOH were added. The resulting mixture was stirred at 0 °C until full conversion of the spiroindolenine intermediate was observed on TLC. Subsequently, the mixture was neutralized with Na_2_CO_3_ and diluted with CH_2_Cl_2_. The aqueous layer was extracted with CH_2_Cl_2_ (3×). The combined organic layers were washed with brine, dried over Na_2_SO_4_, filtered, and concentrated *in vacuo*. This was followed by purification via FCC using *c*Hex:EtOAc = 6:4 as eluent to obtain the title compound as two diastereomers separately (*combined yield*: 86 mg, 0.25 mmol, 50%, dr = 2.2:1). dr determined via ^1^H NMR of the crude product mixture. **D1 (major):** yellow oil (62 mg, 0.18 mmol, 36%); *R*_*f*_ = 0.60 (*c*Hex:EtOAc = 6:4); ^1^H NMR (500 MHz, CDCl_3_): δ 8.23 (s, 1H), 7.09 (td, *J* = 7.7, 1.1 Hz, 1H), 6.99 (dd, *J* = 7.4, 1.0 Hz, 1H), 6.75 (td, *J* = 7.5, 0.9 Hz, 1H), 6.68 (d, *J* = 7.8 Hz, 1H), 4.52 (dd, *J* = 8.7, 3.8 Hz, 1H), 4.49 (s, 1H), 4.08 (qd, *J* = 7.1, 2.2 Hz, 2H), 3.78 (s, 3H), 3.71 (d, *J* = 9.6 Hz, 1H), 3.52 (d, *J* = 9.5 Hz, 1H), 2.58–2.41 (m, 2H), 1.21 (t, *J* = 7.1 Hz, 3H) ppm; ^13^C{^1^H} NMR (126 MHz, CDCl_3_): δ 172.7 (C_q_), 171.0 (C_q_), 169.6 (C_q_), 151.1 (C_q_), 132.3 (C_q_), 128.9 (CH), 123.5 (CH), 119.6 (CH), 110.3 (CH), 79.7 (CH), 60.5 (CH_2_), 59.0 (CH_2_), 58.7 (CH), 56.6 (C_q_), 52.7 (CH_3_), 40.6 (CH_2_), 14.6 (CH_3_) ppm. HRMS (ESI): *m*/*z* calculated for C_17_H_21_N_2_O_4_ [M+H^+^] = 317.1496, found = 317.1497. **D2 (minor):** yellow oil (24 mg, 0.07 mmol, 14%); *R*_*f*_ = 0.29 (*c*Hex:EtOAc = 6:4); ^1^H NMR (500 MHz, CDCl_3_): δ 8.17 (s, 1H), 7.09 (td, *J* = 7.7, 1.2 Hz, 1H), 7.03 (d, *J* = 7.5 Hz, 1H), 6.76 (td, *J* = 7.5, 0.8 Hz, 1H), 6.69 (d, *J* = 7.8 Hz, 1H), 4.48–4.41 (m, 2H), 4.09 (qd, *J* = 7.2, 2.3 Hz, 2H), 3.79 (s, 3H), 3.60 (dd, *J* = 15.9, 9.2 Hz, 2H), 2.62 (dd, *J* = 13.0, 7.0 Hz, 1H), 2.23 (dd, *J* = 13.0, 9.0 Hz, 1H), 1.22 (t, *J* = 7.1 Hz, 3H) ppm; ^13^C{^1^H} NMR (126 MHz, CDCl_3_): δ 171.8 (C_q_), 170.9 (C_q_), 169.0 (C_q_), 151.3 (C_q_), 131.2 (C_q_), 129.0 (CH), 124.2 (CH), 120.0 (CH), 110.3 (CH), 79.9 (CH), 60.3 (CH_2_), 59.0 (CH_2_), 58.5 (CH), 57.4 (C_q_), 52.7 (CH_3_), 40.7 (CH_2_), 14.6 (CH_3_) ppm. HRMS (ESI): *m*/*z* calculated for C_17_H_21_N_2_O_4_ [M+H^+^] = 317.1501, found = 317.1497.

#### Ethyl (*Z*)-2-((3*S*,4′*R*)-4′-(3-Methoxyphenyl)spiro[indoline-3,3′-pyrrolidin]-2′-ylidene) (**25x**)

To a flame-dried Schlenk flask under N_2_ atmosphere, charged with a stirring bean, was added Bu_4_N[Fe(CO)_3_NO] (10.3 mg, 0.025 mmol, 0.06 equiv). Subsequently, 1,2-DCE was added (0.25 M), and the mixture was stirred until the catalyst was dissolved. This was followed by the addition of 3-(2-isocyano-1-(3-methoxyphenyl)ethyl)-1*H*-indole (112 mg, 0.41 mmol, 1.0 equiv) and ethyl diazoacetate (0.6 mmol,1.5 equiv). The solution was placed in a preheated oil bath and stirred at 80 °C until full conversion of the isocyanide was observed on TLC. Subsequently, the reaction mixture was cooled to 0 °C and diluted with MeOH to a concentration of 0.125 M, after which NaBH_3_CN (33 mg, 0.53 mmol, 1.05 equiv) was added. After 30 min, NaBH_3_CN (31 mg, 0.50 mmol, 1.0 equiv) and a few drops of AcOH were added. The resulting mixture was stirred at 0 °C until full conversion of the spiroindolenine intermediate was observed on TLC. Subsequently, the mixture was neutralized with Na_2_CO_3_ and diluted with CH_2_Cl_2_. The aqueous layer was extracted with CH_2_Cl_2_ (3×). The combined organic layers were washed with brine, dried over Na_2_SO_4_, filtered, and concentrated *in vacuo*. This was followed by purification via FCC using *c*Hex:EtOAc = 2:1 as eluent to obtain both diastereomers separately (*combined yield*: 81 mg, 0.22 mmol, 54%, dr = 3:1). dr determined via ^1^H NMR of the crude product mixture. **D1 (major):** white solid (61 mg, 0.17 mmol, 41%); *R*_*f*_ = 0.44 (*c*Hex:EtOAc = 2:1); ^1^H NMR (500 MHz, CDCl_3_): δ 8.09 (s, 1H), 7.20–7.13 (m, 2H), 7.10 (td, *J* = 7.7, 1.2 Hz, 1H), 6.84–6.75 (m, 2H), 6.66 (d, *J* = 7.6 Hz, 1H), 6.60 (d, *J* = 7.8 Hz, 1H), 6.51 (t, *J* = 1.8 Hz, 1H), 4.53 (s, 1H), 4.09 (q, *J* = 6.9 Hz, 2H), 4.00 (dd, *J* = 10.0, 7.3 Hz, 1H), 3.83 (dd, *J* = 10.3, 6.7 Hz, 1H), 3.65 (s, 3H), 3.57 (t, *J* = 6.9 Hz, 1H), 3.46–3.32 (m, 2H), 1.23 (t, *J* = 7.1 Hz, 3H) ppm; ^13^C{^1^H} NMR (126 MHz, CDCl_3_): δ 171.4 (C_q_), 170.7 (C_q_), 159.6 (C_q_), 151.4 (C_q_), 140.4 (C_q_), 132.3 (C_q_), 129.6 (CH), 128.9 (CH), 123.7 (CH), 119.9 (CH), 119.6 (CH), 113.6 (CH), 112.9 (CH), 110.4 (CH), 78.1 (CH), 61.5 (C_q_), 58.8 (CH_2_), 55.1 (CH), 53.9 (CH_2_), 52.6 (CH_3_), 49.8 (CH_2_), 14.7 (CH_3_) ppm; HRMS (ESI): *m*/*z* calculated for C_22_H_25_N_2_O_3_ [M+H^+^] = 365.1860, found = 365.1868. **D2 (minor):** yellow oil (20 mg, 0.06 mmol, 13%); *R*_*f*_ = 0.26 (*c*Hex: EtOAc = 2:1); ^1^H NMR (500 MHz, CDCl_3_): δ 8.11 (s, 1H), 7.04 (t, *J* = 7.9 Hz, 1H), 6.95 (td, *J* = 7.7, 1.1 Hz, 1H), 6.67 (ddd, *J* = 8.2, 2.6, 0.9 Hz, 1H), 6.58 (d, *J* = 7.7 Hz, 2H), 6.43 (td, *J* = 7.5, 0.7 Hz, 1H), 6.36 (t, *J* = 1.9 Hz, 1H), 6.29 (d, *J* = 7.5 Hz, 1H), 4.49 (s, 1H), 4.18–4.04 (m, 2H), 4.00–3.88 (m, 2H), 3.72 (br, 1H), 3.66 (s, 2H), 3.58 (s, 3H), 3.49 (dd, *J* = 6.5, 3.7 Hz, 1H), 1.23 (t, *J* = 7.1 Hz, 3H) ppm; ^13^C{^1^H} NMR (126 MHz, CDCl_3_): δ 171.3 (C_q_), 169.2 (C_q_), 159.3 (C_q_), 151.8 (C_q_), 141.0 (C_q_), 129.1 (CH), 128.5 (CH), 128.2 (C_q_), 126.3 (CH), 120.5 (CH), 118.7 (CH), 113.7 (CH), 112.9 (CH), 109.9 (CH), 79.0 (CH), 63.0 (C_q_), 59.7 (CH_2_), 58.9 (CH_2_), 55.2 (CH), 51.6 (CH_3_), 50.9 (CH_2_), 14.7 (CH_3_) ppm; HRMS (ESI): *m*/*z* calculated for C_22_H_25_N_2_O_3_ [M+H^+^] = 365.1860, found = 365.1867.

#### Dimethyl (*R*,*Z*)-2-(Spiro[indole-3,3′-pyrrolidin]-2′-ylidene)succinate (**23ab**)

Dimethyl (*R*,*Z*)-2-(spiro[indole-3,3′-pyrrolidin]-2′-ylidene)succinate was prepared according to general procedure C starting from 3-(2-isocyanoethyl)-1*H*-indole (85.1 mg, 0.5 mmol, 1.0 equiv). The title compound was isolated as a white solid (50 mg, 0.16 mmol, 33%). *R*_*f*_ = 0.22 (*c*Hex:EtOAc = 1:1); ^1^H NMR (600 MHz, CDCl_3_): δ 8.90 (s, 1H), 8.12 (s, 1H), 7.67 (d, *J* = 7.7 Hz, 1H), 7.40 (td, *J* = 7.6, 1.3 Hz, 1H), 7.31 (d, *J* = 7.4 Hz, 1H), 7.27 (td, *J* = 7.4, 1.1 Hz, 1H), 3.84 (dddd, *J* = 10.1, 7.2, 6.1, 0.9 Hz, 1H), 3.77 (dddd, *J* = 10.1, 7.7, 5.8, 1.0 Hz, 1H), 3.60 (s, 3H), 3.43 (s, 3H), 2.47 (ddd, *J* = 12.8, 7.6, 6.1 Hz, 1H), 2.36–2.16 (m, 2H), 2.14 (ddd, *J* = 13.0, 7.5, 5.8 Hz, 1H) ppm; ^13^C{^1^H} NMR (150 MHz, CDCl_3_): δ 173.0 (C_q_), 172.7 (CH), 170.5 (C_q_), 160.3 (C_q_), 154.6 (C_q_), 140.3 (C_q_), 129.1 (CH), 127.3 (CH), 122.5 (CH), 122.1 (CH), 85.4 (C_q_), 67.2 (C_q_), 51.6 (CH_3_), 51.1 (CH_3_), 45.4 (CH_2_), 32.7 (CH_2_), 30.7 (CH_2_) ppm. HRMS (ESI): *m*/*z* calculated for C_17_H_19_N_2_O_4_ [M+H^+^] = 315.1339, found = 315.1338.

#### Dimethyl (*R*,*Z*)-2-(2-Methylspiro[indole-3,3′-pyrrolidin]-2′-ylidene)succinate (**23bb**)

Dimethyl (*R*,*Z*)-2-(2-methylspiro[indole-3,3′-pyrrolidin]-2′-ylidene)succinate was prepared according to general procedure C starting from 2-(methyl)-3-(2-isocyanoethyl)-1*H*-indole (92.1 mg, 0.5 mmol, 1.0 equiv). The title compound was isolated as a white solid (84 mg, 0.26 mmol, 51%). *R*_*f*_ = 0.68 (EtOAc); ^1^H NMR (600 MHz, CDCl_3_): 8.95 (s, 1H), 7.53 (d, *J* = 7.7 Hz, 1H), 7.34 (td, *J* = 7.6, 1.3 Hz, 1H), 7.24 (d, *J* = 7.4 Hz, 1H), 7.18 (td, *J* = 7.5, 1.0 Hz, 1H), 3.86–3.75 (m, 2H), 3.60 (s, 3H), 3.37 (s, 3H), 2.31 (s, 3H), 2.30–2.19 (m, 4H). δ ppm; ^13^C{^1^H} NMR (150 MHz, CDCl_3_): δ ^13^C NMR (150 MHz, CDCl_3_) δ 182.4 (C_q_), 172.6 (C_q_), 170.7 (C_q_), 161.9 (C_q_), 154.6 (C_q_), 142.1 (C_q_), 128.9 (CH), 126.3 (CH), 122.5 (CH), 120.8 (CH), 85.3 (C_q_), 67.9 (C_q_), 51.4 (CH_3_), 51.0 (CH_3_), 45.2 (CH_2_), 34.3 (CH_2_), 30.4 (CH_2_), 16.8 (CH_3_) ppm. HRMS (ESI): *m*/*z* calculated for C_18_H_21_N_2_O_4_ [M+H^+^] = 329.1496, found = 329.1495.

#### Benzyl (*R*,*Z*)-2-(2-Methylspiro[indole-3,3′-pyrrolidin]-2′-ylidene)-3-oxobutanoate (**23be**)

Benzyl (*R*,*Z*)-2-(2-methylspiro[indole-3,3′-pyrrolidin]-2′-ylidene)-3-oxobutanoate was prepared according to general procedure C starting from 2-(methyl)-3-(2-isocyanoethyl)-1*H*-indole (92.1 mg, 0.5 mmol, 1.0 equiv). The title compound was isolated as a white solid (17 mg, 0.05 mmol, 9%). *R*_*f*_ = 0.16 (*c*Hex:EtOAc = 3:7); ^1^H NMR (500 MHz, CDCl_3_): δ 12.02 (s, 1H), 7.53 (d, *J* = 7.7 Hz, 1H), 7.34 (td, *J* = 7.7, 7.2, 2.1 Hz, 1H), 7.25–7.16 (m, 5H), 7.08–7.01 (m, 2H), 4.45 (s, 2H), 3.97–3.80 (m, 2H), 2.37 (ddd, *J* = 13.1, 7.6, 5.9 Hz, 1H), 2.31 (s, 3H), 2.26–2.16 (m, 4H) ppm; ^13^C{^1^H} NMR (126 MHz, CDCl_3_): δ 196.7 (C_q_), 181.5 (C_q_), 168.6 (C_q_), 167.0 (C_q_), 155.0 (C_q_), 141.7 (C_q_), 136.3 (C_q_), 128.8 (CH), 128.4 (CH), 128.3 (CH), 127.9 (CH), 126.0 (CH), 121.2 (CH), 120.5 (CH), 100.7 (C_q_), 69.5 (C_q_), 64.8 (CH_2_), 45.6 (CH_2_), 35.6 (CH_2_), 29.5 (CH_3_), 17.0 (CH_3_) ppm. HRMS (ESI): *m*/*z* calculated for C_23_H_23_N_2_O_3_ [M+H^+^] = 375.1703, found = 375.1702.

#### Ethyl (*Z*)-2-(2-(2-Hydroxyethyl)spiro[indoline-3,3′-pyrrolidin]-2′-ylidene)acetate (**25y**)

2-(3-(2-Isocyanoethyl)-1*H*-indol-2-yl)ethan-1-ol (1.35 g, 6.33 mmol, 1.0 equiv) was added to a solution of Bu_4_[Fe(CO)_3_NO] (260 mg, 0.63 mmol, 0.10 equiv) in anhydrous 1,2-DCE (25 mL). Ethyl 2-diazoacetate (0.94 mL, 7.60 mmol, 1.2 equiv) was added, and the mixture was heated to 80 °C for 1.5 h and then allowed to cool to room temperature. The reaction was placed in an ice bath, and MeOH (10 mL) and NaBH_4_ (251 mg, 6.65 mmol, 1.05 equiv) were added. After complete conversion of the spiroindolenine was observed on TLC, the reaction was quenched with a saturated NH_4_Cl solution and extracted with CH_2_Cl_2_ (3×). The combined organic layers were dried over Na_2_SO_4_, filtered, and concentrated *in vacuo*. FCC (gradient: 20% → 80% EtOAc in cyclohexane) yielded the product as a light-brown solid as a single diastereomer (1.12 g, 3.70 mmol, 59%). *R*_*f*_ = 0.28 (EtOAc/cyclohexane 4:1); ^1^H NMR (500 MHz, CDCl_3_): δ 7.97 (s, 1H), 7.08 (t, *J* = 7.7 Hz, 1H), 7.03 (d, *J* = 7.4 Hz, 1H), 6.76 (t, *J* = 7.4 Hz, 1H), 6.67 (d, *J* = 7.8 Hz, 1H), 4.30 (s, 1H), 4.04 (q, *J* = 7.1 Hz, 2H), 3.94–3.76 (m, 3H), 3.69–3.52 (m, 2H), 2.49 (dt, *J* = 13.1, 8.8 Hz,, 1H), 2.17 (ddd, *J* = 13.1, 7.0, 2.8 Hz, 1H), 2.03–1.87 (m, 1H), 1.84–1.49 (m, 3H), 1.20 (t, *J* = 7.1 Hz, 3H) ppm; ^13^C{^1^H} NMR (126 MHz, CDCl_3_): δ 171.2 (C_q_), 167.0 (C_q_), 150.9 (C_q_), 132.3 (C_q_), 128.7 (CH), 123.8 (CH), 119.6 (CH), 110.3 (CH), 79.5 (CH), 68.6 (CH), 61.9 (CH_2_), 60.1 (C_q_), 58.7 (CH_2_), 44.9 (CH_2_), 37.0 (CH_2_), 33.8 (CH_2_), 14.7 (CH_3_) ppm; HRMS (ESI): *m*/*z* calculated for C_17_H_23_N_2_O_3_ [M+H^+^] = 303.1703, found = 303.1705.

#### Ethyl 2,3,5,6,6a,7-Hexahydro-1*H*-pyrrolo[2,3-*d*]carbazole-4-carboxylate (**26**)

To a mixture of imidazole (0.31 g, 4.6 mmol, 1.35 equiv), PPh_3_ (1.15 g, 4.4 mmol 1.30 equiv), and iodine (1.12 g, 4.4 mmol, 1.30 equiv) in CH_2_Cl_2_ (35 mL) was added ethyl (*Z*)-2-(2-(2-hydroxyethyl)spiro[indoline-3,3′-pyrrolidin]-2′-ylidene)acetate (1.02 g, 3.4 mmol, 1.0 equiv). After heating for an hour at reflux, the reaction mixture was allowed to cool to room temperature, after which MeOH (5 mL) was added causing the reaction mixture to turn to a clear solution. This solution was washed with a saturated Na_2_SO_3_ solution and subsequently extracted with CH_2_Cl_2_ (3×). The combined organic layer was dried over Na_2_SO_4_, filtered, and concentrated *in vacuo*. FCC (gradient: 5% → 40% EtOAc in cyclohexane) yielded the product as a light yellow solid and as a single diastereomer (858 mg, 3.0 mmol, 88%). *R*_*f*_ = 0.30 (EtOAc:*c*Hex = 1:4); ^1^H NMR (500 MHz, CDCl_3_): δ 7.53 (s, 1H), 7.03 (t, *J* = 7.8 Hz, 1H), 6.97 (d, *J* = 7.5 Hz, 1H), 6.65 (t, *J* = 7.5 Hz,1H), 6.61 (d, *J* = 7.8 Hz, 2H), 4.11 (q, *J* = 7.1 Hz, 2H), 3.94 (dd, *J* = 5.3, 2.9 Hz, 1H), 3.76 (td, *J* = 10.4, 6.1 Hz, 1H), 3.59 (ddd, *J* = 10.8, 9.2, 2.2 Hz, 1H), 2.42 (dt, *J* = 15.1, 4.5 Hz, 1H), 2.29 (dd, *J* = 12.0, 6.0 Hz, 1H), 2.14–2.05 (m,1H), 1.89 (ddd, *J* = 14.8, 10.9, 3.5 Hz, 1H), 1.75–1.59 (m, 2H), 1.24 (t, *J* = 7.1 Hz, 3H) ppm; ^13^C{^1^H} NMR (150 MHz, CDCl_3_): δ 169.4 (C_q_), 162.8 (C_q_), 150.2 (C_q_), 132.8 (C_q_), 128.6 (CH), 123.2 (CH), 118.8 (CH), 109.0 (CH), 89.6 (C_q_), 63.7 (CH), 59.0 (CH_2_), 55.4 (C_q_), 44.2 (CH_2_), 39.4 (CH_2_), 33.5 (CH_2_), 18.5 (CH_2_), 14.8 (CH_3_) ppm; HRMS (ESI): *m*/*z* calculated for C_17_H_21_N_2_O_2_ [M+H^+^] = 285.1597, found = 285.1598.

#### 7-(*tert*-Butyl) 4-Ethyl 1,2,3,5,6,6a-Hexahydro-7*H*-pyrrolo[2,3-*d*]carbazole-4,7-dicarboxylate (**20**)^3g^

Ethyl 2,3,5,6,6a,7-hexahydro-1*H*-pyrrolo[2,3-*d*]carbazole-4-carboxylate (142 mg, 0.5 mmol, 1.0 equiv) was dissolved in anhydrous CH_2_Cl_2_ (0.5 M), followed by the addition of DMAP (12 mg, 0.1 mmol, 0.2 equiv) and Boc_2_O (372 mg, 1.5 mmol, 3.0 equiv). No full conversion was observed on TLC after 24 h, and an additional portion of Boc_2_O (164 mg, 0.75 mmol, 1.5 equiv) was added. After 48 h no full conversion was observed and additional amounts of Boc_2_O (372 mg, 1.5 mmol, 1.5 equiv) and DMAP (12 mg, 0.1 mmol, 0.2 equiv) were added. Additional portions of Boc_2_O (372 mg, 1.5 mmol, 1.5 equiv) and DMAP (12 mg, 0.1 mmol, 0.2 equiv) were added after 72 h and stirred until full conversion was observed. After completion of the reaction, the reaction was diluted with CH_2_Cl_2_, washed with H_2_O and brine, and dried over Na_2_SO_4_, followed by filtration and concentration *in vacuo*. The crude reaction mixture was then purified by FCC using EtOAc:*c*Hex = 1:9 as eluent to obtain the product as a white foam (136 mg, 0.35 mmol, 71%). Characterization data is accordance with that reported in the literature.^3g^*R*_*f*_ = 0.26 EtOAc:*c*Hex = 1:9); ^1^H NMR (600 MHz, CDCl_3_): δ 7.97–7.34 (m, 1H), 7.18 (s, 1H), 7.01 (d, *J* = 7.5 Hz, 1H), 6.90 (td, *J* = 7.5, 1.1 Hz, 1H), 4.46 (m, 1H), 4.11 (qd, *J* = 7.1, 1.3 Hz, 1H), 3.74 (td, *J* = 10.3, 6.3 Hz, 1H), 3.61 (t, *J* = 9.5 Hz, 1H), 2.48–2.38 (m, 1H), 2.26 (dd, *J* = 12.1, 6.1 Hz, 1H), 2.22–2.05 (m, 2H), 1.73–1.52 (m, 12H), 1.24 (t, *J* = 7.1 Hz, 3H) ppm; ^13^C{^1^H} NMR (150 MHz, CDCl_3_): (presence of rotameric signals) δ 169.3 (C_q_), 162.2 (C_q_), 152.0 (C_q_), 142.3 (C_q_), 134.4 (C_q_), 128.7 (CH), 123.0 (CH), 122.7 (CH), 114.7 (CH), 89.9 (C_q_), 81.1 (C_q_), 66.2 (CH), 59.1 (CH_2_), 53.8 (C_q_), 44.0 (CH_2_), 39.3 (CH_2_), 31.4 (CH_2_), 28.6 (CH_3_), 18.4 (CH_2_), 14.8 (CH_3_) ppm. HRMS (ESI): *m*/*z* calculated for C_22_H_29_N_2_O_4_ [M+H^+^] = 385.2122, found = 385.2127.

## Data Availability

The data underlying this study are available in the published article and its Supporting Information.

## Supporting Information Available

Experimental procedures, characterization data, and ^1^H and ^13^C NMR spectra for new compounds (PDF)
FAIR data, including the primary NMR FID files, for compounds **1g**, **1h**, **1i**, **1k**–**1n**, **1p**, **1x**, **1y**, **20**, **23a**, **23ab**, **23b**, **23ab**, **23bb**, **23be**, **23c**–**23g**, **23i**, **23j**–**23o**, **25a**, **25b**, **25q**, **25r**–**25v, 25w_D1**, **25w_D2**, **25x_D1**, **25x_D2**, **25y**, and **26** (ZIP)
