# Supplementary material for: Dearomative Spirocyclization of Tryptamine-Derived Isocyanides via Iron-Catalyzed Carbene Transfer
Source: J Org Chem. 2023 Dec 4;88(24):17345–55. doi: 10.1021/acs.joc.3c02160 (PMC10729054; doi:10.1021/acs.joc.3c02160)

Supporting Information

Dearomative Spirocyclization of Tryptamine-Derived Isocyanides via Iron-Catalyzed Carbene Transfer

Thomas R. Roose,[a] Finn McSorley[a], Bryan Groenhuijzen [a], Jordy. M. Saya,[c] Bert U. W. Maes* [b], Eelco Ruijter* [a] and Romano V. A. Orrù* [a, c]

[a] Department of Chemistry and Pharmaceutical Sciences and Amsterdam Institute for Molecules, Medicines & Systems (AIMMS), Vrije Universiteit Amsterdam, De Boelelaan 1108, 1081 HZ Amsterdam, The Netherlands. E-mails: e.ruijter@vu.nl; t.r.roose@vu.nl

Institute for Molecules Medicines and Systems (AIMMS), Vrije Universiteit
Amsterdam,
De Boelelaan 1108, 1081 HZ Amsterdam, The Netherlands
E-mail: e.ruijter@vu.nl; r.v.a.orru@vu.nl
http://syborch.com/
[b] Organic Synthesis, Department of Chemistry, University of Antwerp, Groenenborgerlaan 171, 2020 Antwerp, Belgium. E-mail : bert.maes@uantwerpen.be

[c] Organic Chemistry, Aachen-Maastricht Institute for Biobased Materials (AMIBM), Maastricht University, Urmonderbaan 22, 6167 KD Geleen, Netherlands. E-mail : r.orru@maastrichtuniversity.nl

DOI:

Table of Contents

1. General information ... 2

2. Optimization data: $Bu_4N[Fe(CO)_3NO]$-catalyzed carbene transfer to tryptamine-derived isocyanide ... 3

3. Optimization data: One-pot $Bu_4N[Fe(CO)_3NO]$-catalyzed carbene transfer to tryptamine-derived isocyanide and reduction to spiroindolines ... 4

4. Synthesis of starting materials ... 5

4.1 Synthesis of $Bu_4N[Fe(CO)_3NO]$ ... 5

4.2 Synthesis of Diazo compounds ... 5

4.3 Synthesis of isocyanides ... 6

5. Scope tryptamine derived isocyanides ... 12

6 Diazo scope ... 19

7 Towards total synthesis ... 21

8 References ... 23

9 ^{1}H-, ^{13}C-, ^{19}F-NMR spectra of compounds ... 24

1. General information

Unless stated otherwise, all solvents and commercially available reagents were used as purchased. Anhydrous dichloromethane, THF, DMF and toluene were obtained via the PureSolv MD 5 Solvent Purification System. All other solvents were used as purchased from the corresponding supplier. Used diazo compounds in this work were either obtained commercially or synthesized according to the corresponding literature procedures. ***Caution!*** It should be noted that diazo compounds can be potentially explosive. Correct safety measures, such as the scale of the reaction, and careful handling are required. Use of appropriate safety gear, including a blast shield, is strongly recommended. Nuclear magnetic resonance (NMR) spectra were recorded on a Bruker Avance 600 MHz (150 MHz for ^{13}C), Bruker Avance 500 MHz (126 MHz for ^{13}C) & (470 MHz for ^{19}F) or Bruker Avance 300 MHz (75.4 MHz for ^{13}C) using the residual solvent as internal standard (^{1}H: δ 7.26 ppm, ^{13}C {^{1}H}: δ 77.16 ppm for $CDCl_3$, ^{1}H: δ 2.50 ppm, ^{13}C{^{1}H}: δ 39.52 ppm for DMSO-*d*6). Chemical shifts (δ) are given in ppm and coupling constants (J) are quoted in hertz (Hz). Resonances are described as s (singlet), d (doublet), t (triplet), q (quartet), quint (quintet), sex (sextet), sep (septet), br (broad singlet) and m (multiplet) or combinations thereof. Electrospray Ionization (ESI) high-resolution mass spectrometry was carried out using a Bruker QTOF impact II instrument in positive ion mode (capillary potential of 4500 V). Flash chromatography was performed on Silicycle Silia-P Flash Silica Gel (particle size 40-63 µm, pore diameter 60Å) using the indicated eluent. Thin Layer Chromatography (TLC) was performed using TLC plates from Merck (SiO_2, Kieselgel 60 F254 neutral, on aluminium with fluorescence indicator) and compounds were visualized by UV detection (254 nm) and $KMnO_4$ stain. SFC-MS analysis was conducted using a Shimadzu Nexera SFC-MS equipped with a Nexera X2 SIL-30AC autosampler, Nexera UC LC-30AD SF CO_2 pump, Nexera X2 LC-30AD liquid chromatograph, Nexera UC SFC-30A back pressure regulator, prominence SPD-M20A diode array detector, prominence CTO-20AC column oven and CBM-20A system controller. A gradient of supercritical CO_2 (A) and methanol (B) was used. Method: 2% B/98% A ◊ 100% B/0% A over the course of 7 min. The flow was maintained at 2.0 mL/min and the sample injection volume was 5 µL. Mass spectrometry analyses were performed using a Shimadzu LCMS-2020 mass spectrometer. The data were acquired in full-scan APCI mode (MS) from m/z 100 to 800 in positive ionisation mode. Data was processed using Shimadzu Labsolutions 5.82.

2. **Optimization data:** $Bu_4N[Fe(CO)_3NO]$-catalyzed carbene transfer to tryptamine-derived isocyanide

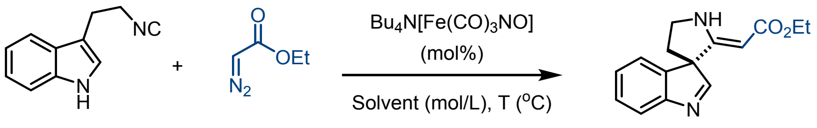

1a **22** **23a**

To a flame dried Schlenk flask under N_2 atmosphere, charged with a stirring bean, was added $Bu_4N[Fe(CO)_3NO]$. Subsequently, the solvent was added, and the mixture was stirred until the catalyst was dissolved. This was followed by the addition of isocyanide **1a** (0.5 mmol) and ethyl diazoacetate (**2a**). The solution was placed in a pre-heated oil bath and stirred at the given temperature until full conversion of **1a** was observed on TLC. If no full conversion of substrate **1a** was observed after 22-24h, the reaction was stopped, nonetheless. Subsequently, the reaction mixture was filtered through a pad of silica using 5% Et_3N solution in EtOAc:*c*Hex (1:1) as eluent. The filtrate was collected and concentrated *in vacuo*. All reactions were performed on a 0.5 mmol scale and yields were determined via ^{1}H-NMR, using 2,5-dimethylfuran as internal standard.

Table S1. Optimization data $Bu_4N[Fe(CO)_3NO]$-catalyzed carbene transfer to tryptamine-derived isocyanide (**23a**)

Entry	Catalyst (mol %)	Additive (mol%)	Equivalents 22	Solvent (mol/L)	T (°C)	Yield 23a (%)[a]
**1**	$Bu_4N[Fe(CO)_3NO]$ (5)	-	1.0	1,2-DCE (0.25)	80	83
**2**	$Bu_4N[Fe(CO)_3NO]$ (5)	PPh_3 (5.5)	1.2	1,2-DCE (0.25)	80	89
**3**	$Bu_4N[Fe(CO)_3NO]$ (5)	P(2-Fur)$_3$ (5.5)	1.2	1,2-DCE (0.25)	80	86
**4**	$Bu_4N[Fe(CO)_3NO]$ (5)	-	1.2	1,2-DCE (0.25)	80	98 (96)[b], [c]
**5**	$Bu_4N[Fe(CO)_3NO]$ (5)	-	1.2	1,2-DCE (0.25)	60	22[d]
**6**	$Bu_4N[Fe(CO)_3NO]$ (5)	-	1.2	1,2-DCE (0.125)	80	82
**7**	$Bu_4N[Fe(CO)_3NO]$ (10)	-	1.2	1,2-DCE (0.25)	80	87
**8**	$Bu_4N[Fe(CO)_3NO]$ (2.5)	-	1.2	1,2-DCE (0.25)	80	81
**9**	$Bu_4N[Fe(CO)_3NO]$ (5)	*p*-nitroanisole (10)	1.2	1,2-DCE (0.25)	80	63
**10**	$Bu_4N[Fe(CO)_3NO]$ (5)	-	1.2	1,4-dioxane (0.25)	80	70
**11**	$Bu_4N[Fe(CO)_3NO]$ (5)	-	1.2	CH_3CN (0.25)	80	89
**12**	$Bu_4N[Fe(CO)_3NO]$ (5)	-	1.2	PhMe (0.25)	80	66
**13**	$Bu_4N[Fe(CO)_3NO]$ (5)	-	1.2	DMF (0.25)	80	85
**14**	$Bu_4N[Fe(CO)_3NO]$ (5)	-	1.2	*i*-PrOH (0.25)	80	56
**15**	-	-	1.2	1,2-DCE (0.25)	80	n.p.[d]
**16**	$Fe(CO)_5$ (5)	-	1.2	1,2-DCE (0.25)	80	92
**17**	Fe(Pc) (5)	-	1.2	1,2-DCE (0.25)	80	21[e]
**18**	Fe(TPP)Cl (5)	-	1.2	1,2-DCE (0.25)	80	18[e]
**19**	Fe(TPP)Cl (5)	Zn (50)	1.2	1,2-DCE (0.25)	80	trace[e]
**20**	$FeCp(CO)_2I$ (5)	-	1.2	1,2-DCE (0.25)	80	n.p.[d]
**21**	$FeCp(CO)_2I$ (5)	$AgBF_4$ (6)	1.2	1,2-DCE (0.25)	80	n.p.[d]
**22**	$Fe(ClO_4)_2.4H_2O$ (5)	-	1.2	1,2-DCE (0.25)	80	n.p.[d]
**23**	$Fe(ClO_4)_2.4H_2O$ (5)	TMEDA (6) $NaBar_F$ (6)	1.2	1,2-DCE (0.25)	80	trace[d]
**24**	$Fe(ClO_4)_2.4H_2O$ (5)	DPPE (6) $NaBar_F$ (6)	1.2	1,2-DCE (0.25)	80	trace[d]

[a] Reactions performed on a 0.5 mmol scale. Yields are determined via ^{1}H-NMR, using 2,5-dimethylfuran as internal standard; [b] Isolated yield. [c] Full conversion of isocyanide **1a**, based on TLC, after 75 min. [d] No full conversion observed of isocyanide **1a** on TLC after 22-24 h stirring at 80 °C. [e] Full conversion of ethyl diazoacetate (**22**) prior to full conversion of isocyanide **1a**.

3. **Optimization data:** One-pot $Bu_4N[Fe(CO)_3NO]$-catalyzed carbene transfer to tryptamine-derived isocyanide and reduction to spiroindolines

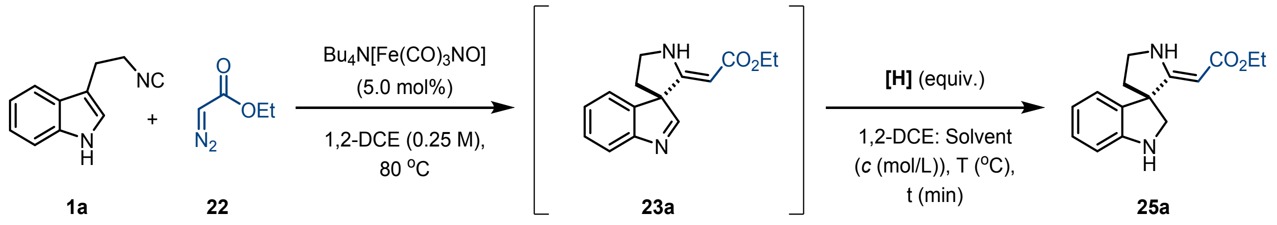

To a flame dried Schlenk flask under N_2 atmosphere, charged with a stirring bean, was added $Bu_4N[Fe(CO)_3NO]$ (10.3 mg, 0.025 mmol, 0.05 equiv.). Subsequently, 1,2-DCE (2 mL) was added, and the mixture was stirred until the catalyst was dissolved. This was followed by the addition of isocyanide **1a** (85.1, mg, 0.5 mmol, 1.0 equiv.) and ethyl diazoacetate (**22**) (0.6 mmol, 1.2 equiv.). The solution was placed in a pre-heated oil bath and stirred at 80 °C until full conversion of **1a** was observed on TLC. Subsequently, the reaction mixture was cooled to the given temperature and diluted with the indicated protic solvent. Then, reducing agent **[H]** was added and the reaction was stirred at the given temperature until full conversion of indolenine **23a** was observed on TLC. Afterwards, the reaction mixture was quenched with saturated aqueous NH_4Cl solution and stirred vigorously for 15 minutes. The aqueous layer was extracted with CH_2Cl_2 (3x). The organic layers were collected, washed with brine, dried over Na_2SO_4, and filtered. The filtrate was collected and concentrated *in vacuo*. All reactions were performed on a 0.5 mmol scale and yields were determined via ^1H-NMR, using 1,3,5-trimeyhoxybenzene as internal standard.

Table S2. Optimisation data for $Bu_4N[Fe(CO)_3NO]$-catalysed carbene transfer to tryptamine-derived isocyanide (**1a**) followed by one-pot reduction.

Entry	[H] (equiv.)	Solvent (mol/L)	T (°C)	Yield 25a (%)[a]
**1**	$NaBH_4$ (5.0)	MeOH:TFE (0.125)[b]	rt	29[c]
**2**	$NaBH_4$ (5.0)	MeOH (0.125)	rt	56[c]
**3**	$NaBH_4$ (1.05)	MeOH (0.125)	rt	75
**4**	$NaBH_4$ (1.05)	MeOH (0.125)	0	83 (77)[f]
**5**	$NaBH_4$ (1.05)	EtOH (0.125)	0	76
**6**	$NH_3 \cdot BH_3$ (1.05)	MeOH (0.125)	0->rt	75[d]
**7**	$NH_3 \cdot BH_3$ (1.20)	MeOH (0.125)	0->rt	67
**8**	$NaBH_3CN$ (1.05)	MeOH (0.125)	0->rt	28[d]
**9**	$NaBH_3CN$ (1.05)[e]	MeOH (0.125)	0->rt	75 (72)[f]

[a] Yield determined via ^1H-NMR analysis using 1,3,5-trimethoxybenzene as internal standard. [b] MeOH:TFE = (4:1) as solvent system. [c] Partial reduction of enaminone observed on crude ^1H-NMR. [d] No full conversion of spiroindolenine intermediate based on TLC after prolonged stirring. [e] Addition of few drops of AcOH to the reaction mixture prior to addition of [H]. [f] Isolated yield.

4. Synthesis of starting materials

4.1 Synthesis of $Bu_4N[Fe(CO)_3NO]$

Tetra-*N*-butylammonium Tricarbonylnitosoferrate ($Bu_4N[Fe(CO)_3NO]$)

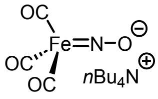

TBA[Fe] was synthesized according to well-established literature procedures[1] starting from $Fe(CO)_5$ (15.0 mmol, 1.0 equiv., 1.97 mL), $NaNO_2$ (15.0 mmol, 1.0 equiv., 1.035 g) and BrN(*n*-Bu)$_4$ (15.0 mmol, 1.0 equiv., 4.863 g) to give TBA[Fe] as a yellow solid (5.196 g, 84%). **IR (Neat):** *v*max (cm^{-1}) = 1975 (w), 1846 (s), 1636 (m). **IR (1,2-DCE, film):** *v*max (cm^{-1}) = 1988 (w), 1872 (s), 1639 (m).

4.2 Synthesis of Diazo compounds

Used diazo compounds in this work were either obtained commercially or synthesized according to the corresponding literature procedures.

Caution! *It should be noted that diazo compounds can be potentially explosive. Correct safety measures, such as the scale of the reaction, and careful handling are required. Use of appropriate safety gear, including a blast shield, is strongly recommended.*

methyl 2-diazo-2-phenylacetate (18a)

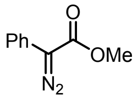

Methyl 2-diazo-2-phenylacetate **18a** was synthesized according to a procedure found in literature, starting methyl 3-oxo-2-phenylbutanoate (422.6 µL, 3.0 mmol).[4] The title compound was isolated as a slight orange solid (230 mg, 1.31 mmol, 44%). The characterization data matched the data reported in literature.[5] **^{1}H NMR** (500 MHz, $CDCl_3$): δ 7.48 (dd, *J* = 8.6, 1.2 Hz, 2H), 7.39 (dd, *J* = 8.4, 7.4 Hz, 2H), 7.19 (tt, *J* = 7.6, 1.2 Hz, 1H), 3.87 (s, 3H) ppm.

dimethyl 2-diazosuccinate (18b)

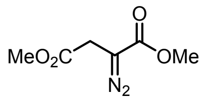

Dimethyl 2-diazosuccinate **18b** was synthesized according to a procedure found in literature, starting from dimethyl acetylsuccinate (1.46 mL, 9.0 mmol).[4] The title compound was isolated as a yellow oil (1.27 g, 7.3 mmol, 83%). The characterization data matched the data reported in literature.[4] **^{1}H NMR** (500 MHz, $CDCl_3$): δ 3.77 (s, 3H), 3.74 (s, 3H), 3.32 (s, 2H) ppm.

dimethyl 2-diazopentanedioate (18c)

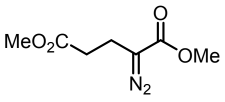

Dimethyl 2-diazopentanedioate **18c** was synthesized according to a procedure found in literature, starting from L-Glutamic acid dimethylester hydrochloride (1.06 g, 5.0 mmol).[2] The title compound was isolated as a yellow oil (471 mg, 2.53 mmol, 51%). The characterization data matched the data reported in literature.[3] **^{1}H NMR** (500 MHz, $CDCl_3$): δ 3.74 (s, 3H), 3.68 (s, 3H), 2.73 – 2.49 (m, 4H) ppm.

5-ethyl 1-methyl (E)-4-diazopent-2-enedioate (18d)

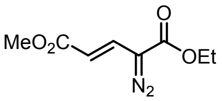

α-Diazo ester **18d** was synthesized according to a procedure found in literature, starting from methyl (E)-3-iodoacrylate (4.15 g, 19.6 mmol), 1.0 equiv.).[6] The title compound was isolated as a yellow solid (2.20 g, 11.1 mmol, 57%). The characterization data matched the data reported in literature.[6] **^{1}H NMR** (300 MHz, $CDCl_3$): δ 7.31 (d, *J* = 15.7 Hz, 1H), 5.69 (d, *J* = 15.4 Hz, 1H), 4.28 (q, *J* = 7.1 Hz, 2H), 3.72 (s, 3H), 1.29 (t, *J* = 7.1 Hz, 3H) ppm.

benzyl 2-diazo-3-oxobutanoate (18e)

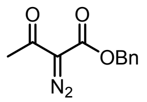

Benzyl 2-diazo-3-oxobutanoate **18e** was in stock and can be synthesized via a well-established procedure.[4] The characterization data matched the data reported in literature.[4] **^{1}H NMR** (600 MHz, $CDCl_3$): δ 7.42 – 7.32 (m, 5H), 5.27 (s, 2H), 2.49 (s, 3H) ppm.

4.3 Synthesis of isocyanides

Tryptamine derived isocyanides used in this work were either in stock on the lab or synthesized from their formamide precursor via conventional dehydration methods.[7] The corresponding formamides were synthesized either via formylation of the commercially available tryptamine derivative[7] (general procedure A) or from the commercially available indole via a reductive coupling with *N*-(2,2-dimethoxyethyl)formamide[8] (General procedure B), and were used in the dehydration step towards the isocyanide. Quality of tryptamine derived isocyanides in stock was determined via ^{1}H NMR prior to their transformation to the spiroindolenine/spiroindoline. If required purification was performed via flash column chromatography using a mixture of EtOAc:*c*Hex as eluent to obtain the pure isocyanide.

General procedure A:

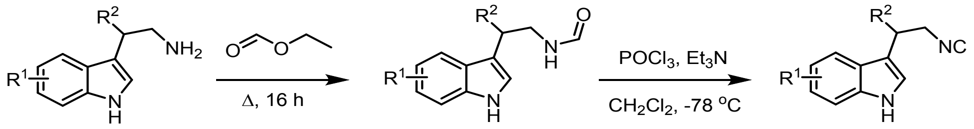

Tryptamine derivative (1 equiv.) was added to ethyl formate and heated under reflux for 16 h. Subsequently, the volatiles were removed *in vacuo*. The crude formamide was directly used in the dehydration step without any further purification. Et_3N was added prior to the start of the reaction if the tryptamine derivative was purchased as HCl salt. If required, the crude product was subjected to flash column chromatography to obtain the title compound.

Next, to a flame-dried Schlenk tube under N_2-atmosphere, tryptamine derived formamide (1.0 equiv.) was added and dissolved in anhydrous CH_2Cl_2 (0.5 M), after which Et_3N (5.0 equiv.) was added. Subsequently, the solution was cooled to -78 °C and $POCl_3$ (1.5 equiv.) was added dropwise. After completion, the reaction was quenched with H_2O and vigorously stirred for 30 minutes. The product was extracted with CH_2Cl_2 (3x), and the combined organic layers were washed with water and brine, dried over Na_2SO_4, filtered, and concentrated *in vacuo*. The crude mixture was purified by FCC to obtain the title compound.

General procedure B:

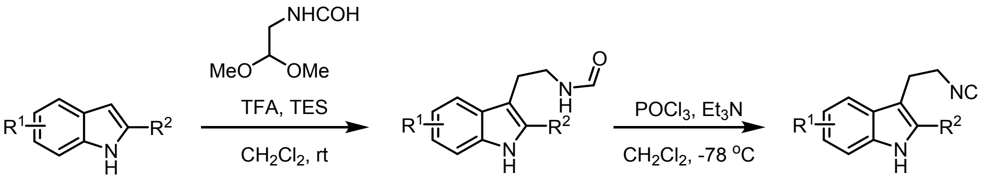

Reactions were performed in flame-dried glassware under a N_2-atmospere. A solution of *N*-(2,2-dimethoxyethyl)formamide (1.1 equiv.) and indole derivative (1.0 equiv.) in anhydrous CH_2Cl_2 (0.5 M) was added dropwise to a solution of triethylsilane (TES) (3.0 equiv.) and trifluoroacetic acid (TFA) (5.0 equiv.) in CH_2Cl_2 (0.5 M) at room temperature. The reaction mixture was stirred for 16 hours. Subsequently, the reaction mixture was basified at 0 °C, using saturated $NaHCO_3$ solution. The aqueous layer was extracted with CH_2Cl_2 (3x). The collected organic layers were washed with brine, dried over Na_2SO_4, filtered, and concentrated *in vacuo*. The crude product was then subjected to flash column chromatography to obtain the title compound, which was directly used in the next dehydration step.

Next, to a flame-dried Schlenk tube under N_2-atmosphere, tryptamine derived formamide (1.0 equiv.) was added and dissolved in anhydrous CH_2Cl_2 (0.5 M), after which Et_3N (5.0 equiv.) was added. Subsequently, the solution was cooled to -78 °C and $POCl_3$ (1.5 equiv.) was added dropwise. After completion, the reaction was quenched with H_2O and vigorously stirred for 30 minutes. The product was extracted with CH_2Cl_2 (3x), and the combined organic layers were washed with water and brine, dried over Na_2SO_4, filtered, and concentrated *in vacuo*. The crude mixture was purified by FCC, using CH_2Cl_2 or a mixture of EtOAc:*c*Hex as eluent, to obtain the title compound.

3-(2-isocyanoethyl)-1H-indole (1a)

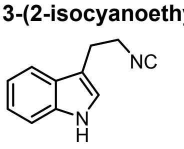

3-(2-isocyanoethyl)-1H-indole was in stock and can be synthesized via well-established a procedure (General procedure A).[7] The characterization data matched the data reported in literature.[7] **R_f** = 0.88 (CH_2Cl_2); **^{1}H NMR** (500 MHz, $CDCl_3$): δ 8.19 (s, 1H), 7.60 (d, J = 7.7 Hz, 1H), 7.40 (d, J = 8.1 Hz, 1H), 7.28 (td, J = 9.0, 8.1, 2.8 Hz, 1H), 7.21 (td, J = 7.5, 2.8 Hz, 1H), 7.10 (t, J = 2.1 Hz, 1H), 3.67 (tt, J = 7.0, 1.9 Hz, 2H), 3.18 (tt, J = 7.0, 2.0 Hz, 2H) ppm.

3-(2-isocyanoethyl)-2-methyl-1H-indole (1b)

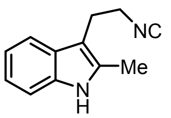

3-(2-isocyanoethyl)-2-methyl-1H-indole was in stock and can be synthesized via well-established procedure (General procedure A).[7] The ^{1}H-NMR data matched the data reported in literature.[7] **^{1}H NMR** (500 MHz, $CDCl_3$): δ 7.89 (s, 1H), 7.44 (d, *J* = 7.6 Hz, 1H), 7.30 (d, *J* = 8.0 Hz, 1H), 7.15 (td, *J* = 8.0, 7.5, 1.3 Hz, 1H), 7.11 (td, *J* = 7.5, 1.2 Hz, 1H), 3.59 (tt, *J* = 7.2, 1.9 Hz, 2H), 3.13 (tt, *J* = 7.1, 2.0 Hz, 2H), 2.45 (s, 3H) ppm.

2-(tert-butyl)-3-(2-isocyanoethyl)-1H-indole (1c)

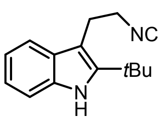

2-(tert-butyl)-3-(2-isocyanoethyl)-1H-indole was in stock and can be synthesized via well-established procedure (General procedure A).[8] The ^{1}H-NMR data matched the data reported in literature.[8] **^{1}H NMR** (300 MHz, $CDCl_3$): δ 7.94 (s, 1H), 7.47 (d, *J* = 7.5 Hz, 1H), 7.32 (d, *J* = 7.2 Hz, 1H), 7.19 – 7.08 (m, 2H), 3.62 (t, *J* = 8.1 Hz, 2H), 3.37 (t, *J* = 8.3 Hz, 2H), 1.49 (s, 9H) ppm.

3-(2-isocyanoethyl)-5-methoxy-2-methyl-1H-indole (1d)

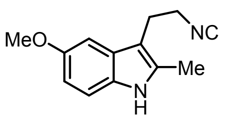

3-(2-isocyanoethyl)-5-methoxy-2-methyl-1H-indole was prepared according to a slight adaptation from General procedure B; the reaction was performed at 60 ^{o}C instead of room temperature. The reaction was executed using 5-methoxy-2-methyl-1H-indole (2.139 g, 14.4 mmol, 1.0 equiv.), *N*-(2,2-dimethoxyethyl)formamide (2.11 g mg, 15.8 mmol, 1.1 equiv.), TES (6.9 mL, 43.2 mmol, 3.0 equiv.) and TFA (5.5 mL, 71.9 mmol, 5.0 equiv.).The crude formamide was purified by FCC (eluent CH_2Cl_2:MeOH = 95:5) to obtain the compound as yellow oil (485 mg, 2.09 mmol, 15%). The formamide (595 mg, 2.54 mmol, 1.0 equiv.) was converted to the isocyanide utilizing Et_3N (1.77 mL, 12.7 mmol, 5.0 equiv.) and $POCl_3$ (0.35 mL, 3.81 mmol, 1.5 equiv.). The crude mixture was purified by FCC, using CH_2Cl_2 as eluent to obtain the title compound as a yellow solid (366 mg, 1.71 mmol, 67 %). **R_f** = 0.82 (CH_2Cl_2:MeOH = 97:3); The ^{1}H-NMR data matched the data reported in literature.[14] **^{1}H NMR** (300 MHz, $CDCl_3$): δ 7.74 (s, 1H), 7.21 (d, *J* = 8.7 Hz, 1H), 6.88 (d, *J* = 2.4 Hz, 1H), 6.79 (dd, *J* = 8.7, 2.4 Hz, 1H), 3.88 (s, 3H), 3.57 (t, *J* = 7.3, 2H), 3.09 (t, *J* = 7.2, 2H), 2.43 (s, 3H) ppm.

3-(2-isocyanoethyl)-2,5-dimethyl-1H-indole (1e)

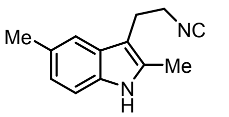

3-(2-isocyanoethyl)-2,5-dimethyl-1H-indole was prepared according to General procedure B, starting from 2,5-dimethyl-1H-indole (2.50 g, 17.2 mmol, 1.0 equiv.), *N*-(2,2-dimethoxyethyl)formamide (2.53 mg, 19.0 mmol, 1.1 equiv.), TES (8.2 mL, 51.6 mmol, 3.0 equiv.) and TFA (6.6 mL, 86.2 mmol, 5.0 equiv.).The crude formamide was purified by FCC (eluent cHex:EtOAc = 1:2) to obtain the compound as a light-yellow solid (2.233 g, 10.32 mmol, 90%). Next, the purified formamide (2.23 g, 10.3 mmol, 1.0 equiv.) was converted to the isocyanide utilizing Et_3N (7.2 mL, 51.6 mmol, 5.0 equiv.) and $POCl_3$ (1.42 mL, 15.5 mmol, 1.5 equiv.). The crude mixture was purified by FCC, using CH_2Cl_2 as eluent to obtain the title compound as a white solid (1.72 g, 8.65 mmol, 84 %). **R_f** = 0.92 (*c*Hex:EtOAc = 1:3); The ^{1}H-NMR data matched the data reported in literature.[14] **^{1}H NMR** (300 MHz, $CDCl_3$): δ 7.75 (s, 1H), 7.21 (s, 1H), 7.18 (d, *J* = 8.2 Hz, 1H), 6.96 (dd, *J* = 8.2, 1.6 Hz, 1H), 3.58 (tt, *J* = 7.5, 1.5 Hz, 2H), 3.09 (tt, *J* = 7.3, 1.6 Hz, 2H), 2.44 (s, 3H), 2.43 (s, 3H) ppm.

5-fluoro-3-(2-isocyanoethyl)-2-methyl-1H-indole (1f)

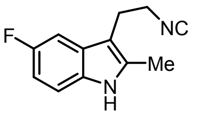

5-fluoro-3-(2-isocyanoethyl)-2-methyl-1H-indole was prepared according to General procedure B, starting from 5-fluoro-2-methyl-1H-indole (3.00 g, 20.1 mmol, 1.0 equiv.), *N*-(2,2-dimethoxyethyl)formamide (2.975 g, 22.3 mmol, 1.1 equiv.), TES (9.6 mL, 60.3 mmol, 3.0 equiv.) and TFA (7.7 mL, 101 mmol, 5.0 equiv.).The crude formamide was purified by FCC (eluent EtOAc:cHex = 2:1) to obtain the compound as a light-yellow oil (2.92 g, 13.3 mmol, 66%). Next, the purified formamide (2.39 g, 10.9 mmol, 1.0 equiv.) was converted to the isocyanide utilizing Et_3N (7.55 mg, 54.4 mmol, 5.0 equiv.) and $POCl_3$ (1.5 mL, 16.3 mmol, 1.5 equiv.). The crude mixture was purified by FCC, using CH_2Cl_2 as eluent to obtain the title compound as a yellow solid (1.70 g, 8.39 mmol, 77 %). **R_f** = 0.72 (*c*Hex:EtOAc = 1:2); The ^{1}H-NMR data matched the data reported in literature.[14] **^{1}H NMR** (300 MHz, $CDCl_3$): δ 7.86 (s, 1H), 7.18 (dd, *J* = 8.7, 4.4 Hz, 1H), 7.06 (dd, *J* = 9.5, 2.5 Hz, 1H), 6.88 (td, *J* = 9.0, 2.4 Hz, 1H), 3.58 (tt, *J* = 7.0, 1.8 Hz, 2H), 3.07 (tt, *J* = 7.1, 2.0 Hz, 2H), 2.45 (s, 3H) ppm.

4-bromo-3-(2-isocyanoethyl)-2-methyl-1H-indole (1g)

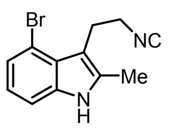

4-bromo-3-(2-isocyanoethyl)-2-methyl-1H-indole was prepared according to General procedure B, starting from 4-bromo-2-methyl-1H-indole (1.0 g mg, 4.76 mmol, 1.0 equiv.), *N*-(2,2-dimethoxyethyl)formamide (0.70 g, 5.26 mmol, 1.1 equiv.), TES (2.28 mL, 14.3 mmol, 3.0 equiv.) and TFA (1.82 mL, 23.8 mmol, 5.0 equiv.).The crude formamide was purified by FCC (eluent CH_2Cl_2:MeOH = 99:1) to obtain the compound as a light-yellow solid (0.47 g, 1.67 mmol, 35%). Next, the purified formamide (411 mg, 1.57 mmol, 1.0 equiv.) was converted to the isocyanide utilizing Et_3N (1.10 mL, 7.85 mmol, 5.0 equiv.) and $POCl_3$ (0.22 mL, 2.35 mmol, 1.5 equiv.). The crude mixture was purified by FCC, using CH_2Cl_2:MeOH = 95:5 as eluent to obtain the title compound as a light-yellow solid (351 mg, 1.33 mmol, 85 %). **R_f** = 0.78 (MeOH: CH_2Cl_2 5:95); **^{1}H NMR** (500 MHz, DMSO-*d6*) δ (ppm): 11.31 (s, 1H), 7.29 (dd, *J* = 8.0, 0.9 Hz, 1H), 7.14 (dd, *J* = 7.6, 0.9 Hz, 1H), 6.91 (t, *J* = 7.8 Hz, 1H), 3.68 (t, *J* = 7.1, 2H), 3.22 (t=, *J* = 7.1 Hz, 2H), 2.39 (s, 3H); **^{13}C{^{1}H} NMR** (126 MHz, DMSO-

*d*6) δ 155.9 (C_q), 136.6 (C_q), 135.9 (C_q), 125.3 (C_q), 122.8 (CH), 121.3 (CH), 111.5 (C_q), 110.4 (CH), 105.6 (C_q), 43.2 (t, *J* = 5.7 Hz, CH_2), 24.2 (CH_2), 11.4 (CH_3) ppm; **HRMS (ESI):** m/z calculated for $C_{12}H_{12}BrN_2$ [M+H$^+$] = 263.0178, found = 263.0183.

5-bromo-3-(2-isocyanoethyl)-2-methyl-1H-indole (1h)

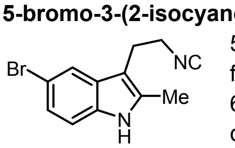

5-bromo-3-(2-isocyanoethyl)-2-methyl-1H-indole was prepared according to General procedure B, starting from 5-bromo-2-methyl-1H-indole (1.20 g, 5.17 mmol, 1.0 equiv.), *N*-(2,2-dimethoxyethyl)formamide (0.84 g, 6.31 mmol, 1.1 equiv.), TES (2.27 mL, 14.3 mmol, 3.0 equiv.) and TFA (1.82 mL, 23.8 mmol, 5.0 equiv.). The crude formamide was purified by FCC (eluent EtOAc:cHex = 2:1) to obtain the compound as a slight pink solid (1.012 g, 3.60 mmol, 63%). Next, the purified formamide (926 mg, 3.29 mmol, 1.0 equiv.) was converted to the isocyanide utilizing Et_3N (2.3 mL, 16.5 mmol, 5.0 equiv.) and $POCl_3$ (0.45 mL, 4.94 mmol, 1.5 equiv.). The crude mixture was purified by FCC, using CH_2Cl_2 as eluent to obtain the title compound as a yellow solid (678 mg, 2.56 mmol, 78 %). $\mathbf{R_f}$ = 0.88 (*c*Hex:EtOAc = 1:2); **^{1}H NMR** (500 MHz, $CDCl_3$): δ 8.04 (s, 1H), 7.53 (d, *J* = 1.8 Hz, 1H), 7.21 (dd, *J* = 8.5, 1.8 Hz, 1H), 7.14 (d, *J* = 8.5 Hz, 1H), 3.57 (tt, *J* = 7.0, 1.7 Hz, 2H), 3.05 (tt, *J* = 7.0, 2.0 Hz, 2H), 2.42 (s, 3H) ppm; **^{13}C{^{1}H} NMR** (126 MHz, $CDCl_3$): δ 156.2 (C_q, t, *J* = 5.7 Hz), 134.5 (C_q), 134.0 (C_q), 129.8 (C_q), 124.2 (CH), 119.9 (CH), 112.8 (C_q), 112.1 (CH), 106.4 (C_q), 42.0 (CH_2, t, *J* = 6.5 Hz), 24.7 (CH_2), 11.9 (CH_3) ppm. **HRMS (ESI):** m/z calculated for $C_{12}H_{12}BrN_2$ [M+H$^+$] = 263.0178, found = 263.0174.

7-bromo-3-(2-isocyanoethyl)-2-methyl-1H-indole (1i)

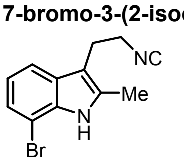

7-bromo-3-(2-isocyanoethyl)-2-methyl-1H-indole was prepared according to General procedure B, starting from 7-bromo-2-methyl-1H-indole (1.0 g, 4.76 mmol, 1.0 equiv.), *N*-(2,2-dimethoxyethyl)formamide (0.71 g, 5.33 mmol, 1.1 equiv.), TES (2.3 mL, 14.3 mmol, 3.0 equiv.) and TFA (1.8 mL, 23.8 mmol, 5.0 equiv.).The crude formamide was purified by FCC (eluent EtOAc:*c*Hex = 2:3) to obtain the compound as a yellow solid (0.753 g, 2.68 mmol, 56%). Next, the purified formamide (753 mg, 2.68 mmol, 1.0 equiv.) was converted to the isocyanide utilizing Et_3N (1.86 mL, 13.4 mmol, 5.0 equiv.) and $POCl_3$ (0.37 mL, 4.02 mmol, 1.5 equiv.). The crude mixture was purified by FCC, using CH_2Cl_2 as eluent to obtain the title compound as a white solid (639 mg, 2.43 mmol, 91 %). $\mathbf{R_f}$ = 0.92 (*c*Hex:EtOAc = 2:3); **^{1}H NMR** (500 MHz, $CDCl_3$): δ 8.10 (s, 1H), 7.38 (d, *J* = 7.8 Hz, 1H), 7.29 (d, *J* = 7.6 Hz, 1H), 6.99 (t, *J* = 7.8 Hz, 1H), 3.58 (tt, *J* = 7.1, 1.6 Hz, 2H), 3.09 (tt, *J* = 6.9, 1.7 Hz, 2H), 2.47 (s, 3H) ppm; **^{13}C{^{1}H} NMR** (126 MHz, $CDCl_3$): δ 156.5 (C_q, t, *J* = 5.7 Hz), 134.0 (C_q), 133.8 (C_q), 129.2 (C_q), 123.8 (CH), 120.9 (CH), 116.6 (CH), 108.0 (C_q), 104.3 (C_q), 42.1 (CH_2, t, *J* = 6.5 Hz), 25.1 (CH_2), 11.9 (CH_3) ppm. **HRMS (ESI):** m/z calculated for $C_{12}H_{12}BrN_2$ [M+H$^+$] = 263.0178, found = 263.0176.

3-(2-isocyanoethyl)-2-phenyl-1H-indole (1j)

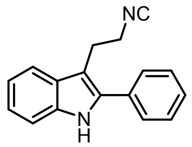

3-(2-isocyanoethyl)-2-phenyl-1H-indole was in stock and can be synthesized via a well-established procedure (General procedure B).[8] The ^{1}H-NMR data matched the data reported in literature.[8] **^{1}H NMR** (300 MHz, $CDCl_3$): δ 8.13 (s, 1H), 7.64 – 7.48 (m, 4H), 7.47 – 7.35 (m, 2H), 7.29 – 7.12 (m, 3H), 3.65 (t, *J* = 7.8 Hz, 2H), 3.35 (t, *J* = 7.7 Hz, 2H).ppm.

3-(2-isocyanoethyl)-2-(p-tolyl)-1H-indole (1k)

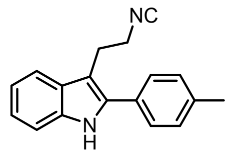

3-(2-isocyanoethyl)-2-(p-tolyl)-1H-indole was prepared according to General procedure B, starting from 2-(p-tolyl)-1H-indole (1.0 g, 4.82 mmol, 1.0 equiv.), *N*-(2,2-dimethoxyethyl)formamide (0.71 g, 5.31 mmol, 1.1 equiv.), TES (2.31 mL, 14.5 mmol, 3.0 equiv.) and TFA (1.85 mL, 24.1 mmol, 5.0 equiv.).The crude formamide was used in the next step. Next, the crude formamide (701 mg, 2.52 mmol, 1.0 equiv.) was converted to the isocyanide utilizing Et_3N (1.5 mL, x 10.8 mmol, 5.0 equiv.) and $POCl_3$ (0.4 mL, 4.28 mmol, 1.5 equiv.). The crude mixture was purified by FCC, using EtOAc:cHex = 1:4 as eluent to obtain the title compound as a light brown solid (371 mg, 1.43 mmol, 30% (2 steps)). $\mathbf{R_f}$ = 0.37 (EtOAc:cHex = 1:4); **^{1}H NMR** (500 MHz, $CDCl_3$): δ 8.20 (s, 1H), 7.62 (dd, *J* = 7.8, 1.1 Hz, 1H), 7.45 (d, *J* = 8.2 Hz, 2H), 7.39 (d, *J* = 8.1, 1H), 7.34 (d, *J* = 7.9 Hz, 2H), 7.27 (ddd, *J* = 8.1, 7.7, 1.2 Hz, 1H), 7.22 (ddd, *J* = 8.1, 7.6, 1.1 Hz, 1H), 3.64 (t, *J* = 7.7 Hz, 2H), 3.35 (t, *J* = 7.8 Hz, 2H), 2.48 (s, 3H) ppm; **^{13}C{^{1}H} NMR** (126 MHz, $CDCl_3$): δ 156.0 (t, *J* = 5.7 Hz, C_q), 138.3 (C_q), 136.2 (C_q), 135.7 (C_q), 129.8 (CH), 129.5 (C_q), 128.4 (C_q), 128.1 (CH), 122.5 (CH), 120.0 (CH), 118.3 (CH), 111.2 (CH), 107.1 (C_q), 41.7 (t, *J* = 6.3 Hz, CH_2), 25.4 (CH_2), 21.3 (CH_3) ppm; **HRMS (ESI):** m/z calculated for $C_{18}H_{17}N_2$ [M+H$^+$] = 261.1386, found = 261.1374.

2-(4-fluorophenyl)-3-(2-isocyanoethyl)-1H-indole (1l)

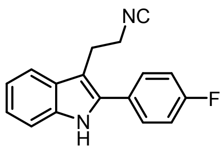

2-(4-fluorophenyl)-3-(2-isocyanoethyl)-1H-indole was prepared according to General procedure B, starting from 2-(4-fluorophenyl)-1H-indole (0.95 g, 4.5o mmol, 1.0 equiv.), *N*-(2,2-dimethoxyethyl)formamide (0.69 g, 5.18 mmol, 1.1 equiv.), TES (2.26 mL, 14.2 mmol, 3.0 equiv.) and TFA (1.81 mL, 23.7 mmol, 5.0 equiv.).The crude formamide was purified by FCC with eluent EtOAc:*c*Hex = 2:3 to obtain the compound as a light-yellow solid (0.672 g, 2.38 mmol, 53%). Next, the purified formamide (593 mg, 2.11 mmol, 1.0 equiv.) was converted to the isocyanide utilizing Et_3N (1.47 mL, 10.6 mmol, 5.0 equiv.) and $POCl_3$ (0.29 mL, 3.17 mmol, 1.5 equiv.). The crude mixture was purified by FCC, using CH_2Cl_2 as eluent to obtain the title compound as a yellow solid (434 mg, 1.64 mmol, 78 %). $\mathbf{R_f}$ = 0.66 (cHex:EtOAc = 3:2); **^{1}H NMR** (500 MHz, $CDCl_3$): δ 8.16 (s, 1H), 7.59 (d, *J* = 7.8 Hz, 1H), 7.55 – 7.48 (m, 2H), 7.40 (d, *J f*= 8.0 Hz, 1H), 7.26 (t, *J* = 7.5 Hz, 1H), 7.23 – 7.16 (m, 3H), 3.65 (t, *J* = 7.5 Hz, 2H), 3.29 (t, *J* = 7.5 Hz, 2H) ppm; **^{13}C{^{1}H} NMR** (126 MHz, $CDCl_3$): δ 162.8 (d, *J* = 249 Hz, C_q), 156.4 (t, *J* = 5.6 Hz, C_q), 135.8 (C_q), 135.3 (C_q), 130.2 (CH, d, *J* = 8.2 Hz), 128.6 (C_q, d, *J* = 3.4 Hz), 128.3 (C_q), 122.9 (CH), 120.3 (CH), 118.5 (CH), 116.3 (d, *J* = 21.7 Hz, CH), 111.3 (CH), 107.7 (C_q),

41.8 (CH_2, t, *J* = 6.3 Hz), 25.3 (CH_2) ppm. **^{19}F{^{1}H} NMR** (470 MHz, $CDCl_3$): δ -112.7 ppm; **HRMS (ESI):** m/z calculated for $C_{17}H_{14}FN_2$ [M+H$^+$] = 265.1136, found = 265.1134.

2-(4-chlorophenyl)-3-(2-isocyanoethyl)-1H-indole (1m)

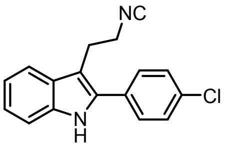

2-(4-chlorophenyl)-3-(2-isocyanoethyl)-1H-indole was prepared according to General procedure B, starting from 2-(4-chlorophenyl)-1H-indole (1.0 g, 4.39 mmol, 1.0 equiv.), *N*-(2,2-dimethoxyethyl)formamide (650 mg, 4.88 mmol, 1.1 equiv.), TES (2.1 mL, 13.2 mmol, 3.0 equiv.) and TFA (1.7 mL, 22.0 mmol, 5.0 equiv.).The crude formamide was purified by FCC (eluent EtOAc:cHex =2:3) to obtain the compound as a light-yellow solid (1.22 g, 4.09 mmol, 93%). Next, the purified formamide was (1.08 g, 3.62 mmol, 1.0 equiv.) converted to the isocyanide utilizing Et_3N (2.5 mL, 18.1 mmol, 5.0 equiv.) and $POCl_3$ (0.5 mL, 5.42 mmol, 1.5 equiv.). The crude mixture was purified by FCC, using CH_2Cl_2 as eluent to obtain the title compound as a yellow solid (346 mg, 1.23 mmol, 34%). **R_f** = 0.71 (*c*Hex:EtOAc 3:2); **^{1}H NMR** (500 MHz, $CDCl_3$): δ 8.22 (s, 1H), 7.59 (d, *J* = 7.8 Hz, 1H), 7.51-7.43 (m, 4H) 7.39 (d, *J* = 8.1 Hz, 1H), 7.26 (t, *J* = 7.2 Hz, 1H), 7.20 (t, *J* = 7.5 Hz, 1H), 3.65 (t, *J* = 7.5 Hz, 2H), 3.30 (t, *J* = 7.5 Hz, 2H) ppm; **^{13}C{^{1}H} NMR** (126 MHz, $CDCl_3$): δ 156.4 (t, *J* = 5.6 Hz, C_q), 136.0 (C_q), 135.0 (C_q), 134.4 (C_q), 130.9 (C_q), 129.6 (CH), 129.4 (CH), 128.3 (C_q), 123.0 (CH), 120.4 (CH), 118.3 (CH), 111.3 (CH), 108.0 (C_q), 41.8 (t, *J* = 6.3 Hz, CH_2), 25.2 (CH_2) ppm.; **HRMS (ESI):** m/z calculated for $C_{17}H_{14}ClN_2$ [M+H$^+$] = 281.0840, found = 281.0838.

3-(2-isocyanoethyl)-2-(naphthalen-2-yl)-1H-indole (1n)

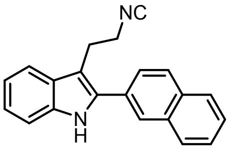

3-(2-isocyanoethyl)-2-(naphthalen-2-yl)-1H-indole was prepared according to General procedure B, starting from 2-(naphthalen-2-yl)-1H-indole (1.0 g, 4.11 mmol, 1.0 equiv.), *N*-(2,2-dimethoxyethyl)formamide (602 mg, 4.52 mmol, 1.1 equiv.), TES (1.97 mL, 12.33 mmol, 3.0 equiv.) and TFA (1.57 mL, 20.55 mmol, 5.0 equiv.).The crude formamide was purified by FCC (eluent EtOAc:cHex = 1:4) to obtain the compound as a orange solid (611 mg, 2.06 mmol, 47 %). Next, the purified formamide (593 mg, 1.89 mmol, 1.0 equiv.) was converted to the isocyanide utilizing Et_3N (1.32 mg, 9.45 mmol, 5.0 equiv.) and $POCl_3$ (265 µL, 2.84 mmol, 1.5 equiv.). The title compound was isolated as a light-yellow solid (611 mg, 2.06 mmol, 76 %). **R_f** = 0.80 (CH_2Cl_2); **^{1}H NMR** (500 MHz, $CDCl_3$): δ 8.27 (s, 1H), 8.01 (s, 1H), 7.97 (d, *J* = 8.4 Hz, 1H), 7.94 – 7.87 (m, 2H), 7.66 (dd, *J* = 8.5, 1.8 Hz, 1H), 7.63 (dd, *J* = 7.8, 1.0 Hz, 1H), 7.59 – 7.53 (m, 2H), 7.43 (d, *J* = 8.0 Hz, 1H), 7.30 – 7.25 (m, 1H), 7.21 (ddd, *J* = 8.0, 7.1, 1.1 Hz, 1H), 3.67 (t, *J* = 7.5 Hz, 2H), 3.41 (t, *J* = 7.7 Hz, 2H) ppm. **^{13}C{^{1}H} NMR** (126 MHz, $CDCl_3$): δ 156.4 (t, *J* = 5.8 Hz, C_q), 136.2 (C_q), 136.1 (C_q), 133.5 (C_q), 133.0 (C_q), 129.9 (C_q), 129.0 (CH), 128.6 (CH), 128.3 (CH), 128.0 (CH), 127.4 (CH), 127.0 (CH), 126.8 (CH), 125.9 (CH), 122.9 (CH), 120.3 (CH), 118.6 (CH), 111.3 (CH), 108.1 (C_q), 41.9 (t, *J* = 6.2 Hz, CH_2), 25.5 (CH_2) ppm. **HRMS (ESI):** m/z calculated for $C_{21}H_{17}N_2$ [M+H$^+$] = 297.1386, found = 297.1380.

2-bromo-3-(2-isocyanoethyl)-1H-indole (1o)

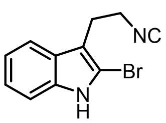

2-bromo-3-(2-isocyanoethyl)-1H-indole was in stock and can be synthesized via well-established procedure (General procedure A).[8] The obtained ^{1}H-NMR matched the data reported in literature.[8] **^{1}H NMR** (600 MHz, $CDCl_3$): δ 8.19 (s, 1H), 7.51 (d, *J* = 7.8 Hz, 1H), 7.30 (d, *J* = 8.1 Hz, 1H), 7.21 (t, *J* = 7.6 Hz, 1H), 7.15 (t, *J* = 7.6 Hz, 1H), 3.62 (t, *J* = 7.2 Hz, 2H), 3.17 (t, *J* = 7.5 Hz, 2H) ppm.

methyl 2-(3-(2-isocyanoethyl)-1H-indol-2-yl)acetate (1p)

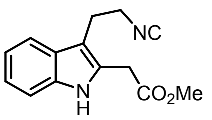

methyl 2-(3-(2-isocyanoethyl)-1H-indol-2-yl)acetate was prepared according to General procedure B starting from methyl 2-(1H-indo l-2-yl)acetate (2.095 g, 11.07 mmol, 1.0 equiv.), *N*-(2,2-dimethoxyethyl)formamide (1.622 g, 12.18 mmol, 1.1 equiv.) and TES (5.30 mL, 33.2 mmol, 3.0 equiv.) and TFA (4.26 mL, 55.4 mmol, 5.0 equiv.). The crude formamide was purified by FCC (eluent EtOAc:cHex = 1.5:1) to obtain the compound as a brown oil (1.536 g, 5.9 mmol, 53%). Next, the purified formamide (1.536 g, 5.9 mmol, 1.0 equiv.) was converted to the isocyanide utilizing Et_3N (4.11 mL, 29.5 mmol, 5.0 equiv.) and $POCl_3$ (0.83, 8.85 mmol, 1.5 equiv.). The crude mixture was purified by FCC, using CH_2Cl_2 as eluent to obtain the title compound as an orange oil (800 mg, 3.3 mmol, 56 %). **R_f** = 0.5 (EtOAc/cHex = 1.5:1)); **^{1}H NMR** (600 MHz, $CDCl_3$): δ 8.69 (s, 1H), 7.48 (d, *J* = 8.3 Hz, 1H), 7.36 (d, *J* = 8.1 Hz, 1H), 7.20 (ddd, *J* = 8.1, 7.0, 1.2 Hz, 1H), 7.13 (ddd, *J* = 7.9, 7.0, 0.8 Hz, 1H), 3.87 (s, 2H), 3.76 (s, 3H), 3.61 (t, *J* = 7.1 Hz, 2H), 3.15 (tt, *J* = 7.1, 1.9 Hz, 2H). ppm**;** **^{13}C{^{1}H} NMR** (150 MHz, $CDCl_3$): δ 170.8 (C_q), 156.5 (t, *J* = 5.4 Hz, C_q) 135.8 (C_q), 128.3 (C_q), 127.3 (C_q), 122.4 (CH), 119.9 (CH), 117.9 (CH), 111.3 (CH), 108.4 (C_q), 52.7 (CH_3), 42.1 (CH_2), 31.8 (CH_2), 24.9 (CH_2) ppm; **HRMS (ESI):** m/z calculated for $C_{14}H_{15}N_2O_2$ [M+H$^+$] = 243.1128 , found = 243.1128.

3-(2-isocyanoethyl)-5-methoxy-1H-indole (1q)

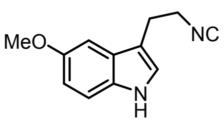

3-(2-isocyanoethyl)-5-methoxy-1H-indole was synthesized according to General procedure A, starting from 2-(5-methoxy-1H-indol-3-yl)ethan-1-amine (1.044 g, 5.49 mmol, 1.0 equiv.) and ethyl formate (5.0 mL mmol). The crude formamide was used direclty in the next step. Next, the formamide was converted to the isocyanide utilizing Et_3N (3.82 mL, 27.4 mmol, 5.0 equiv.) and $POCl_3$ (767 µL, 7.73 mmol, 1.5 equiv.). The crude mixture was purified by FCC, using CH_2Cl_2:MeOH =98:2 as eluent. The title compound was isolated as a brown oil (772 mg, 3.86 mmol, 70% (2 steps)). The characterization data matched the data reported in literature.[9] **R_f** = 0.78 (MeOH: CH_2Cl_2 = 2: 98); **^{1}H NMR** (500 MHz, $CDCl_3$): δ 7.99 (s, 1H), 7.28 (d, *J* = 8.8 Hz, 1H), 7.26 (s, 1H), 7.12 (d, *J* = 2.5 Hz, 1H), 6.98 (d, *J* = 2.4 Hz, 1H), 6.89 (dd, *J* = 8.7, 2.4 Hz, 1H), 3.87 (s, 3H), 3.66 (tt, *J* = 7.1, 1.8 Hz, 2H), 3.14 (tt, *J* = 7.2, 2.0 Hz, 2H) ppm.

3-(2-isocyanoethyl)-6-methoxy-1H-indole (1r)

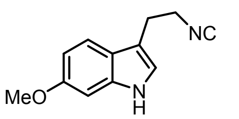

3-(2-isocyanoethyl)-6-methoxy-1H-indole was synthesized according to General procedure A, starting from 2-(6-methoxy-1H-indol-3-yl)ethan-1-amine (530 mg, 2.79 mmol, 1.0 equiv.) and ethyl formate (5 mL). The crude formamide was used direclty in the next step. Next, the formamide was converted to the isocyanide utilizing Et_3N (1.94 mL, 13.9 mmol, 5.0 equiv.) and $POCl_3$ (0.39 mL, 4.18 mmol, 1.5 equiv.). The crude mixture was purified by FCC, using CH_2Cl_2:MeOH =97:3 as eluent. The title compound was isolated as a yellow solid (426 mg, 2.13 mmol, 77% (2 steps)). The characterization data matched the data reported in literature.[10] **R_f** = 0.75 (MeOH: CH_2Cl_2 = 3: 97); **^{1}H NMR** (500 MHz, $CDCl_3$): δ 7.94 (s, 1H), 7.42 (d, *J* = 8.7 Hz, 1H), 7.03 (d, *J* = 2.2 Hz, 1H), 6.88 (d, *J* = 2.2 Hz, 1H), 6.82 (dd, *J* = 8.6, 2.2 Hz, 1H), 3.85 (s, 3H), 3.66 (t, *J* = 7.1 Hz, 2H), 3.14 (t, *J* = 7.0 Hz, 2H) ppm.

3-(2-isocyanoethyl)-5-methyl-1H-indole (1s)

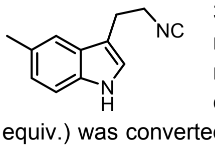

3-(2-isocyanoethyl)-5-methyl-1H-indole was synthesized according to General procedure A, starting from 5-methyltryptamine hydrochloride (1.04 g, 4.95 mmol, 1.0 equiv.), ethyl formate (3.8 mL, 51.7 mmol) and Et_3N (6.6 mL, 47.5 mmol). The crude formamide was purified by FCC (eluent CH_2Cl_2:MeOH = 97:3) to obtain the compound as a yellow solid (0.845 g, 4.18 mmol, 71%). Next, the purified formamide (800 mg, 3.96 mmol, 1.0 equiv.) was converted to the isocyanide utilizing Et_3N (2.75 mL, 19.8 mmol, 5.0 equiv.) and $POCl_3$ (0.54 mL, 5.90 mmol, 1.5 equiv.). The crude mixture was purified by FCC, using CH_2Cl_2 as eluent. The title compound was isolated as a light-yellow solid (628 mg, 3.41 mmol, 86%). The characterization data matched the data reported in literature.[9] **R_f** = 0.7 (*c*Hex:EtOAc = 1:1); **^{1}H NMR** (300 MHz, $CDCl_3$): δ 8.02 (s, 1H), 7.35 (s, 1H), 7.28 (d, J = 8.3 Hz, 1H), 7.11 – 7.03 (m, 2H), 3.67 (tt, J = 7.1, 1.8 Hz, 2H), 3.15 (tt, J = 7.1, 2.0 Hz, 2H), 2.49 (s, 3H) ppm.

5-fluoro-3-(2-isocyanoethyl)-1H-indole (1t)

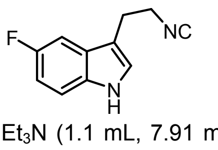

5-fluoro-3-(2-isocyanoethyl)-1H-indole was synthesized according to General procedure A, starting from 5-fluorotryptamine (0.494 g, 2.77 mmol, 1.0 equiv.), ethyl formate (2.3 mL, 28.1 mmol). The crude formamide was purified by FCC (eluent CH_2Cl_2:MeOH = 97:3) to obtain the compound as a yellow solid (332 mg, 1.61 mmol, 58%). Next, the purified formamide (325 mg, 1.58 mmol, 1.0 equiv.) was converted to the isocyanide utilizing Et_3N (1.1 mL, 7.91 mmol, 5.0 equiv.) and $POCl_3$ (0.22 mL, 2.37 mmol, 1.5 equiv.). The crude mixture was purified by FCC, using CH_2Cl_2 as eluent. The title compound was isolated as a light-yellow solid (265 mg, 1.41 mmol, 89%). The characterization data matched the data reported in literature.[9] **R_f** = 0.67 (*c*Hex:EtOAc = 1:1); **^{1}H NMR** (300 MHz, $CDCl_3$): δ 8.10 (s, 1H), 7.31 (dd, J = 8.8, 4.3 Hz, 1H), 7.23 – 7.15 (m, 2H), 6.97 (td, J = 9.0, 2.5 Hz, 1H), 3.66 (tt, J = 7.0, 1.9 Hz, 2H), 3.12 (tt, J = 6.3, 2.2 Hz, 2H) ppm.

5-chloro-3-(2-isocyanoethyl)-1H-indole (1u)

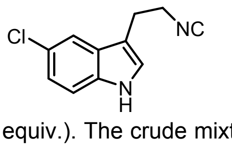

5-chloro-3-(2-isocyanoethyl)-1H-indole was synthesized according to General procedure A, starting from 5-chlorotryptamine hydrochloride (2.316 g, 10 mmol, 1.0 equiv.), ethyl formate (8.0 mL, 100 mmol) and Et_3N (2.8 mL, 20 mmol). The crude formamide was obtained as a brown solid. Next, the crude formamide was converted to the isocyanide utilizing Et_3N (7.0 mL, 50 mmol, 5.0 equiv.) and $POCl_3$ (1.4 mL, 15 mmol, 1.5 equiv.). The crude mixture was purified by FCC, using CH_2Cl_2 as eluent. The title compound was isolated as an off white solid (1.45 g, 7.1 mmol, 71% (2 steps)). The characterization data matched the data reported in literature.[9] **^{1}H NMR** (300 MHz, $CDCl_3$): δ 8.17 (s, 1H), 7.53 (d, *J* = 1.9 Hz, 1H), 7.31 (t, *J* = 8.1 Hz, 1H), 7.24-7.13 (m, 2H), 3.68 (t, *J* = 6.8 Hz, 2H), 3.14 (t, *J* = 6.9 Hz, 2H) ppm.

5-bromo-3-(2-isocyanoethyl)-1H-indole (1v)

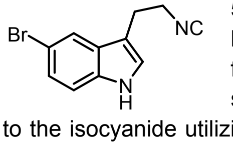

5-bromo-3-(2-isocyanoethyl)-1H-indole was synthesized according to General procedure A, starting from 5-bromotryptamine (369 mg, 1.54 mmol, 1.0 equiv.) and ethyl formate (2.0 mL, 24.8 mmol). The crude formamide was purified by FCC (eluent CH_2Cl_2:MeOH = 95:5) to obtain the compound as a brown-orange solid (391 mg, 1.46 mmol, 95%). Next, the purified formamide (391 mg, 1.46 mmol, 1.0 equiv.) was converted to the isocyanide utilizing Et_3N (1.0 mL, 7.2 mmol, 5.0 equiv.) and $POCl_3$ (0.20 mL, 2.18 mmol, 1.5 equiv.). The crude mixture was purified by FCC, using CH_2Cl_2 as eluent. The title compound was isolated as a light-yellow solid (277 mg, 1.11 mmol, 76 %). The characterization data matched the data reported in literature.[9] **R_f** = 0.88 (CH_2Cl_2:MeOH = 95:5); **^{1}H NMR** (300 MHz, $CDCl_3$): δ 8.16 (s, 1H), 7.68 (q, *J* = 0.8 Hz, 1H), 7.36 – 7.24 (m, 3H), 7.19 (d, *J* = 2.5 Hz, 1H), 3.67 (tt, *J* = 6.9, 1.8 Hz, 2H), 3.13 (tt, *J* = 6.2, 1.7 Hz, 2H) ppm.

methyl 3-(1H-indol-3-yl)-2-isocyanopropanoate (1w)

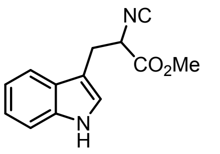

Methyl 3-(1H-indol-3-yl)-2-isocyanopropanoate was in stock and can be synthesized via well-established procedure.[11] ^{1}H-NMR data matched the data reported in literature.[11] **^{1}H NMR** (600 MHz, $CDCl_3$): δ 8.17 (s, 1H), 7.57 (d, *J* = 7.9 Hz, 1H), 7.39 (ddd, *J* = 8.1, 1.8, 0.9 Hz, 1H), 7.25 – 7.20 (m, 2H), 7.16 (t, *J* = 7.5 Hz, 1H), 4.55 (dd, *J* = 8.0, 4.7 Hz, 1H), 3.76 (s, 3H), 3.48 (dd, *J* = 14.7, 4.7 Hz, 1H), 3.36 (dd, *J* = 14.7, 8.0 Hz, 1H) ppm.

3-(2-isocyano-1-(3-methoxyphenyl)ethyl)-1H-indole (1x)

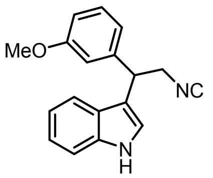

3-(2-isocyano-1-(3-methoxyphenyl)ethyl)-1H-indole was synthesized according to General procedure A, starting from 2-(1H-indol-3-yl)-2-(3-methoxyphenyl)ethan-1-amine (1.07 g, 3.76 mmol, 1.0 equiv.) and ethyl formate (3.0 mL, 37.6 mmol). The formamide was used in the next step without further purification. Next, the formamide (was converted to the isocyanide utilizing Et_3N (2.53 mL, 18.2 mmol, 5.0 equiv.) and $POCl_3$ (510 μL, 5.46 mmol, 1.5 equiv.). The crude mixture was purified by FCC, using EtOAc:cHex = 2:3 as eluent to obtain the isolated product as an orange oil (690 mg, 2.50 mmol, 66% (2 steps)). $\mathbf{R_f}$ = 0.80 (EtOAc:*c*Hex = 1:1); **^{1}H NMR** (500 MHz, $CDCl_3$): 8.15 (s, 1H), 7.41 – 7.34 (m, 2H), 7.27 (t, J = 7.9 Hz, 1H), 7.20 (ddd, J = 8.2, 7.1, 0.9 Hz, 1H), 7.12 – 7.03 (m, 2H), 6.95 (d, J = 7.9 Hz, 1H), 6.89 (t, J = 2.1 Hz, 1H), 6.82 (ddd, J = 8.3, 2.4, 0.7 Hz, 1H), 4.59 (t, J = 7.1 Hz, 1H), 4.11 – 3.91 (m, 2H), 3.78 (s, 3H).ppm. **^{13}C{^{1}H} NMR** (126 MHz, $CDCl_3$): δ 159.9 (C_q), 157.2 (C_q), 141.9 (C_q), 136.5 (C_q), 129.9 (CH), 126.5 (C_q), 122.6 (CH), 121.8 (CH), 120.4 (CH), 119.9 (CH), 119.1 (CH), 115.1 (C_q), 114.3 (CH), 112.5 (CH), 111.5 (CH), 55.3 (CH_3), 46.6 (t, *J* = 6.2 Hz, CH_2), 43.0 (CH) ppm. **HRMS (ESI):** m/z calculated for $C_{18}H_{17}N_2O$ [M+H$^+$] = 277.1335, found = 277.1346.

5. Scope tryptamine derived isocyanides

5.1 General procedure C: Iron catalyzed carbene transfer spirocyclisation cascade towards spiroindolenines

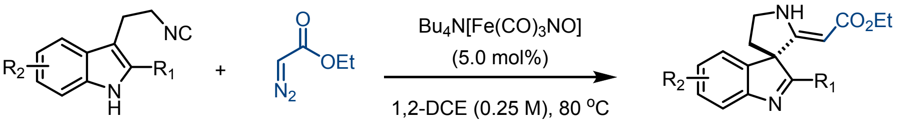

1a-q **22** **23a-q**

To a flame dried Schlenk flask under N_2 atmosphere, charged with a stirring bean, was added $Bu_4N[Fe(CO)_3NO]$ (10.3 mg, 0.025 mmol, 0.05 equiv.). Subsequently, 1,2-DCE was added (2 mL), and the mixture was stirred until the catalyst was dissolved. This was followed by the addition of tryptamine-derived isocyanide (0.5 mmol, 1.0 equiv.) and ethyl diazoacetate (**22**) (0.6 mmol, 1.2 equiv.). The solution was placed in a pre-heated oil bath and stirred at 80 °C until full conversion of the isocyanide was observed on TLC. Subsequently, the reaction mixture cooled to room temperature and directly subjected to purification by flash column chromatography, using a mixture of EtOAc:*c*Hex as eluent.

ethyl (Z)-2-(spiro[indole-3,3'-pyrrolidin]-2'-ylidene)acetate (23a)

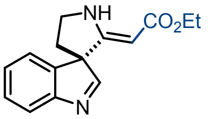

Ethyl (Z)-2-(spiro[indole-3,3'-pyrrolidin]-2'-ylidene)acetate was prepared according to General procedure C starting from 3-(2-isocyanoethyl)-1H-indole (85.3 mg, 0.50 mmol, 1.0 equiv.). The title compound was isolated via FCC using EtOAc:*c*Hex + 5% Et_3N as eluent to obtain the title compound as a light-yellow oil (124 mg, 0.48 mmol, 96 %). $\mathbf{R_f}$ = 0.30 (EtOAc:*c*Hex = 1:9 + 5% Et_3N); $\mathbf{^1H}$ **NMR** (600 MHz, $CDCl_3$) δ 8.11 (s, 1H), 7.99 (s, 1H), 7.63 (d, *J* = 7.7 Hz, 1H), 7.37 (td, *J* = 7.5, 1.5 Hz, 1H), 7.31 – 7.24 (m, 2H), 4.06 – 3.97 (m, 2H), 3.94 (s, 1H), 3.88 (dddd, *J* = 10.2, 7.8, 5.0, 1.0 Hz, 1H), 3.85 – 3.79 (m, 1H), 2.43 (ddd, *J* = 12.5, 7.4, 4.9 Hz, 1H), 2.31 (ddd, *J* = 12.9, 7.8, 6.7 Hz, 1H), 1.15 (t, *J* = 7.1 Hz, 3H).ppm; $\mathbf{^{13}C\{^1H\}}$ **NMR** (150 MHz, $CDCl_3$): δ 171.9 (CH), 170.5 (C_q), 162.0 (C_q), 155.6 (C_q), 140.1 (C_q), 128.9 (CH), 127.3 (CH), 122.3 (CH), 121.5 (CH), 77.4 (CH), 67.2 (C_q), 58.9 (CH_2), 46.0 (CH_2), 30.5 (CH_2), 14.5 (CH_3) ppm; **HRMS (ESI):** m/z calculated for $C_{15}H_{17}N_2O_2$ [M+H$^+$] = 257.1285, found = 257.1281.

ethyl (Z)-2-(2-methylspiro[indole-3,3'-pyrrolidin]-2'-ylidene)acetate (23b)

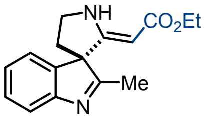

Ethyl (Z)-2-(2-methylspiro[indole-3,3'-pyrrolidin]-2'-ylidene)acetate was prepared according to General procedure C starting from 3-(2-isocyanoethyl)-2-methyl-1H-indole (91.4 mg, 0.50 mmol, 1.0 equiv.). The title compound was isolated as a yellow solid (112 mg, 0.41 mmol, 83%). $\mathbf{R_f}$ = 0.30 (cyclohexane:EtOAc 3:2); $\mathbf{^1H}$ **NMR** (500 MHz, $CDCl_3$): δ 8.14 (s, 1H), 7.51 (d, *J* = 7.7 Hz, 1H), 7.32 (td, *J* = 7.5, 1.3 Hz, 1H), 7.23 (d, *J* = 6.9 Hz, 1H), 7.17 (t, *J* = 7.2 Hz, 1H), 4.01 (q, *J* = 7.1 Hz, 2H), 3.94 – 3.79 (m, 2H), 3.88 (s, 1H), 2.39 – 2.29 (m, 2H), 2.27 (s, 3H), 1.16 (t, *J* = 7.1 Hz, 3H) ppm; $\mathbf{^{13}C\{^1H\}}$ **NMR** (126 MHz, $CDCl_3$): δ 181.8 (C_q), 170.8 (C_q), 164.2 (C_q), 155.2 (C_q), 142.1 (C_q), 128.8 (CH), 126.2 (CH), 122.1 (CH), 120.2 (CH), 77.1 (CH), 67.7 (C_q), 58.9 (CH_2), 46.0 (CH_2), 31.6 (CH_2), 16.4 (CH_3), 14.5 (CH_3) ppm. **HRMS (ESI):** m/z calculated for $C_{16}H_{19}N_2O_2$ [M+H$^+$] = 271.1441, found = 271.1446.

ethyl (Z)-2-(2-(tert-butyl)spiro[indole-3,3'-pyrrolidin]-2'-ylidene)acetate (23c)

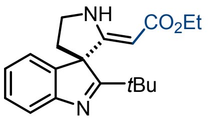

Ethyl (Z)-2-(2-(tert-butyl)spiro[indole-3,3'-pyrrolidin]-2'-ylidene)acetate was prepared according to General procedure C starting from 2-(tert-butyl)-3-(2-isocyanoethyl)-1H-indole (113.2 mg, 0.50 mmol, 1.0 equiv.). The title compound was isolated as a white solid (92.0 mg, 0.294 mmol, 59%). $\mathbf{R_f}$ = 0.35 (cyclohexane:EtOAc 4:1); $\mathbf{^1H}$ **NMR** (500 MHz, $CDCl_3$): δ 8.22 (s, 1H), 7.54 (dz, *J* = 7.7 Hz, 1H), 7.30 (ddd, *J* = 7.8, 5.1, 3.7 Hz, 1H), 7.18 – 7.14 (m, 2H), 4.06 – 3.91 (m, 5H), 2.89 (ddd, J = 13.5, 9.3, 7.7 Hz, 1H), 2.23 (ddd, J = 13.5, 8.0, 3.6 Hz, 1H).1.41 (s, 9H), 1.16 (t, *J* = 7.1 Hz, 3H) ppm; $\mathbf{^{13}C\{^1H\}}$ **NMR** (126 MHz, $CDCl_3$): δ 190.3 (C_q), 170.8 (C_q), 164.7 (C_q), 153.6 (C_q), 144.6 (C_q), 128.5 (CH), 126.4 (CH), 120.9 (CH), 120.3 (CH), 76.8 (CH), 68.1 (C_q), 58.8 (CH_2), 46.3 (CH_2), 38.1 (C_q), 30.3 (CH_3), 30.3 (CH_2), 14.6 (CH_3) ppm. **HRMS (ESI):** m/z calculated for $C_{19}H_{25}N_2O_2$ [M+H$^+$] = 313.1911, found = 313.1914.

ethyl (Z)-2-(5-methoxy-2-methylspiro[indole-3,3'-pyrrolidin]-2'-ylidene)acetate (23d)

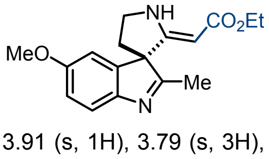

Ethyl (Z)-2-(5-methoxy-2-methylspiro[indole-3,3'-pyrrolidin]-2'-ylidene)acetate was prepared according to General procedure C starting from 3-(2-isocyanoethyl)-5-methoxy-2-methyl-1H-indole (107.3 mg, 0.50 mmol, 1.0 equiv.). The title compound was isolated as a yellow waxy solid (136 mg, 0.45 mmol, 90%). $\mathbf{R_f}$ = 0.19 (cyclohexane:EtOAc 2:1); $\mathbf{^1H}$ **NMR** (500 MHz, $CDCl_3$): δ 8.12 (s, 1H), 7.41 (d, *J* = 8.5 Hz, 1H), 6.83 (dd, *J* = 8.4, 2.5 Hz, 1H), 6.79 (d, *J* = 2.5 Hz, 1H), 4.03 (q, *J* = 7.1 Hz, 2H), 3.93 – 3.80 (m, 2H), 3.91 (s, 1H), 3.79 (s, 3H), 2.41 – 2.25 (m, 2H), 2.24 (s, 3H), 1.17 (t, *J* = 7.1 Hz, 3H) ppm; $\mathbf{^{13}C\{^1H\}}$ **NMR** (126 MHz, $CDCl_3$): δ 179.7 (C_q), 170.9 (C_q), 164.3 (C_q), 158.6 (C_q), 148.7 (C_q), 143.6 (C_q), 120.5 (CH), 113.3 (CH), 108.9 (CH), 77.3 (CH), 68.0 (C_q), 59.0 (CH_2), 55.8 (CH_3), 46.0 (CH_2), 31.8 (CH_2) 16.3 (CH_3), 14.6 (CH_3) ppm. **HRMS (ESI):** m/z calculated for $C_{17}H_{21}N_2O_3$ [M+H$^+$] = 301.1547, found = 301.1552.

ethyl (Z)-2-(2,5-dimethylspiro[indole-3,3'-pyrrolidin]-2'-ylidene)acetate (23e)

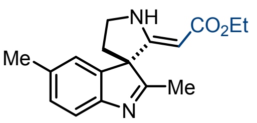

Ethyl (Z)-2-(2,5-dimethylspiro[indole-3,3'-pyrrolidin]-2'-ylidene)acetatewas prepared according to General procedure C starting from 3-(2-isocyanoethyl)-2,5-dimethyl-1H-indole (99.5 mg, 0.50 mmol, 1.0 equiv.). The title compound was isolated as a yellow waxy solid (116 mg, 0.41 mmol, 82%). $\mathbf{R_f}$ = 0.24 (*c*Hex:EtOAc 2:1); **^{1}H NMR** (500 MHz, $CDCl_3$): δ 8.14 (s, 1H), 7.39 (d, *J* = 7.8 Hz, 1H), 7.12 (d, *J* = 7.8 Hz, 1H), 7.05 (s, 1H), 4.03 (q, *J* = 7.1 Hz, 2H), 3.94 – 3.81 (m, 2H), 3.90 (s, 1H), 2.39 – 2.27 (m, 2H), 2.35 (s, 3H), 2.26 (s, 3H), 1.17 (t, *J* = 7.1 Hz, 3H) ppm; **^{13}C{^{1}H} NMR** (126 MHz, $CDCl_3$): δ 180.8 (C_q), 170.8 (C_q), 164.5 (C_q), 152.9 (C_q), 142.3 (C_q), 136.1 (C_q), 129.3 (CH), 122.9 (CH), 119.8 (CH), 77.1 (CH), 67.7 (C_q), 58.9 (CH_2), 46.0 (CH_2), 31.7 (CH_2), 21.5 (CH_3), 16.3 (CH_3), 14.6 (CH_3) ppm. **HRMS (ESI):** m/z calculated for $C_{17}H_{21}N_2O_2$ [M+H$^+$] = 285.1598, found = 285.1603.

ethyl (Z)-2-(5-fluoro-2-methylspiro[indole-3,3'-pyrrolidin]-2'-ylidene)acetate (23f)

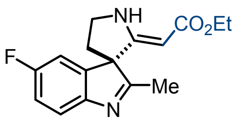

Ethyl (Z)-2-(5-fluoro-2-methylspiro[indole-3,3'-pyrrolidin]-2'-ylidene)acetate was prepared according to General procedure C starting from 5-fluoro-3-(2-isocyanoethyl)-2-methyl-1H-indole (101.3 mg, 0.5 mmol, 1.0 equiv.). The title compound was isolated as a light-yellow solid (140 mg, 0.49 mmol, 97%). $\mathbf{R_f}$ = 0.23 (cyclohexane:EtOAc 3:2); **^{1}H NMR** (500 MHz, $CDCl_3$): δ 8.13 (s, 1H), 7.44 (dd, *J* = 8.4, 4.6 Hz, 1H), 7.01 (td, *J* = 8.6, 2.5 Hz, 1H), 6.95 (dd, *J* = 7.8, 2.6 Hz, 1H), 4.03 (q, *J* = 7.1 Hz, 2H), 3.94 – 3.80 (m, 2H), 3.89 (s, 1H), 2.41 – 2.27 (m, 2H), 2.26 (s, 3H), 1.17 (t, *J* = 7.1 Hz, 3H) ppm; **^{13}C{^{1}H} NMR** (126 MHz, $CDCl_3$): δ 181.7 (C_q, d, *J* = 3.6 Hz), 170.7 (C_q), 163.4 (C_q), 161.5 (d, *J* = 245.2 Hz, C_q), 151.2 (C_q, d, *J* = 2.3 Hz), 143.9 (C_q, d, *J* = 8.8 Hz), 120.9 (CH, d, *J* = 8.8 Hz), 115.4 (CH, d, *J* = 23.6 Hz), 110.1 (CH, d, *J* = 25.1 Hz), 77.3 (CH), 68.2 (C_q, d, *J* = 2.3 Hz), 59.0 (CH_2), 45.9 (CH_2), 31.6 (CH_2), 16.3 (CH_3), 14.5 (CH_3) ppm; **^{19}F{^{1}H} NMR** (470.4 MHz, $CDCl_3$): δ -115.90 ppm; **HRMS (ESI):** m/z calculated for $C_{16}H_{18}FN_2O_2$ [M+H$^+$] = 289.1347 , found = 289.1356.

ethyl (Z)-2-(4-bromo-2-methylspiro[indole-3,3'-pyrrolidin]-2'-ylidene)acetate (23g)

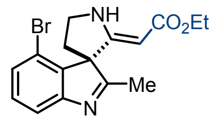

Ethyl (Z)-2-(4-bromo-2-methylspiro[indole-3,3'-pyrrolidin]-2'-ylidene)acetate was prepared according to General procedure C starting from 4-bromo-3-(2-isocyanoethyl)-2-methyl-1H-indole (132.0 mg, 0.5 mmol, 1.0 equiv.). The title compound was isolated as light brown solid (136 mg, 0.39 mmol, 78%). $\mathbf{R_f}$ = 0.30 (EtOAc:cHex = 1:5); **^{1}H NMR** (500 MHz, $CDCl_3$): δ 8.19 (s, 1H), 7.43 (d, J = 7.6 Hz, 1H), 7.28 (d, J = 8.1 Hz, 1H), 7.18 (t, J = 7.8 Hz, 1H), 4.08-3.95 (m, 3H), 3.91 – 3.81 (m, 2H), 2.86 (ddd, J = 13.8, 9.7, 7.5 Hz, 1H), 2.23 (s, 3H), 2.12 (ddd, J = 13.8, 8.4, 3.4 Hz, 1H), 1.15 (t, J = 7.1 Hz, 3H) ppm. **^{13}C{^{1}H} NMR** (126 MHz, $CDCl_3$): δ 183.3 (C_q), 170.8 (C_q), 161.4 (C_q), 157.4 (C_q), 140.1 (C_q), 130.5 (CH), 129.8 (CH), 119.3 (CH), 118.0 (C_q), 76.6 (CH), 69.8 (C_q), 59.0 (CH_2), 46.4 (CH_2), 26.7 (CH_2), 16.2 (CH_3), 14.6 (CH_3) ppm .**HRMS (ESI):** m/z calculated for $C_{16}H_{18}BrN_2O_2$ [M+H$^+$] = 349.0546, found = 349.0555.

ethyl (Z)-2-(5-bromo-2-methylspiro[indole-3,3'-pyrrolidin]-2'-ylidene)acetate (23h)

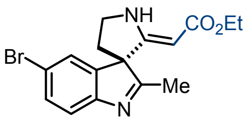

Ethyl (Z)-2-(5-bromo-2-methylspiro[indole-3,3'-pyrrolidin]-2'-ylidene)acetatewas prepared according to General procedure C starting from 5-bromo-3-(2-isocyanoethyl)-2-methyl-1H-indole (132.0 mg, 0.5 mmol, 1.0 equiv.). The title compound was isolated as a yellow solid (124 mg, 0.35 mmol, 71%). $\mathbf{R_f}$ = 0.23 (cyclohexane:EtOAc 2:1); **^{1}H NMR** (500 MHz, $CDCl_3$): δ 8.12 (s, 1H), 7.45 (dd, *J* = 8.2, 1.9 Hz, 1H), 7.37 (d, *J* = 8.3 Hz, 1H), 7.36 (d, *J* = 1.9 Hz, 1H), 4.03 (qd, *J* = 7.1, 1.4 Hz, 2H), 3.93 – 3.79 (m, 2H), 3.88 (s, 1H), 2.40 – 2.27 (m, 2H), 2.26 (s, 3H), 1.17 (t, *J* = 7.1 Hz, 3H) ppm; **^{13}C{^{1}H} NMR** (126 MHz, $CDCl_3$): δ 182.4 (C_q), 170.6 (C_q), 163.1 (C_q), 154.2 (C_q), 144.2 (C_q), 131.9 (CH), 125.6 (CH), 121.6 (CH), 119.7 (C_q), 77.4 (CH), 66.1 (C_q), 59.1 (CH_2), 45.9 (CH_2), 31.5 (CH_2), 16.4 (CH_3), 14.5 (CH_3) ppm; **HRMS (ESI):** m/z calculated for $C_{16}H_{18}BrN_2O_2$ [M+H$^+$] = 349.0546 , found = 349.0553.

ethyl (Z)-2-(7-bromo-2-methylspiro[indole-3,3'-pyrrolidin]-2'-ylidene)acetate (23i)

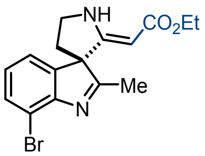

Ethyl (Z)-2-(7-bromo-2-methylspiro[indole-3,3'-pyrrolidin]-2'-ylidene)acetate was prepared according to General procedure C starting from 7-bromo-3-(2-isocyanoethyl)-2-methyl-1H-indole (132.0 mg, 0.5 mmol, 1.0 equiv.). The title compound was isolated as brown solid (110 mg, 0.31 mmol, 63%). $\mathbf{R_f}$ = 0.23 (cyclohexane:EtOAc 2:1); **^{1}H NMR** (500 MHz, $CDCl_3$): δ 8.12 (s, 1H), 7.47 (dd, *J* = 7.9, 1.1 Hz, 1H), 7.16 (dd, *J* = 7.4, 1.0 Hz, 1H), 7.05 (t, *J* = 7.7 Hz, 1H), 4.02 (q, *J* = 7.1 Hz, 2H), 3.93 – 3.81 (m, 3H), 2.43 – 2.25 (m, 5H), 1.16 (t, *J* = 7.1 Hz, 3H) ppm; **^{13}C{^{1}H} NMR** (126 MHz, $CDCl_3$): δ 183.4 (C_q), 170.7 (C_q), 163.2 (C_q), 153.6 (C_q), 143.8 (C_q), 132.2 (CH), 127.6 (CH), 121.2 (CH), 113.9 (C_q), 77.5 (CH), 69.4 (C_q), 59.0 (CH_2), 45.9 (CH_2), 31.6 (CH_2), 16.6 (CH_3), 14.5 (CH_3) ppm; **HRMS (ESI):** m/z calculated for $C_{16}H_{18}BrN_2O_2$ [M+H$^+$] = 349.0546, found = 349.0550.

ethyl (Z)-2-(2-phenylspiro[indole-3,3'-pyrrolidin]-2'-ylidene)acetate (23j)

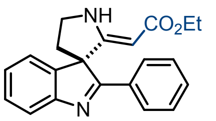

Ethyl (Z)-2-(2-phenylspiro[indole-3,3'-pyrrolidin]-2'-ylidene)acetatewas prepared according to General procedure C starting from 3-(2-isocyanoethyl)-2-phenyl-1H-indole (123.2 mg, 0.5 mmol, 1.0 equiv.). The title compound was isolated as an off-white solid (141 mg, 0.424 mmol, 85%). $\mathbf{R_f}$ = 0.27 (EtOAc : cHex = 1:5); **^{1}H NMR** (500 MHz, $CDCl_3$): δ 8.30 (s, 1H), 7.97 (dd, *J* = 8.0, 1.7 Hz, 2H), 7.71 (d, *J* = 7.8 Hz, 1H), 7.53 – 7.42 (m, 3H), 7.40 (td, *J* = 7.6, 1.3 Hz, 1H), 7.30 (d, *J* = 7.2 Hz, 1H), 7.24 (td, *J* = 7.4, 1.1 Hz, 1H), 4.08 (s, 1H), 4.07 – 3.93 (m, 4H), 2.68 (dt, *J* = 13.1, 9.0 Hz, 1H), 2.19 (ddd, *J* = 13.1, 7.3, 2.7 Hz, 1H), 1.14 (t, *J* = 7.1 Hz, 3H) ppm. **^{13}C{^{1}H} NMR** (126 MHz, $CDCl_3$): δ 178.0 (C_q), 171.0 (C_q), 165.4 (C_q), 154.0 (C_q), 144.5 (C_q), 131.8 (C_q), 131.2 (CH), 128.9 (2 x CH), 128.8 (CH), 126.8 (CH), 121.4 (CH), 121.3 (CH), 77.7 (CH), 66.6 (C_q), 59.0 (CH_2), 46.3 (CH_2), 33.1 (CH_2), 14.5 (CH_3) ppm. **HRMS** (ESI): m/z calculated for $C_{21}H_{21}N_2O_2$ [M+H$^+$] 333.1598, found = 333.1604.

ethyl (Z)-2-(2-(p-tolyl)spiro[indole-3,3'-pyrrolidin]-2'-ylidene)acetate (23k)

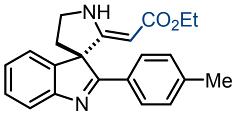

Ethyl (Z)-2-(2-(p-tolyl)spiro[indole-3,3'-pyrrolidin]-2'-ylidene)acetate was prepared according to General procedure C starting from 3-(2-isocyanoethyl)-2-(p-tolyl)-1H-indole (130.7 mg, 0.5 mmol, 1.0 equiv.). The product was purified by flash column chromatography using EtOAc:*c*Hex = (1:4) as eluent to obtain the product as white solid (125 mg, 0.36 mmol, 72%). $\mathbf{R_f}$ = 0.25 (EtOAc: cHex = 1:4); **^{1}H NMR** (500 MHz, $CDCl_3$): δ 8.29 (s, 1H), 7.88 (d, *J* = 8.1 Hz, 2H), 7.70 (d, *J* = 7.7 Hz, 1H), 7.38 (t, *J* = 7.6 Hz, 1H), 7.29 (d, *J* = 7.2 Hz, 1H), 7.26 (d, *J* = 8.0 Hz, 2H), 7.22 (t, *J* = 7.3 Hz, 1H), 4.07 (s, 1H), 4.05 – 3.94 (m, 4H), 2.67 (dt, *J* = 13.2, 9.0 Hz, 1H), 2.40 (s, 3H), 2.22 – 2.12 (m, 1H), 1.13 (t, *J* = 7.2 Hz, 3H) ppm; **^{13}C{^{1}H} NMR** (126 MHz, $CDCl_3$): δ 178.0 (C_q), 171.0 (C_q), 165.5 (C_q), 154.1 (C_q), 144.5 (C_q), 141.7 (C_q), 129.6 (CH), 129.0 (C_q), 128.8 (CH), 128.7 (CH), 126.5 (CH), 121.2 (CH), 121.1 (CH), 77.6 (CH), 66.5 (C_q), 58.9 (CH_2), 46.2 (CH_2), 33.3 (CH_2), 21.7 (CH_3), 14.5 (CH_3) ppm.; **HRMS (ESI):** m/z calculated for $C_{22}H_{23}N_2O_2$ [M+H$^+$] = 347.1754, found = 347.1758.

ethyl (Z)-2-(2-(4-fluorophenyl)spiro[indole-3,3'-pyrrolidin]-2'-ylidene)acetate (23l)

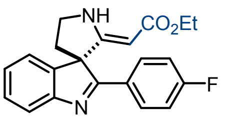

Ethyl (Z)-2-(2-(4-fluorophenyl)spiro[indole-3,3'-pyrrolidin]-2'-ylidene)acetate was prepared according to General procedure C starting from 2-(4-fluorophenyl)-3-(2-isocyanoethyl)-1H-indole (132.3 mg, 0.5 mmol, 1.0 equiv.). The title compound was isolated as a light-yellow solid (125 mg, 0.36 mmol, 72%). $\mathbf{R_f}$ = 0.25 (EtOAc: cHex = 1:4); **^{1}H NMR** (500 MHz, $CDCl_3$): δ 8.29 (s, 1H), 7.98 (dd, *J* = 8.8, 5.4 Hz, 2H), 7.69 (d, *J* = 7.7 Hz, 1H), 7.39 (td, *J* = 7.7, 1.0 Hz, 1H), 7.29 (d, *J* = 7.1 Hz, 1H), 7.23 (t, *J* = 7.4 Hz, 1H), 7.13 (t, *J* = 8.6 Hz, 2H), 4.06 (s, 1H), 4.05 – 3.93 (m, 4H), 2.62 (dt, *J* = 13.2, 9.0 Hz, 1H), 2.22 – 2.13 (m, 1H), 1.14 (t, *J* = 7.1 Hz, 3H) ppm; **^{13}C{^{1}H} NMR** (126 MHz, $CDCl_3$): δ 176.8 (C_q), 170.9 (C_q), 165.1 (C_q), 164.8 (d, *J* = 253.2 Hz, C_q), 153.8 (C_q), 144.4 (C_q), 130.9 (d, *J* = 8.6 Hz, CH), 128.9 (CH), 128.0 (C_q, d, *J* = 3.3 Hz), 126.7 (CH), 121.3 (2 x CH), 116.0 (d, *J* = 21.8 Hz, CH), 77.8 (CH), 66.5 (C_q), 59.0 (CH_2), 46.2 (CH_2), 33.2 (CH_2), 14.5 (CH_3) ppm; **^{19}F{^{1}H} NMR** (470 MHz, $CDCl_3$): δ -108.27 ppm; **HRMS (ESI):** m/z calculated for $C_{21}H_{20}FN_2O_2$ [M+H$^+$] = 351.1503, found = 351.1515.

ethyl (Z)-2-(2-(4-chlorophenyl)spiro[indole-3,3'-pyrrolidin]-2'-ylidene)acetate (23m)

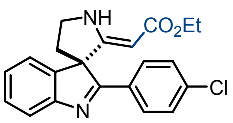

ethyl (Z)-2-(2-(4-chlorophenyl)spiro[indole-3,3'-pyrrolidin]-2'-ylidene)acetate was prepared according to General procedure C starting from 2-(4-chlorophenyl)-3-(2-isocyanoethyl)-1H-indole (140.3 mg, 0.5 mmol, 1.0 equiv.). The title compound was isolated as a light-yellow solid (138 mg, 0.38 mmol, 76%). $\mathbf{R_f}$ = 0.21 (EtOAc: cHex = 1:4); **^{1}H NMR** (500 MHz, $CDCl_3$): δ 8.28 (s, 1H), 7.92 (d, *J* = 8.6 Hz, 2H), 7.9 (d, *J* = 7.7 Hz, 1H), 7.46-7.37 (m, 3H), 7.30 (d, *J* = 7.1 Hz, 1H), 7.24 (t, *J* = 7.3 Hz, 1H), 4.05 (s, 1H), 4.06 – 3.93 (m, 4H), 2.62 (dt, *J* = 13.0, 8.9 Hz, 1H), 2.17 (dd, *J* = 12.9, 6.1 Hz, 2H)1.14 (t, *J* = 7.1 Hz, 1H) ppm; **^{13}C{^{1}H} NMR** (126 MHz, $CDCl_3$): δ 176.8 (C_q), 170.9 (C_q), 164.9 (C_q), 153.8 (C_q), 144.5 (C_q), 137.4 (C_q), 130.1 (C_q), 130.0 (CH), 129.2 (CH), 129.0 (CH), 126.9 (CH) 121.4 (CH), 121.3 (CH), 77.8 (CH), 66.4 (C_q), 59.0 (CH_2), 46.2 (CH_2), 33.1 (CH_2), 14.5 (CH_3) ppm. **HRMS (ESI):** m/z calculated for $C_{21}H_{20}ClN_2O_2$ [M+H$^+$] = 367.1208, found = 367.1215.

ethyl (Z)-2-(2-(naphthalen-2-yl)spiro[indole-3,3'-pyrrolidin]-2'-ylidene)acetate (23n)

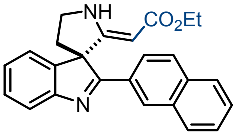

Ethyl (Z)-2-(2-(naphthalen-2-yl)spiro[indole-3,3'-pyrrolidin]-2'-ylidene)acetate was prepared according to General procedure C starting from 3-(2-isocyanoethyl)-2-(naphthalen-2-yl)-1H-indole (148.1 mg, 0.5 mmol, 1.0 equiv.). The product was purified by flash column chromatography using EtOAc:cHex = 1:4 as eluent to obtain the product as white solid (115 mg, 0.30 mmol, 60%). $\mathbf{R_f}$ = 0.30 (1% Et_3N in EtOAc:cHex = 1:4); **^{1}H NMR** (500 MHz, $CDCl_3$) δ (ppm): 8.44-8.30 (m, 2H), 8.18 (dd, *J* = 8.7, 1.8 Hz, 1H), 7.95 – 7.89 (m, 2H), 7.87 (d, *J* = 7.9, 1H), 7.75 (d, *J* = 7.8 Hz, 1H), 7.59 – 7.50 (m, 2H), 7.45 (td, *J* = 7.5, 1.3 Hz, 1H), 7.34 (dd, *J* = 7.5, 1.2 Hz, 1H), 7.26 (td, *J* = 7.4, 1.0 Hz, 1H), 4.12 (s, 1H), 4.11 – 3.94 (m, 4H), 2.78 (dt, *J* = 13.2, 9.0 Hz, 1H), 2.24 (ddd, *J* = 13.1, 6.7, 3.1 Hz, 1H), 1.12 (t, *J* = 7.2 Hz, 3H) ppm; **^{13}C{^{1}H} NMR** (126 MHz, $CDCl_3$): δ 177.9 (C_q), 170.9 (C_q), 165.4 (C_q), 154.0 (C_q), 144.7 (C_q), 134.6 (C_q), 133.0 (C_q), 129.3 (CH), 129.3 (CH), 129.2 (C_q), 128.9 (CH), 128.6 (CH), 127.8 (CH), 127.8 (CH), 126.8 (CH), 126.6 (CH), 125.3 (CH), 121.4 (CH), 121.3 (CH), 77.8 (CH), 66.6 (CH_q), 58.9 (CH_2), 46.3 (CH_2), 33.4 (CH_2), 14.5 (CH_3) ppm. **HRMS (ESI):** m/z calculated for $C_{25}H_{22}N_2O_2$ [M+H$^+$] = 383.1754, found = 383.1750.

ethyl (Z)-2-(2-bromospiro[indole-3,3'-pyrrolidin]-2'-ylidene)acetate (23o)

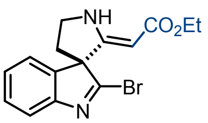

Ethyl (Z)-2-(2-bromospiro[indole-3,3'-pyrrolidin]-2'-ylidene)acetatewas prepared according to General procedure C starting from 2-bromo-3-(2-isocyanoethyl)-1H-indole (129.9 mg, 0.52 mmol, 1.0 equiv.). The title compound was isolated as a light-yellow solid (136 mg, 0.40 mmol, 78%). $\mathbf{R_f}$ = 0.43 (EtOAc: cHex = 1:3); **^{1}H NMR** (500 MHz, $CDCl_3$): δ 8.12 (s, 1H), 7.55 (d, *J* = 7.7 Hz, 1H), 7.35 (td, *J* = 7.3, 2.0 Hz, 1H), 7.30 – 7.22 (m, 2H), 4.10-3.94 (m, 5H), 2.53 (ddd, *J* = 13.1, 7.9, 5.0 Hz, 1H), 2.34 (ddd, *J* = 13.7, 8.1, 6.2 Hz, 1H), 1.17 (t, *J* = 7.2 Hz, 3H) ppm; **^{13}C{^{1}H} NMR** (126 MHz, $CDCl_3$): δ 170.6 (C_q), 164.1 (C_q), 161.9 (C_q), 154.0 (C_q), 141.8 (C_q), 129.2 (CH), 127.2 (CH) 122.3 (CH), 120.7 (CH), 78.0 (CH), 71.0 (C_q), 59.1 (CH_2), 45.9 (CH_2), 31.9 (CH_2), 14.5 (CH_3) ppm. **HRMS (ESI):** m/z calculated for $C_{15}H_{16}BrN_2O_2$ [M+H$^+$] = 335.0390, found = 335.0388.

ethyl (Z)-2-((Z)-2-(2-methoxy-2-oxoethylidene)spiro[indoline-3,3'-pyrrolidin]-2'-ylidene)acetate (23p)

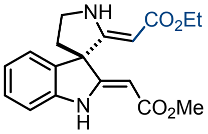

Ethyl (Z)-2-((Z)-2-(2-methoxy-2-oxoethylidene)spiro[indoline-3,3'-pyrrolidin]-2'-ylidene)acetate was prepared according to General procedure C starting from methyl 2-(3-(2-isocyanoethyl)-1H-indol-2-yl)acetate (121.6 mg, 0.5 mmol, 1.0 equiv.). The title compound was isolated as a white solid (102 mg, 0.31 mmol, 62%). $\mathbf{R_f}$ = 0.28 (EtOAc: cHex = 1:4); **^{1}H NMR** (500 MHz, $CDCl_3$): δ 9.66 (s, 1H), 8.06 (s, 1H), 7.20 (t, *J* = 7.7 Hz, 1H), 7.12 (d, *J* = 7.4 Hz, 1H), 6.92 (t, *J* = 7.4 Hz, 1H), 6.85 (d, *J* = 7.8 Hz, 1H), 4.88 (s, 1H), 4.11 (s, 1H), 4.04 (qd, *J* = 7.1, 1.1 Hz, 2H), 3.82 (t, *J* = 6.8 Hz, 2H), 3.70 (s, 3H), 2.46 – 2.30 (m, 2H), 1.18 (t, *J* = 7.1 Hz, 3H) ppm; **^{13}C{^{1}H} NMR** (126 MHz, $CDCl_3$): δ 171.0 (C_q), 170.5 (C_q), 167.2 (C_q), 167.0 (C_q), 143.8 (C_q), 132.6 (C_q), 129.2 (CH), 123.4 (CH), 122.0 (CH), 109.4 (CH), 82.3 (CH), 78.8 (CH), 61.1 (C_q), 59.0 (CH_2), 50.9 (CH_3), 45.4 (CH_2), 38.5 (CH_2), 14.6 (CH_3) ppm. **HRMS (ESI):** m/z calculated for $C_{18}H_{21}N_2O_4$ [M+H$^+$] = 329.1496 , found = 329.1497.

5.2 General procedure D: Carbene transfer spirocyclisation cascade and one-pot reduction towards spiroindolines

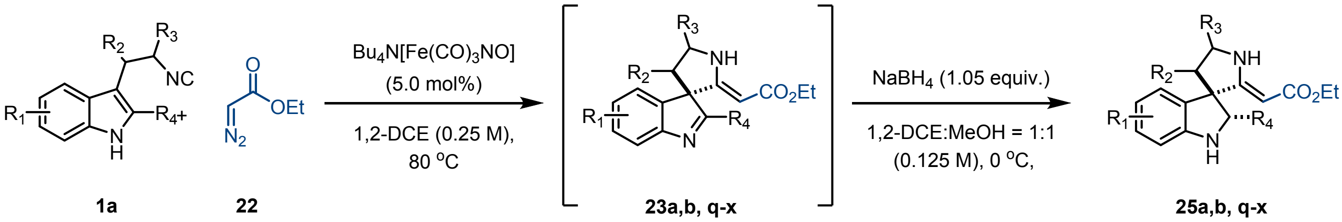

To a flame dried Schlenk flask under N_2 atmosphere, charged with a stirring bean, was added $Bu_4N[Fe(CO)_3NO]$ (0.05 equiv.). Subsequently, 1,2-DCE was added (0.25 M), and the mixture was stirred until the catalyst was dissolved. This was followed by the addition of tryptamine-derived isocyanide (0.5 mmol, 1.0 equiv.) and ethyl diazoacetate (**22**) (0.6 mmol, 1.2 equiv.). The solution was placed in a pre-heated oil bath and stirred at 80 °C until full conversion of the isocyanide was observed on TLC. Subsequently, the reaction mixture was cooled to 0 °C and diluted with MeOH to a concentration of 0.125 M, after which $NaBH_4$ (1.05 equiv.) was added. The reaction was stirred at 0 °C until full conversion of indolenine intermediate **23** was observed on TLC. Afterwards, the reaction mixture was quenched with saturated aqueous NH_4Cl solution and stirred vigorously for 15 minutes. The aqueous layer was extracted with CH_2Cl_2 (3x) and the organic layers were collected, washed with brine, dried over Na_2SO_4, and filtered. The filtrate was collected and concentrated *in vacuo*. Subsequently, the crude product was subjected to flash column chromatography, using a mixture of EtOAc:*c*Hex as eluent, to obtain the pure title compound.

ethyl (Z)-2-(spiro[indoline-3,3'-pyrrolidin]-2'-ylidene)acetate (25a)

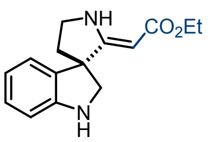

Ethyl (Z)-2-(spiro[indoline-3,3'-pyrrolidin]-2'-ylidene)acetate was prepared according to General procedure D starting from 3-(2-isocyanoethyl)-1H-indole (85.2 mg, 0.5 mmol, 1.0 equiv.), ethyl diazoacetate (0.6 mmol, 1.2 equiv.), $Bu_4N[Fe(CO)_3NO]$ (10.3 mg, 0.025 mmol, 0.05 equiv.) and $NaBH_4$ (20 mg, 0.53 mmol). The title compound was isolated as a yellow solid (99 mg, 0.38 mmol, 77%). **R_f** = 0.25 (EtOAc:cHex 1:2); **^{1}H NMR** (500 MHz, $CDCl_3$) δ (ppm): 7.96 (s, 1H), 7.08 (td, J = 7.6, 1.3 Hz, 1H), 7.01 (dd, J = 7.4, 1.3 Hz, 1H), 6.74 (td, J = 7.4, 1.0 Hz, 1H), 6.68 (d, J = 7.9, 1H), 4.44 (s, 1H), 4.07 (qd, J = 7.1, 3.1 Hz, 2H), 3.79 (br, 1H), 3.72 – 3.48 (m, 4H), 2.29 – 2.12 (m, 2H), 1.21 (t, J = 7.1 Hz, 3H) ppm; **^{13}C{^{1}H} NMR** (126 MHz, $CDCl_3$): δ 171.3 (C_q), 170.7 (C_q), 151.2 (C_q), 132.5 (C_q), 128.6 (CH), 123.7 (CH), 119.5 (CH), 110.1 (CH), 77.5 (CH), 59.4 (CH_2), 58.7 (CH_2), 57.3 (C_q), 45.0 (CH_2), 37.2 (CH_2), 14.7 (CH_3) ppm; **HRMS (ESI):** m/z calculated for $C_{15}H_{19}N_2O_2$ [M+H$^+$] = 259.1441, 259.1441.

ethyl (Z)-2-(2-methylspiro[indoline-3,3'-pyrrolidin]-2'-ylidene)acetate (25b)

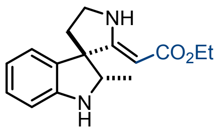

To a flame dried Schlenk flask under N_2 atmosphere, charged with a stirring bean, was added $Bu_4N[Fe(CO)_3NO]$ (10.3 mg, 0.025 mmol, 0.05 equiv.). Subsequently, 1,2-DCE was added (0.25 M), and the mixture was stirred until the catalyst was dissolved. This was followed by the addition of 3-(2-isocyanoethyl)-2-methyl-1H-indole (92.4 mg, 0.5 mmol, 1.0 equiv.), ethyl diazoacetate (0.6 mmol, 1.2 equiv.). The solution was placed in a pre-heated oil bath and stirred at 80 °C until full conversion of the isocyanide was observed on TLC. Subsequently, the reaction mixture was cooled to 0 °C and diluted with MeOH to a concentration of 0.125 M, after which $NaBH_3CN$ (32 mg, 0.51 mmol, 1.02 equiv.) and a few drops of AcOH were added. The resulting mixture was stirred at 0 °C until full conversion of the spiroindolenine intermediate was observed on TLC. Subsequently, the mixture was neutralized with Na_2CO_3 and diluted with CH_2Cl_2. The aqueous layer was extracted with CH_2Cl_2 (3 x). The combined organic layers were washed with brine, dried over Na_2SO_4, filtered, and concentrated *in vacuo*. This was followed by purification via FCC using a gradient of *c*Hex:EtOAc to obtain the title compound as a light-yellow solid (99 mg, 0.36 mmol, 73%). **R_f** = 0.29 (cHex:EtOAc = 2:1); **^{1}H NMR** (500 MHz, $CDCl_3$): δ 7.96 (s, 1H), 7.08 (td, *J* = 7.6, 1.3 Hz, 1H), 7.03 (dd, *J* = 7.4, 1.2 Hz, 1H), 6.76 (td, *J* = 7.4, 0.8 Hz, 1H), 6.66 (d, *J* = 7.7 Hz, 1H), 4.25 (s, 1H), 4.04 (q, *J* = 7.1 Hz, 2H), 3.86 (q, *J* = 6.5 Hz, 1H), 3.65 – 3.51 (m, 2H), 2.53-2.41 (m, 1H), 2.13 (ddd, *J* = 13.0, 6.6, 2.2 Hz, 1H).zf), 1.23 (d, *J* = 6.4 Hz, 3H), 1.20 (t, *J* = 7.1 Hz, 3H) ppm; **^{13}C{^{1}H} NMR** (126 MHz, $CDCl_3$): δ 171.1 (C_q), 166.6 (C_q), 151.0 (C_q), 132.3 (C_q), 128.6 (CH), 124.0 (CH), 119.6 (CH), 110.1 (CH), 79.5 (CH), 65.5 (CH), 60.0 (C_q), 58.6 (CH_2), 44.7 (CH_2), 36.8 (CH_2), 17.1 (CH_3), 14.6 (CH_3) ppm; **HRMS (ESI):** m/z calculated for $C_{16}H_{21}N_2O_2$ [M+H$^+$] = 273.1598, found = 273.1603.

ethyl (Z)-2-(5-methoxyspiro[indoline-3,3'-pyrrolidin]-2'-ylidene)acetate (25q)

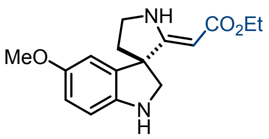

Ethyl (Z)-2-(5-methoxyspiro[indoline-3,3'-pyrrolidin]-2'-ylidene)acetate was prepared according to General procedure D starting from 3-(2-isocyanoethyl)-5-methoxy-1H-indole (100.2 mg, 0.5 mmol, 1.0 equiv.), ethyl diazoacetate (0.6 mmol, 1.2 equiv.), $Bu_4N[Fe(CO)_3NO]$ (10.3 mg, 0.025 mmol, 0.05 equiv.) and $NaBH_4$ (20 mg, 0.53 mmol). The title compound was isolated as a light-yellow solid (81 mg, 0.28 mmol, 56%). **Rf**: indoline = 0.16 (*c*Hex:EtOAc = 6:4); **^{1}H NMR** (500 MHz, $CDCl_3$): δ 7.94 (s, 1H), 6.69-6.59 (m, 3H), 4.43 (s, 1H), 4.07 (qd, *J* = 7.1, 1.2 Hz, 2H), 3.72 (s, 3H), 3.68 – 3.48 (m, 4H), 3.26 (br, 1H), 2.29 – 2.11 (m, 2H), 1.21 (t, *J* = 7.1 Hz, 3H) ppm; **^{13}C{^{1}H} NMR** (126 MHz, $CDCl_3$): δ 171.3 (C_q), 170.4 (C_q), 154.3 (C_q), 144.9 (C_q), 134.2 (C_q), 114.1 (CH), 111.2 (CH), 110.1 (CH), 77.7 (CH), 59.9 (CH_2), 58.8 (CH_2), 58.0 (C_q), 56.0 (CH_3), 45.0 (CH_2), 36.9 (CH_2), 14.7 (CH_3) ppm; **HRMS (ESI):** m/z calculated for $C_{16}H_{21}N_2O_3$ [M+H$^+$] = 289.1547, found = 289.1553.

ethyl (Z)-2-(6-methoxyspiro[indoline-3,3'-pyrrolidin]-2'-ylidene)acetate (25r)

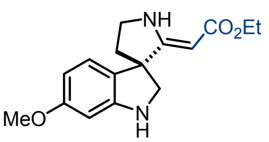

Ethyl (Z)-2-(6-methoxyspiro[indoline-3,3'-pyrrolidin]-2'-ylidene)acetate was prepared according to General procedure D starting from 3-(2-isocyanoethyl)-6-methoxy-1H-indole (100.1 mg, 0.5 mmol, 1.0 equiv.), ethyl diazoacetate (0.6 mmol, 1.2 equiv.), $Bu_4N[Fe(CO)_3NO]$ (10.3 mg, 0.025 mmol, 0.05 equiv.) and $NaBH_4$ (20 mg, 0.53 mmol). Extra portions of $NaBH_4$ were added over time until full conversion of the indolenine intermediate was observed. The title compound was isolated as a light-yellow solid (97 mg, 0.34 mmol, 67%). $\mathbf{R_f}$ = 0.24 (*c*Hex:EtOAc = 6:4); **^{1}H NMR** (500 MHz, $CDCl_3$): δ 7.93 (s, 1H), 6.89 (d, *J* = 8.1 Hz, 1H), 6.29 (dd, *J* = 8.2, 2.3 Hz, 1H), 6.25 (d, *J* = 2.3 Hz, 1H), 4.43 (s, 1H), 4.07 (qd, *J* = 7.2, 2.2 Hz, 2H), 3.78 (s, 1H), 3.75 (s, 3H), 3.67-3.50 (m, 4H), 2.26 – 2.10 (m, 2H), 1.21 (t, *J* = 7.1 Hz, 3H) ppm; **^{13}C{^{1}H} NMR** (126 MHz, $CDCl_3$): δ 171.4 (C_q), 171.0 (C_q), 160.9 (C_q), 152.6 (C_q), 124.9 (C_q), 124.2 (CH), 104.7 (CH), 96.5 (CH), 77.3 (CH), 59.9 (CH_2), 58.8 (CH_2), 56.7 (C_q), 55.5 (CH_3), 45.0 (CH_2), 37.3 (CH_2), 14.7 (CH_3) ppm. **HRMS (ESI):** m/z calculated for $C_{16}H_{21}N_2O_3$ [M+H$^+$] = 289.1547, found = 289.1554.

ethyl (Z)-2-(5-methylspiro[indoline-3,3'-pyrrolidin]-2'-ylidene)acetate (25s)

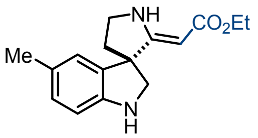

Ethyl (Z)-2-(5-methylspiro[indoline-3,3'-pyrrolidin]-2'-ylidene)acetate was prepared according to General procedure D starting from 3-(2-isocyanoethyl)-5-methyl-1H-indole (92.2 mg, 0.5 mmol, 1.0 equiv.), ethyl diazoacetate (0.6 mmol, 1.2 equiv.), $Bu_4N[Fe(CO)_3NO]$ (10.3 mg, 0.025 mmol, 0.05 equiv.) and $NaBH_4$ (20 mg, 0.53 mmol). Extra portions of $NaBH_4$ were added over time until full conversion of the indolenine intermediate was observed. The title compound was isolated as a white solid (100 mg, 0.37 mmol, 74%). $\mathbf{R_f}$ = 0.64 (*c*Hex:EtOAc = 1:1); **^{1}H NMR** (500 MHz, $CDCl_3$): δ 7.97 (s, 1H), 6.89 (d, *J* = 7.9 Hz, 1H), 6.83 (s, 1H), 6.61 (d, *J* = 7.9 Hz, 1H), 4.44 (s, 1H), 4.08 (qd, *J* = 7.1, 4.0 Hz, 2H), 3.72 – 3.50 (m, 4H), 3.47 (br, 1H), 2.27 – 2.12 (m, 5H), 1.22 (t, *J* = 7.1 Hz, 3H) ppm; **^{13}C{^{1}H} NMR** (126 MHz, $CDCl_3$): δ 171.4 (C_q), 170.8 (C_q), 148.7 (C_q), 132.9 (C_q), 129.2 (C_q), 129.1 (CH), 124.3 (CH), 110.3 (CH), 77.5 (CH), 59.6 (CH_2), 58.7 (CH_2), 57.5 (C_q), 45.0 (CH_2), 37.1 (CH_2), 21.0 (CH_3), 14.7 (CH_3) ppm; **HRMS (ESI):** m/z calculated for $C_{16}H_{21}N_2O_2$ [M+H$^+$] = 273.1598, found = 273.1597.

ethyl (Z)-2-(5-fluorospiro[indoline-3,3'-pyrrolidin]-2'-ylidene)acetate (25t)

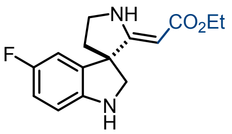

Ethyl (Z)-2-(5-fluorospiro[indoline-3,3'-pyrrolidin]-2'-ylidene)acetate was prepared according to General procedure D starting from 5-fluoro-3-(2-isocyanoethyl)-1H-indole (94.4 mg, 0.5 mmol, 1.0 equiv.), ethyl diazoacetate (0.6 mmol, 1.2 equiv.), $Bu_4N[Fe(CO)_3NO]$ (10.3 mg, 0.025 mmol, 0.05 equiv.) and $NaBH_4$ (20 mg, 0.53 mmol). The title compound was isolated as a light-brown solid (110 mg, 0.70 mmol, 79%). **$\mathbf{R_f}$ = 0.25** (*c*Hex:EtOAc = 2:1); **^{1}H NMR** (500 MHz, $CDCl_3$): δ 7.94 (s, 1H), 6.78 (td, *J* = 8.8, 2.7 Hz, 1H), 6.73 (dd, *J* = 8.3, 2.6 Hz, 1H), 6.60 (dd, *J* = 8.5, 4.3 Hz, 1H), 4.42 (s, 1H), 4.08 (q, *J* = 7.0 Hz, 2H), 3.68 – 3.52 (m, 4H), 3.31 (br, 1H), 2.31 – 2.10 (m, 2H), 1.22 (t, *J* = 7.2 Hz, 3H) ppm; **^{13}C{^{1}H} NMR** (126 MHz, $CDCl_3$): δ 171.3 (C_q), 169.9 (C_q), 157.5 (C_q, d, *J* = 236.6 Hz), 147.1 (C_q, d, *J* = 1.6 Hz), 134.2 (C_q, d, *J* = 7.7 Hz), 115.0 (CH, d, *J* = 23.5 Hz), 111.1 (CH, d, *J* = 24.2 Hz), 110.7 (CH, d, *J* = 8.2 Hz), 77.8 (CH), 59.9 (CH_2), 58.9 (CH_2), 57.7 (C_q), 45.0 (CH_2), 37.0 (CH_2), 14.7 (CH_3) ppm; **^{19}F{^{1}H} NMR** (470.4 MHz, $CDCl_3$): δ -124.9 ppm; **HRMS (ESI):** m/z calculated for $C_{15}H_{18}FN_2O_2$ [M+H$^+$] = 277.1347, found = 277.1346.

ethyl (Z)-2-(5-chlorospiro[indoline-3,3'-pyrrolidin]-2'-ylidene)acetate (25u)

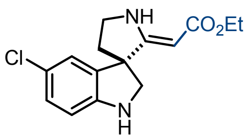

Ethyl (Z)-2-(5-chlorospiro[indoline-3,3'-pyrrolidin]-2'-ylidene)acetate was prepared according to General procedure D starting from 5-chloro-3-(2-isocyanoethyl)-1H-indole (102.8 mg, 0.5 mmol, 1.0 equiv.), ethyl diazoacetate (0.6 mmol, 1.2 equiv.), $Bu_4N[Fe(CO)_3NO]$ (10.3 mg, 0.025 mmol, 0.05 equiv.) and $NaBH_4$ (20 mg, 0.53 mmol). The title compound was isolated as a white solid (96 mg, 0.33 mmol, 66%). $\mathbf{R_f}$ = 0.48 (*c*Hex:EtOAc = 1:1); **^{1}H NMR** (500 MHz, $CDCl_3$): δ 7.94 (s, 1H), 7.02 (dd, *J* = 8.4, 2.1 Hz, 1H), 6.95 (d, *J* = 2.1 Hz, 1H), 6.57 (d, *J* = 8.3 Hz, 1H), 4.42 (s, 1H), 4.08 (q, *J* = 7.1 Hz, 2H), 3.79 (br, 1H), 3.69 – 3.50 (m, 4H), 2.28 – 2.09 (m, 2H), 1.22 (t, *J* = 7.1 Hz, 3H) ppm; **^{13}C{^{1}H} NMR** (126 MHz, $CDCl_3$): δ 171.2 (C_q), 169.9 (C_q), 149.8 (C_q), 134.3 (C_q), 128.5 (CH), 124.0 (CH), 123.9 (C_q), 110.8 (CH), 77.8 (CH), 59.7 (CH_2), 58.9 (CH_2), 57.3 (C_q), 45.0 (CH_2), 37.2 (CH_2), 14.7 (CH_3) ppm**. HRMS (ESI):** m/z calculated for $C_{15}H_{18}ClN_2O_2$ [M+H$^+$] = 293.1051, found = 293.1058.

ethyl (Z)-2-(5-bromospiro[indoline-3,3'-pyrrolidin]-2'-ylidene)acetate (25v)

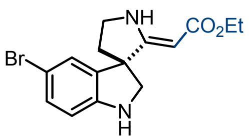

Ethyl (Z)-2-(5-bromospiro[indoline-3,3'-pyrrolidin]-2'-ylidene)acetate was prepared according to General procedure D starting from 5-bromo-3-(2-isocyanoethyl)-1H-indole (124.4 mg, 0.5 mmol, 1.0 equiv.), ethyl diazoacetate (0.6 mmol, 1.2 equiv.), $Bu_4N[Fe(CO)_3NO]$ (10.3 mg, 0.025 mmol, 0.05 equiv.) and $NaBH_4$ (20 mg, 0.53 mmol). The title compound was isolated as a light-brown solid (102 mg, 0.30 mmol, 61%). $\mathbf{R_f}$ = 0.27 (*c*Hex:EtOAc = 2:1); **^{1}H NMR** (500 MHz, $CDCl_3$): δ 7.94 (s, 1H), 7.15 (dd, *J* = 8.3, 2.0 Hz, 1H), 7.08 (d, *J* = 2.1 Hz, 1H), 6.54 (d, *J* = 8.3 Hz, 1H), 4.42 (s, 1H), 4.08 (q, *J* = 7.1 Hz, 2H), 3.80 (br, 1H), 3.68 – 3.50 (m, 4H), 2.24 (ddd, *J* = 12.8, 7.1, 4.1 Hz, 1H), 2.14 (dt, *J* = 12.7, 7.7 Hz, 1H), 1.23 (t, *J* = 7.1 Hz, 3H) ppm; **^{13}C{^{1}H} NMR** (126 MHz, $CDCl_3$): δ 171.2 (C_q), 169.9 (C_q), 150.2 (C_q), 134.8 (C_q), 133.4 (CH), 126.8 (CH), 111.4 (CH), 110.8 (C_q), 77.8 (CH), 59.6 (CH_2), 58.9 (CH_2), 57.3 (C_q), 45.0 (CH_2), 37.2 (CH_2), 14.7 (CH_3) ppm; **HRMS (ESI):** m/z calculated for $C_{15}H_{18}BrN_2O_2$ [M+H$^+$] = 337.0546, found = 337.0552.

methyl (3S,Z)-2'-(2-ethoxy-2-oxoethylidene)spiro[indoline-3,3'-pyrrolidine]-5'-carboxylate (25w)

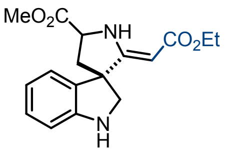

To a flame dried Schlenk flask under N_2 atmosphere, charged with a stirring bean, was added $Bu_4N[Fe(CO)_3NO]$ (10.3 mg, 0.025 mmol, 0.05 equiv.). Subsequently, 1,2-DCE was added (0.25 M), and the mixture was stirred until the catalyst was dissolved. This was followed by the addition of 3-(1*H*-indol-3-yl)-2-isocyanopropanoate (114, mg, 0.50 mmol, 1.0 equiv.) and ethyl diazoacetate (0.6 mmol, 1.2 equiv.). The solution was placed in a pre-heated oil bath and stirred at 80 °C until full conversion of the isocyanide was observed on TLC. Subsequently, the reaction mixture was cooled to 0 °C and diluted with MeOH to a concentration of 0.125 M, after which $NaBH_3CN$ (33 mg, 0.53 mmol, 1.05 equiv.) and a few drops of AcOH were added. The resulting mixture was stirred at 0 °C until full conversion of the spiroindolenine intermediate was observed on TLC. Subsequently, the mixture was neutralized with Na_2CO_3 and diluted with CH_2Cl_2. The aqueous layer was extracted with CH_2Cl_2 (3 x). The combined organic layers were washed with brine, dried over Na_2SO_4, filtered, and concentrated *in vacuo*. This was followed by purification via FCC using *c*Hex:EtOAc = 6:4 as eluent to obtain the title compound as two diastereomers separately. (*combined yield:* 86 mg, 0.25 mmol, 50%, *d.r.* = 2.2:1). *d.r.* determined via ^{1}H-NMR of crude product mixture.

D1 (major): yellow oil (62 mg, 0.18 mmol, 36%); $\mathbf{R_f}$ = 0.60 (*c*Hex:EtOAc = 6:4); **^{1}H NMR** (500 MHz, $CDCl_3$): δ 8.23 (s, 1H), 7.09 (td, *J* = 7.7, 1.1 Hz, 1H), 6.99 (dd, *J* = 7.4, 1.0 Hz, 1H), 6.75 (td, *J* = 7.5, 0.9 Hz, 1H), 6.68 (d, *J* = 7.8 Hz, 1H), 4.52 (dd, *J* = 8.7, 3.8 Hz, 1H), 4.49 (s, 1H), 4.08 (qd, *J* = 7.1, 2.2 Hz, 2H), 3.78 (s, 3H), 3.71 (d, J = 9.6 Hz, 1H), 3.52 (d, J = 9.5 Hz, 1H), 2.58 – 2.41 (m, 2H), 1.21 (t, *J* = 7.1 Hz, 3H) ppm; **^{13}C{^{1}H} NMR** (126 MHz, $CDCl_3$): δ 172.7 (C_q), 171.0 (C_q), 169.6 (C_q), 151.1 (C_q), 132.3 (C_q), 128.9 (CH), 123.5 (CH), 119.6 (CH), 110.3 (CH), 79.7 (CH), 60.5 (CH_2), 59.0 (CH_2), 58.7 (CH), 56.6 (C_q), 52.7 (CH_3), 40.6 (CH_2), 14.6 (CH_3) ppm. **HRMS (ESI):** m/z calculated for $C_{17}H_{21}N_2O_4$ [M+H$^+$] = 317.1496, found = 317.1497.

D2 (minor): yellow oil (24 mg, 0.07 mmol, 14%); $\mathbf{R_f}$ = 0.29 (*c*Hex:EtOAc = 6:4); **^{1}H NMR** (500 MHz, $CDCl_3$): δ 8.17 (s, 1H), 7.09 (td, *J* = 7.7, 1.2 Hz, 1H), 7.03 (d, *J* = 7.5 Hz, 1H), 6.76 (td, *J* = 7.5, 0.8 Hz, 1H), 6.69 (d, *J* = 7.8 Hz, 1H), 4.48 – 4.41 (m, 2H), 4.09 (qd, *J* = 7.2, 2.3 Hz, 2H), 3.79 (s, 3H), 3.60 (dd, *J* = 15.9, 9.2 Hz, 2H), 2.62 (dd, *J* = 13.0, 7.0 Hz, 1H), 2.23 (dd, *J* = 13.0, 9.0 Hz, 1H), 1.22 (t, *J* = 7.1 Hz, 3H) ppm; **^{13}C{^{1}H} NMR** (126 MHz, $CDCl_3$): δ 171.8 (C_q), 170.9 (C_q), 169.0 (C_q), 151.3 (C_q), 131.2 (C_q), 129.0 (CH), 124.2 (CH), 120.0 (CH), 110.3 (CH), 79.9 (CH), 60.3 (CH_2), 59.0 (CH_2), 58.5 (CH), 57.4 (C_q), 52.7 (CH_3), 40.7 (CH_2), 14.6 (CH_3) ppm. **HRMS (ESI):** m/z calculated for $C_{17}H_{21}N_2O_4$ [M+H$^+$] = 317.1501, found = 317.1497.

ethyl (Z)-2-((3S,4'R)-4'-(3-methoxyphenyl)spiro[indoline-3,3'-pyrrolidin]-2'-ylidene)acetate (25x)

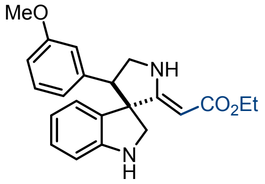

To a flame dried Schlenk flask under N_2 atmosphere, charged with a stirring bean, was added $Bu_4N[Fe(CO)_3NO]$ (10.3 mg, 0.025 mmol, 0.06 equiv.). Subsequently, 1,2-DCE was added (0.25 M), and the mixture was stirred until the catalyst was dissolved. This was followed by the addition of 3-(2-isocyano-1-(3-methoxyphenyl)ethyl)-1H-indole (112 mg, 0.41 mmol, 1.0 equiv.) and ethyl diazoacetate (0.6 mmol,1.5 equiv.). The solution was placed in a pre-heated oil bath and stirred at 80 °C until full conversion of the isocyanide was observed on TLC. Subsequently, the reaction mixture was cooled to 0 °C and diluted with MeOH to a concentration of 0.125 M, after which $NaBH_3CN$ (33 mg, 0.53 mmol, 1.05 equiv.). After 30 minutes $NaBH_3CN$ (31 mg, 0.50 mmol, 1.0 equiv.)and a few drops of AcOH were added. The resulting mixture was stirred at 0 °C until full conversion of the spiroindolenine intermediate was observed on TLC. Subsequently, the mixture was neutralized with Na_2CO_3 and diluted with CH_2Cl_2. The aqueous layer was extracted with CH_2Cl_2 (3 x). The combined organic layers were washed with brine, dried over Na_2SO_4, filtered, and concentrated *in vacuo*. This was followed by purification via FCC using *c*Hex:EtOAc = 2:1 as eluent to obtain both diastereomers separately. (*Combined yield:* 81 mg, 0.22 mmol, 54%, *d.r.* = 3:1). *d.r.* determined via ^{1}H-NMR of crude product mixture.

D1 (major) white solid (61 mg, 0.17 mmol, 41%); $\mathbf{R_f}$ = 0.44 (*c*Hex:EtOAc = 2:1); **^{1}H NMR** (500 MHz, $CDCl_3$): δ 8.09 (s, 1H), 7.20 – 7.13 (m, 2H), 7.10 (td, J = 7.7, 1.2 Hz, 1H), 6.84 – 6.75 (m, 2H), 6.66 (d, J = 7.6 Hz, 1H), 6.60 (d, J = 7.8 Hz, 1H), 6.51 (t, *J* = 1.8 Hz, 1H), 4.53 (s, 1H), 4.09 (q, *J* = 6.9 Hz, 2H), 4.00 (dd, *J* = 10.0, 7.3 Hz, 1H), 3.83 (dd, *J* = 10.3, 6.7 Hz, 1H), 3.65 (s, 3H), 3.57 (t, *J* = 6.9 Hz, 1H), 3.46 – 3.32 (m, 2H), 1.23 (t, *J* = 7.1 Hz, 3H) ppm; **^{13}C{^{1}H} NMR** (126 MHz, $CDCl_3$): δ 171.4 (C_q), 170.7 (C_q), 159.6 (C_q), 151.4 (C_q), 140.4 (C_q), 132.3 (C_q), 129.6 (CH), 128.9 (CH), 123.7 (CH), 119.9 (CH), 119.6 (CH), 113.6 (CH), 112.9 (CH), 110.4 (CH), 78.1 (CH), 61.5 (C_q), 58.8 (CH_2), 55.1 (CH), 53.9 (CH_2), 52.6 (CH_3), 49.8 (CH_2), 14.7 (CH_3) ppm; **HRMS (ESI):** m/z calculated for $C_{22}H_{25}N_2O_3$ [M+H$^+$] = 365.1860, found = 365.1868.

D2 (minor): yellow oil (20 mg, 0.06 mmol, 13%); $\mathbf{R_f}$ = 0.26 (*c*Hex: EtOAc = 2:1); **^{1}H NMR** (500 MHz, $CDCl_3$): δ 8.11 (s, 1H), 7.04 (t, *J* = 7.9 Hz, 1H), 6.95 (td, *J* = 7.7, 1.1 Hz, 1H), 6.67 (ddd, *J* = 8.2, 2.6, 0.9 Hz, 1H), 6.58 (d, *J* = 7.7 Hz, 2H), 6.43 (td, *J* = 7.5, 0.7 Hz, 1H), 6.36 (t, *J* = 1.9 Hz, 1H), 6.29 (d, *J* = 7.5 Hz, 1H), 4.49 (s, 1H), 4.18 – 4.04 (m, 2H), 4.00 – 3.88 (m, 2H), 3.72 (br, 1H), 3.66 (s, 2H), 3.58 (s, 3H), 3.49 (dd, *J* = 6.5, 3.7 Hz, 1H), 1.23 (t, *J* = 7.1 Hz, 3H) ppm; **^{13}C{^{1}H} NMR** (126 MHz, $CDCl_3$): δ 171.3 (C_q), 169.2 (C_q), 159.3 (C_q), 151.8 (C_q), 141.0 (C_q), 129.1 (CH), 128.5 (CH), 128.2 (C_q), 126.3 (CH), 120.5 (CH), 118.7 (CH), 113.7 (CH), 112.9 (CH), 109.9 (CH), 79.0 (CH), 63.0 (C_q), 59.7 (CH_2), 58.9 (CH_2), 55.2 (CH), 51.6 (CH_3), 50.9 (CH_2), 14.7 (CH_3) ppm; **HRMS (ESI):** m/z calculated for $C_{22}H_{25}N_2O_3$ [M+H$^+$] = 365.1860 , found = 365.1867.

6 Diazo scope

This section will describe the different attempted carbene precursor in the $Bu_4N[Fe(CO)_3NO]$ catalyzed carbene transfer to tryptamine derived isocyanides. The diazo compounds used, were either in stock in the laberotory or synthesized according to well established literature procedures.

6.1 General procedure E: Iron catalyzed carbene transfer spirocyclisation cascade using substituted α-diazo esters

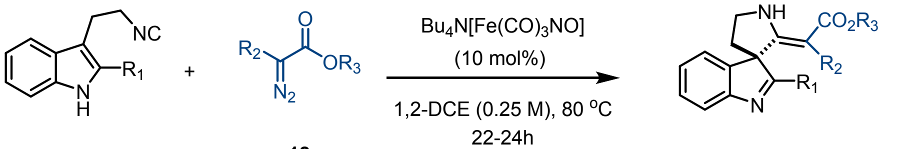

1a-b **18** **23**

To a flame dried Schlenk flask under N_2 atmosphere, charged with a stirring bean, was added $Bu_4N[Fe(CO)_3NO]$ (20.6 mg, 0.025 mmol, 0.05 equiv.). Subsequently, 1,2-DCE was added (2 mL), and the mixture was stirred until the catalyst was dissolved. This was followed by the addition of tryptamine-derived isocyanide (0.5 mmol, 1.0 equiv.) and α-diazoacetate (**18**) (0.6 mmol, 1.2 equiv.). The solution was placed in a pre-heated oil bath and stirred at 80 °C for 22-24 h. Subsequently, the reaction mixture cooled to room temperature and directly purified via flash column chromatography using a mixture of EtOAc:Chex as eluent to provide the title compound.

Dimethyl-(R,Z)-2-(spiro[indole-3,3'-pyrrolidin]-2'-ylidene)succinate (23ab)

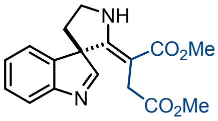

Dimethyl-(R,Z)-2-(spiro[indole-3,3'-pyrrolidin]-2'-ylidene)succinate was prepared according to General procedure E starting from 3-(2-isocyanoethyl)-1H-indole (85.1 mg, 0.5 mmol, 1.0 equiv.). The title compound was isolated as a white solid (50 mg, 0.16 mmol, 33%). **R_f** = 0.22 (*c*Hex:EtOAc = 1:1); **^{1}H NMR** (600 MHz, $CDCl_3$): δ 8.90 (s, 1H), 8.12 (s, 1H), 7.67 (d, *J* = 7.7 Hz, 1H), 7.40 (td, *J* = 7.6, 1.3 Hz, 1H), 7.31 (d, *J* = 7.4 Hz, 1H), 7.27 (td, *J* = 7.4, 1.1 Hz, 1H), 3.84 (dddd, *J* = 10.1, 7.2, 6.1, 0.9 Hz, 1H), 3.77 (dddd, *J* = 10.1, 7.7, 5.8, 1.0 Hz, 1H), 3.60 (s, 3H), 3.43 (s, 3H), 2.47 (ddd, *J* = 12.8, 7.6, 6.1 Hz, 1H), 2.36 – 2.16 (m, 2H), 2.14 (ddd, *J* = 13.0, 7.5, 5.8 Hz, 1H). ppm; **^{13}C{^{1}H} NMR** (150 MHz, $CDCl_3$): δ 173.0 (C_q), 172.7 (CH), 170.5 (C_q), 160.3 (C_q), 154.6 (C_q), 140.3 (C_q), 129.1 (CH), 127.3 (CH), 122.5 (CH), 122.1 (CH), 85.4 (C_q), 67.2 (C_q), 51.6 (CH_3), 51.1 (CH_3), 45.4 (CH_2), 32.7 (CH_2), 30.7 (CH_2) ppm. **HRMS (ESI):** m/z calculated for $C_{17}H_{19}N_2O_4$ [M+H$^+$] = 315.1339, found = 315.1338.

Dimethyl (R,Z)-2-(2-methylspiro[indole-3,3'-pyrrolidin]-2'-ylidene)succinate (23bb)

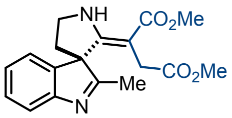

Dimethyl (R,Z)-2-(2-methylspiro[indole-3,3'-pyrrolidin]-2'-ylidene)succinate was prepared according to General procedure E starting from 2-(methyl)-3-(2-isocyanoethyl)-1H-indole (92.1mg, 0.5 mmol, 1.0 equiv.). The title compound was isolated as a white solid (84 mg, 0.26 mmol, 51%). **R_f** = 0.68 (EtOAc); **^{1}H NMR** (600 MHz, $CDCl_3$): 8.95 (s, 1H), 7.53 (d, *J* = 7.7 Hz, 1H), 7.34 (td, *J* = 7.6, 1.3 Hz, 1H), 7.24 (d, *J* = 7.4 Hz, 1H), 7.18 (td, *J* = 7.5, 1.0 Hz, 1H), 3.86 – 3.75 (m, 2H), 3.60 (s, 2H), 3.37 (s, 3H), 2.31 (s, 3H), 2.30 – 2.19 (m, 4H). δ ppm; **^{13}C{^{1}H} NMR** (150 MHz, $CDCl_3$): δ ^{13}C NMR (150 MHz, $CDCl_3$) δ 182.4 (C_q), 172.6 (C_q), 170.7 (C_q), 161.9 (C_q), 154.6 (C_q), 142.1 (C_q), 128.9 (CH), 126.3 (CH), 122.5 (CH), 120.8 (CH), 85.3 (C_q), 67.9 (C_q), 51.4 (CH_3), 51.0 (CH_3), 45.2 (CH_2), 34.3 (CH_2), 30.4 (CH_2), 16.8 (CH_3) ppm. **HRMS (ESI):** m/z calculated for $C_{18}H_{21}N_2O_4$ [M+H$^+$] = 329.1496, found = 329.1495.

Benzyl (R,Z)-2-(2-methylspiro[indole-3,3'-pyrrolidin]-2'-ylidene)-3-oxobutanoate (23be)

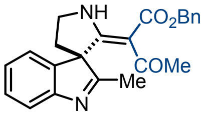

Benzyl (R,Z)-2-(2-methylspiro[indole-3,3'-pyrrolidin]-2'-ylidene)-3-oxobutanoate was prepared according to General procedure E starting from 2-(methyl)-3-(2-isocyanoethyl)-1H-indole (92.1mg, 0.5 mmol, 1.0 equiv.). The title compound was isolated as a white solid (17 mg, 0.05 mmol, 9%). **R_f** = 0.16 (*c*Hex:EtOAc = 3:7); **^{1}H NMR** (500 MHz, $CDCl_3$): δ 12.02 (s, 1H), 7.53 (d, *J* = 7.7 Hz, 1H), 7.34 (td, *J* = 7.7, 7.2, 2.1 Hz, 1H), 7.25 – 7.16 (m, 5H), 7.08 – 7.01 (m, 2H), 4.45 (s, 2H), 3.97 – 3.80 (m, 2H), 2.37 (ddd, *J* = 13.1, 7.6, 5.9 Hz, 1H), 2.31 (s, 3H), 2.26 – 2.16 (m, 4H) ppm; **^{13}C{^{1}H} NMR** (126 MHz, $CDCl_3$): δ 196.7 (C_q), 181.5 (C_q), 168.6 (C_q), 167.0 (C_q), 155.0 (C_q), 141.7 (C_q), 136.3 (C_q), 128.8 (CH), 128.4 (CH), 128.3 (CH), 127.9 (CH), 126.0 (CH), 121.2 (CH), 120.5 (CH), 100.7 (C_q), 69.5 (C_q), 64.8 (CH_2), 45.6 (CH_2), 35.6 (CH_2), 29.5 (CH_3), 17.0 (CH_3) ppm. **HRMS (ESI):** m/z calculated for $C_{23}H_{23}N_2O_3$ [M+H$^+$] = 375.1703, found = 375.1702.

6.2 Effect of catalyst loading on yield using susbtituted α-diazo ester **18b**

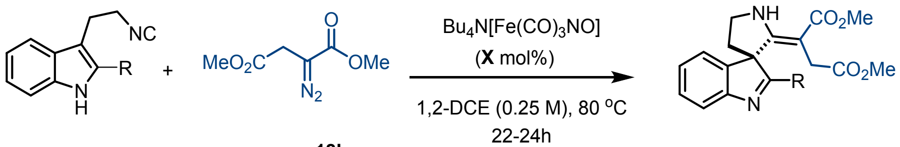

1a-b **18b** **23ab-bb**

To a flame dried Schlenk flask under N_2 atmosphere, charged with a stirring bean, was added $Bu_4N[Fe(CO)_3NO]$ (20.6 mg, 0.025 mmol, 0.05 equiv.). Subsequently, 1,2-DCE was added (2 mL), and the mixture was stirred until the catalyst was dissolved. This was followed by the addition of tryptamine-derived isocyanide **1b** (0.5 mmol, 1.0 equiv.) and α-diazoacetate (**18b**) (0.6 mmol, 1.2 equiv.). The solution was placed in a pre-heated oil bath and stirred at 80 °C for 22-24 h. Subsequently, the reaction mixture cooled to room temperature and filtered over a plug of silica, using CH_2Cl_2:MeOH = 95:5 as eluent. The volatiles were removed, and the crude reaction mixture was analyzed via ^{1}H-NMR, using 1,3,5-trimethoxybenzene as internal standard.

Table S3. Effect on catalyst loading of $Bu_4N[Fe(CO)_3NO]$-catalysed carbene transfer to C2-susbtituted tryptamine-derived isocyanides (**1a-b**)

Entry	Catalyst (mol %)	1a-b (R=)	Yield 23ab (%)[a]
**1**	$Bu_4N[Fe(CO)_3NO]$ (5.0)	**1a** (-H)	18
**2**	$Bu_4N[Fe(CO)_3NO]$ (10.0)	**1a** (-H)	38 (33)[b]
**3**	$Bu_4N[Fe(CO)_3NO]$ (5.0)	**1b** (-Me)	46
**4**	$Bu_4N[Fe(CO)_3NO]$ (10.0)	**1b** (-Me)	60 (50)[b]

[a] Reactions performed on a 0.5 mmol scale and stirred for 22 h. Yields determined via ^{1}H-NMR using 1,3,5-trimethoxybenzene as IS. [b] Isolated yields.

7 Towards total synthesis

2-(1*H*-indol-2-yl)ethan-1-ol

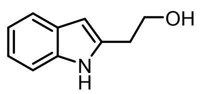

Ethyl 2-(1H-indol-2-yl)acetate (5.0 g, 24.6 mmol, 1.0 equiv.) was dissolved in dry THF (125 mL) and subsequently $LiAlH_4$ (4.67 g, 123 mmol, 5 .0 equiv.) in anhydrous THF (125 mL) was slowly added at 0 °C via a cannula. After addition the reaction mixture was allowed to stir for an hour at room temperature, after which it was diluted with Et_2O (50 mL) and quenched with water (5 mL) and 15% aqueous NaOH solution (5 mL) at 0 °C. More water (15 mL) was added, and the precipitated mixture was stirred for 15 minutes at room temperature. Subsequently, Na_2SO_4 was added to the mixture, which was filtered and concentrated *in vacuo* to yield a dark-red waxy solid (3.80 g, 323.6 mmol, 96%). Spectral data matched those found in literature.[12] **^{1}H NMR** (300 MHz, $CDCl_3$): δ 8.44 (br, 1H), 7.55 (d, *J* = 7.5 Hz, 1H), 7.32 (d, *J* = 7.3 Hz, 1H), 7.14 (t, *J* = 7.2 Hz, 1H), 7.08 (t, *J* = 7.2 Hz, 1H), 6.30 (s, 1H), 3.79 (t, *J* = 5.7 Hz, 2H), 3.01 (t, *J* = 5.8 Hz, 2H), 1.86 (br, 1H).

2-(1*H*-indol-2-yl)ethyl acetate

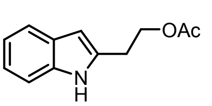

2-(1*H*-indol-2-yl)ethan-1-ol (3.79 g, 23.5 mmol, 1.0 equiv.) was dissolved in a 1:1 mixture of Et_3N (25 mL) and Ac_2O (25 mL) and stirred for one hour at room temperature. After complete conversion of the starting material based on TLC, the crude reaction mixture was concentrated *in vacuo* and partitioned between Et_2O (25 mL) and a saturated Na_2CO_3 solution (25 mL). This was extracted with diethyl ether, dried over Na_2SO_4, filtered, and concentrated *in vacuo* yielding the product as a yellowish solid (4.59 g, 22.6 mmol, 96%). This was used in the follow-up step without further purification. Spectral data matched those found in literature.[13] **^{1}H NMR** (500 MHz, $CDCl_3$): δ 8.10 (s, 1H), 7.55 (d, *J* = 7.8 Hz, 1H), 7.32 (d, *J* = 7.3 Hz, 1H), 7.15 (t, *J* = 7.1 Hz, 1H), 7.09 (t, *J* = 7.1 Hz, 1H), 6.32 (s, 1H), 4.39 (t, *J* = 6.5 Hz, 2H), 3.11 (t, *J* = 6.5 Hz, 2H), 2.11 (s, 3H).

2-(3-(2-isocyanoethyl)-1*H*-indol-2-yl)ethyl acetate

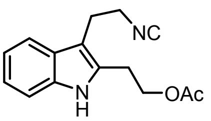

2-(1*H*-indol-2-yl)ethyl acetate (4.58 g, 22.5 mmol, 1.0 equiv.) and *N*-(2,2-dimethoxyethyl)formamide (3.30 g, 24.8 mmol, 1.1 equiv.) in anhydrous CH_2Cl_2 (35 mL) was added dropwise to a solution of trifluoroacetic acid (8.61 mL, 112.5 mmol, 5.0 equiv.) and Et_3SiH (10.8 mL, 67.5 mmol, 3.0 equiv.) in anhydrous CH_2Cl_2 (35 mL). The resulting red solution was left to stir for two hours, after which it was cooled to 0 °C and quenched with a saturated solution of $NaHCO_3$. The crude reaction mixture was extracted with CH_2Cl_2, dried over Na_2SO_4, filtered, and concentrated *in vacuo*. The crude formamide was purified by FCC (gradient: 20% → 80% EtOAc in cyclohexane) to obtain the compound as a brown oil (2.77 g, 10.1 mmol, 45%). Next, to a solution of crude 2-(3-(2-formamidoethyl)-1*H*-indol-2-yl)ethyl acetate (2.77 g, 10.1 mmol, 1.0 equiv.) in anhydrous CH_2Cl_2 (20 mL) was added Et_3N (7.0 mL, 50.5 mmol, 5.0 equiv.). The solution was colled to -78 °C and $POCl_3$ (1.42 mL, 15.2 mmol, 1.5 equiv.) was added dropwise. The reaction mixture was stirred for two hours at this temperature, after which it was diluted with water (20 mL) and extracted with CH_2Cl_2, dried over Na_2SO_4, filtered, and concentrated *in vacuo*. FCC (EtOAc:*c*Hex = 1:1) yielded the product as a yellow oil (1.92 g, 7.48 mmol, 74%). **R_f** = 0.59 (EtOAc/cyclohexane 1:1); **^{1}H NMR** (500 MHz, $CDCl_3$): δ 8.22 (s, 1H), 7.47 (d, *J* = 7.8 Hz, 1H), 7.34 (d, *J* = 8.1 Hz, 1H), 7.19 (t, *J* = 8.1 Hz, 1H), 7.13 (t, *J* = 8.1 Hz, 1H), 4.36 (t, *J* = 6.5 Hz, 2H), 3.62 (t, *J* = 7.3 Hz, 2H), 3.19-3.09 (m, 4H), 2.11 (s, 3H); **^{13}C{^{1}H} NMR** (126 MHz, $CDCl_3$): δ 171.0 (C_q), 156.4 (C_q), 135.7 (C_q), 133.1 (C_q), 127.6 (C_q), 122.2 (CH), 119.9 (CH), 117.8 (CH), 111.0 (CH), 107.9 (C_q), 63.8 (CH_2), 42.3 (CH_2), 25.9 (CH_2), 25.0 (CH_2), 21.2 (CH_3); **HRMS (ESI):** m/z calculated for $C_{15}H_{17}N_2O_2$ [M+H$^+$] = 257.1285 , found = 257.1282.

2-(3-(2-isocyanoethyl)-1*H*-indol-2-yl)ethan-1-ol (1y)

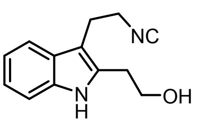

2-(3-(2-isocyanoethyl)-1*H*-indol-2-yl)ethyl acetate (1.86 g, 7.26 mmol, 1.0 equiv.) was dissolved in THF (30 mL) along with MeOH (6 mL) and water (6 mL). The solution was cooled using an ice-bath and KOH (2.00 g, 35.6 mmol, 4.9 equiv.) was added. This reaction mixture was stirred for 20 minutes, quenched with a saturated NH_4Cl solution, and extracted with EtOAc (3 x). The combined organic layers were dried over Na_2SO_4, filtered, and concentrated *in vacuo* to yield the product as an off-white solid (1.35 g, 6.33 mmol, 87%). **R_f** = 0.23 (EtOAc/cyclohexane 1:1); **^{1}H NMR** (500 MHz, $CDCl_3$): δ 8.72 (s, 1H), 7.46 (d, *J* = 7.6 Hz, 1H), 7.33 (d, *J* = 8.0 Hz, 1H), 7.17 (t, *J* = 8.0 Hz, 1H), 7.11 (t, *J* = 7.6 Hz, 1H), 3.97 (t, *J* = 5.6 Hz, 2H), 3.61 (tt, *J* = 7.0, 1.8 Hz, 2H), 3.12 (tt, *J* = 7.0, 1.8 Hz, 2H), 3.02 (t, *J* = 5.6 Hz, 2H), 2.07 (s, 1H); **^{13}C{^{1}H} NMR** (126 MHz, $CDCl_3$): δ 155.8 (t, *J* = 6.0 Hz, C_q), 135.6 (C_q), 135.5 (C_q), 127.5 (C_q), 121.8 (CH), 119.6 (CH), 117.6 (CH), 111.0 (CH), 106.8 (C_q), 62.5 (CH_2), 42.4 (t, *J* = 6.1 Hz, CH_2), 28.7 (CH_2), 24.9 (CH_2); **HRMS (ESI):** m/z calculated for $C_{13}H_{15}N_2O$ [M+H$^+$] = 215.1179, found = 215.1178.

ethyl (*Z*)-2-(2-(2-hydroxyethyl)spiro[indoline-3,3'-pyrrolidin]-2'-ylidene)acetate (25y)

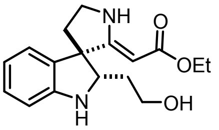

2-(3-(2-isocyanoethyl)-1*H*-indol-2-yl)ethan-1-ol (1.35 g, 6.33 mmol, 1.0 equiv.) was added to a solution of $Bu_4[Fe(CO)_3NO]$ (260 mg, 0.63 mmol, 0.10 equiv.) in anhydrous 1,2-DCE (25 mL). Ethyl 2-diazoacetate (0.94 mL, 7.60 mmol, 1.2 equiv.) was added and the mixture was heated to 80 °C for 1.5 hours, and then allowed to cool to room temperature. The reaction was placed in an ice-bath and MeOH (10 mL) and $NaBH_4$ (251 mg, 6.65 mmol, 1.05 equiv.) were added. After complete conversion of the spiroindolenine was observed on TLC, the reaction was quenched with a saturated NH_4Cl solution and extracted with CH_2Cl_2 (3 x). The combined organic layers were dried over Na_2SO_4, filtered, and concentrated *in vacuo*. FCC (gradient: 20% → 80% EtOAc in cyclohexane)

yielded the product as a light-brown solid as a single diastereomer (1.12 g, 3.70 mmol, 59%). **R_f** = 0.28 (EtOAc/cyclohexane 4:1); **^{1}H NMR** (500 MHz, $CDCl_3$): δ 7.97 (s, 1H), 7.08 (t, *J* = 7.7 Hz, 1H), 7.03 (d, *J* = 7.4 Hz, 1H), 6.76 (t, *J* = 7.4 Hz, 1H), 6.67 (d, *J* = 7.8 Hz, 1H), 4.30 (s, 1H), 4.04 (q, *J* = 7.1 Hz, 2H), 3.94-3.76 (m, 3H), 3.69-3.52 (m, 2H), 2.49 (dt, *J* = 13.1, 8.8 Hz,, 1H), 2.17 (ddd, *J* = 13.1, 7.0, 2.8 Hz, 1H), 2.03-1.87 (m, 1H), 1.84-1.49 (m, 3H), 1.20 (t, *J* = 7.1 Hz, 3H) ppm; **^{13}C{^{1}H} NMR** (126 MHz, $CDCl_3$): δ 171.2 (C_q), 167.0 (C_q), 150.9 (C_q), 132.3 (C_q), 128.7 (CH), 123.8 (CH), 119.6 (CH), 110.3 (CH), 79.5 (CH), 68.6 (CH), 61.9 (CH_2), 60.1 (C_q), 58.7 (CH_2), 44.9 (CH_2), 37.0 (CH_2), 33.8 (CH_2), 14.7 (CH_3) ppm; **HRMS (ESI):** m/z calculated for $C_{17}H_{23}N_2O_3$ [M+H$^+$] = 303.1703 , found = 303.1705.

ethyl 2,3,5,6,6a,7-hexahydro-1*H*-pyrrolo[2,3-d]carbazole-4-carboxylate (26)

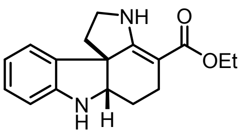

To a mixture of imidazole (0.31 g, 4.6 mmol, 1.35 equiv.), PPh_3 (1.15 g, 4.4 mmol 1.30 equiv.) and iodine (1.12 g, 4.4 mmol, 1.30 equiv.) in CH_2Cl_2 (35 mL) was added ethyl (*Z*)-2-(2-(2-hydroxyethyl)spiro[indoline-3,3'-pyrrolidin]-2'-ylidene)acetate (1.02 g, 3.4 mmol, 1.0 equiv.). After heating for an hour at reflux, the reaction mixture was allowed to cool to room temperature, after which MeOH (5 mL) was added causing the reaction mixture to turn to a clear solution. This solution was washed with a saturated Na_2SO_3 solution, and subsequently extracted with CH_2Cl_2 (3x). The combined organic layer was dried over Na_2SO_4, filtered, and concentrated *in vacuo*. FCC (gradient: 5% → 40% EtOAc in cyclohexane) yielded the product as a light yellow solid and as a single diastereomer (858 mg, 3.0 mmol, 88%). **R_f** = 0.30 (EtOAc:*c*Hex = 1:4); **^{1}H NMR** (500 MHz, $CDCl_3$): δ 7.53 (s, 1H), 7.03 (t, *J* = 7.8 Hz, 1H), 6.97 (d, *J* = 7.5 Hz, 1H), 6.65 (t, *J* = 7.5 Hz,1H), 6.61 (d, *J* = 7.8 Hz, 2H), 4.11 (q, *J* = 7.1 Hz, 2H), 3.94 (dd, *J* = 5.3, 2.9 Hz, 1H), 3.76 (td, *J* = 10.4, 6.1 Hz, 1H), 3.59 (ddd, *J* = 10.8, 9.2, 2.2 Hz, 1H), 2.42 (dt, *J* = 15.1, 4.5 Hz, 1H), 2.29 (dd, *J* = 12.0, 6.0 Hz, 1H), 2.14-2.05 (m,1H), 1.89 (ddd, *J* = 14.8, 10.9, 3.5 Hz, 1H), 1.75-1.59 (m, 2H), 1.24 (t, *J* = 7.1 Hz, 3H) ppm; **^{13}C{^{1}H} NMR** (150 MHz, $CDCl_3$): δ 169.4 (C_q), 162.8 (C_q), 150.2 (C_q), 132.8 (C_q), 128.6 (CH), 123.2 (CH), 118.8 (CH), 109.0 (CH), 89.6 (C_q), 63.7 (CH), 59.0 (CH_2), 55.4 (C_q), 44.2 (CH_2), 39.4 (CH_2), 33.5 (CH_2), 18.5 (CH_2), 14.8 (CH_3) ppm; **HRMS (ESI):** m/z calculated for $C_{17}H_{21}N_2O_2$ [M+H$^+$] = 285.1597, found = 285.1598.

7-(tert-butyl) 4-ethyl 1,2,3,5,6,6a-hexahydro-7H-pyrrolo[2,3-d]carbazole-4,7 dicarboxylate (20)

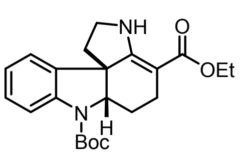

Ethyl-2,3,5,6,6a,7-hexahydro-1*H*-pyrrolo[2,3-d]carbazole-4-carboxylate (142 mg, 0.5 mmol, 1.0 equiv.) was dissolved in anhydrous CH_2Cl_2 (0.5 M), followed by the addition of DMAP (12 mg, 0.1 mmol, 0.2 equiv.) and Boc_2O (372 mg, 1.5 mmol, 3.0 equiv.). No full conversion was observed on TLC after 24 h, and an additional portion of Boc_2O (164 mg, 0.75 mmol, 1.5 equiv.) was added. After 48 hours no full conversion was observed and an additional amount of Boc_2O (372 mg, 1.5 mmol, 1.5 equiv.) and DMAP (12 mg, 0.1 mmol, 0.2 equiv.) were added. An additional potion of Boc_2O (372 mg, 1.5 mmol, 1.5 equiv.) and DMAP (12 mg, 0.1 mmol, 0.2 equiv.) were after 72 h and stirred until full conversion was observed. After completion of the reaction, the reaction was diluted with CH_2Cl_2, washed with H_2O and brine and dried over Na_2SO_4, followed by filtration and concentration *in vacuo*. The crude reaction mixture was then purified by FCC using EtOAc:cHex = 1:9 as eluent to obtain the product as a white foam (136 mg, 0.35 mmol, 71%). Characterization data is accordance with reported in the literature.[8] **R_f** = 0.26 EtOAc:*c*Hex = 1:9); **^{1}H NMR** (600 MHz, $CDCl_3$): δ 7.97 – 7.34 (m, 1H), 7.18 (s, 1H), 7.01 (d, *J* = 7.5 Hz, 1H), 6.90 (td, *J* = 7.5, 1.1 Hz, 1H), 4.46 (m, 1H), 4.11 (qd, J = 7.1, 1.3 Hz, 1H), 3.74 (td, J = 10.3, 6.3 Hz, 1H), 3.61 (t, *J* = 9.5 Hz, 1H), 2.48 – 2.38 (m, 1H), 2.26 (dd, J = 12.1, 6.1 Hz, 1H), 2.22 – 2.05 (m, 2H), 1.73 – 1.52 (m, 12H), 1.24 (t, J = 7.1 Hz, 3H). ppm; **^{13}C{^{1}H} NMR** (150 MHz, $CDCl_3$): (Prescence of rotameric signals) δ 169.3 (C_q), 162.2 (C_q), 152.0 (C_q), 142.3 (C_q), 134.4 (C_q), 128.7 (CH), 123.0 (CH), 122.7 (CH), 114.7 (CH), 89.9 (C_q), 81.1 (C_q), 66.2 (CH), 59.1 (CH_2), 53.8 (C_q), 44.0 (CH_2), 39.3 (CH_2), 31.4 (CH_2), 28.6 (CH_3), 18.4 (CH_2), 14.8 (CH_3). ppm. **HRMS (ESI):** m/z calculated for $C_{22}H_{29}N_2O_4$ [M+H$^+$] = 385.2122, found = 385.2127.

8 References

[1] Klein. J.E.M.N.; Rommel, S.; Plietker, B.; *Organometallics*, **2014**, *33*, 5802-5810.
[2] Matheis, C.; Krause, T.; Bragoni, V.; Goossen, L. J.; *Chem. A Eur. J.*, **2016**, *22*, 12270-12273.
[3] Dar'In, D.; Kantin, G.; Krasavin, M.; *Synthesis,* **2019**, *51*, 4284–4290.
[4] Dar'In, D.; Kantin, G.; Krasavin, M. A.; *Chem. Commun.*, **2019**, *55*, 5239–5242.
[5] Sar, S.; Das, R.; Sen, S., *Adv. Synth. Catal.*, **2021**, *363*, 3521–3531.
[6] Peng, C.; Cheng, J.; Wang, J.; *J. Am. Chem. Soc.*, **2007**, *129*, 8708–8709.
[7] Saya, J. M.; Oppelaar, B.; Cioc, R. C.; Van Der Heijden, G.; Vande Velde, C. M. L.; Orru, R. V. A.; Ruijter, E. *Chem. Commun.* **2016**, *52*, 12482–12485.
[8] Saya, J. M.; Roose, T. R.; Peek, J. J.; Weijers, B.; de Waal, T. J. S.; Vande Velde, C. M. L.; Orru, R. V. A.; Ruijter, E. *Angew. Chem. Int. Ed.* **2018**, *57*, 15232–15236.
[9] Zhao, X.; Liu, X.; Mei, H.; Guo, J.; Lin, L.; Feng, X. *Angew. Chem. Int. Ed.* **2015**, *54*, 4032–4035.
[10] Liu, J.; Li, L.; Yu, L.; Tang, L.; Chen, Q.; Shi, M., *Adv. Synth. Catal.*, **2018**, *360*, 2959-2965.
[11] Chang, W.; Sanyal, D.; Huang, J.-L.; Ittiamornkul, K.; Zhu, Q.; Liu, X.; *Org. Lett.*, **2017**, *19*, 1208-1211.
[12] Huang, H.-Y.; Lin, X.-Y.; Yen, S.-Y.; Liang, C.-F.; *Org. Biomol. Chem.* **2020**, *18*, 5726–5733.
[13] Lee, Y.; Klausen, R. S.; Jacobsen, E. N.; *Org. Lett.* **2011**, *13*, 5564–5567.
[14] Jiang, S.; Cao, W.-B.; Li, H.-Y.; Xu, X.-P.; Ji, S.-J. *Green Chem.* **2021**, *23*, 2619–2623.

9 ^{1}H, ^{13}C, ^{19}F NMR spectra of compounds

^{1}H NMR 500 MHz, DMSO-*d6* **1g**

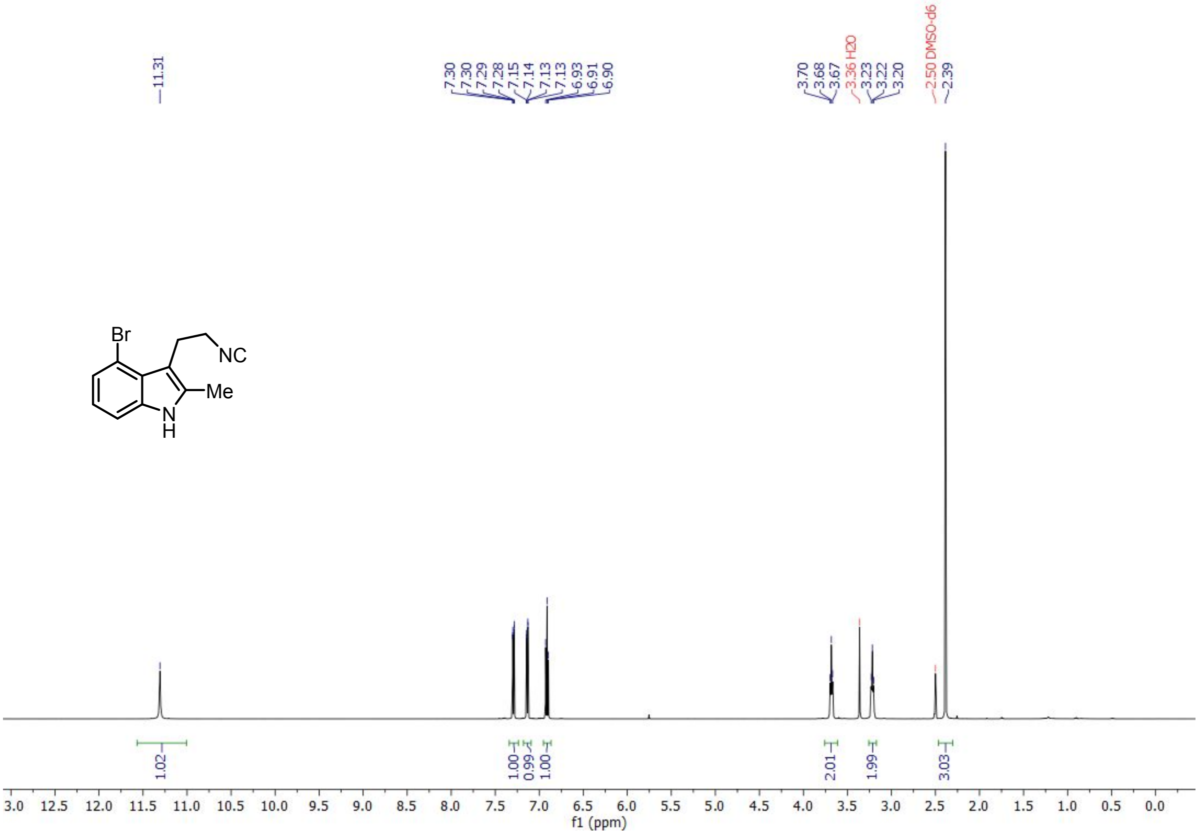

^{13}C{^{1}H} NMR 126 MHz, DMSO-*d6* **1g**

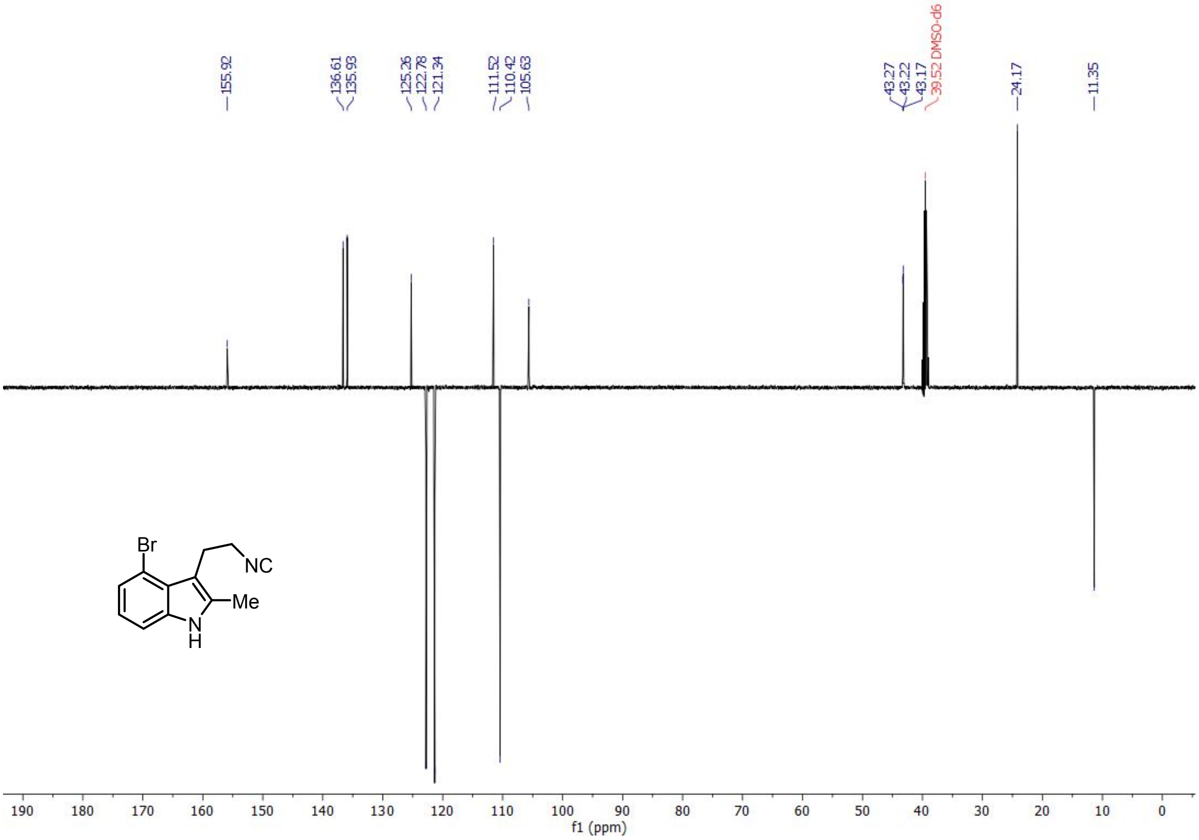

^{1}H NMR 500 MHz, $CDCl_3$ **1h**

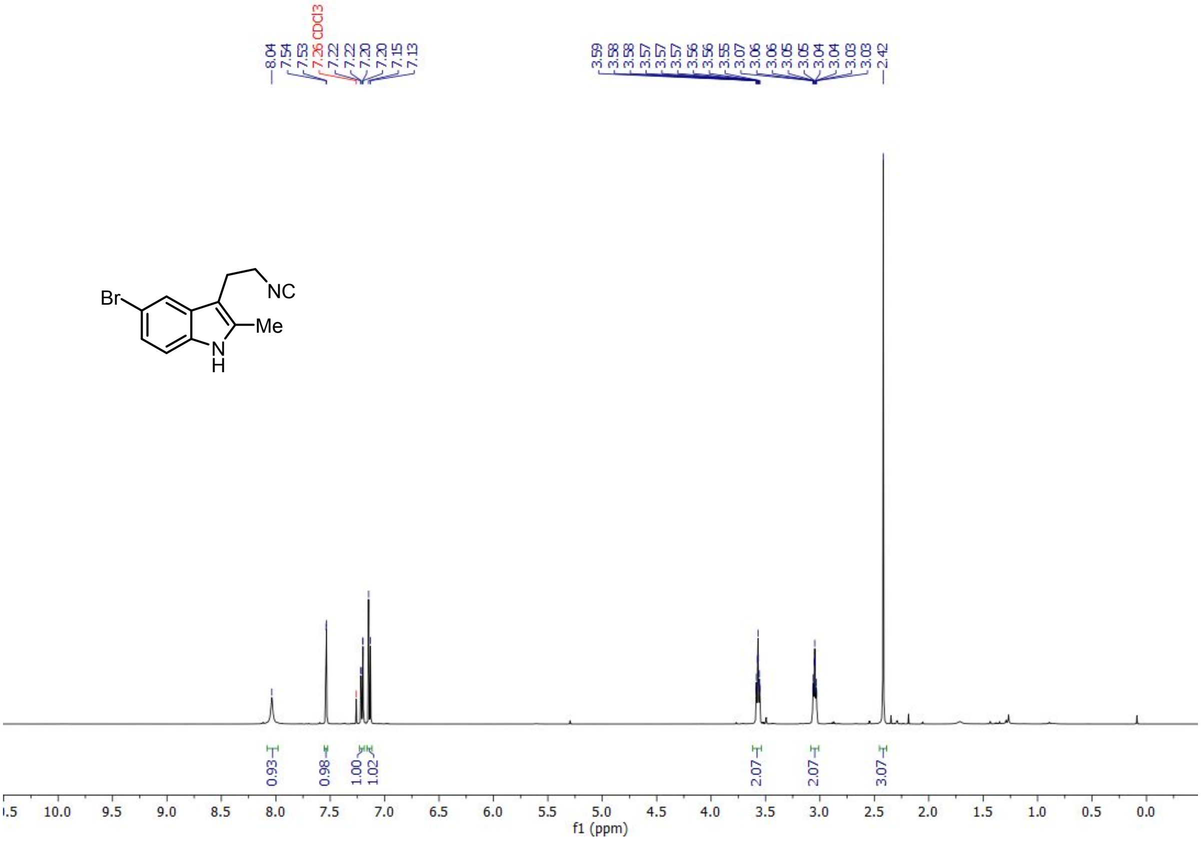

$^{13}C\{^{1}H\}$ NMR 126 MHz, $CDCl_3$ **1h**

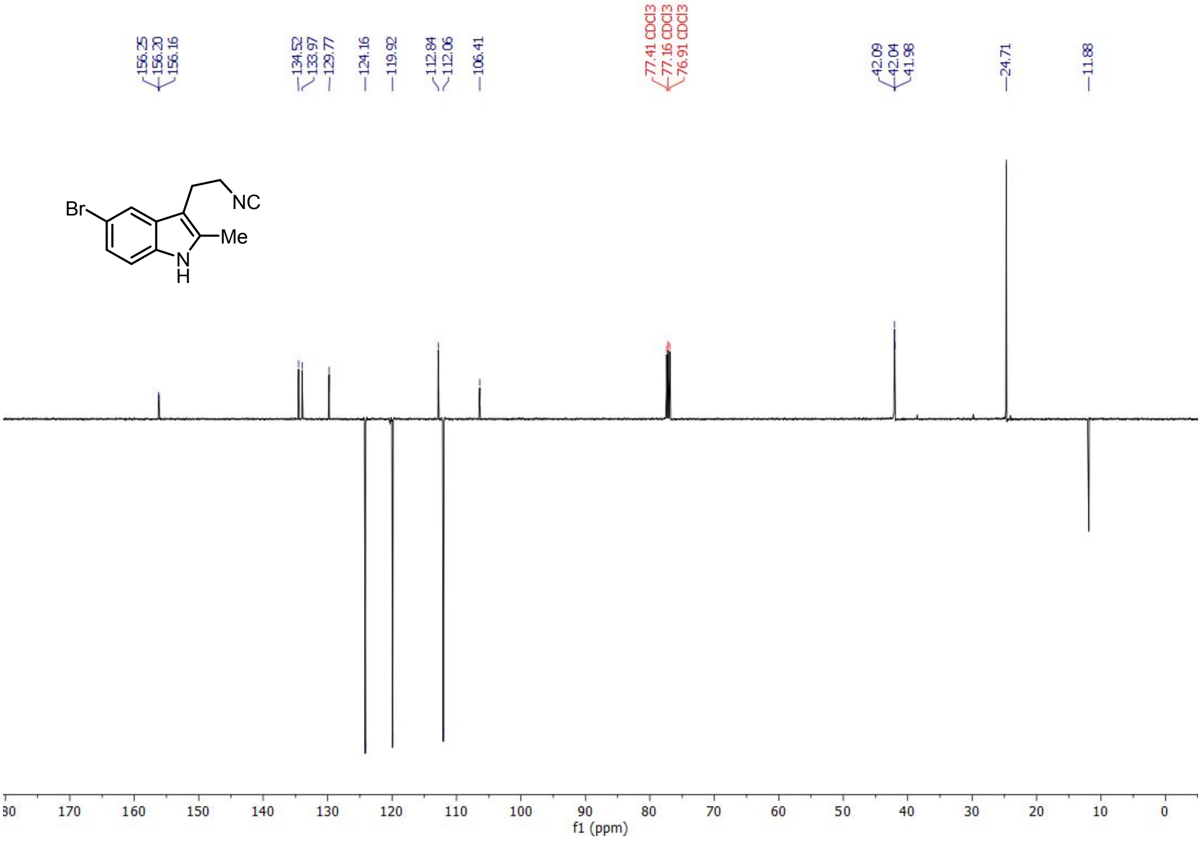

^{1}H NMR 500 MHz, $CDCl_3$ **1i**

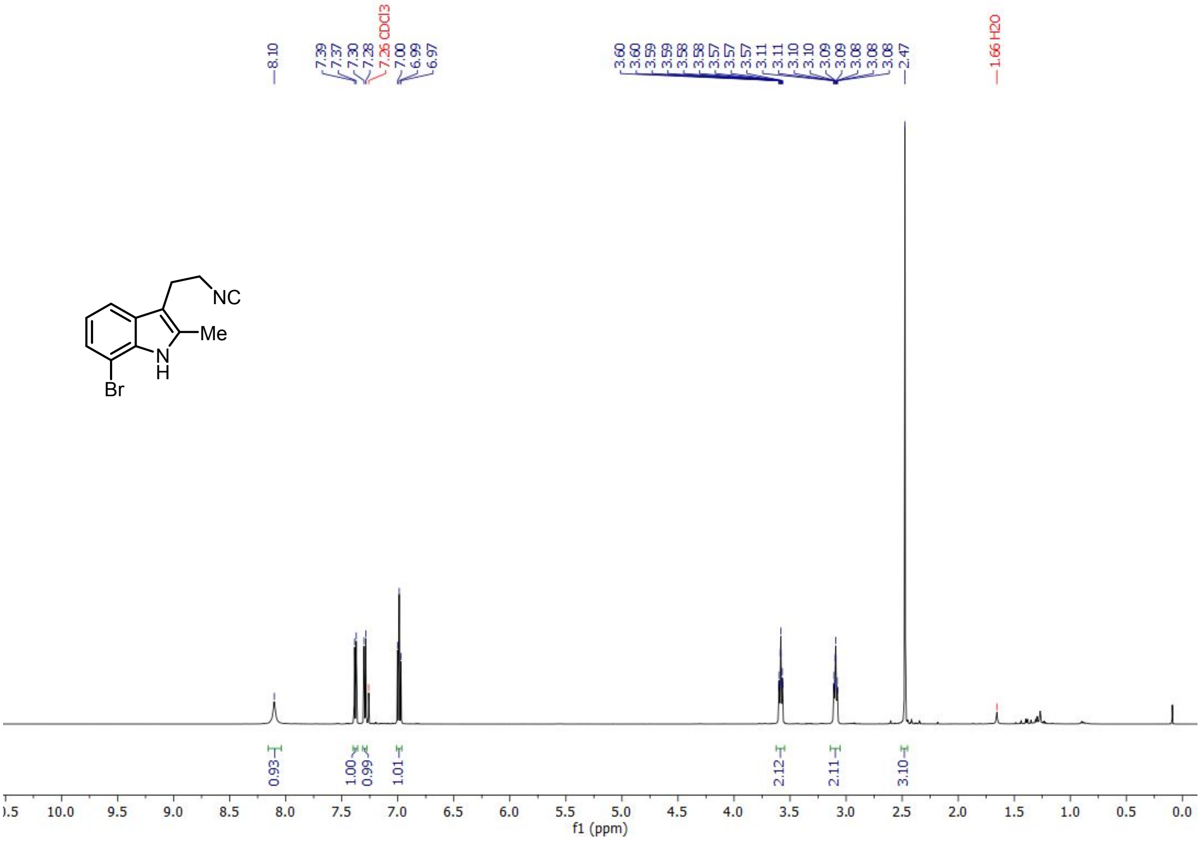

$^{13}C\{^{1}H\}$ NMR 126 MHz, $CDCl_3$ **1i**

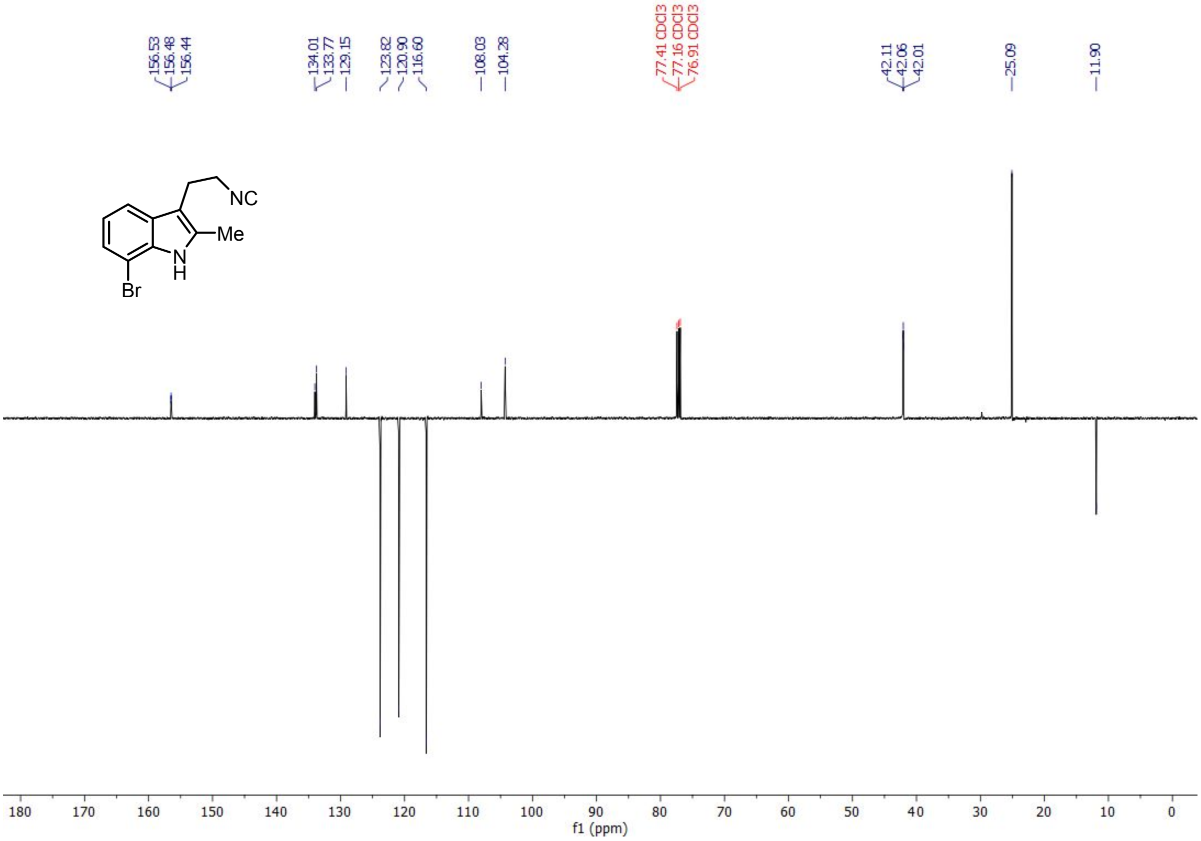

^{1}H NMR 500 MHz, $CDCl_3$ **1k**

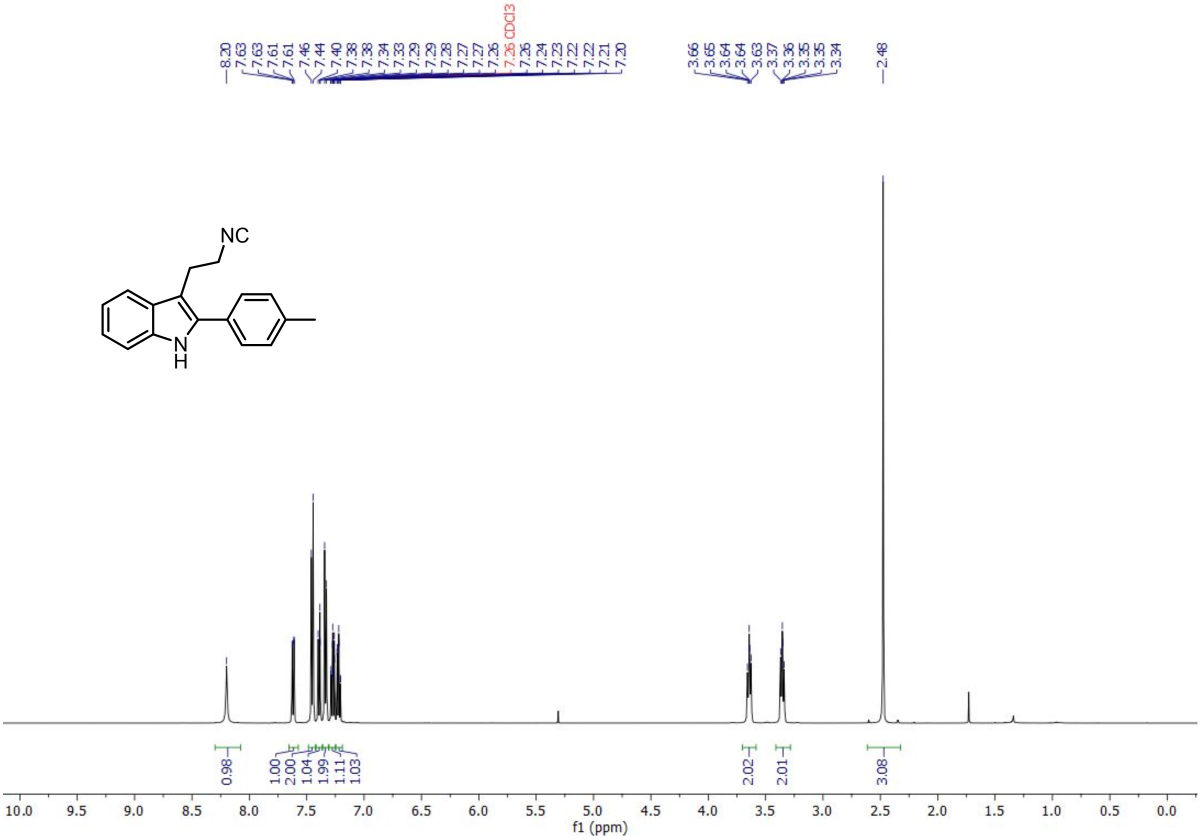

$^{13}C\{^{1}H\}$ NMR 126 MHz, $CDCl_3$ **1k**

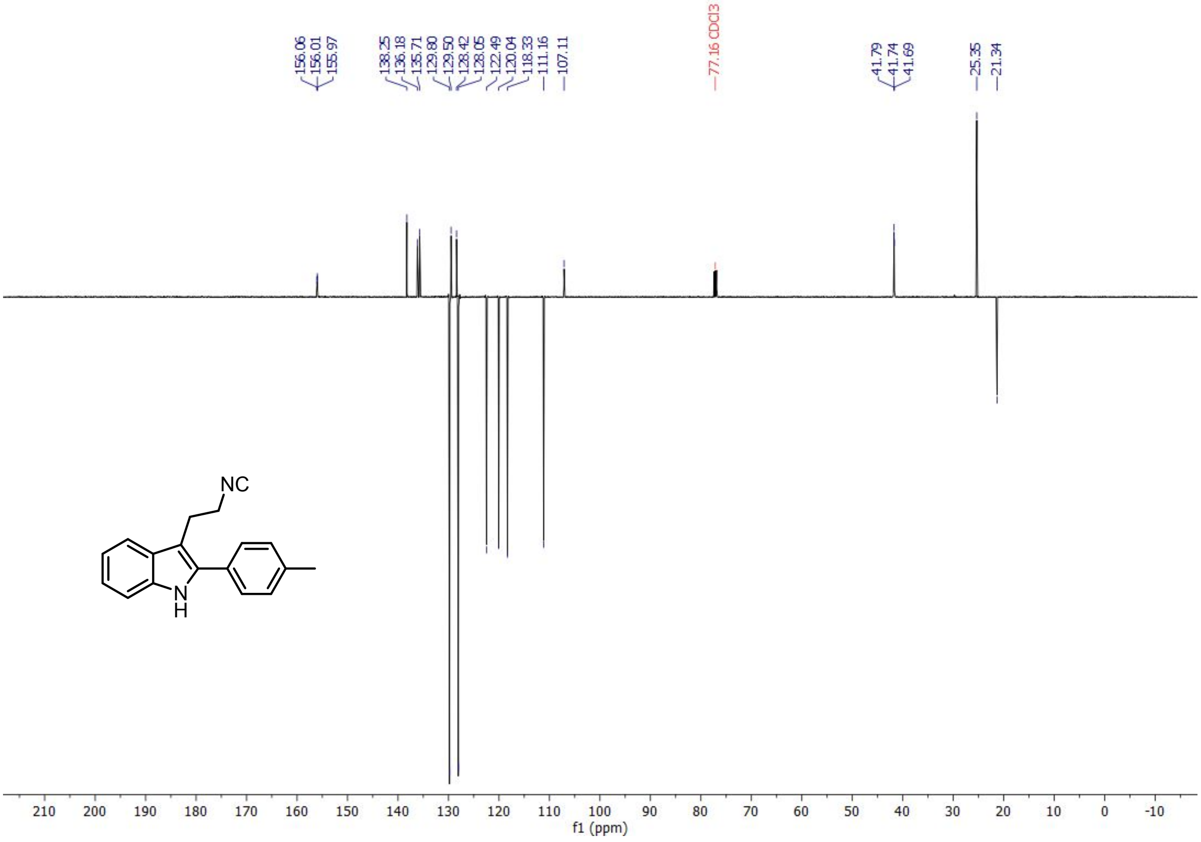

^{1}H NMR 500 MHz, $CDCl_3$ **1l**

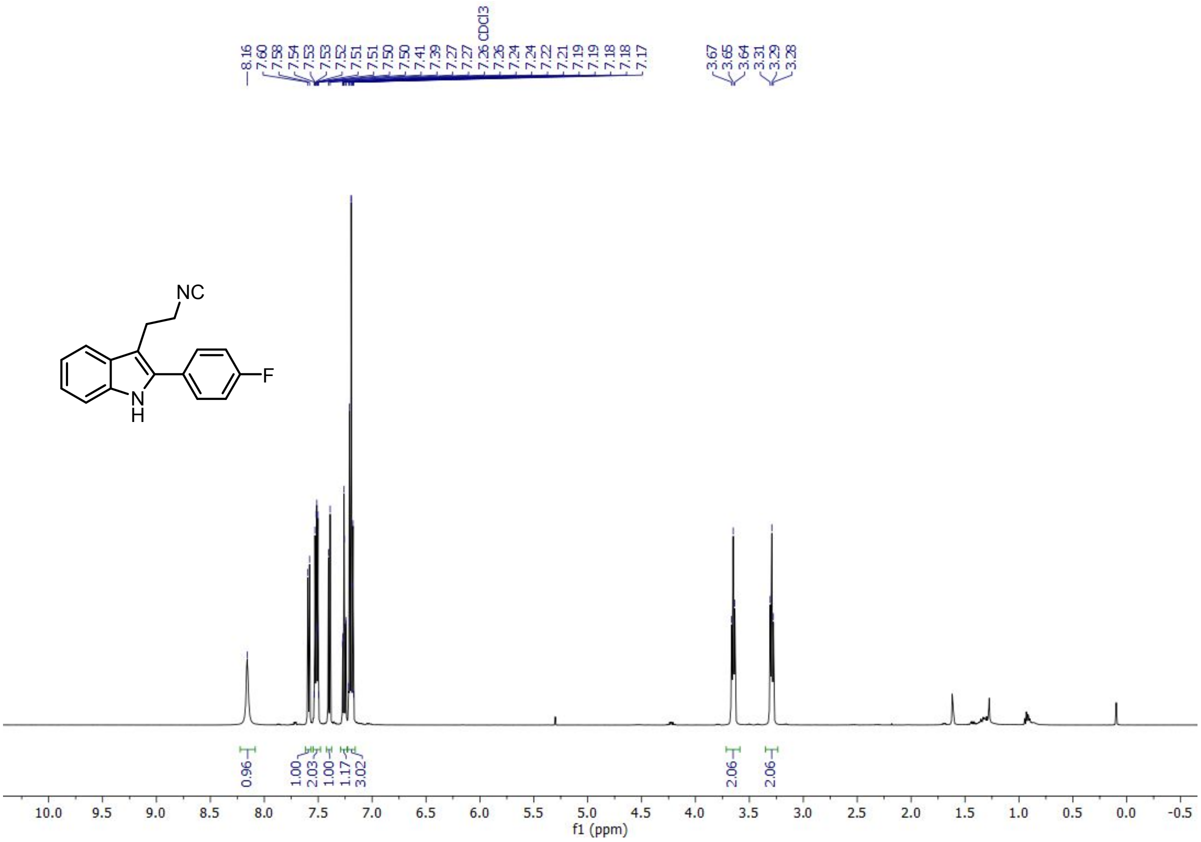

$^{13}C\{^{1}H\}$ NMR 126 MHz, $CDCl_3$ **1l**

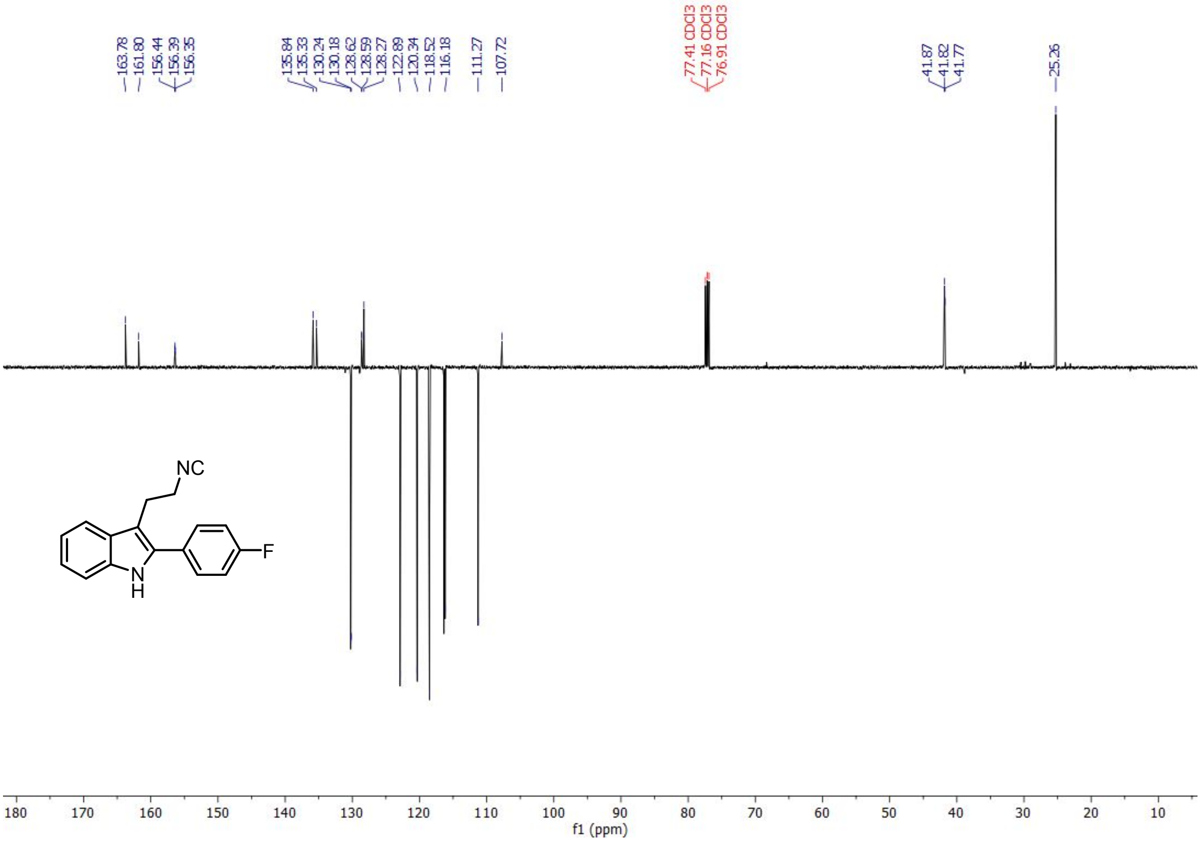

$^{19}F\{^1H\}$ NMR 470.4 MHz, $CDCl_3$ **1l**

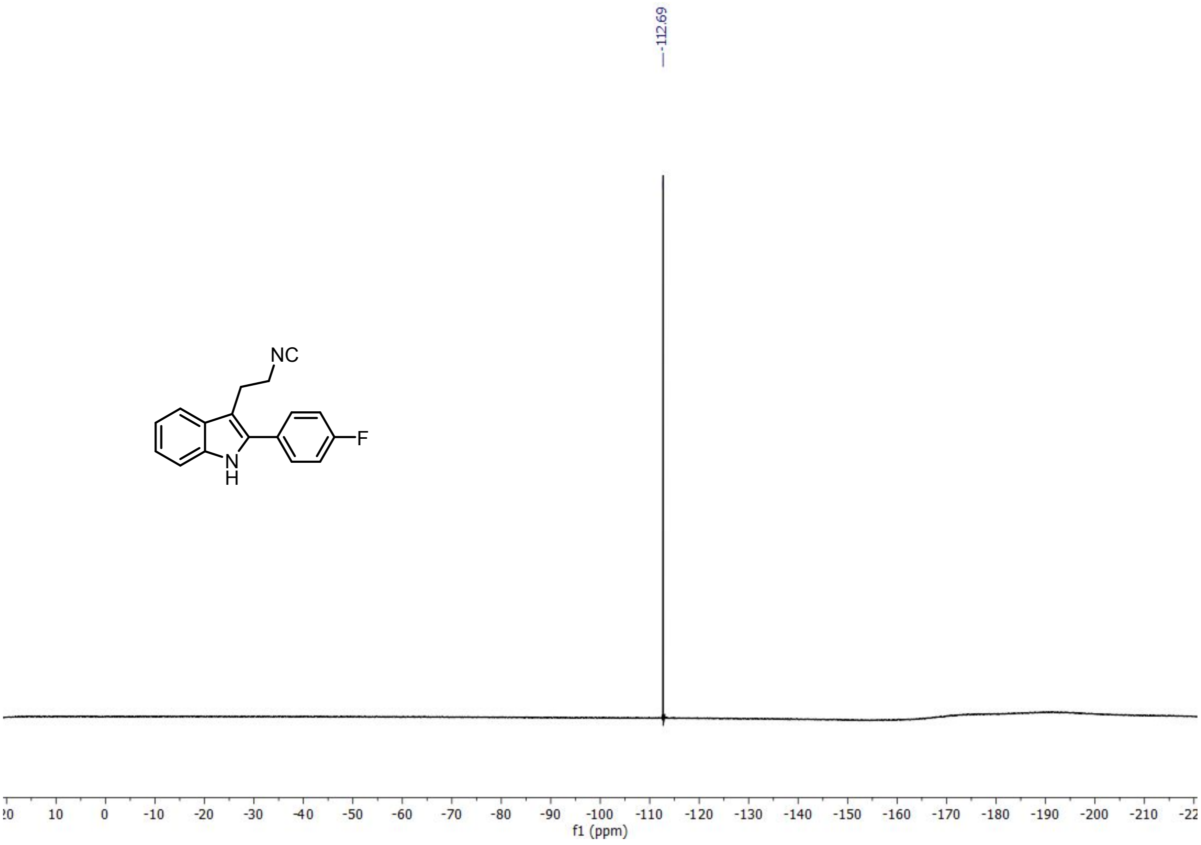

^{1}H NMR 500 MHz, $CDCl_3$ **1m**

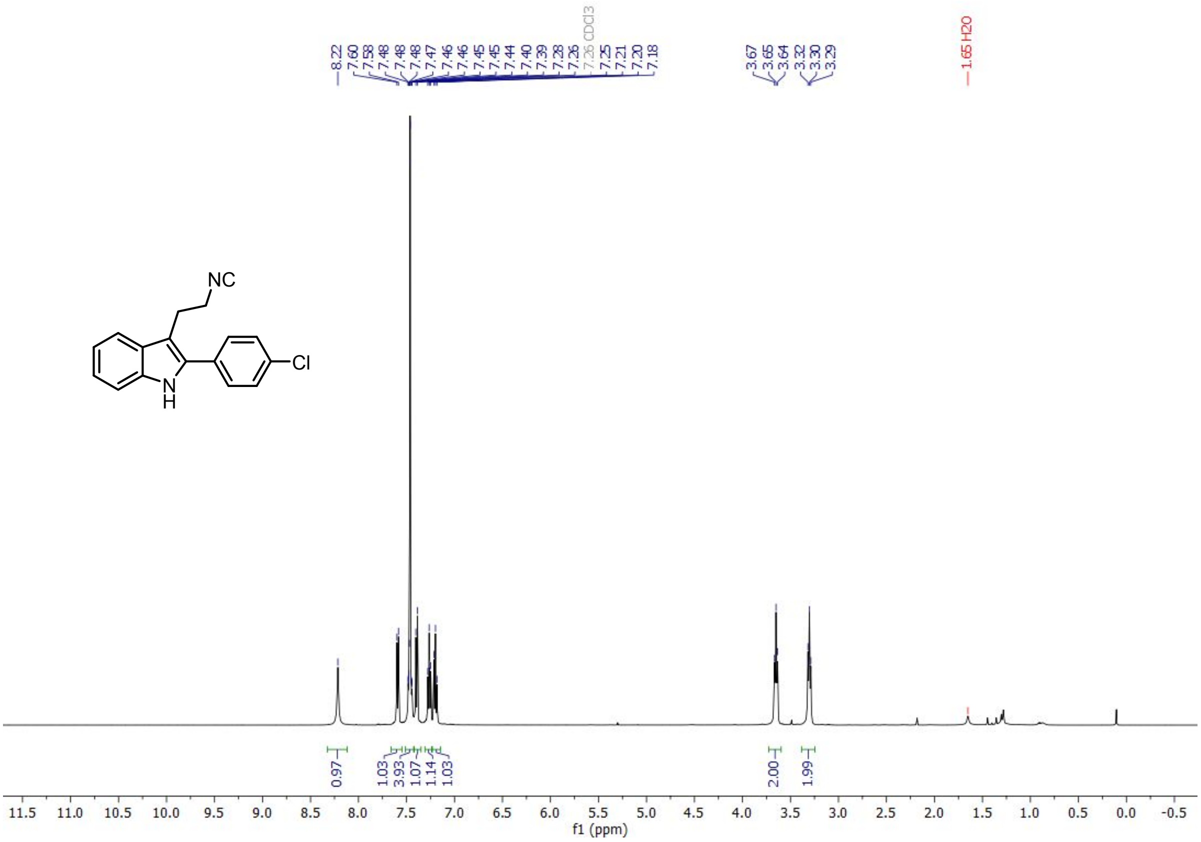

$^{13}C\{^{1}H\}$ NMR 126 MHz, $CDCl_3$ **1m**

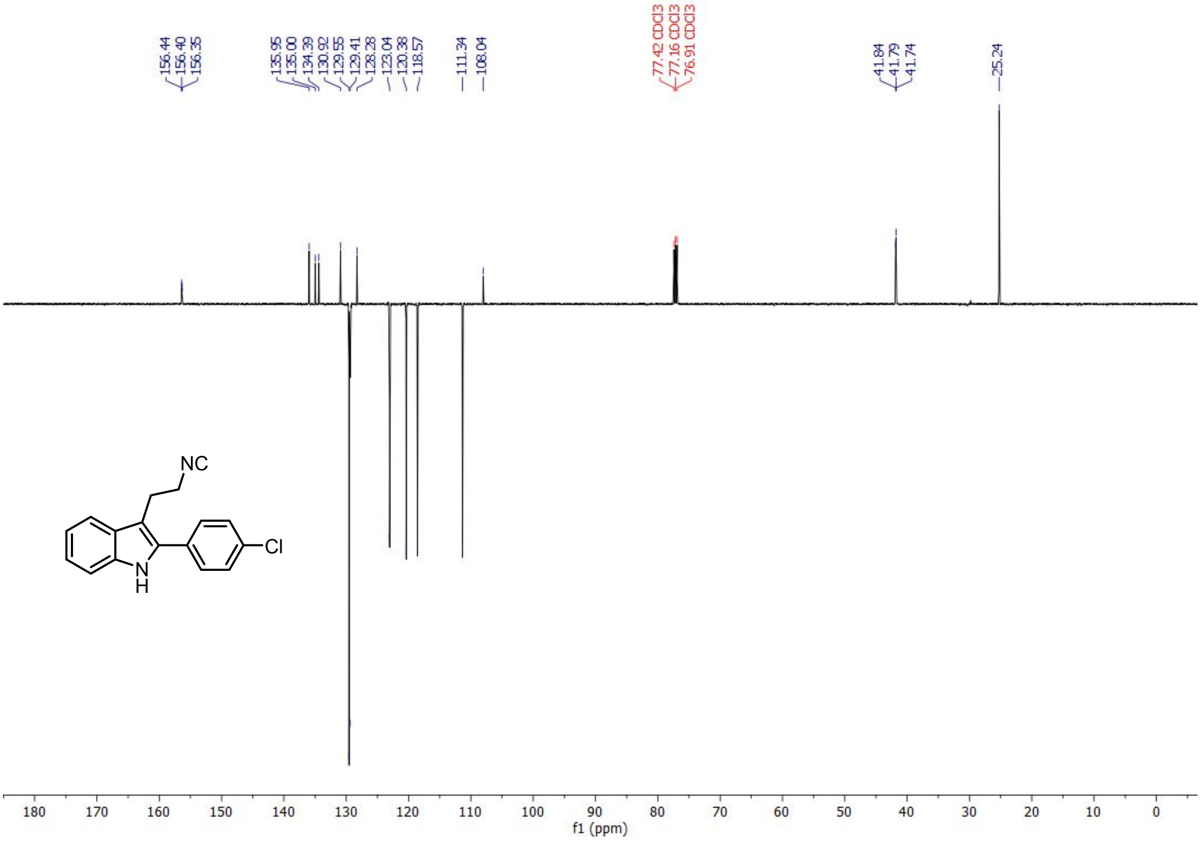

^{1}H NMR 500 MHz, $CDCl_3$ **1n**

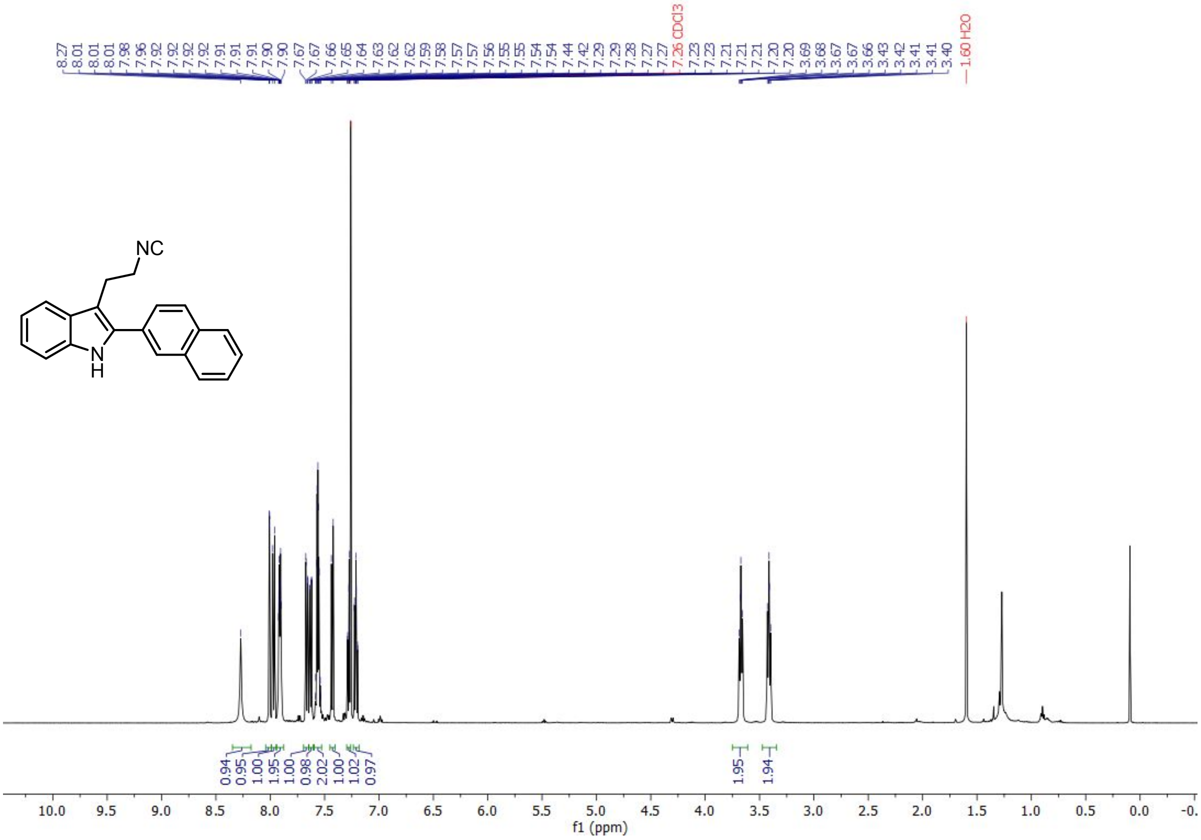

$^{13}C\{^{1}H\}$ NMR 125.7 MHz, $CDCl_3$ **1n**

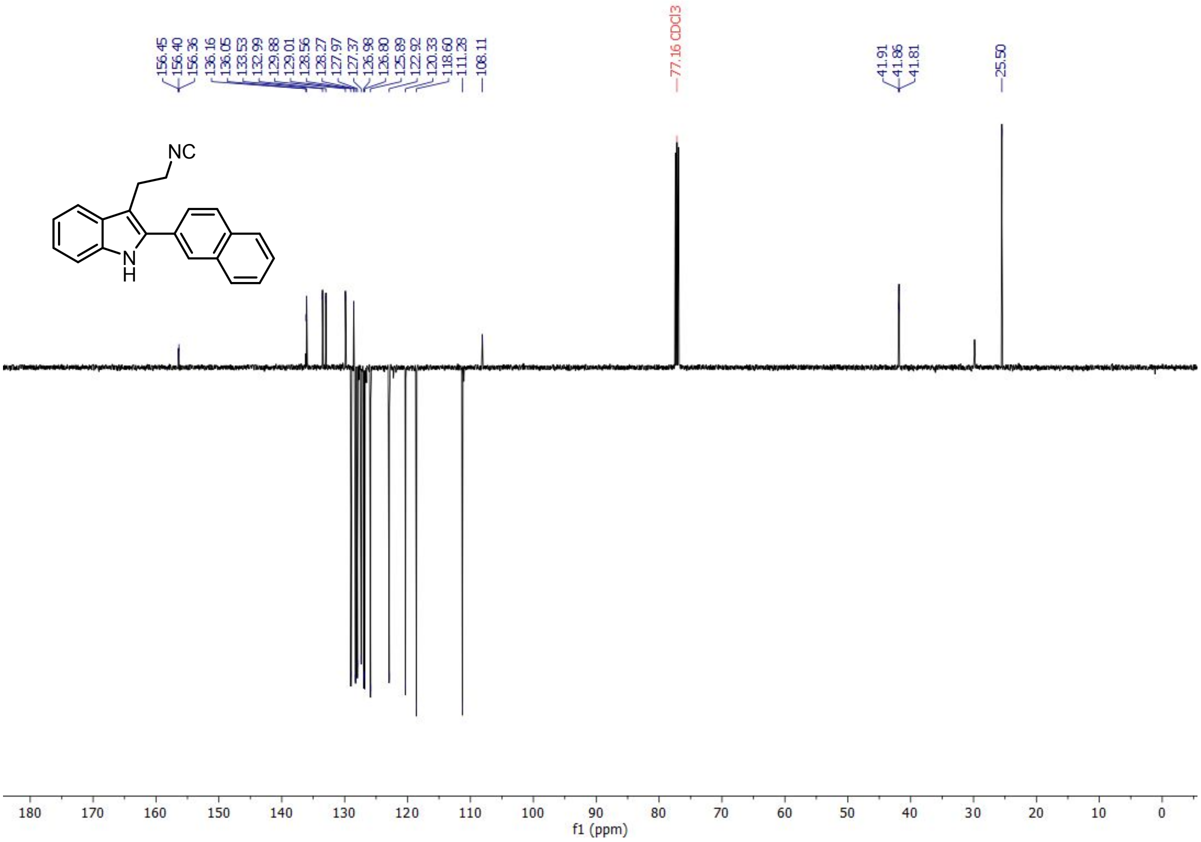

^{1}H NMR 600 MHz, $CDCl_3$ **1p**

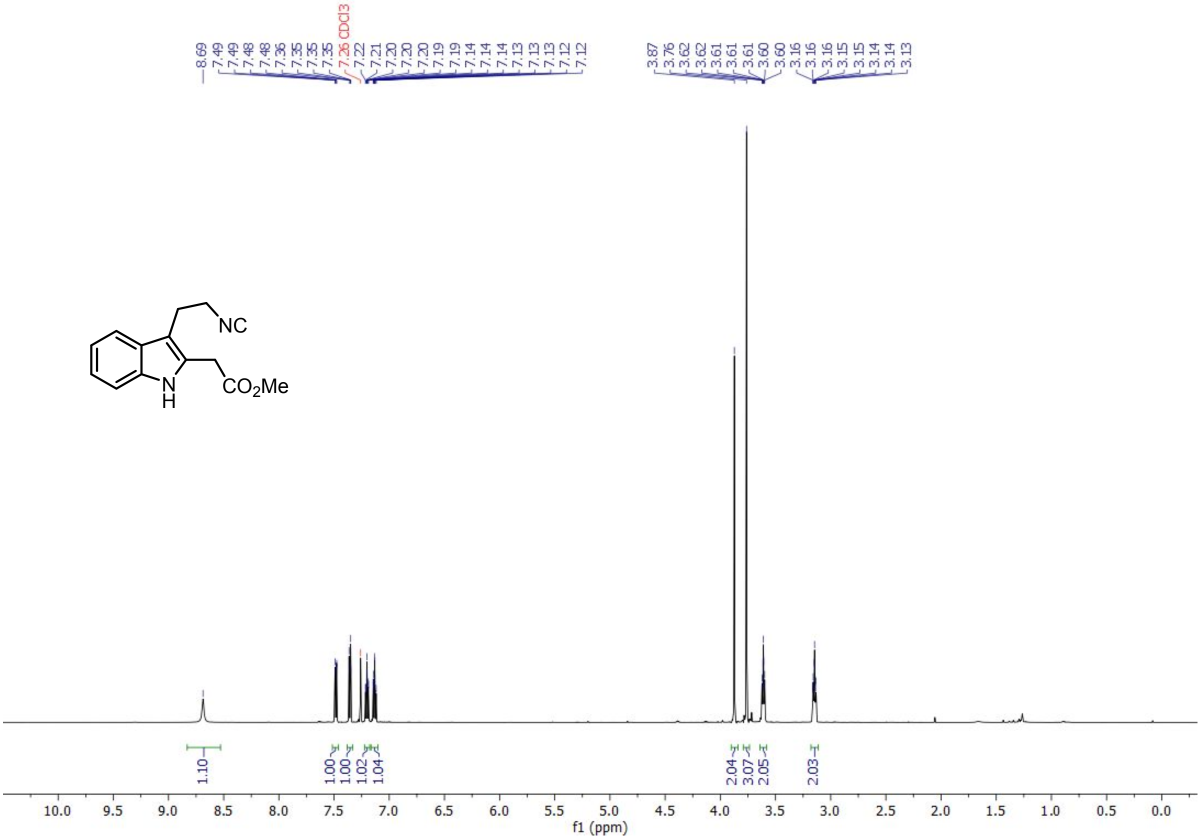

$^{13}C\{^1H\}$ NMR 150 MHz, $CDCl_3$ **1p**

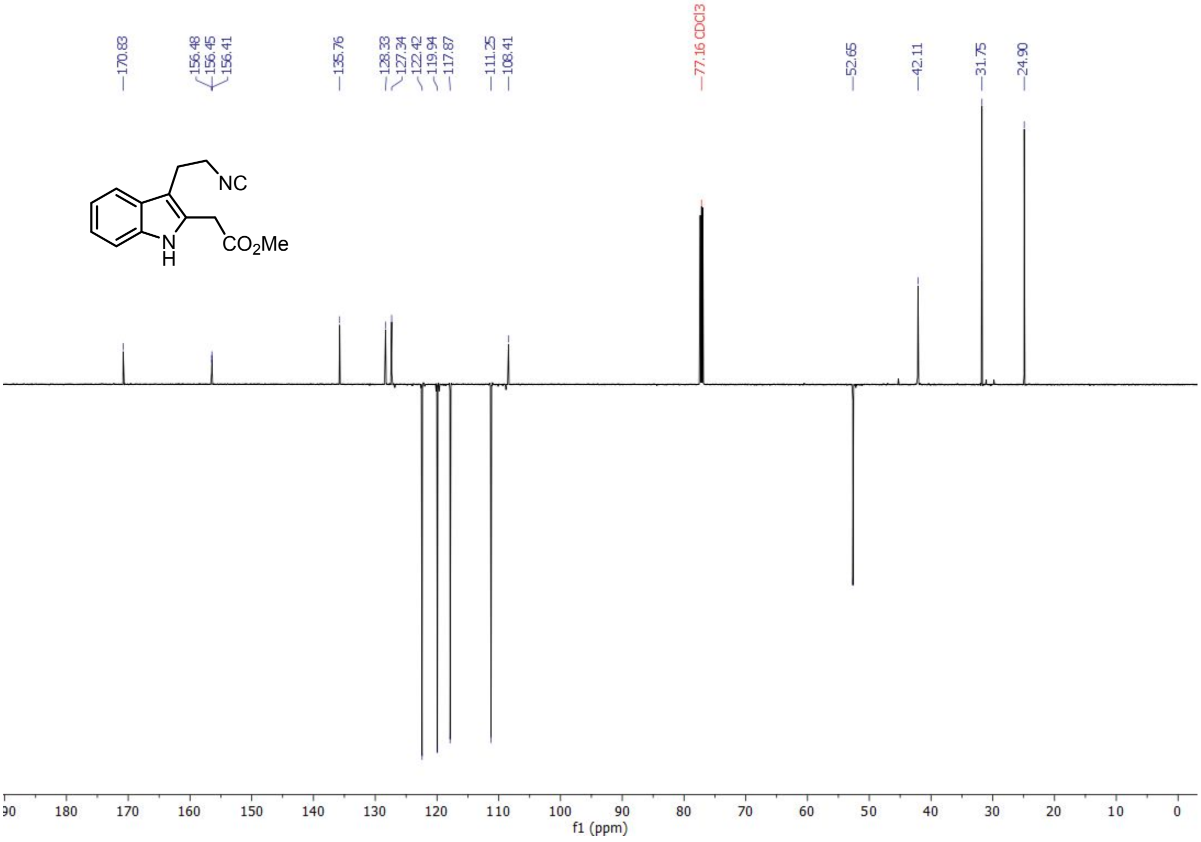

^{1}H NMR 500 MHz, $CDCl_3$ **1x**

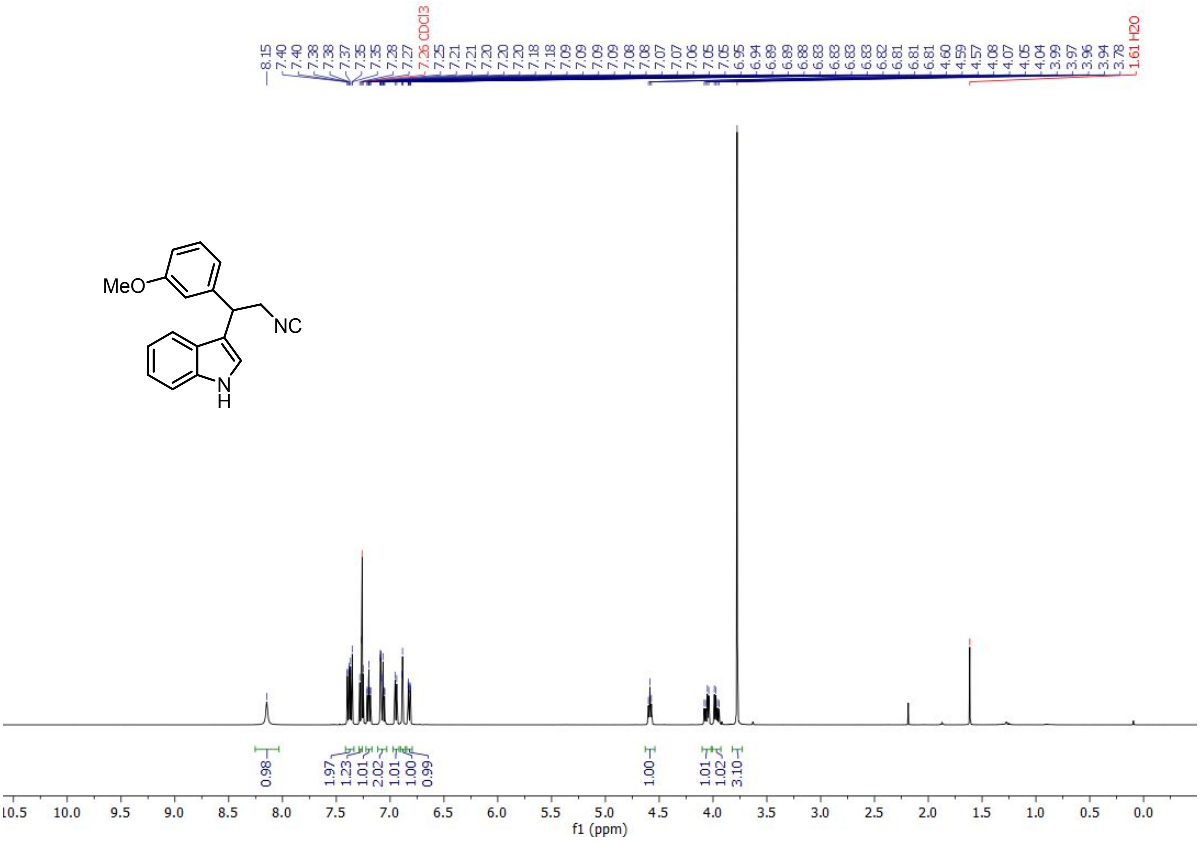

$^{13}C\{^1H\}$ NMR 126 MHz, $CDCl_3$ **1x**

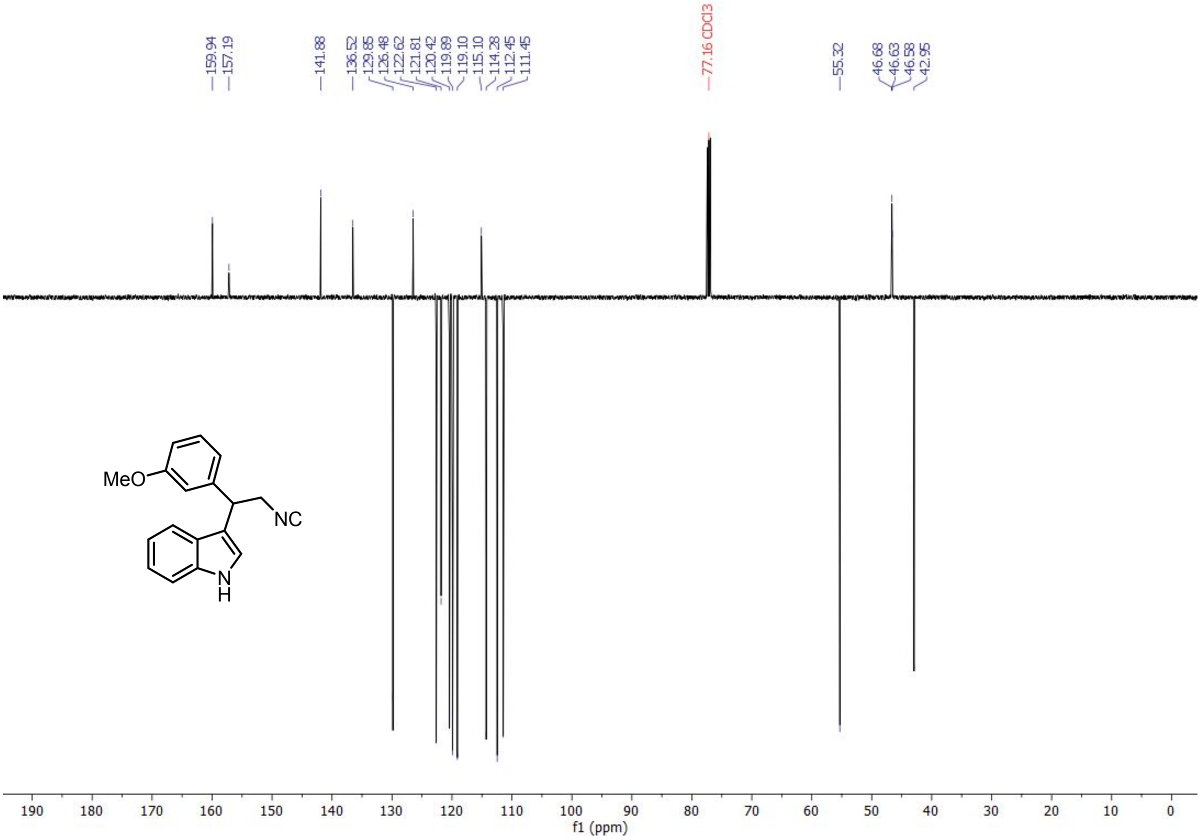

^{1}H NMR 600 MHz, $CDCl_3$ **23a**

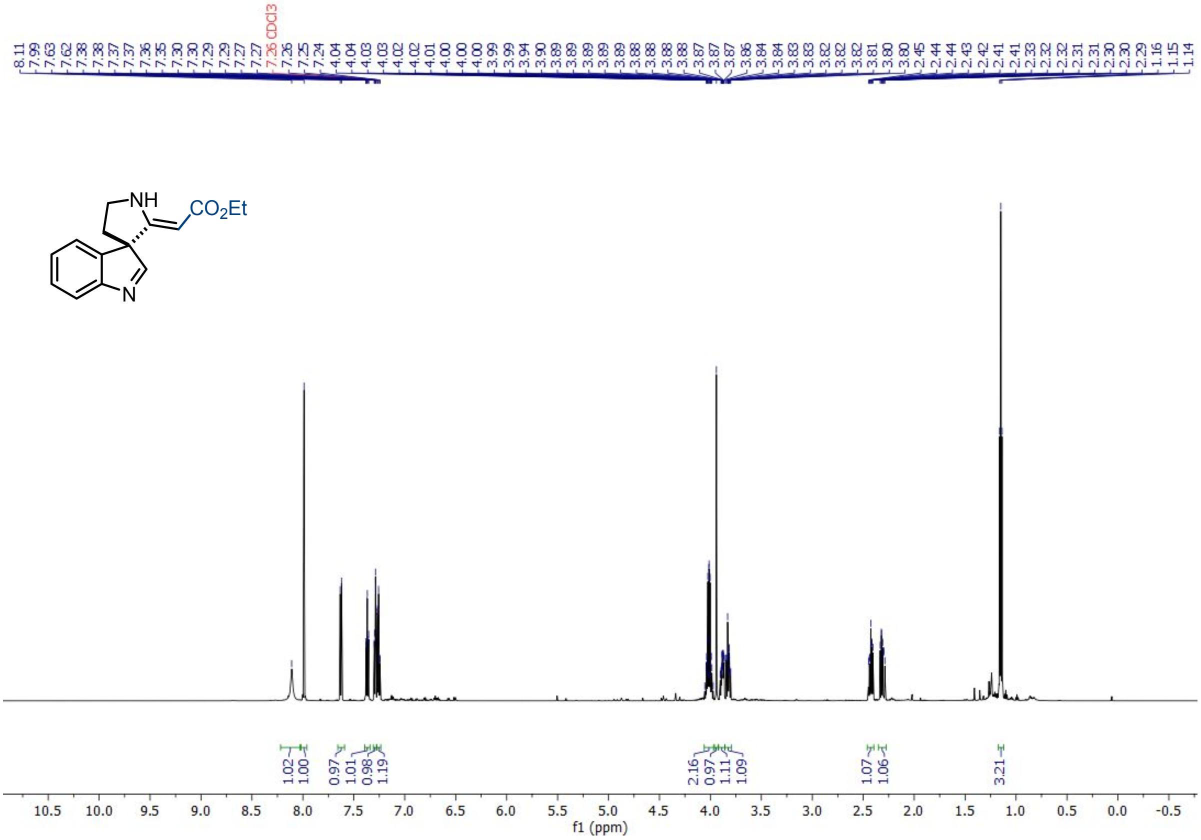

^{13}C{^{1}H} NMR 150 MHz, $CDCl_3$ **23a**

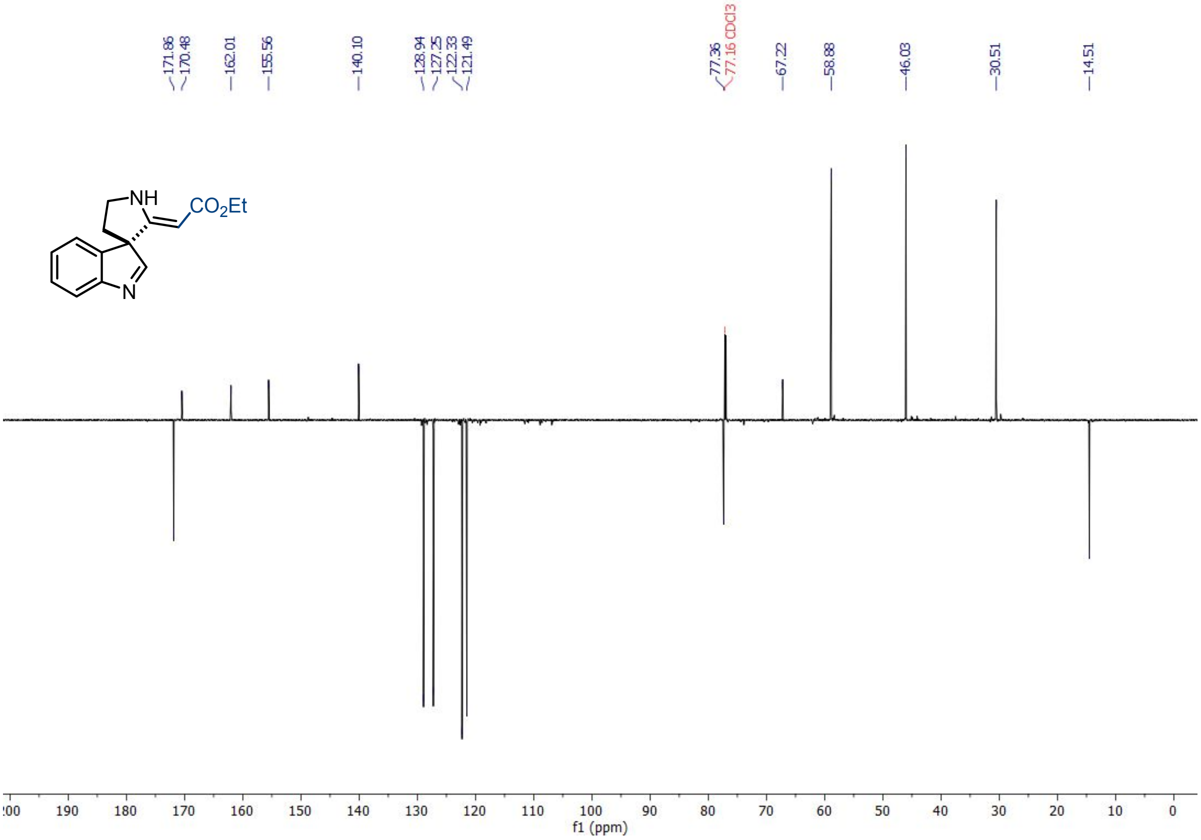

^{1}H NMR 500 MHz, $CDCl_3$ **23b**

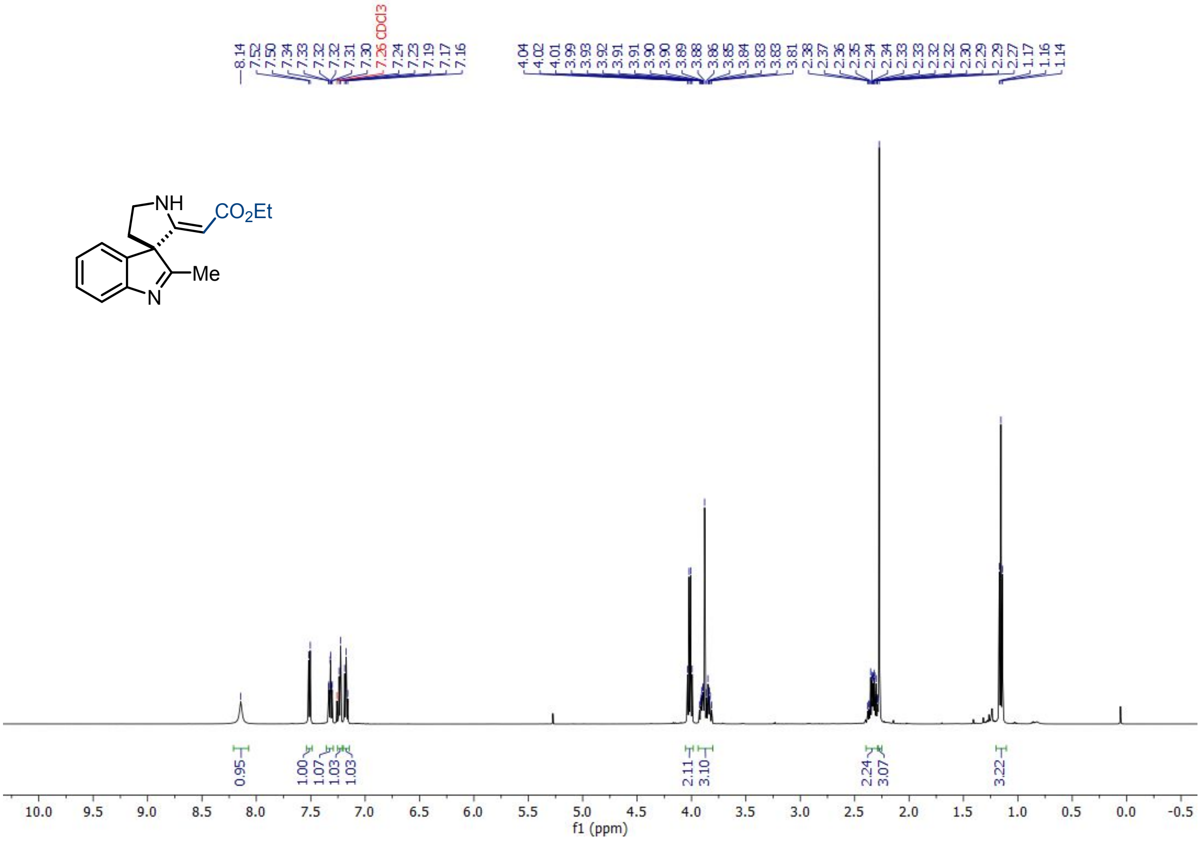

$^{13}C\{^1H\}$ NMR 126 MHz, $CDCl_3$ **23b**

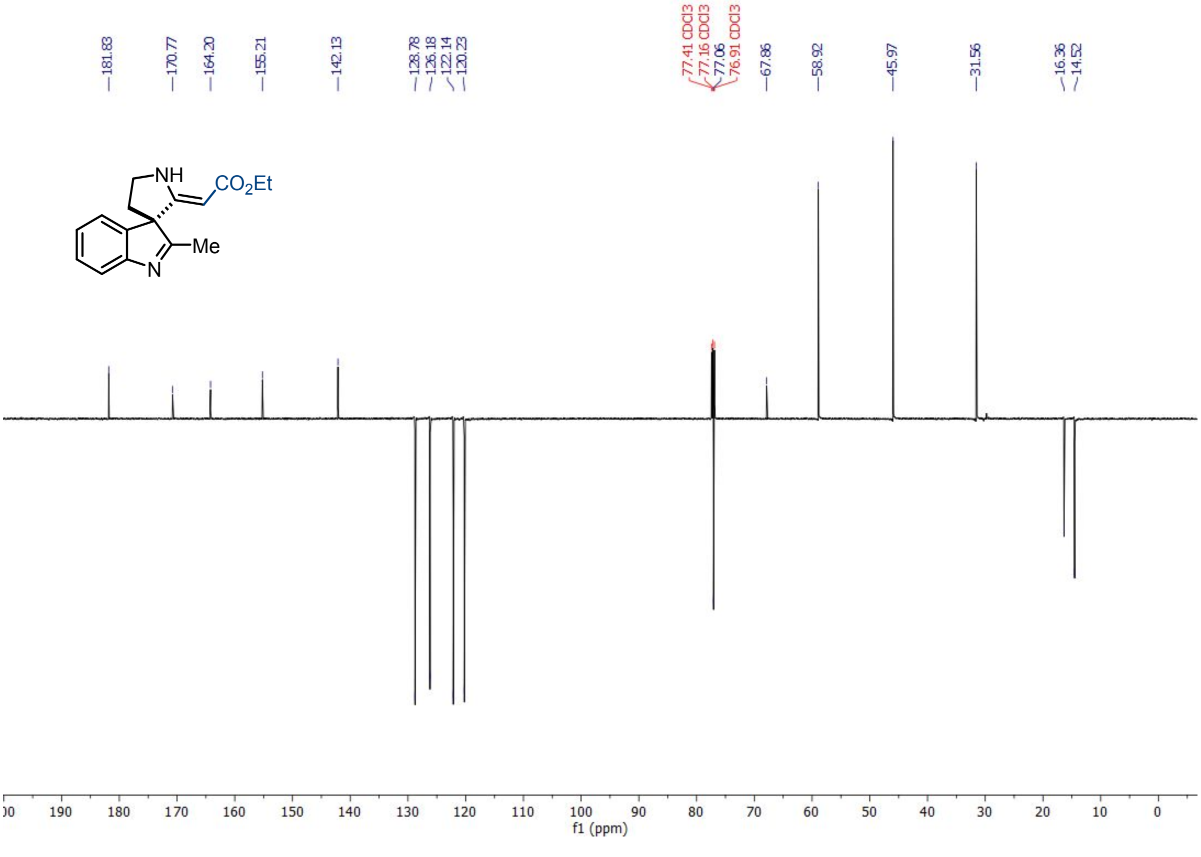

^{1}H NMR 500 MHz, $CDCl_3$ **23c**

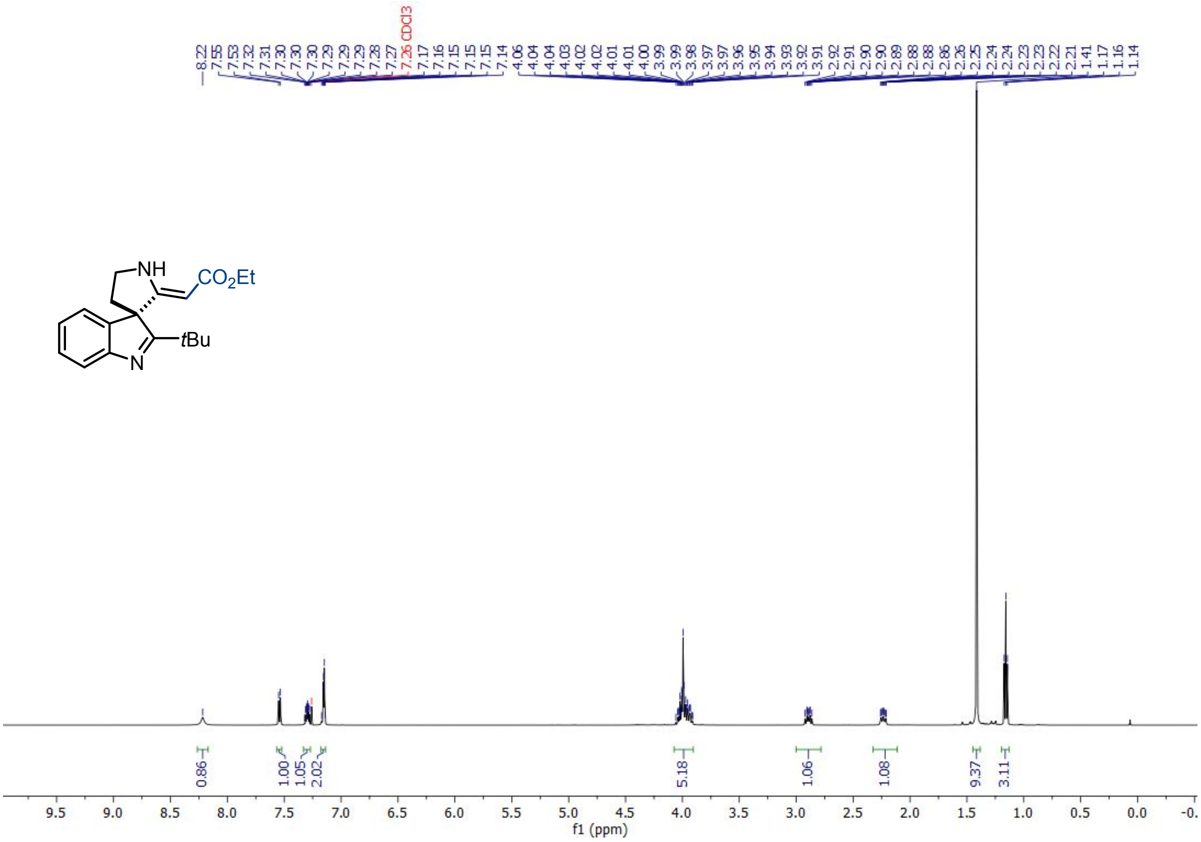

^{13}C{^{1}H} NMR 126 MHz, $CDCl_3$ **23c**

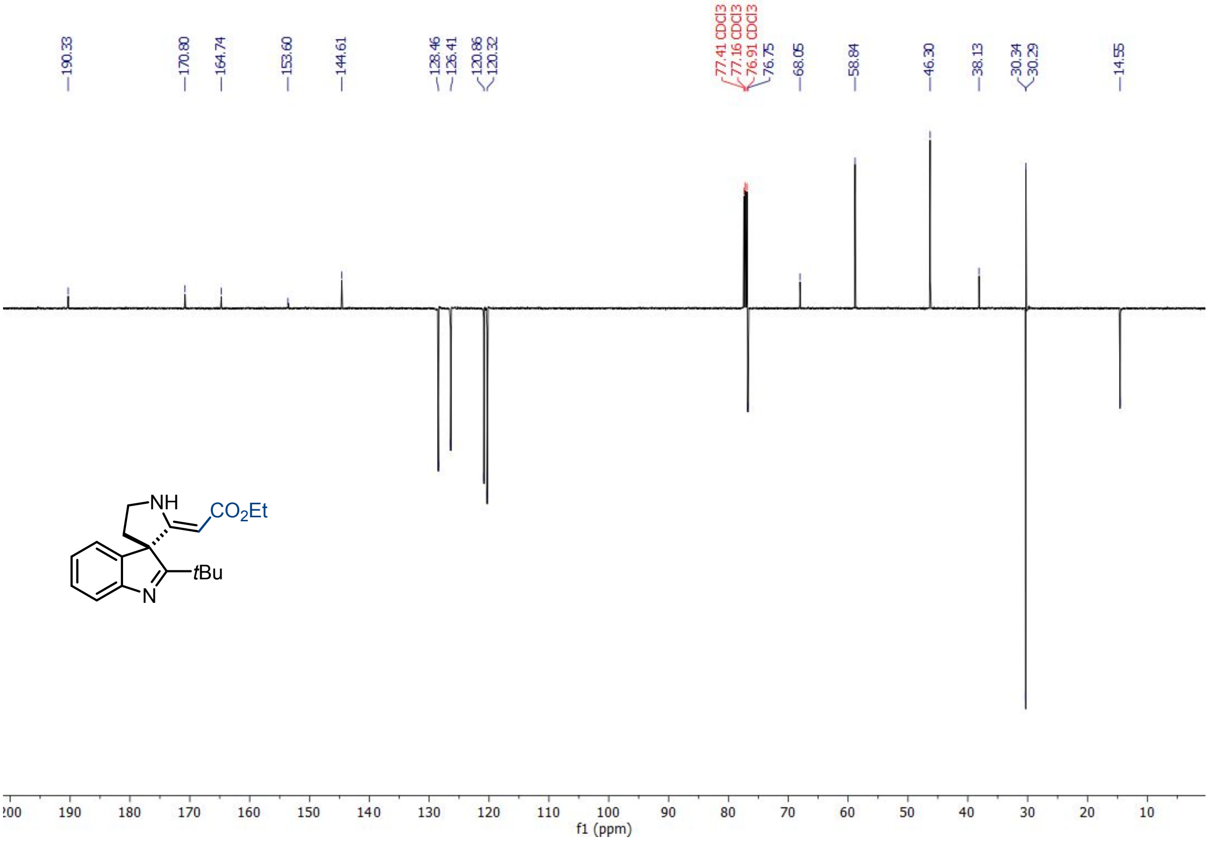

^{1}H NMR 500 MHz, $CDCl_3$ **23d**

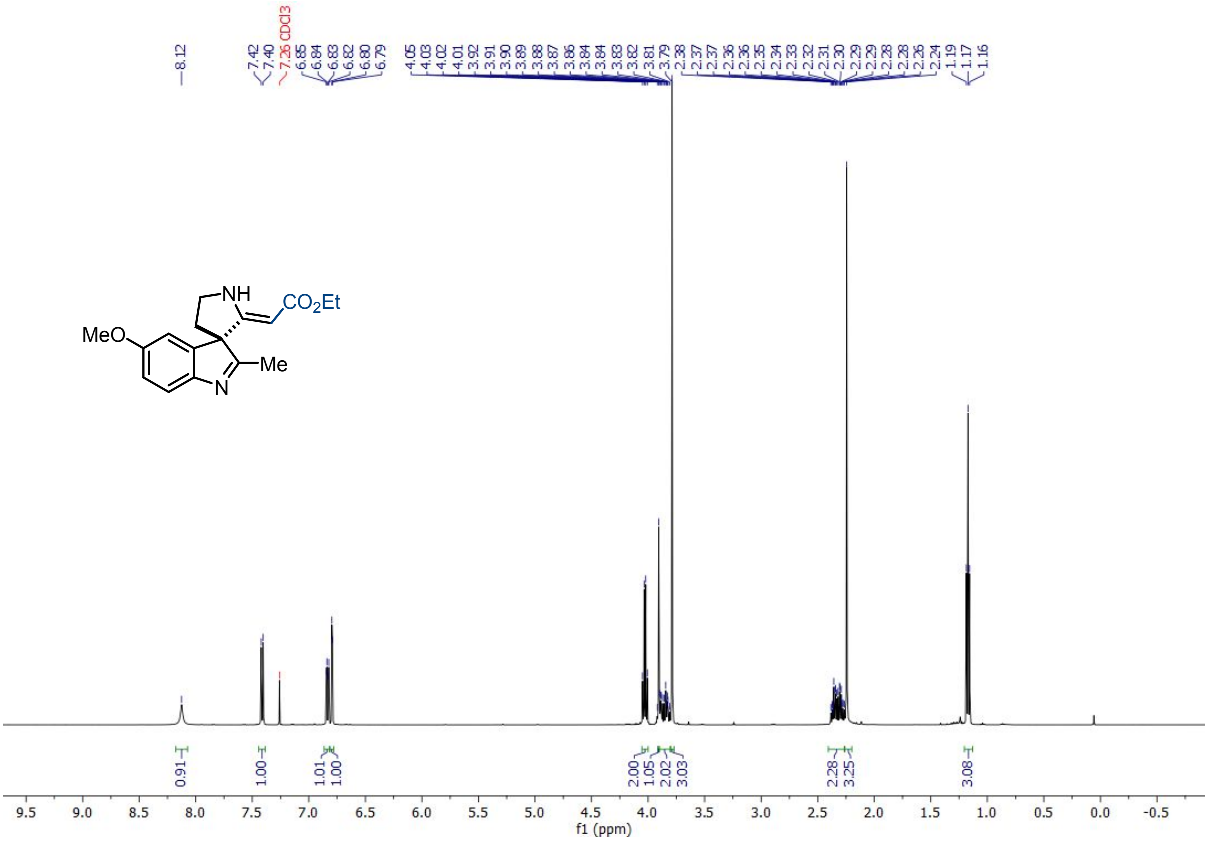

^{13}C{^{1}H} NMR 126 MHz, $CDCl_3$ **23d**

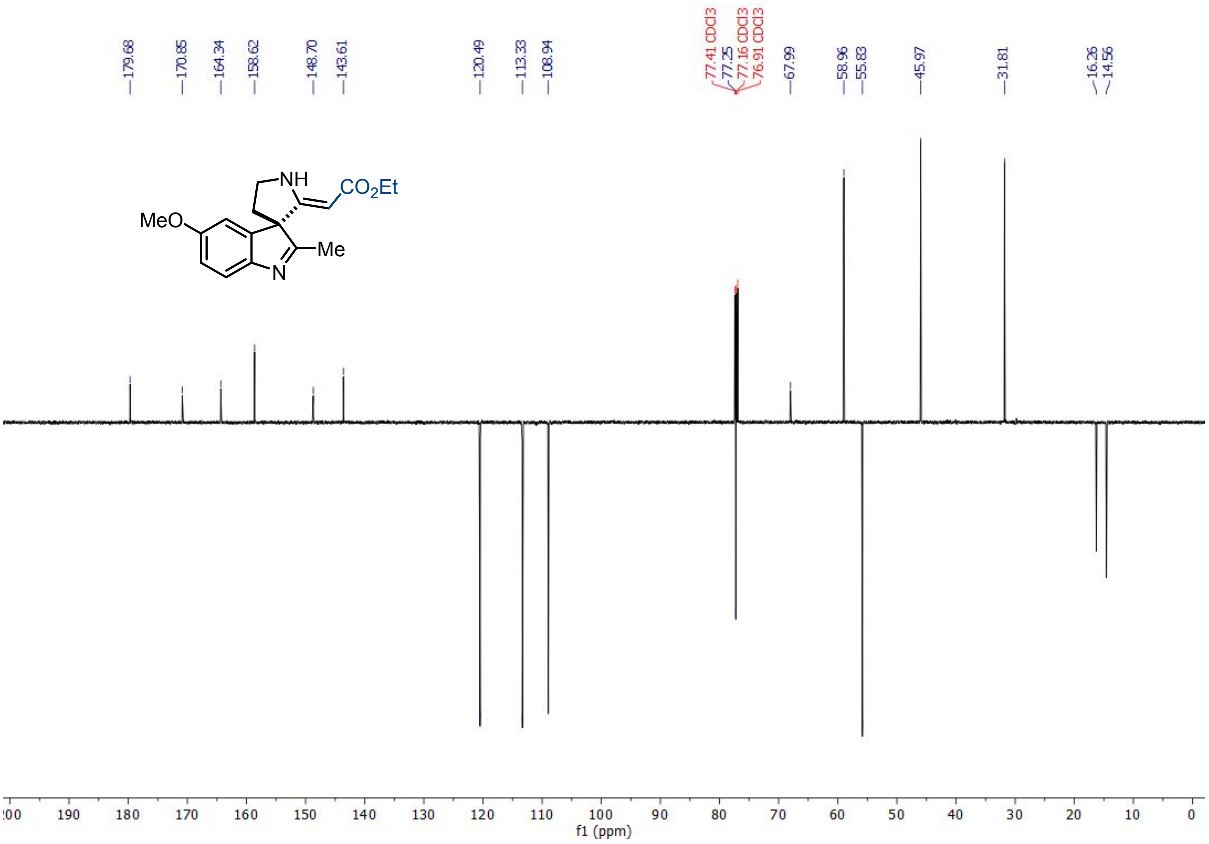

^{1}H NMR 500 MHz, $CDCl_3$ **23e**

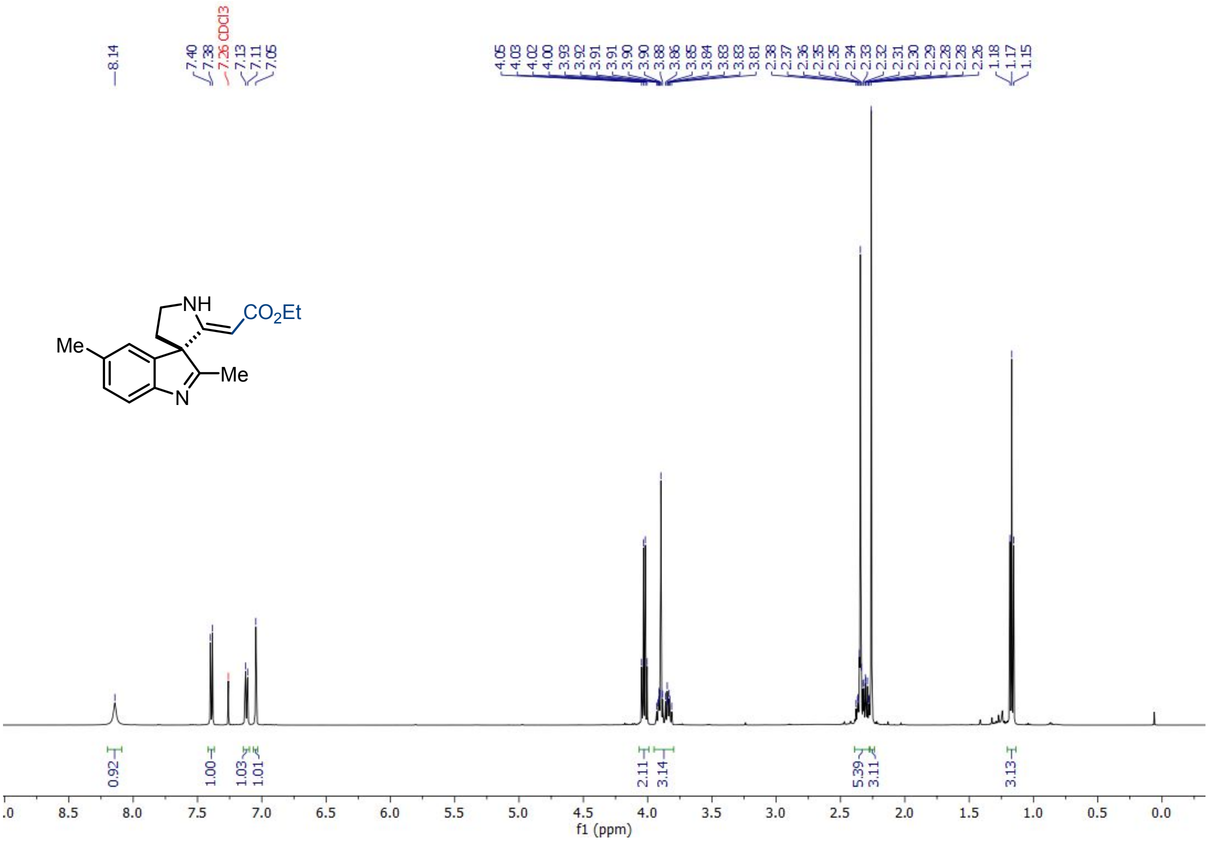

$^{13}C\{^{1}H\}$ NMR 126 MHz, $CDCl_3$ **23e**

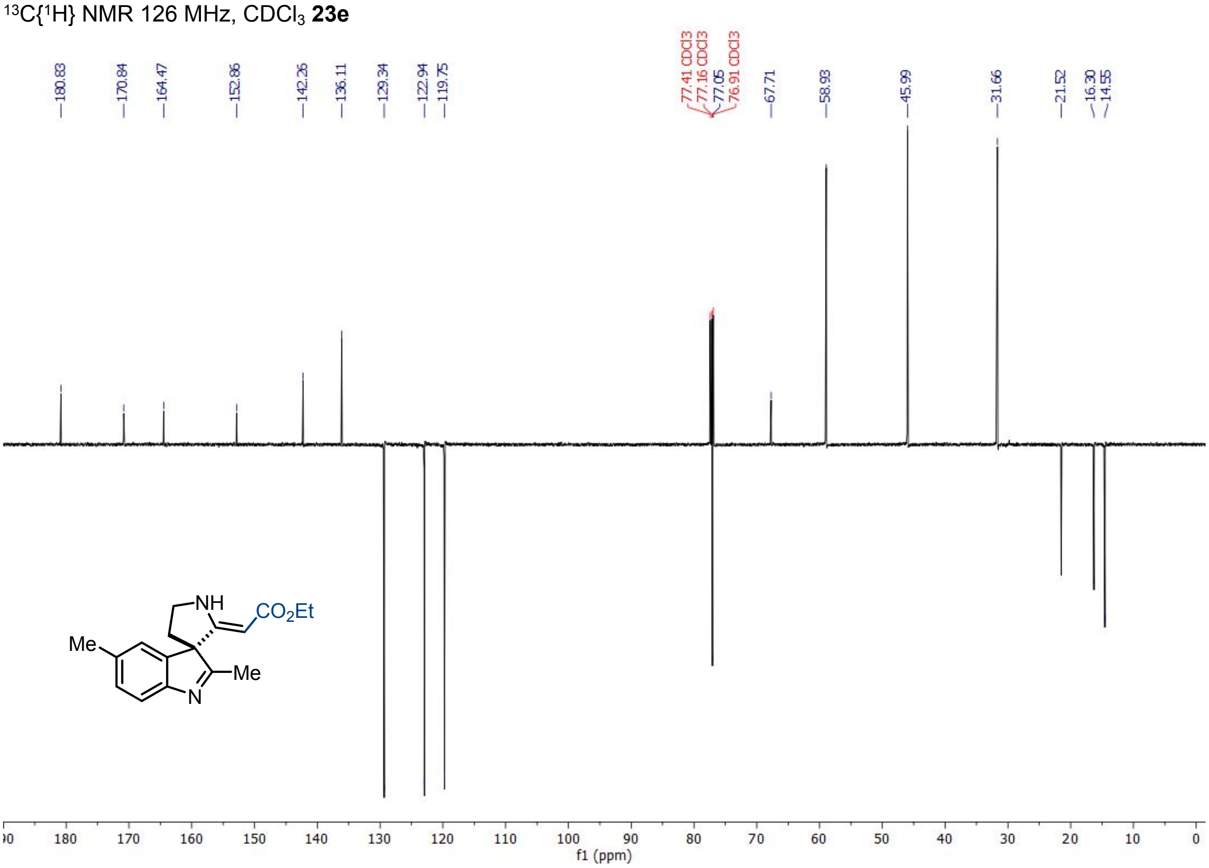

^{1}H NMR 500 MHz, $CDCl_3$ **23f**

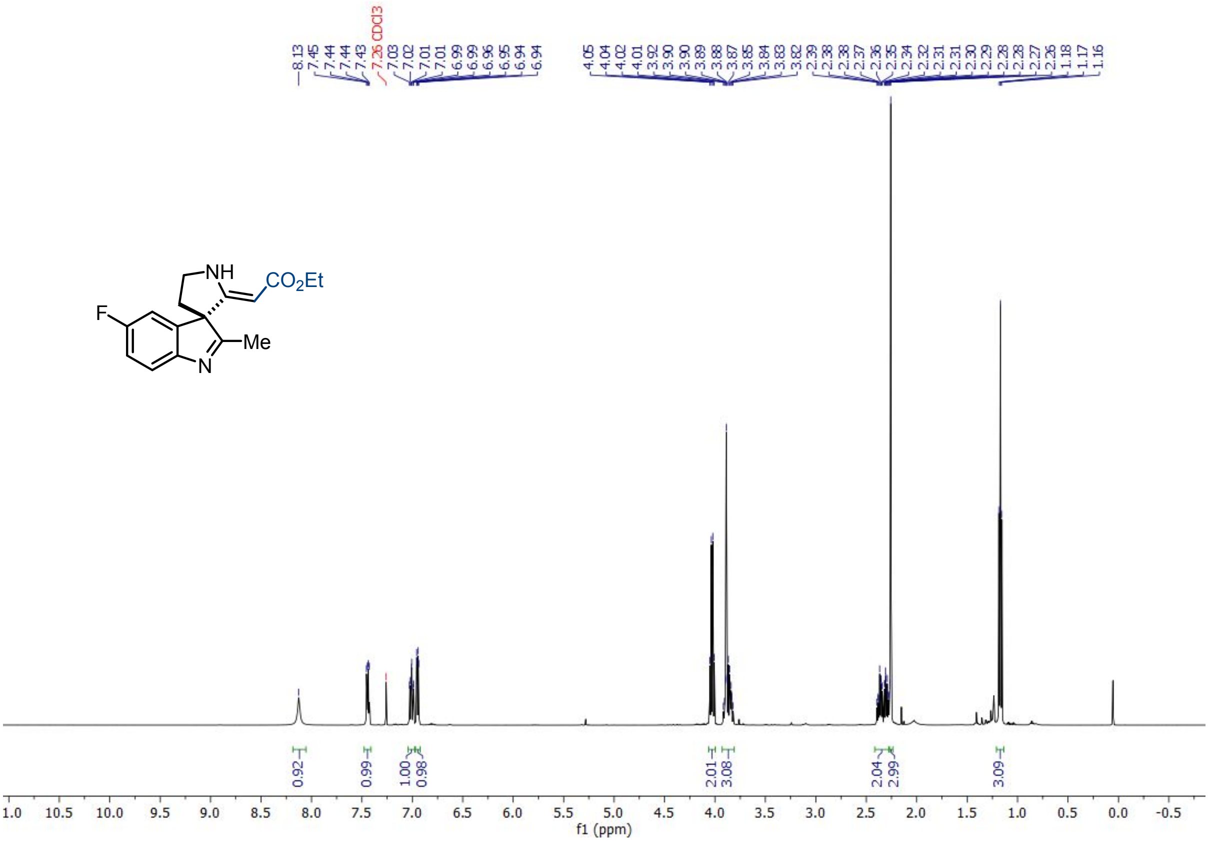

^{13}C{^{1}H} NMR 126 MHz, $CDCl_3$ **23f**

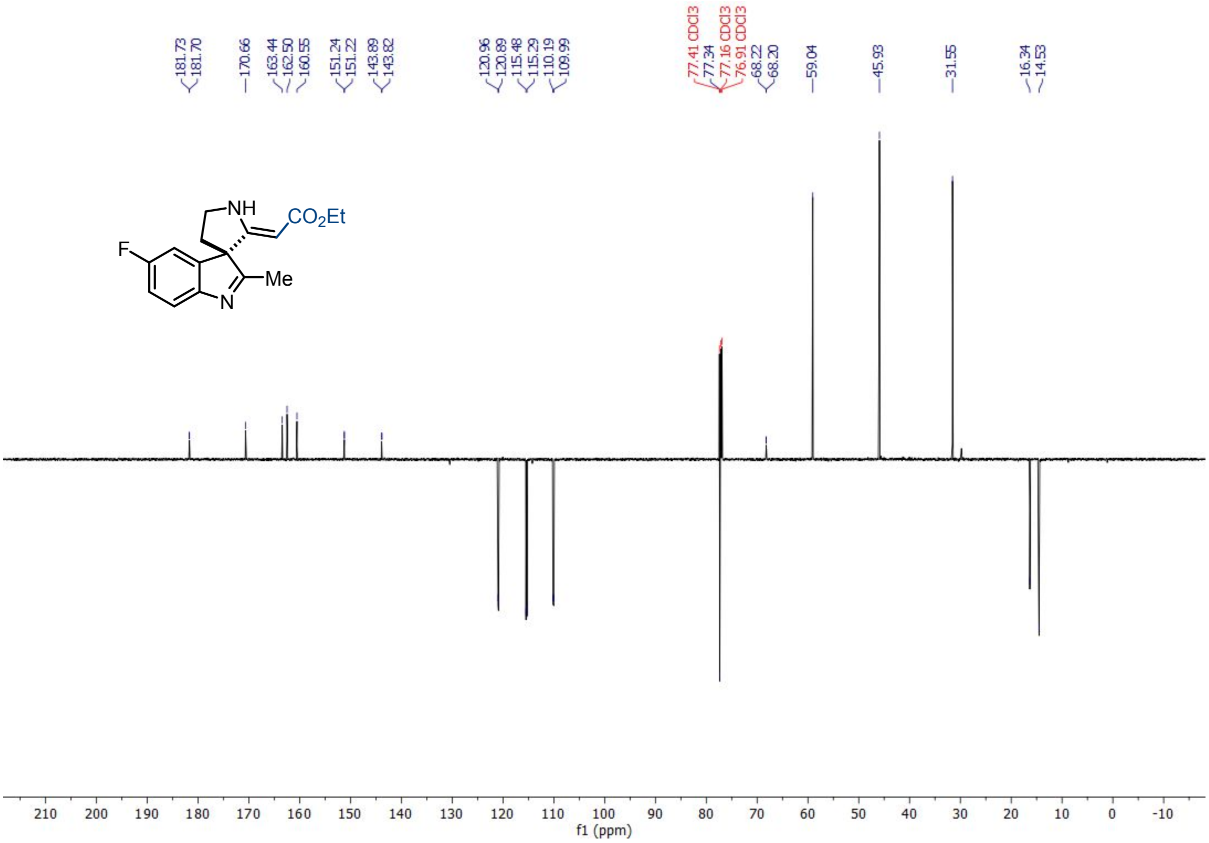

$^{19}F\{^1H\}$ NMR 470.4 MHz, $CDCl_3$ **23f**

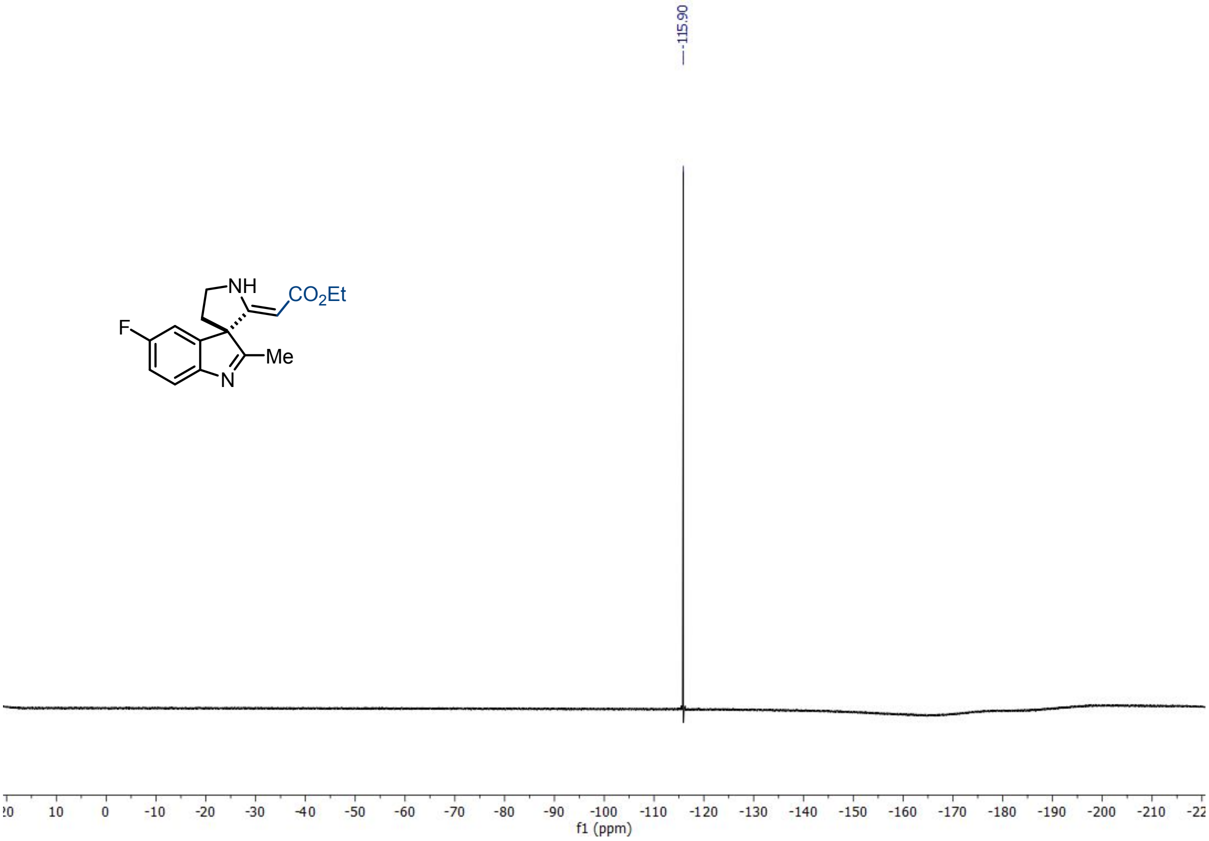

^{1}H NMR 500 MHz, $CDCl_3$ **23g**

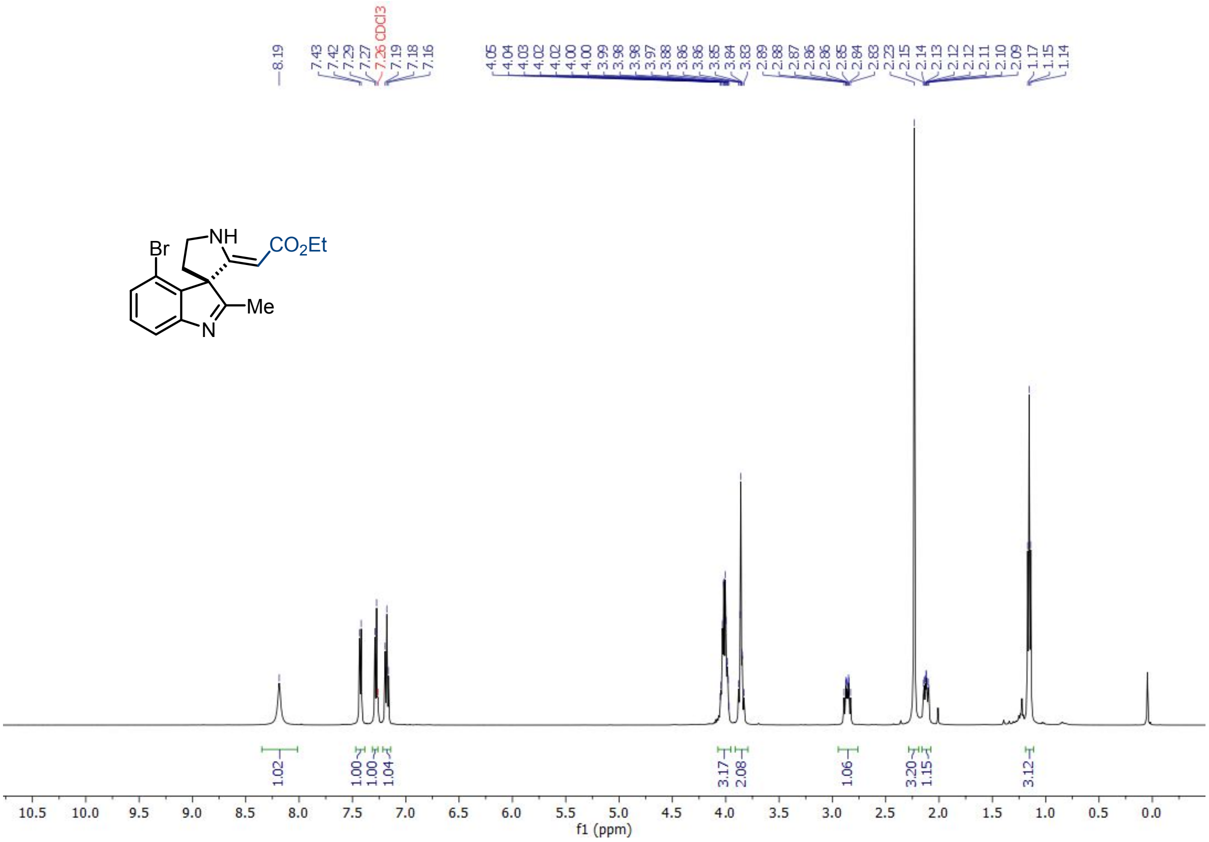

$^{13}C\{^{1}H\}$ NMR 126 MHz, $CDCl_3$ **23g**

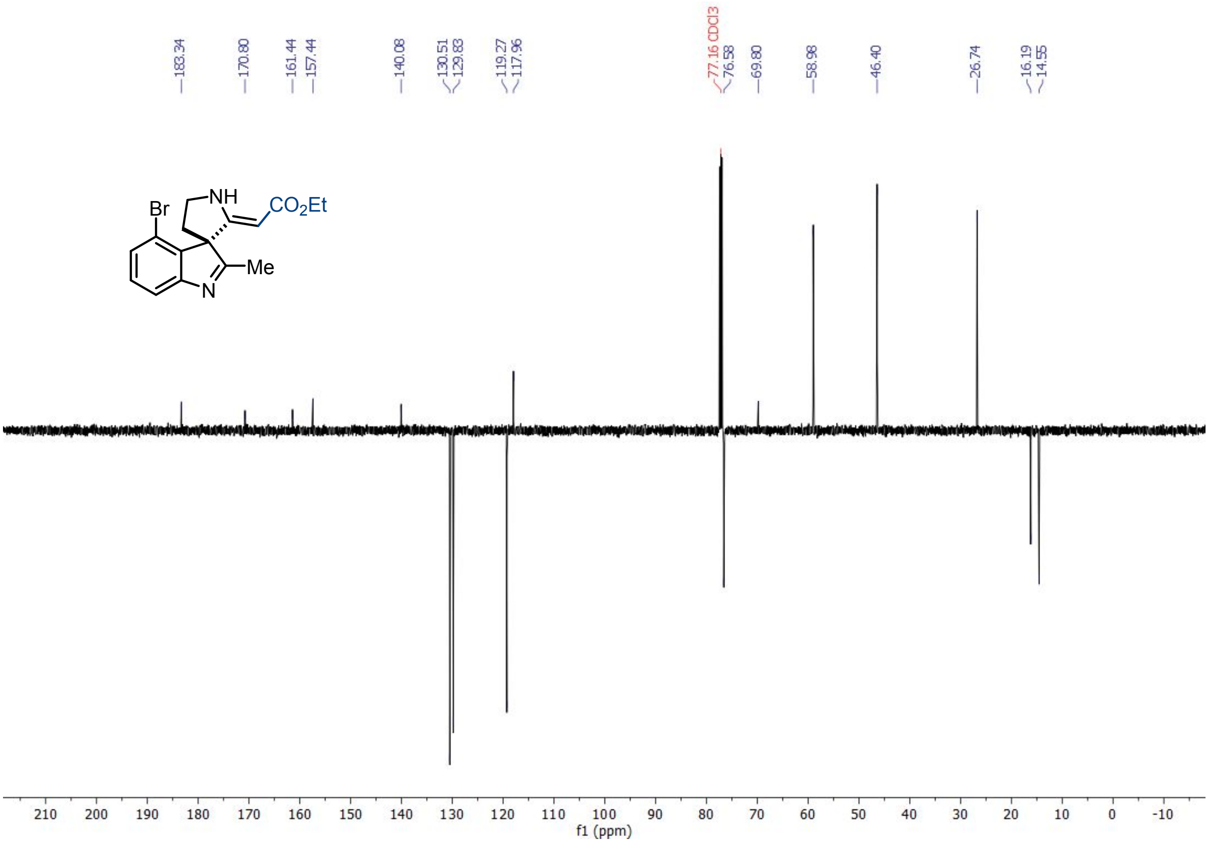

^{1}H NMR 500 MHz, $CDCl_3$ **23h**

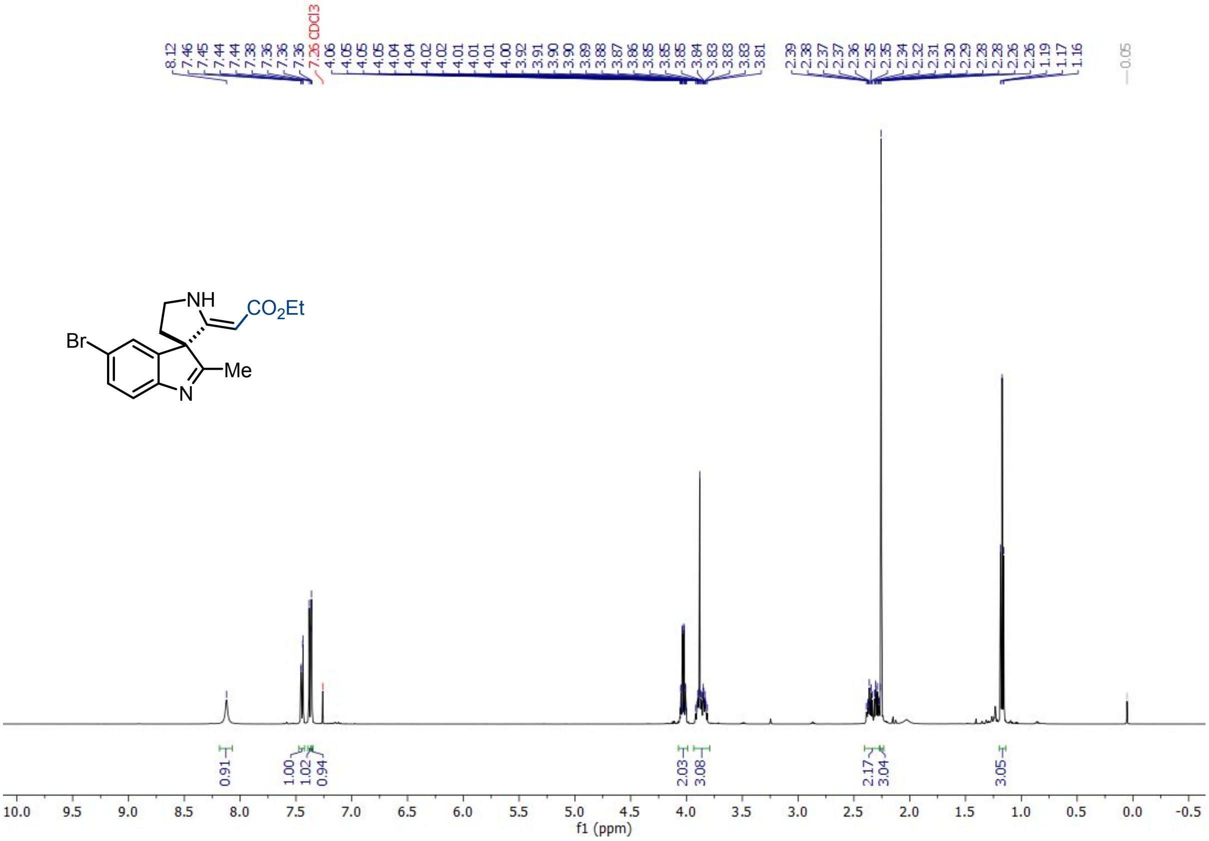

^{13}C{^{1}H} NMR 126 MHz, $CDCl_3$ **23h**

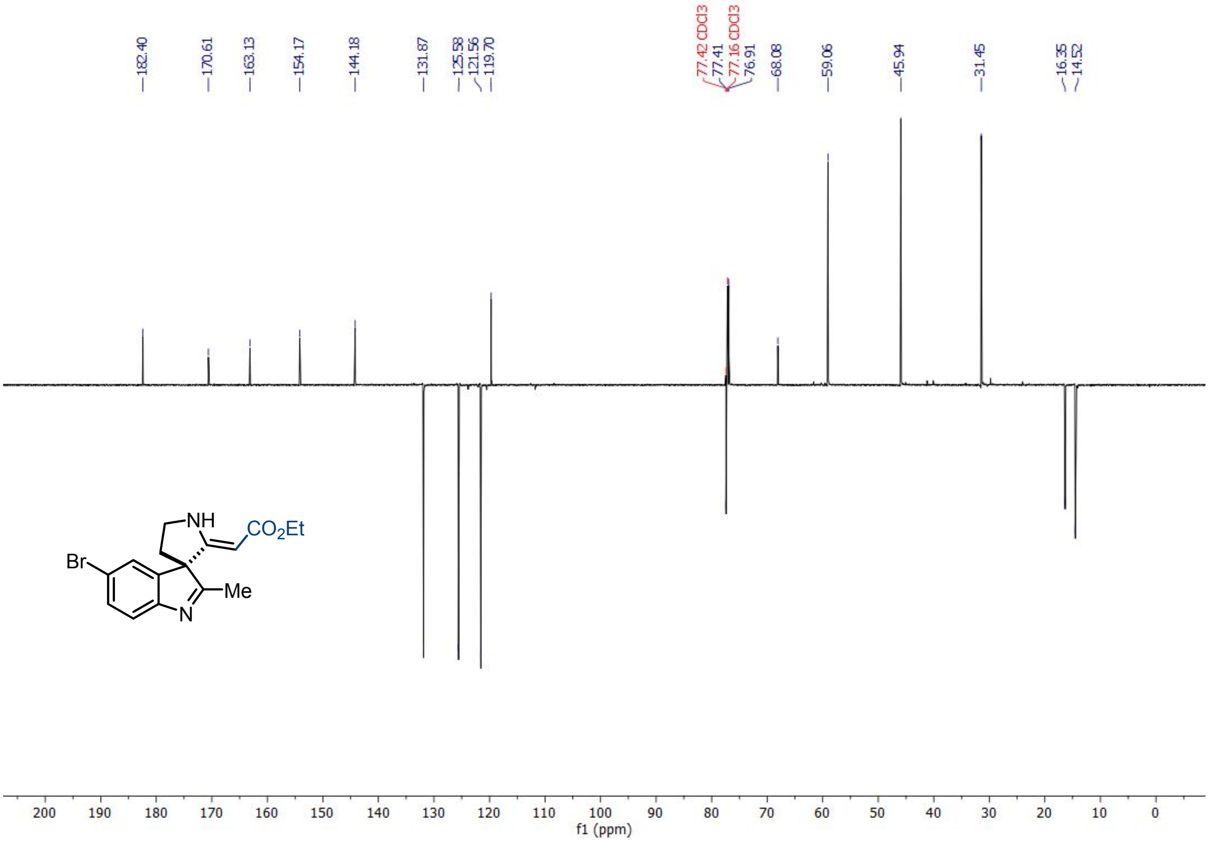

^{1}H NMR 500 MHz, $CDCl_3$ **23i**

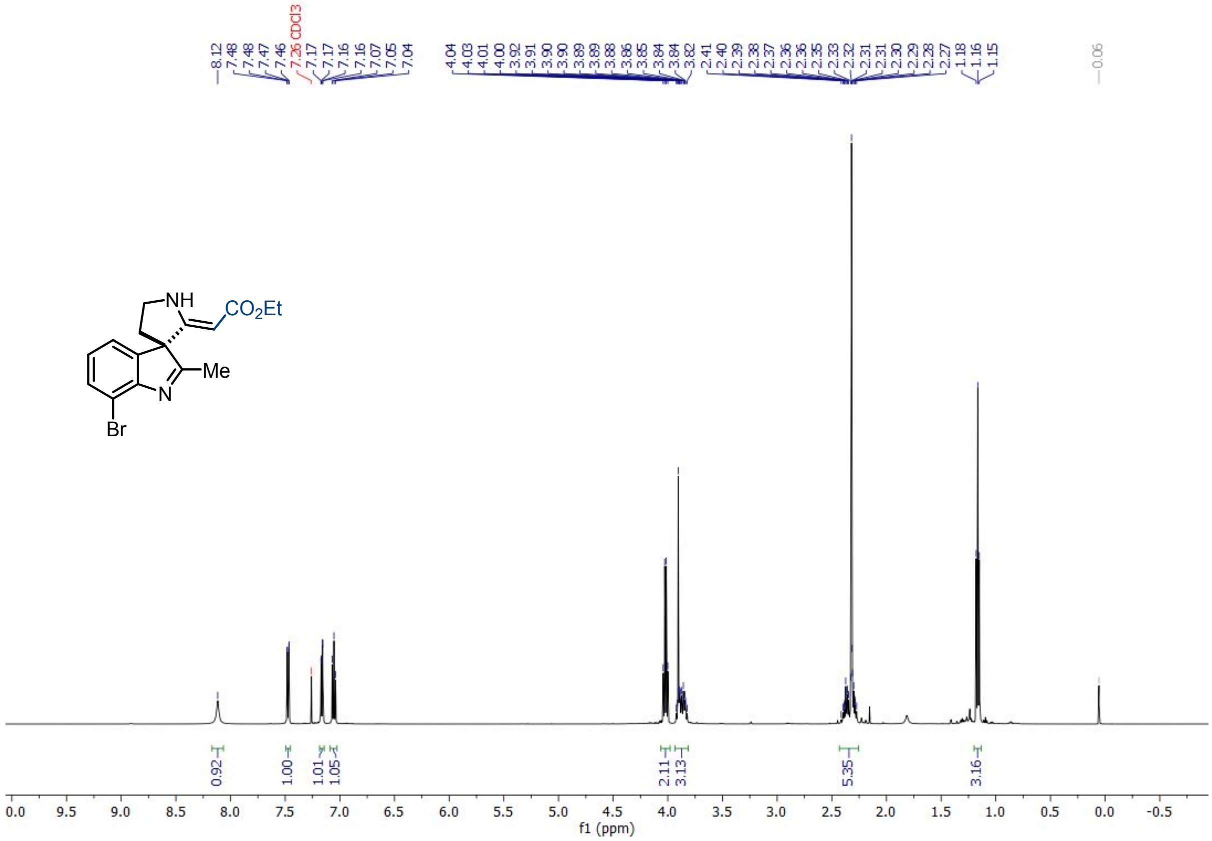

$^{13}C\{^{1}H\}$ NMR 126 MHz, $CDCl_3$ **23i**

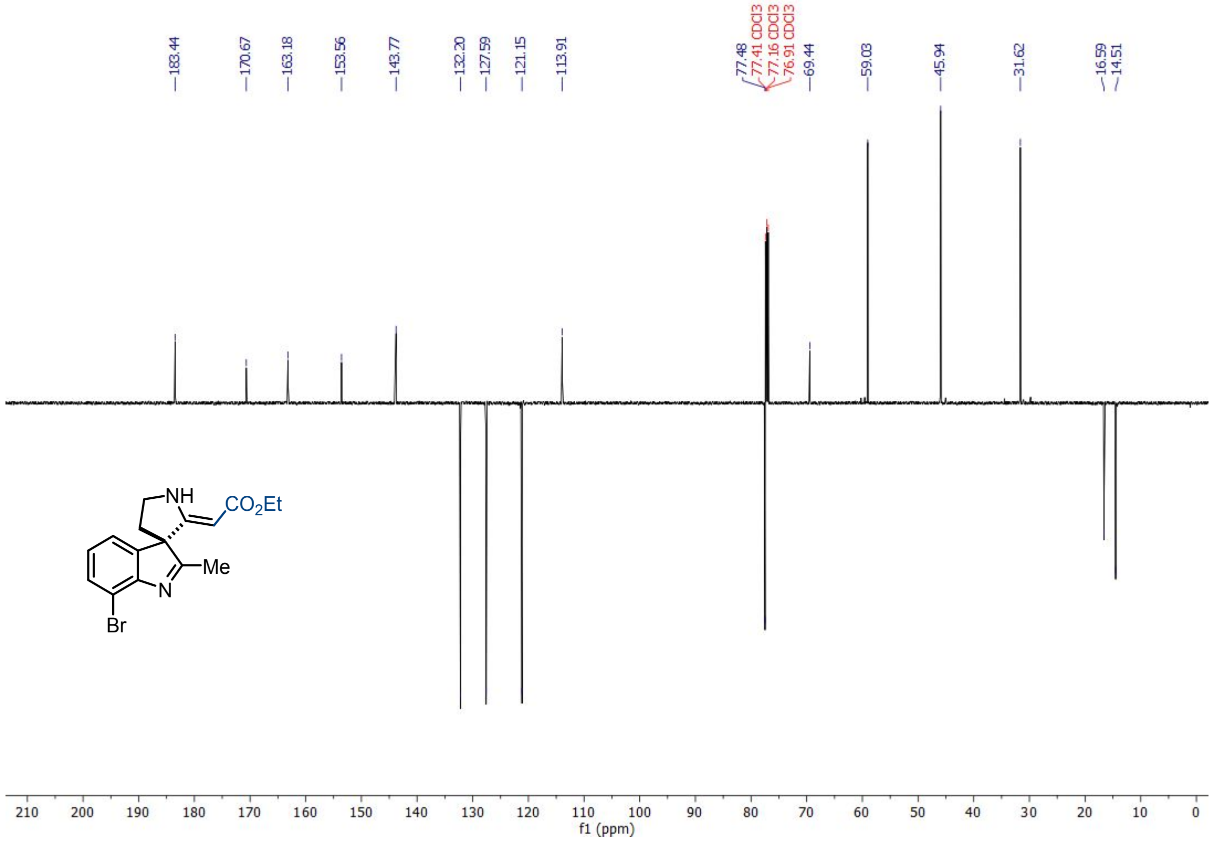

^{1}H NMR 500 MHz, $CDCl_3$ **23j**

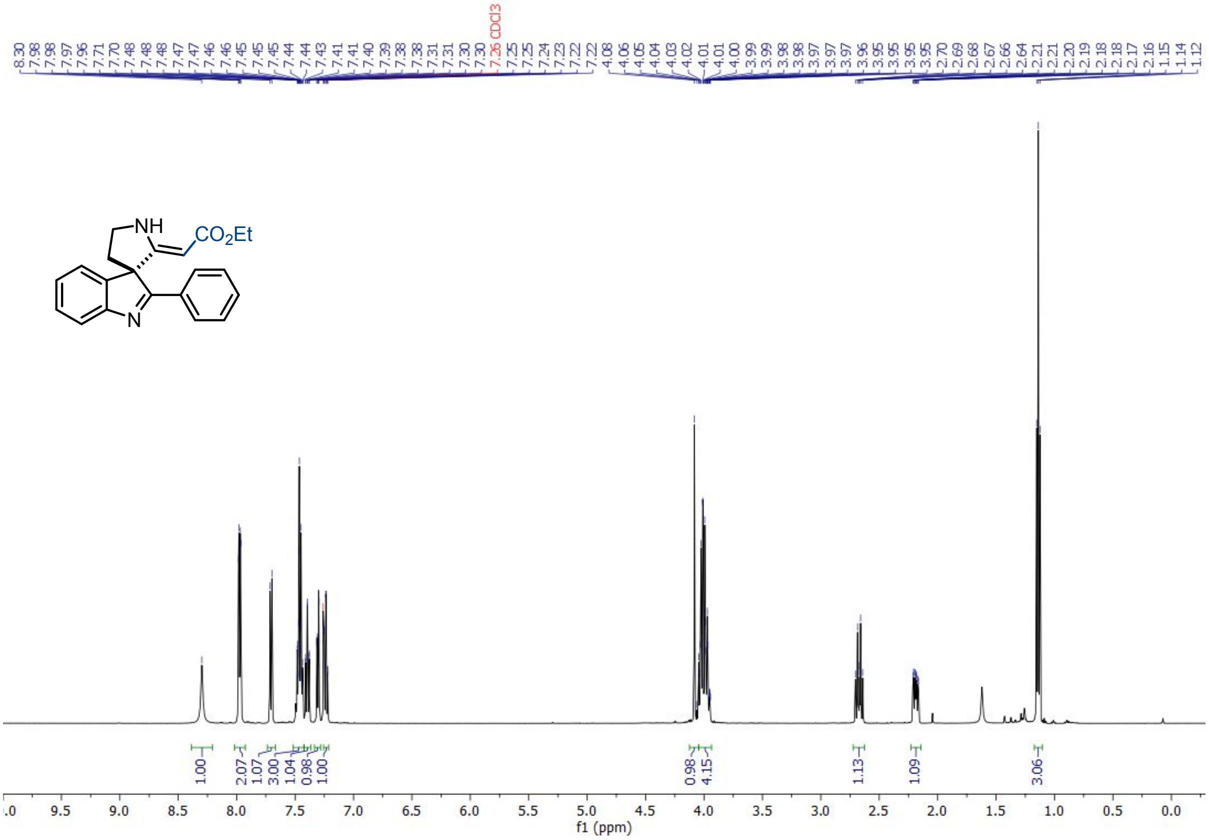

^{13}C{^{1}H} NMR 126 MHz, $CDCl_3$ **23j**

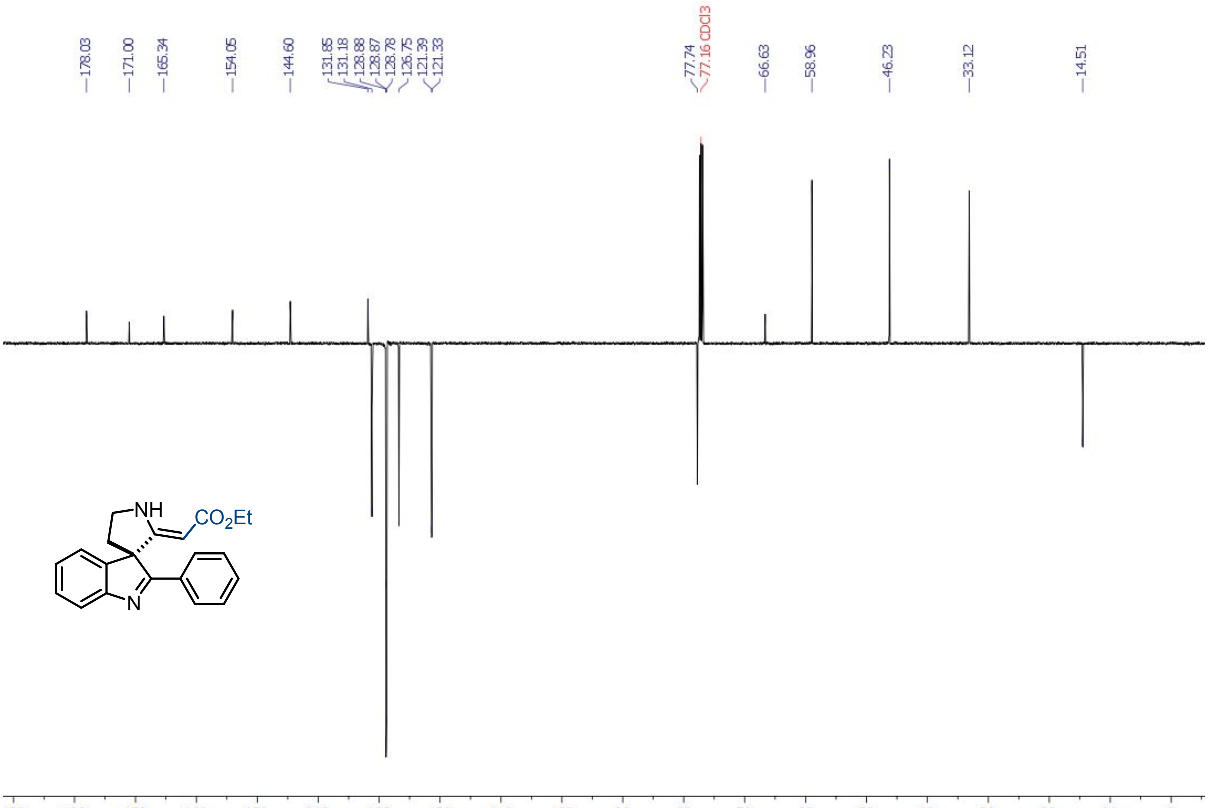

^1H NMR 500 MHz, $CDCl_3$ **23k**

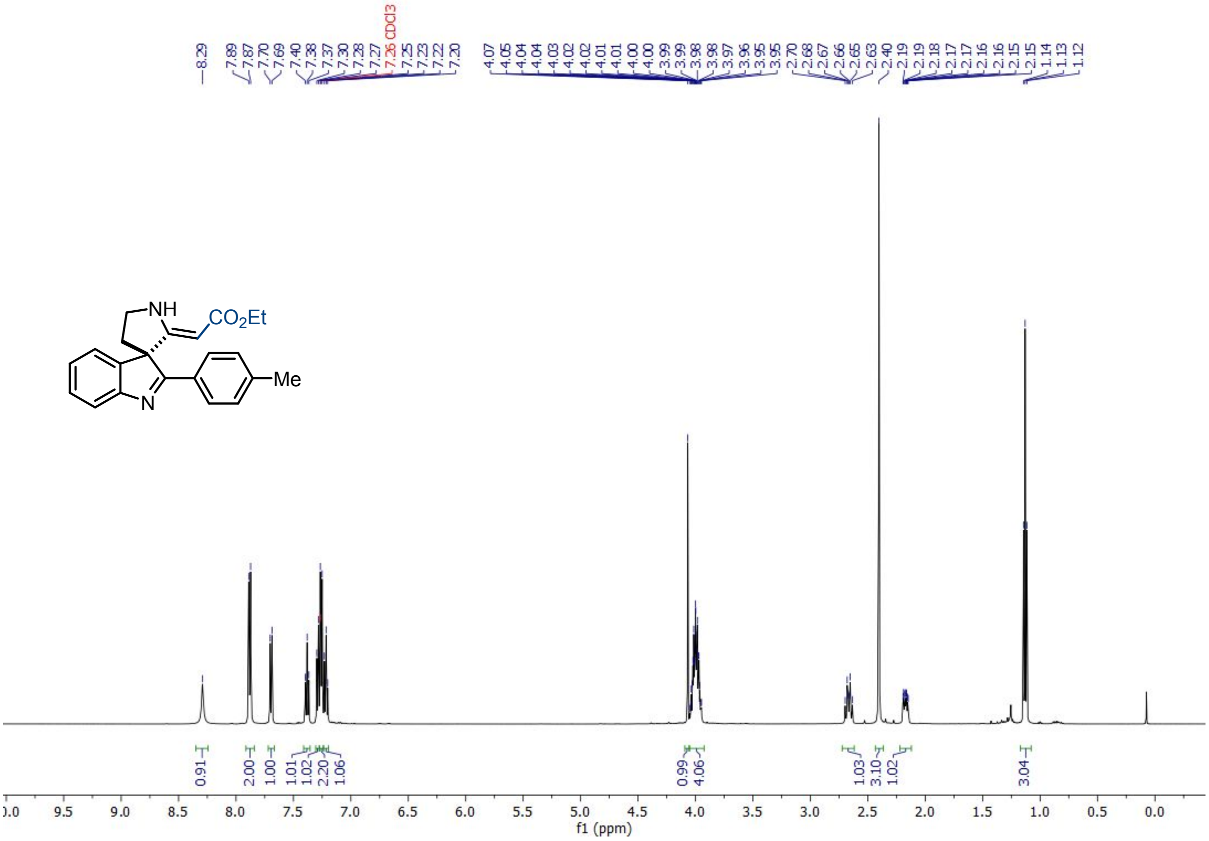

$^{13}C\{^1H\}$ NMR 126 MHz, $CDCl_3$ **23k**

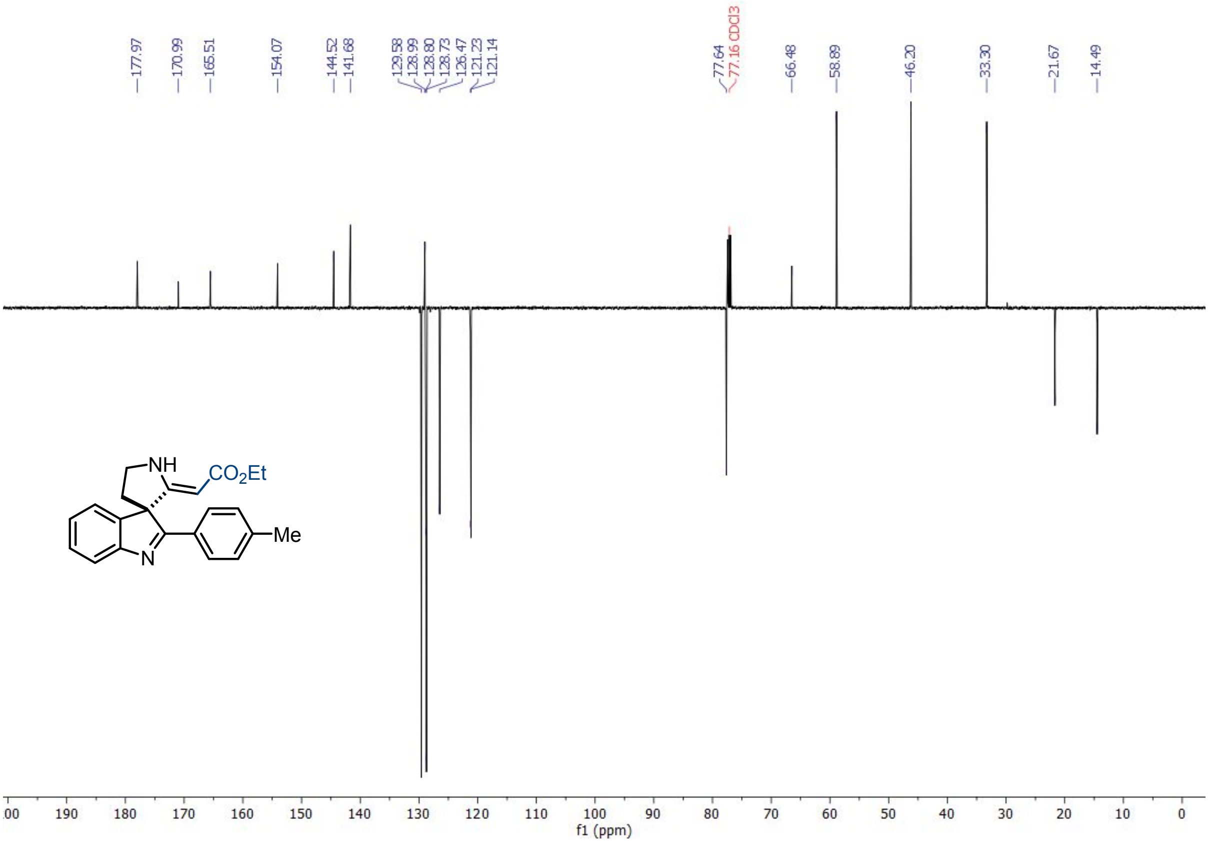

^{1}H NMR 500 MHz, $CDCl_3$ **23l**

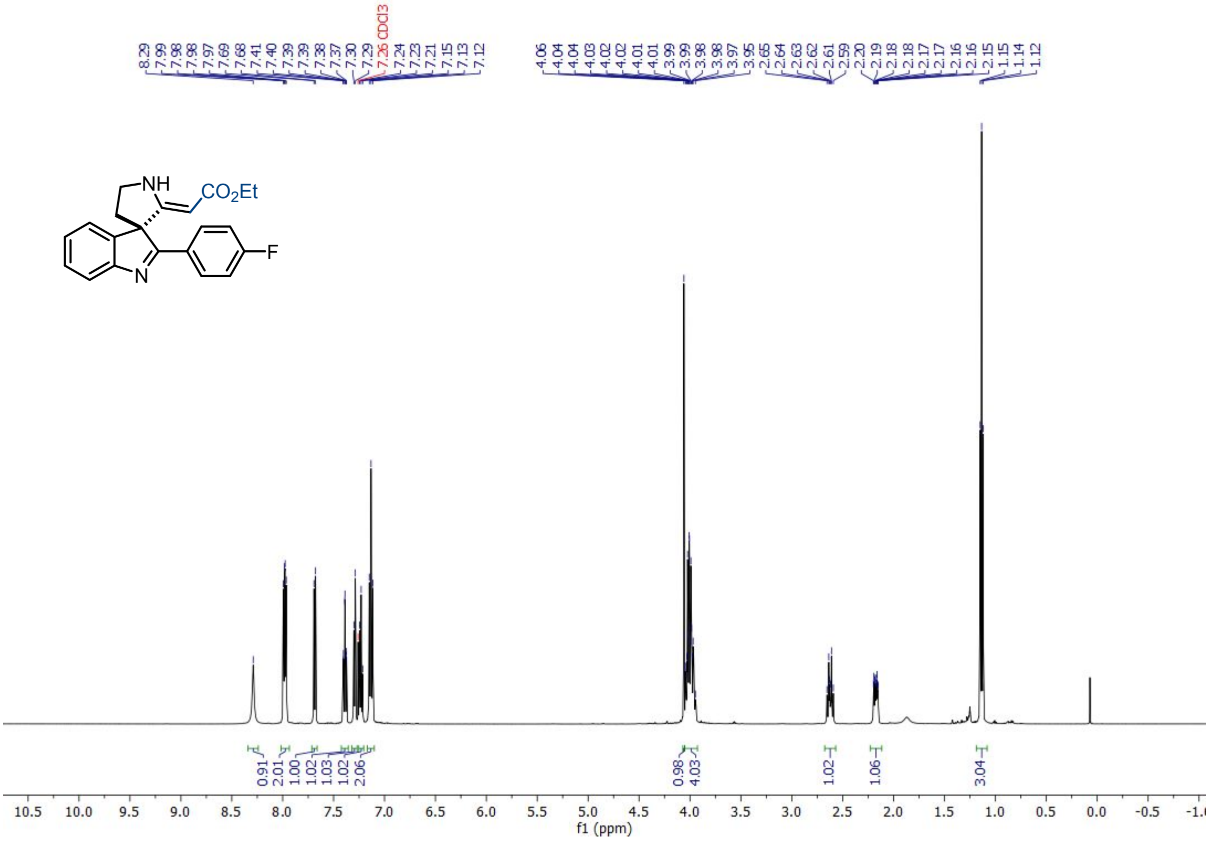

$^{13}C\{^{1}H\}$ NMR 126 MHz, $CDCl_3$ **23l**

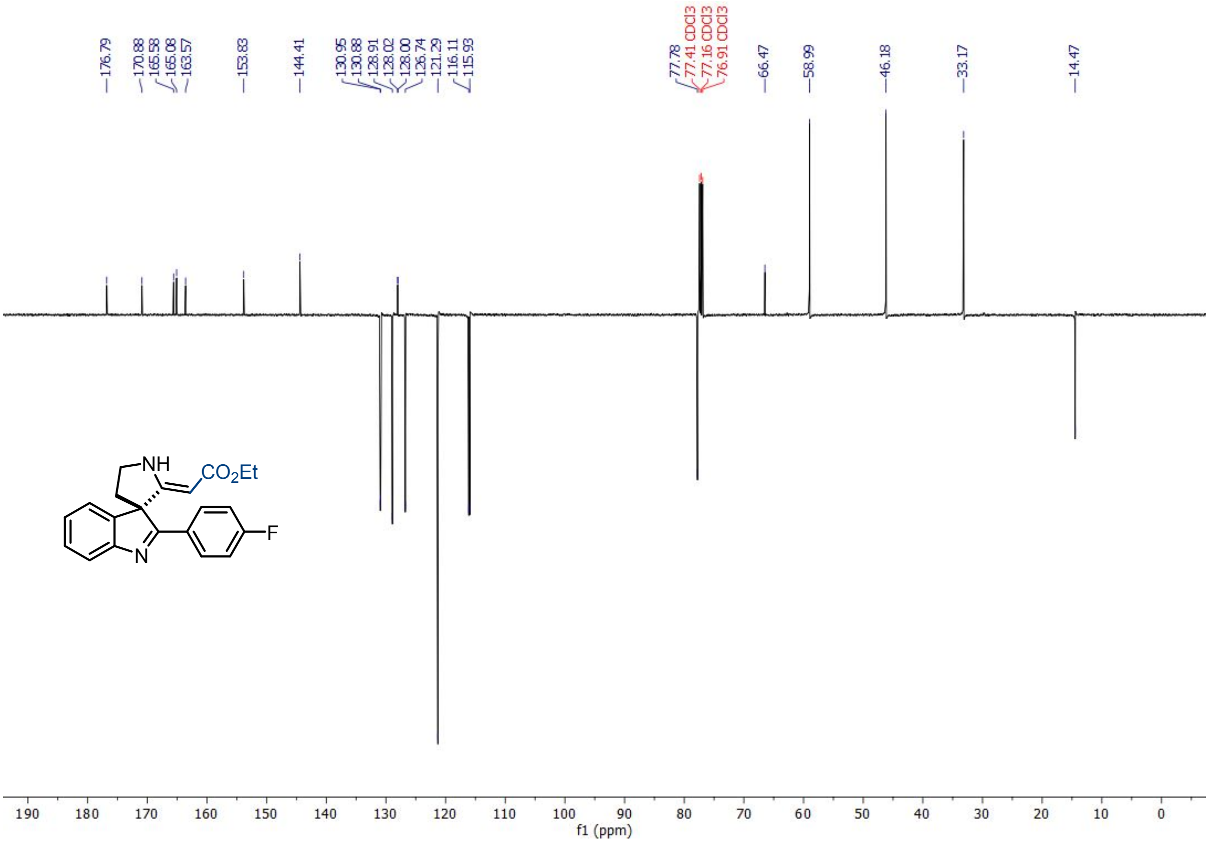

$^{19}F\{^{1}H\}$ NMR 470.4 MHz, $CDCl_3$ **23l**

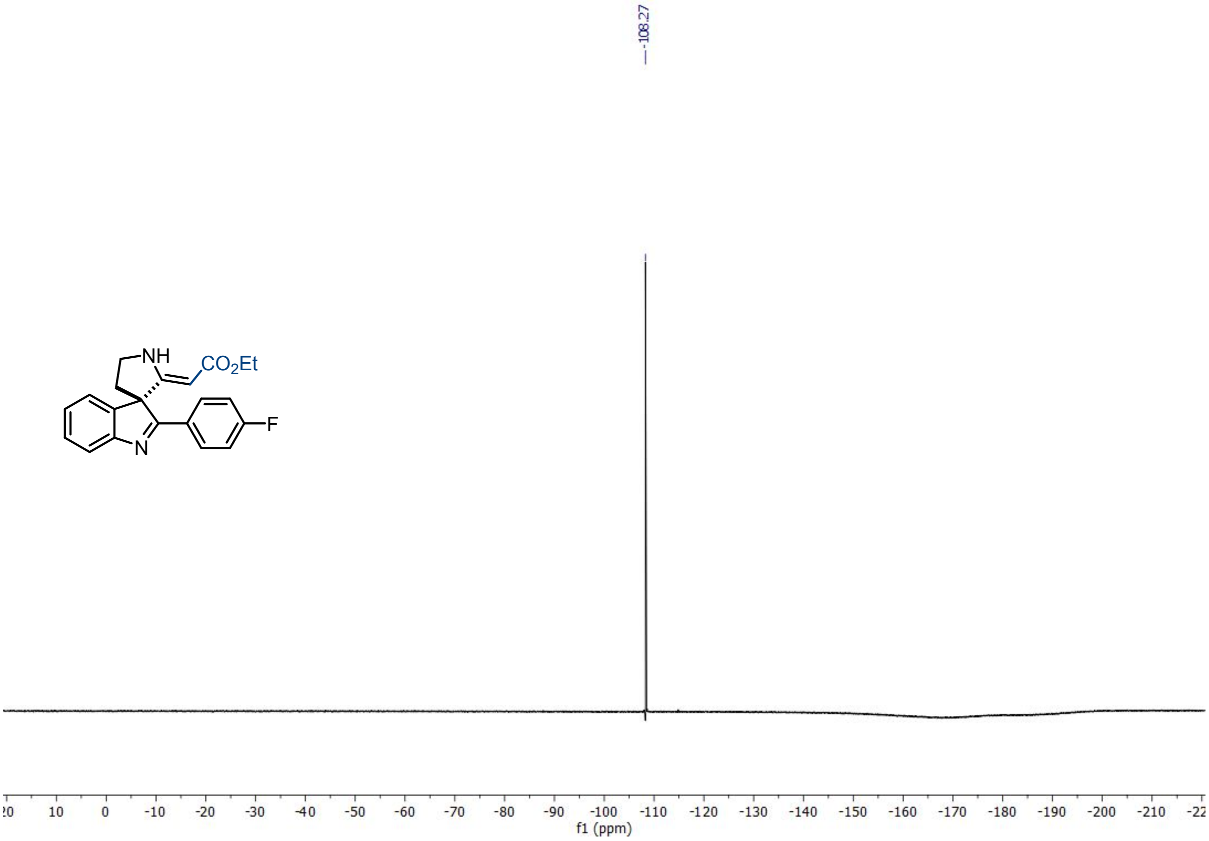

^{1}H NMR 500 MHz, $CDCl_3$ **23m**

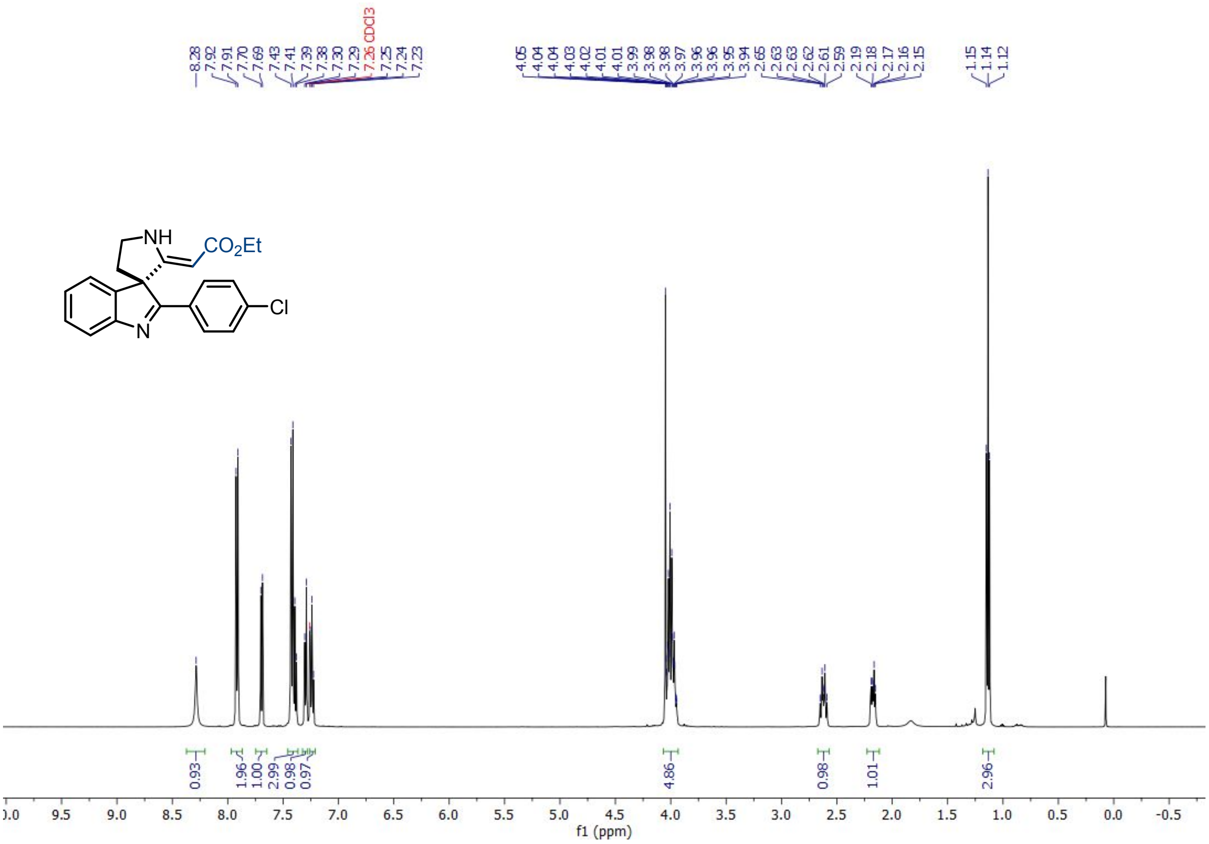

$^{13}C\{^{1}H\}$ NMR 126 MHz, $CDCl_3$ **23m**

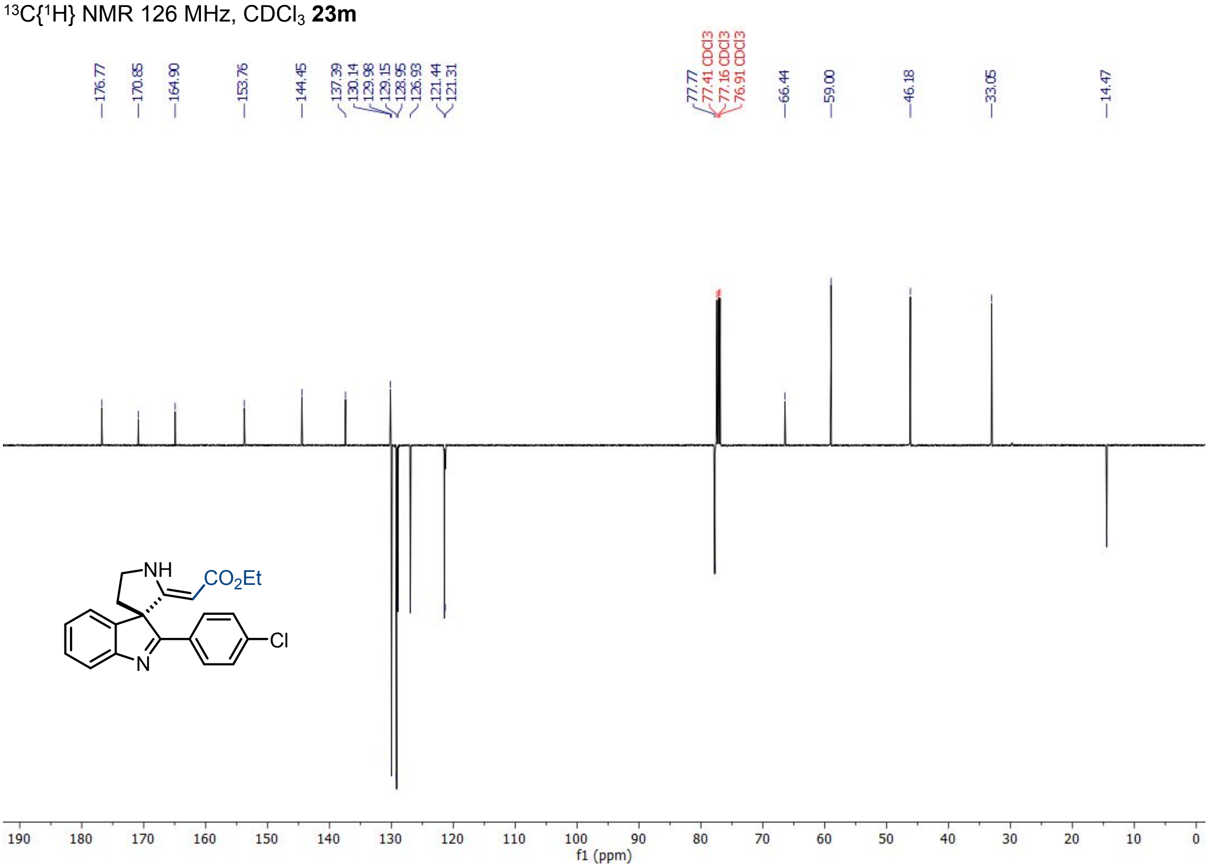

^{1}H NMR 500 MHz, $CDCl_3$ **23n**

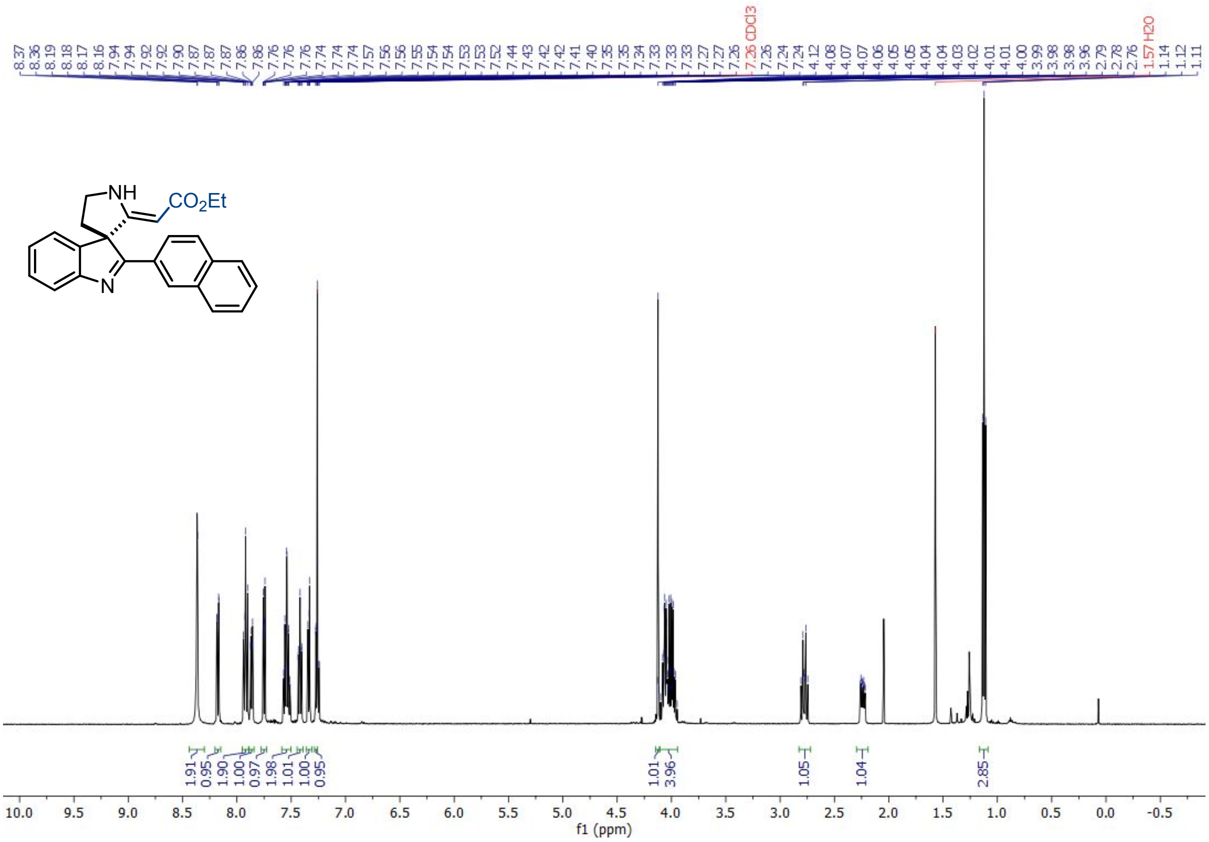

$^{13}C\{^1H\}$ NMR 126 MHz, $CDCl_3$ **23n**

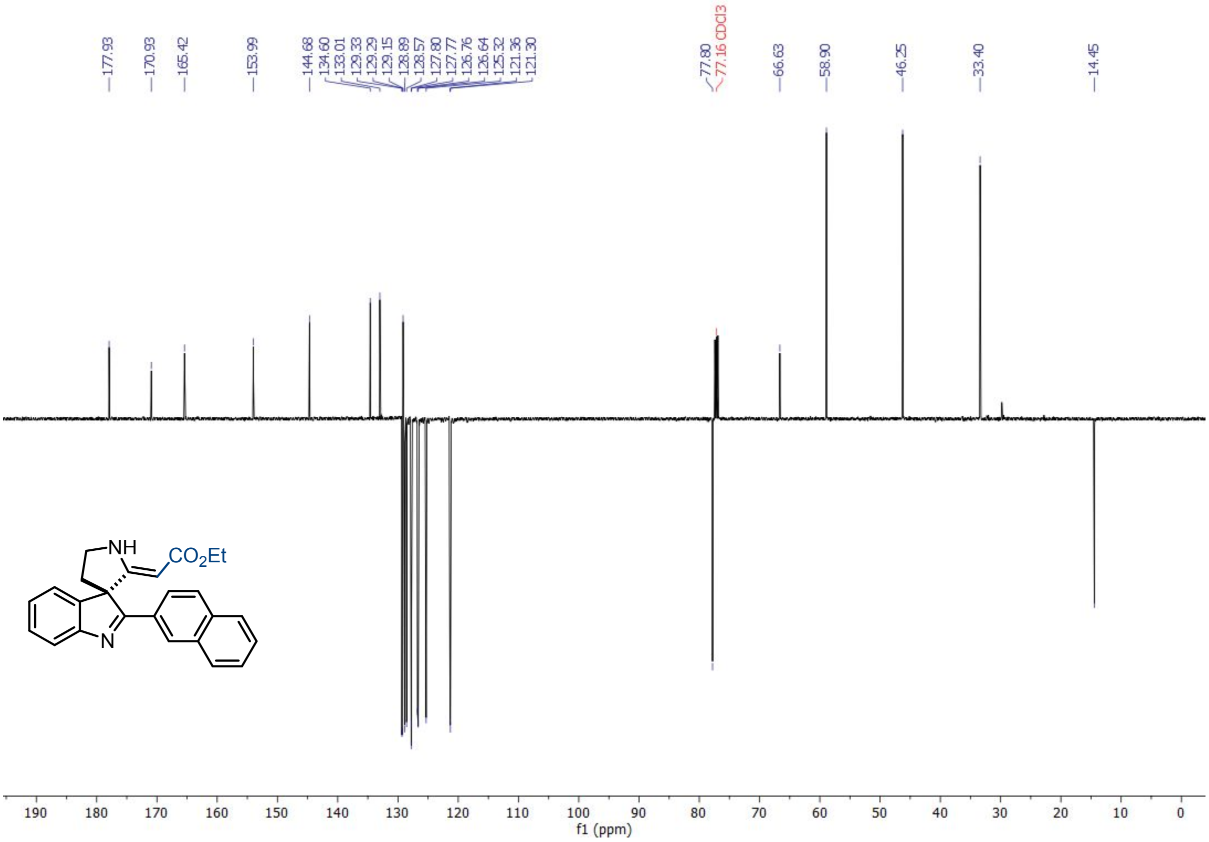

^{1}H NMR 500 MHz, $CDCl_3$ **23o**

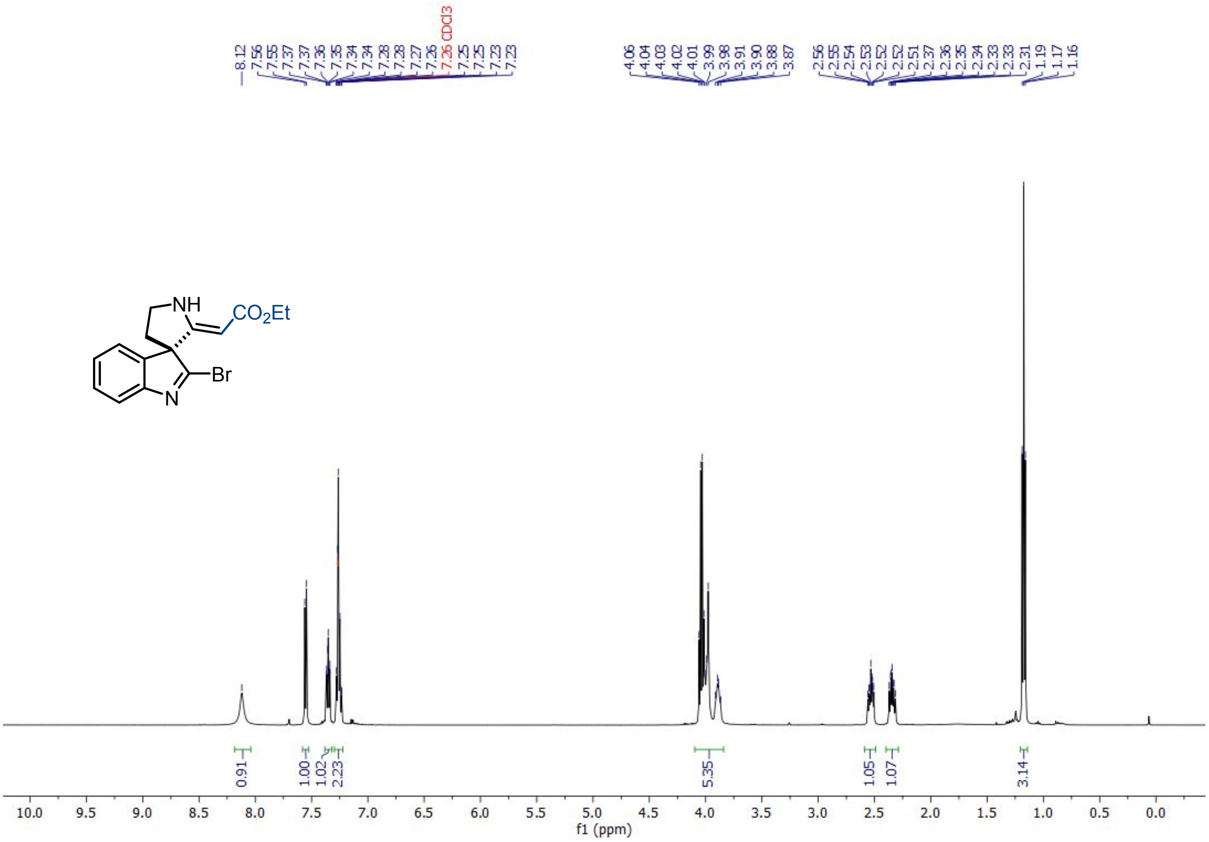

$^{13}C\{^{1}H\}$ NMR 126 MHz, $CDCl_3$ **23o**

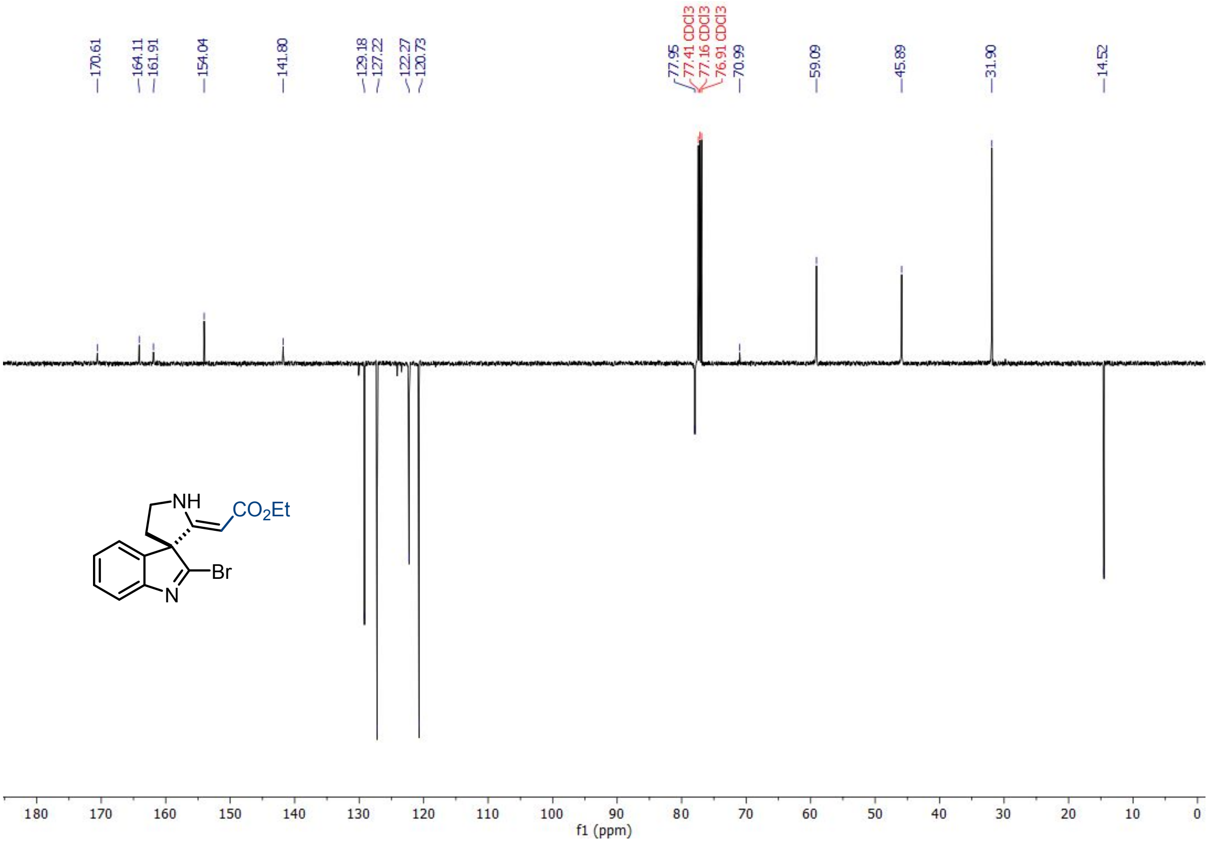

^{1}H NMR 500 MHz, $CDCl_3$ **23p**

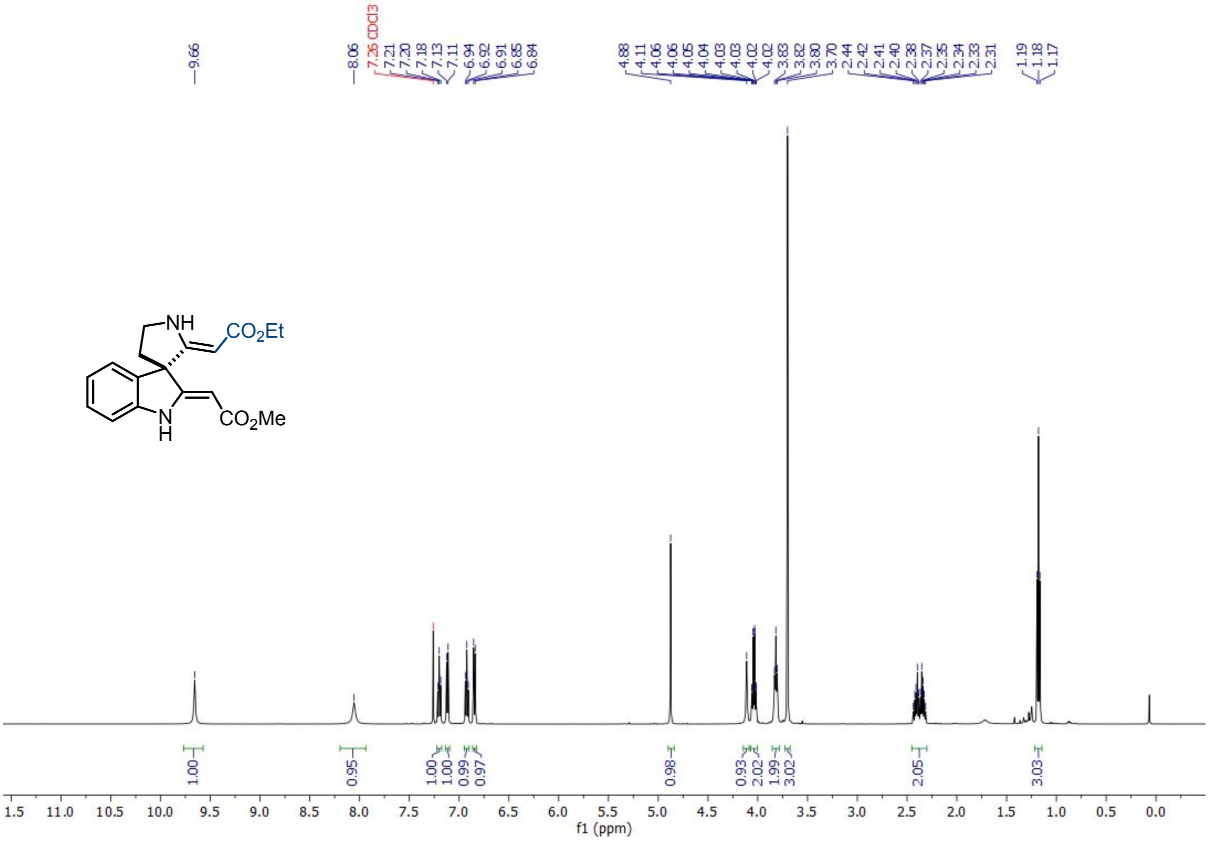

$^{13}C\{^{1}H\}$ NMR 126 MHz, $CDCl_3$ **23p**

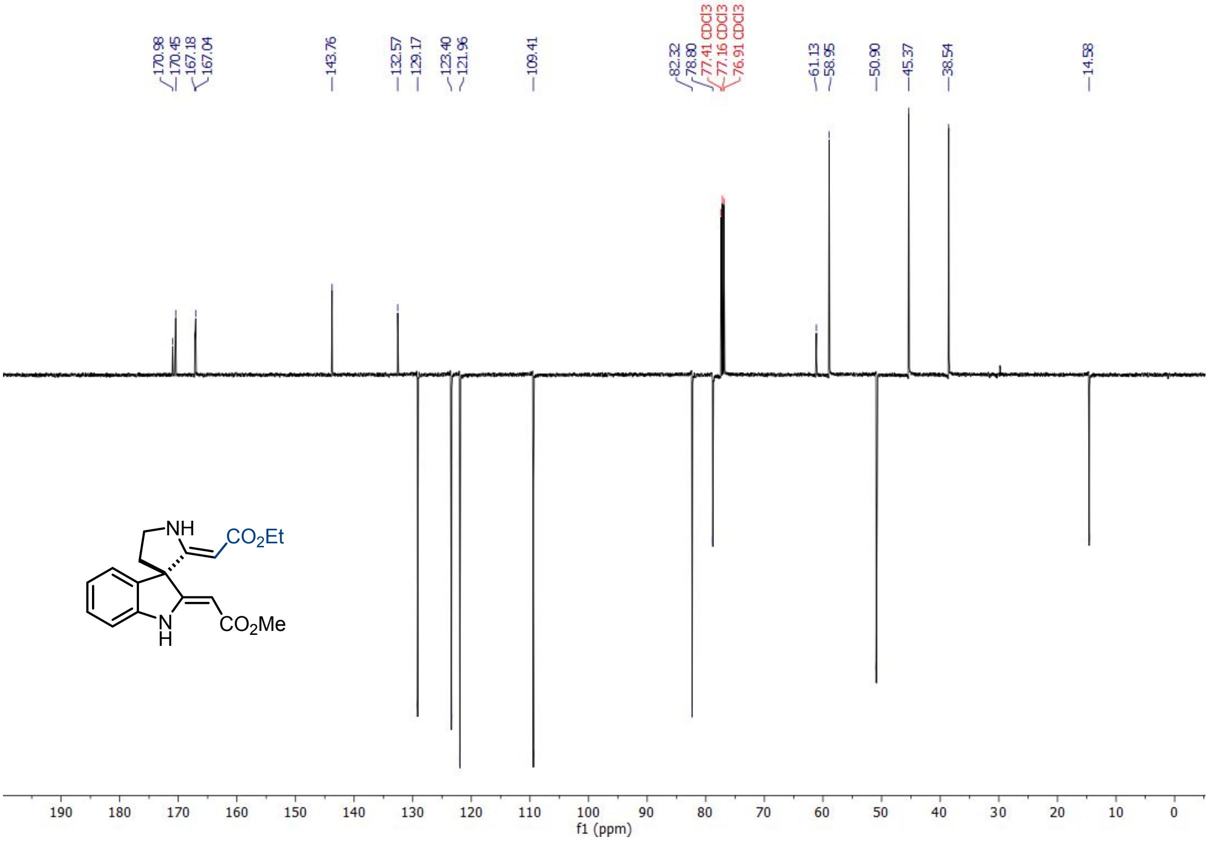

^{1}H NMR 500 MHz, $CDCl_3$ **25a**

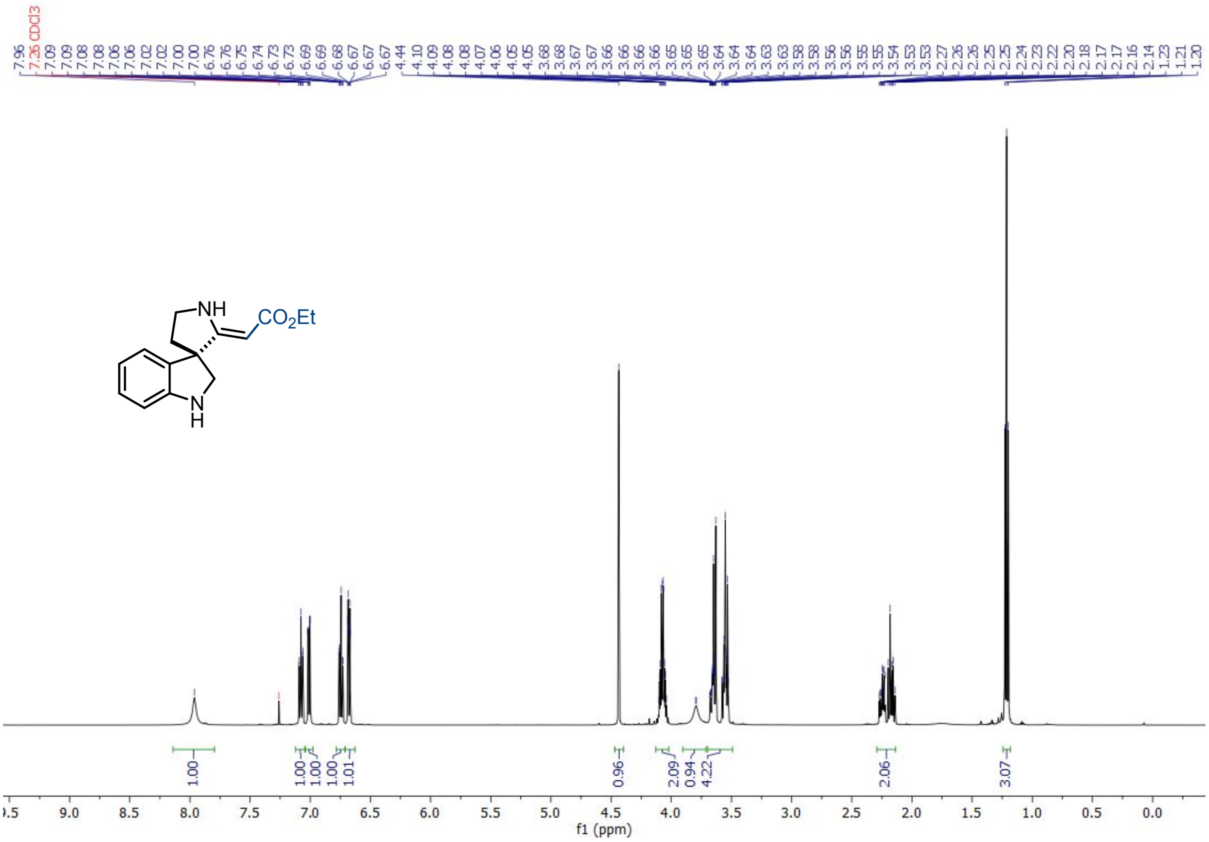

$^{13}C\{^{1}H\}$ NMR 126 MHz, $CDCl_3$ **25a**

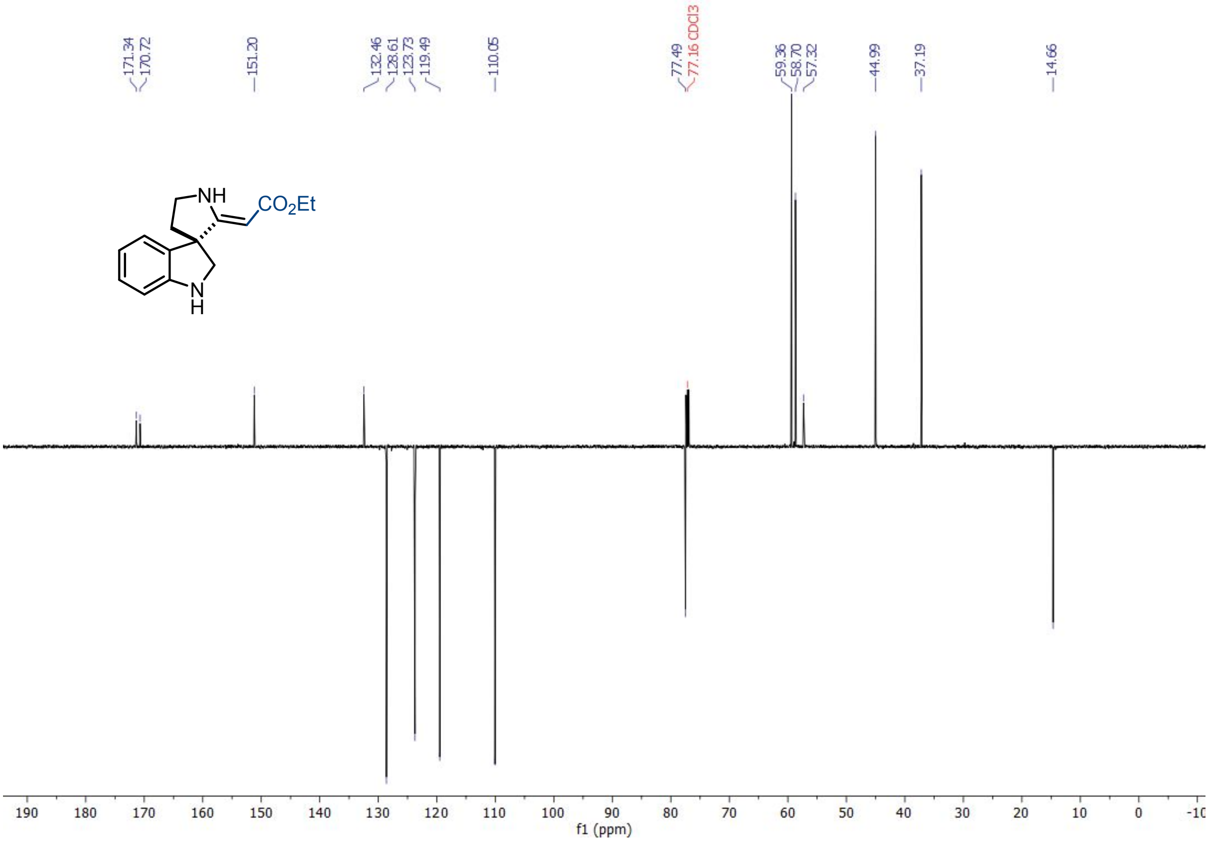

^{1}H NMR 500 MHz, $CDCl_3$ **25b**

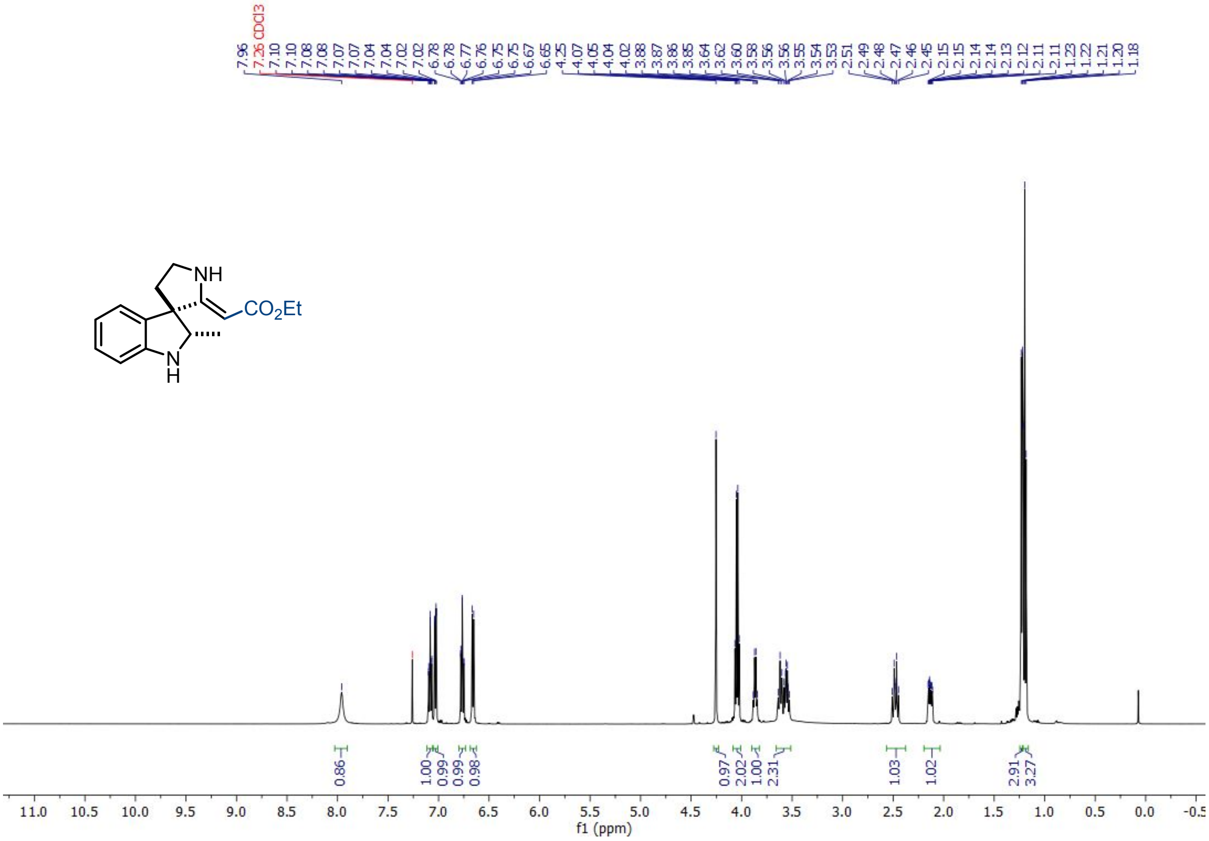

$^{13}C\{^1H\}$ NMR 126 MHz, $CDCl_3$ **25b**

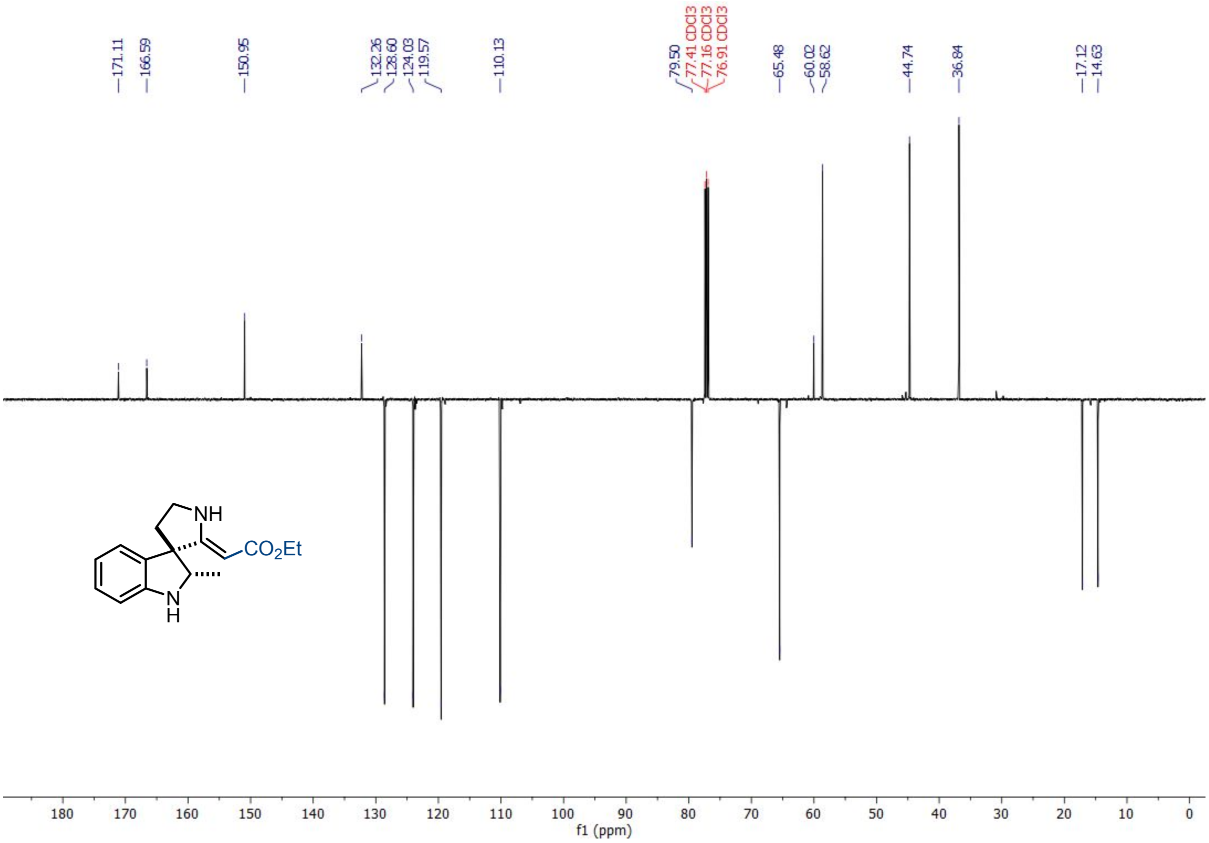

^{1}H NMR 500 MHz, $CDCl_3$ **25q**

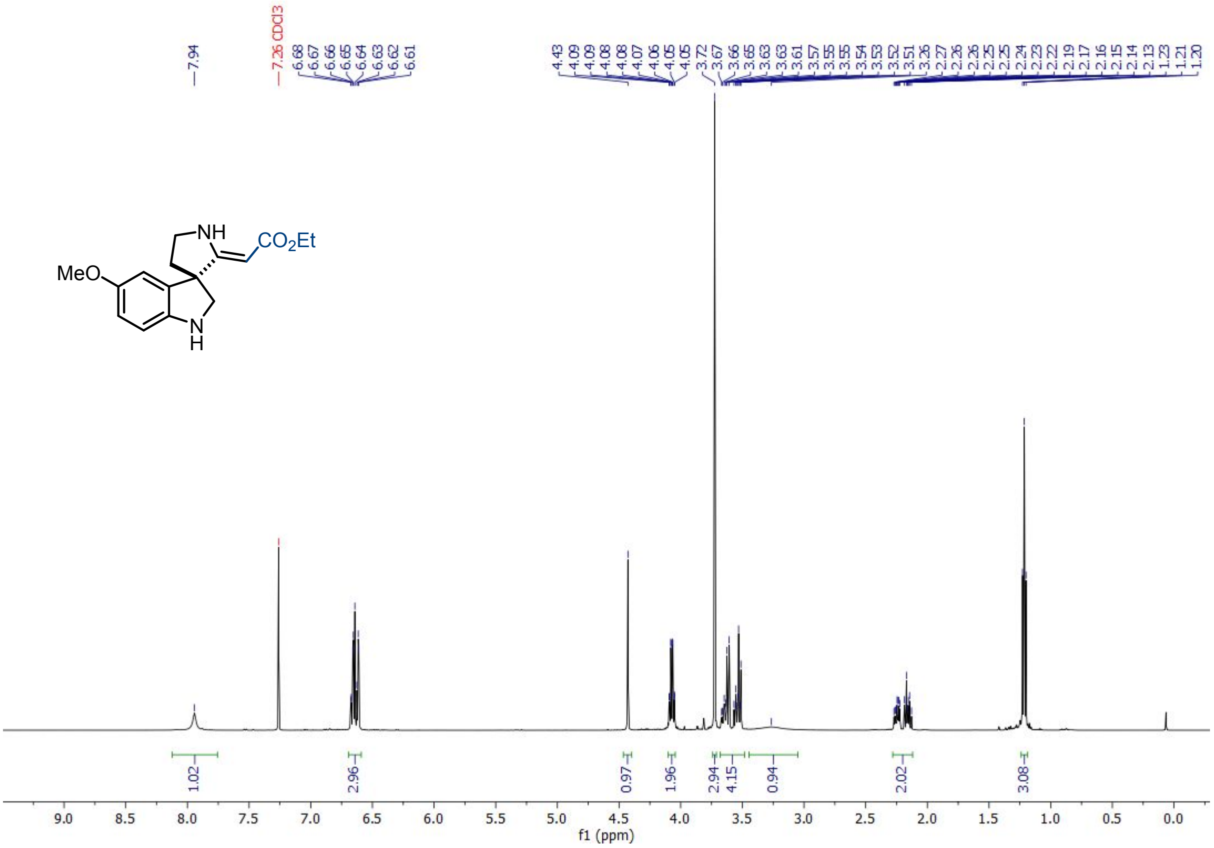

$^{13}C\{^{1}H\}$ NMR 126 MHz, $CDCl_3$ **25q**

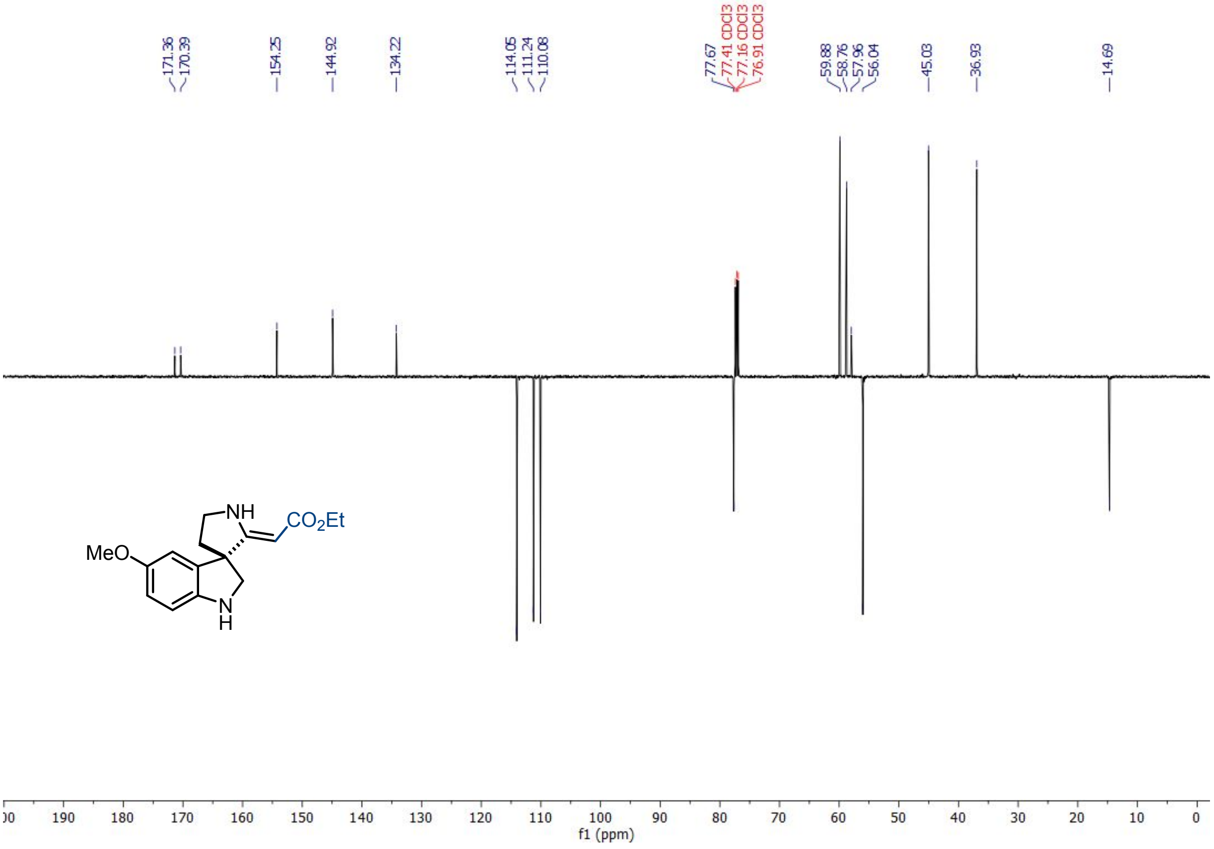

^{1}H NMR 500 MHz, $CDCl_3$ **25r**

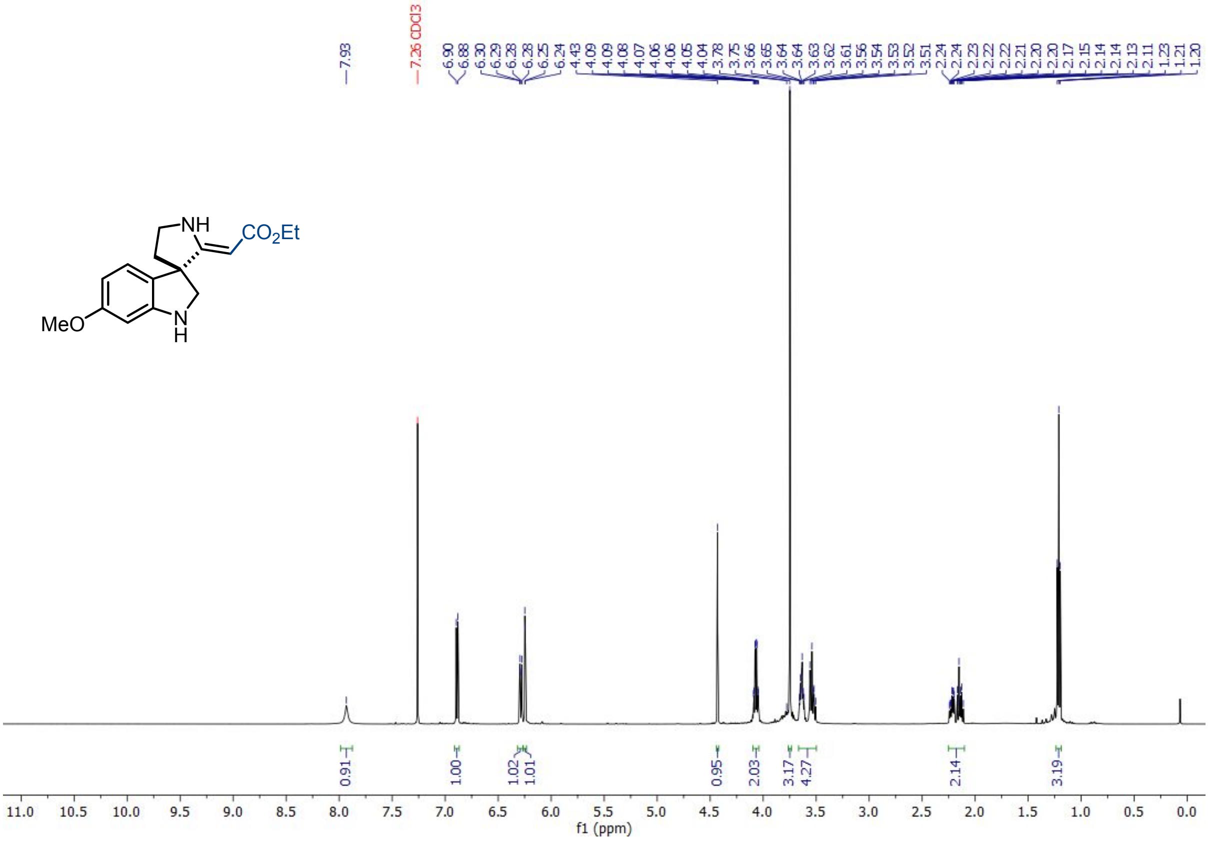

$^{13}C\{^1H\}$ NMR 126 MHz, $CDCl_3$ **25r**

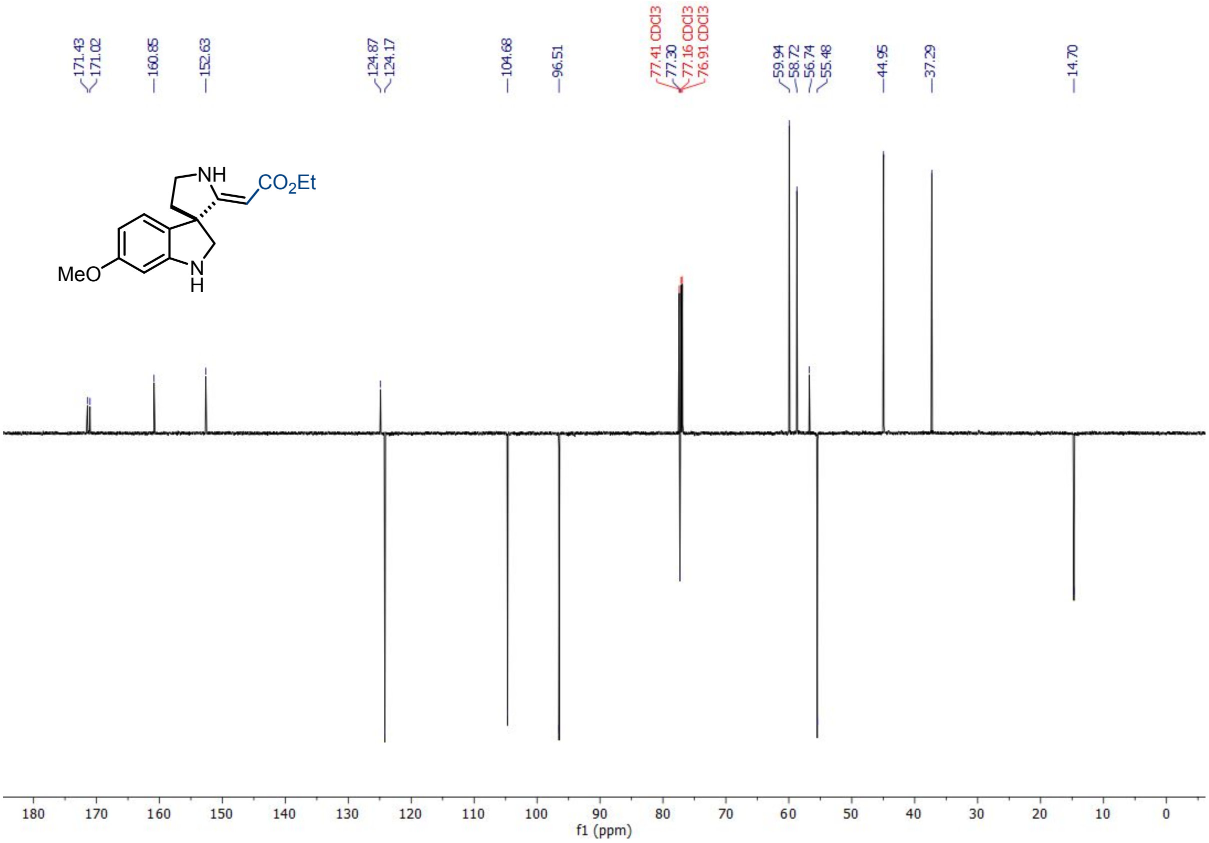

^{1}H NMR 500 MHz, $CDCl_3$ **25s**

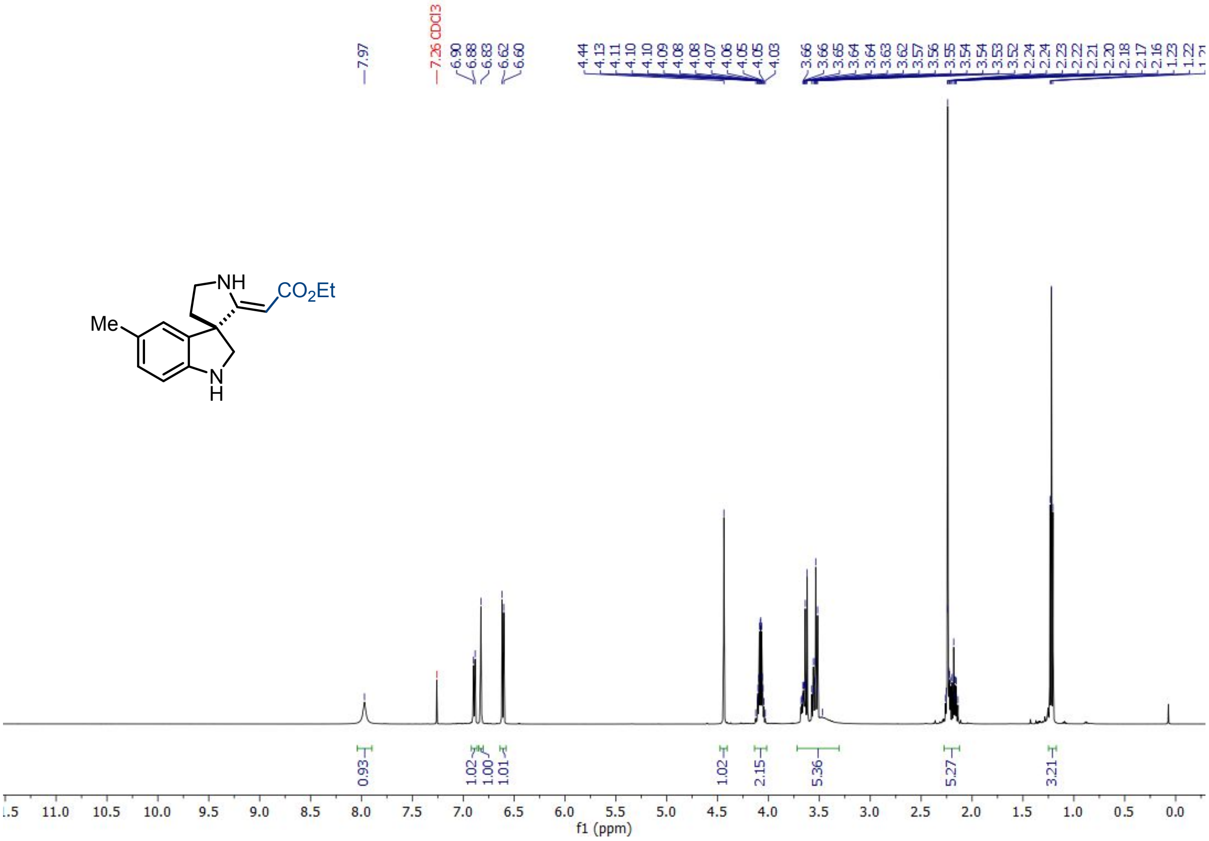

^{13}C{^{1}H} NMR 126 MHz, $CDCl_3$ **25s**

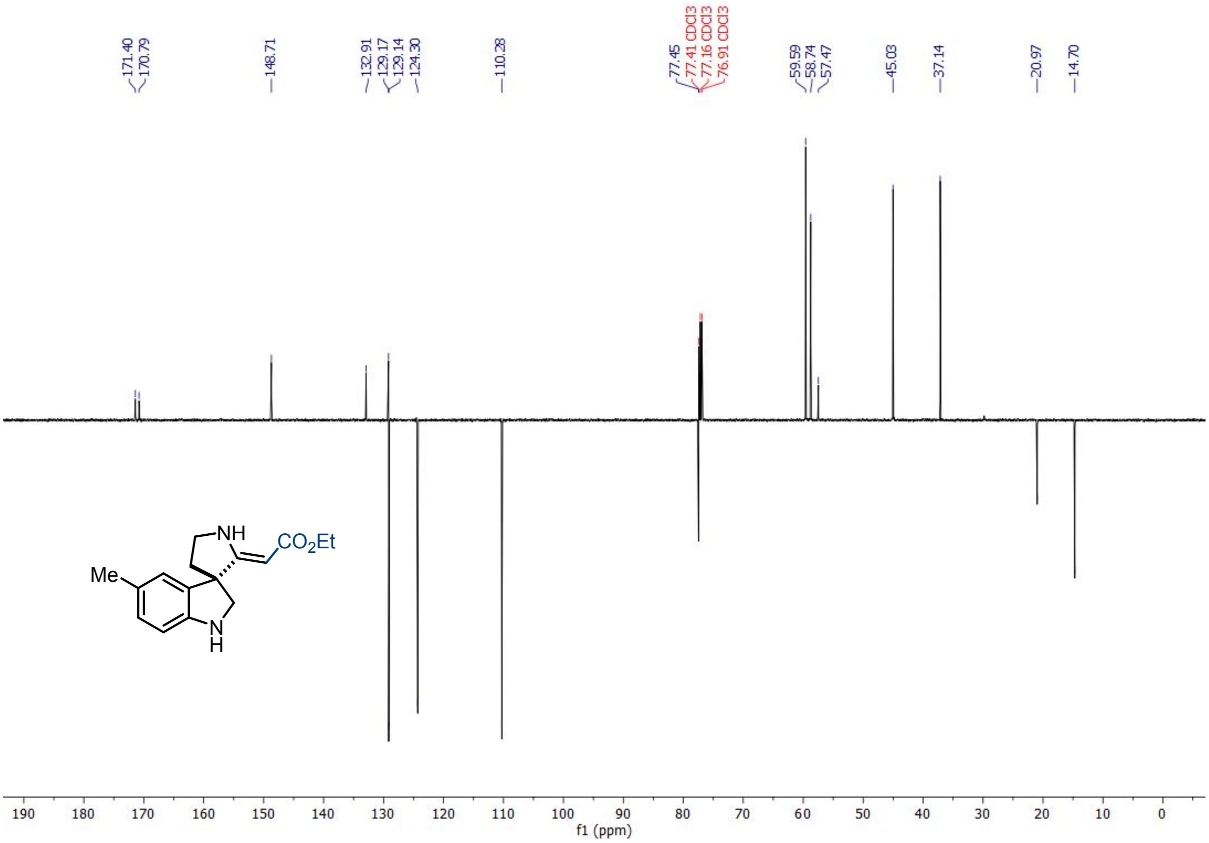

^{1}H NMR 500 MHz, $CDCl_3$ **25t**

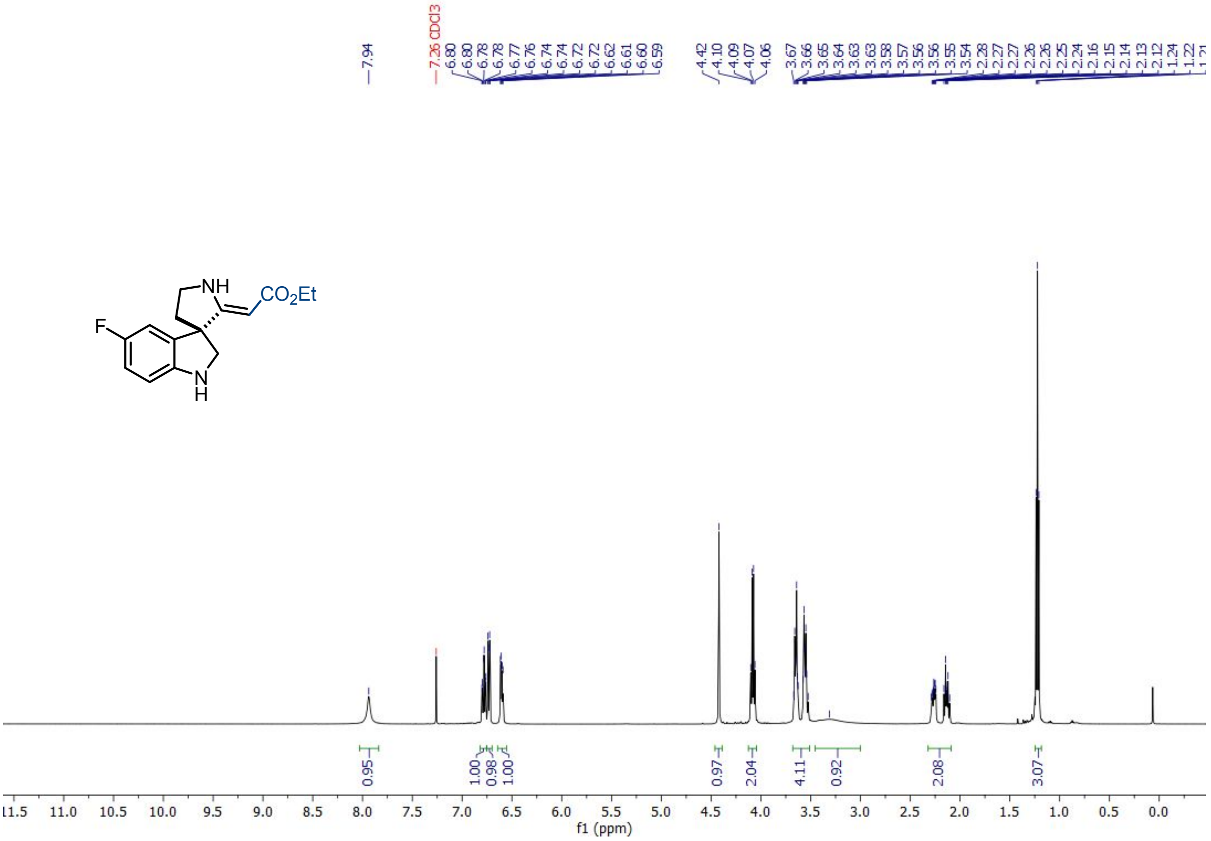

^{13}C{^{1}H} NMR 125.8 MHz, $CDCl_3$ **25t**

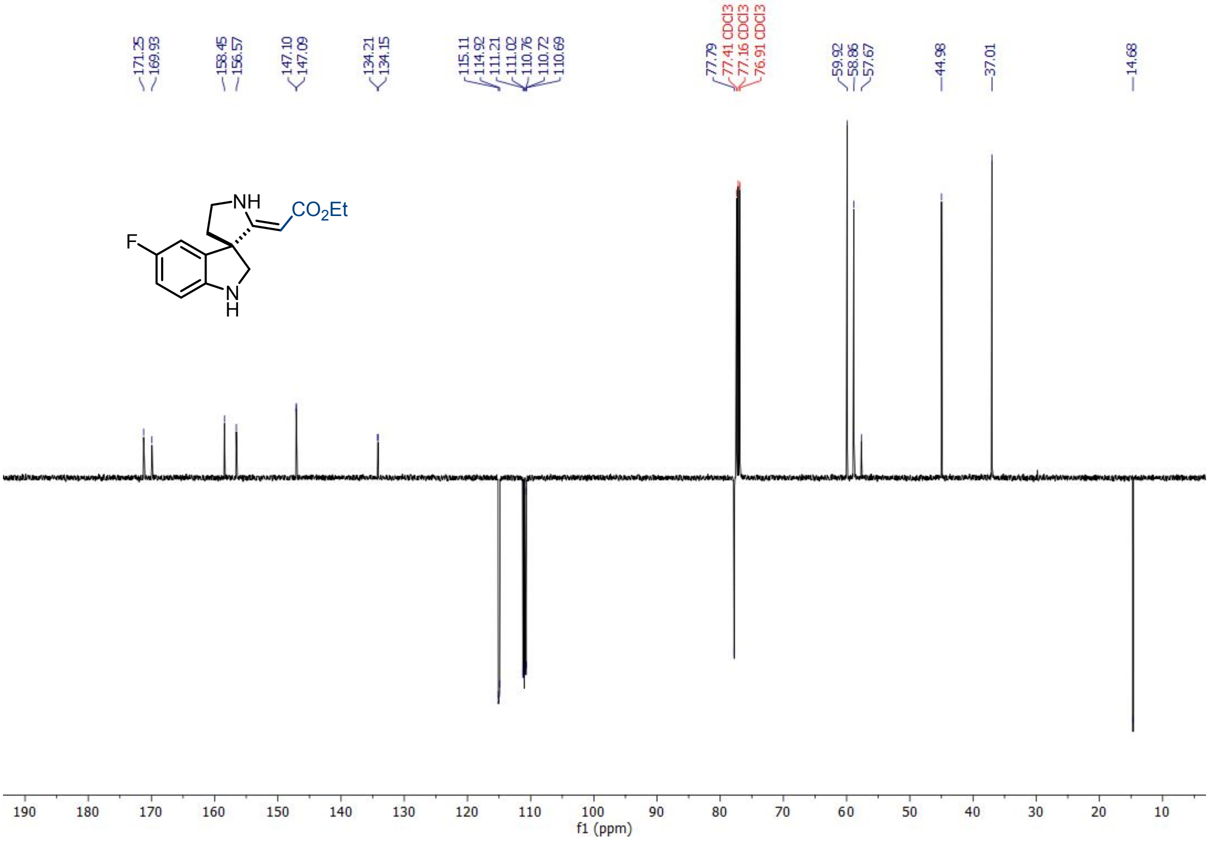

$^{19}F\{^{1}H\}$ NMR 470.4 MHz, $CDCl_3$ **25t**

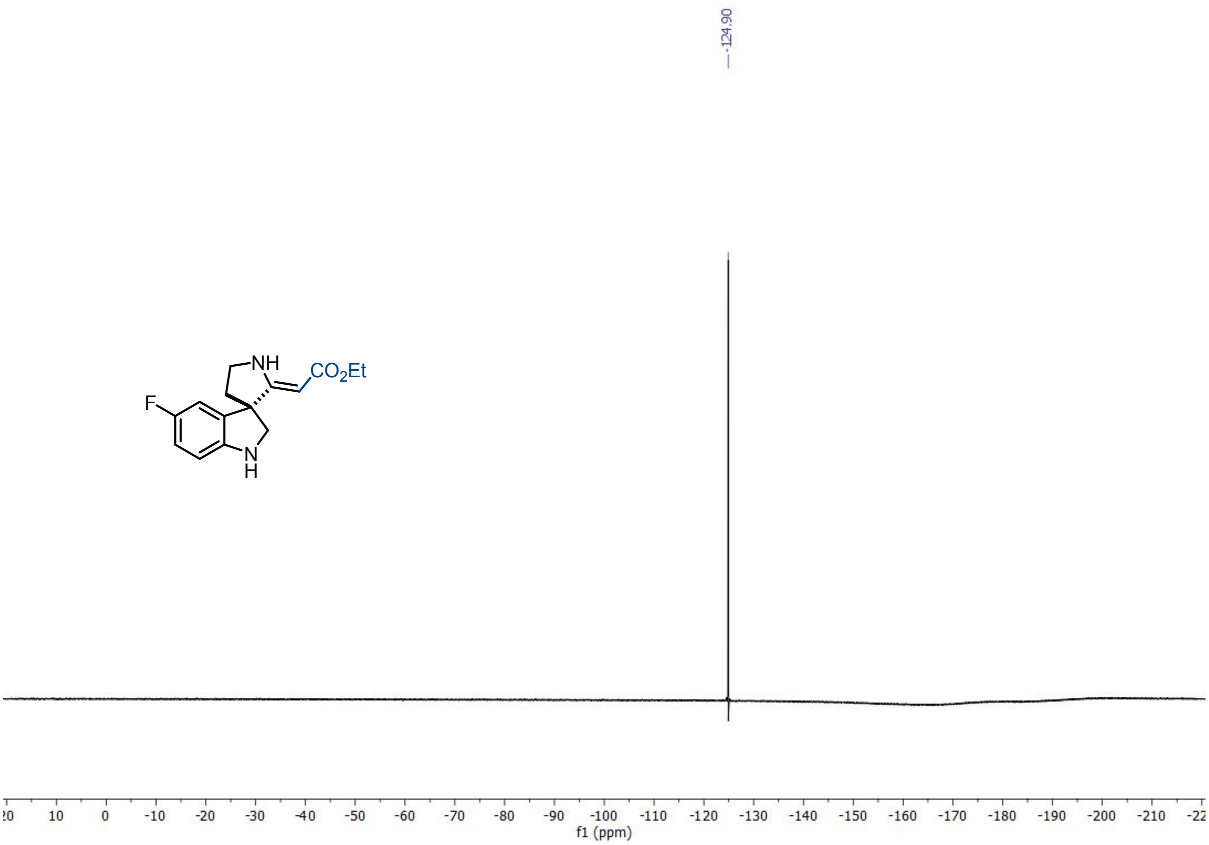

^{1}H NMR 500 MHz, $CDCl_3$ **25u**

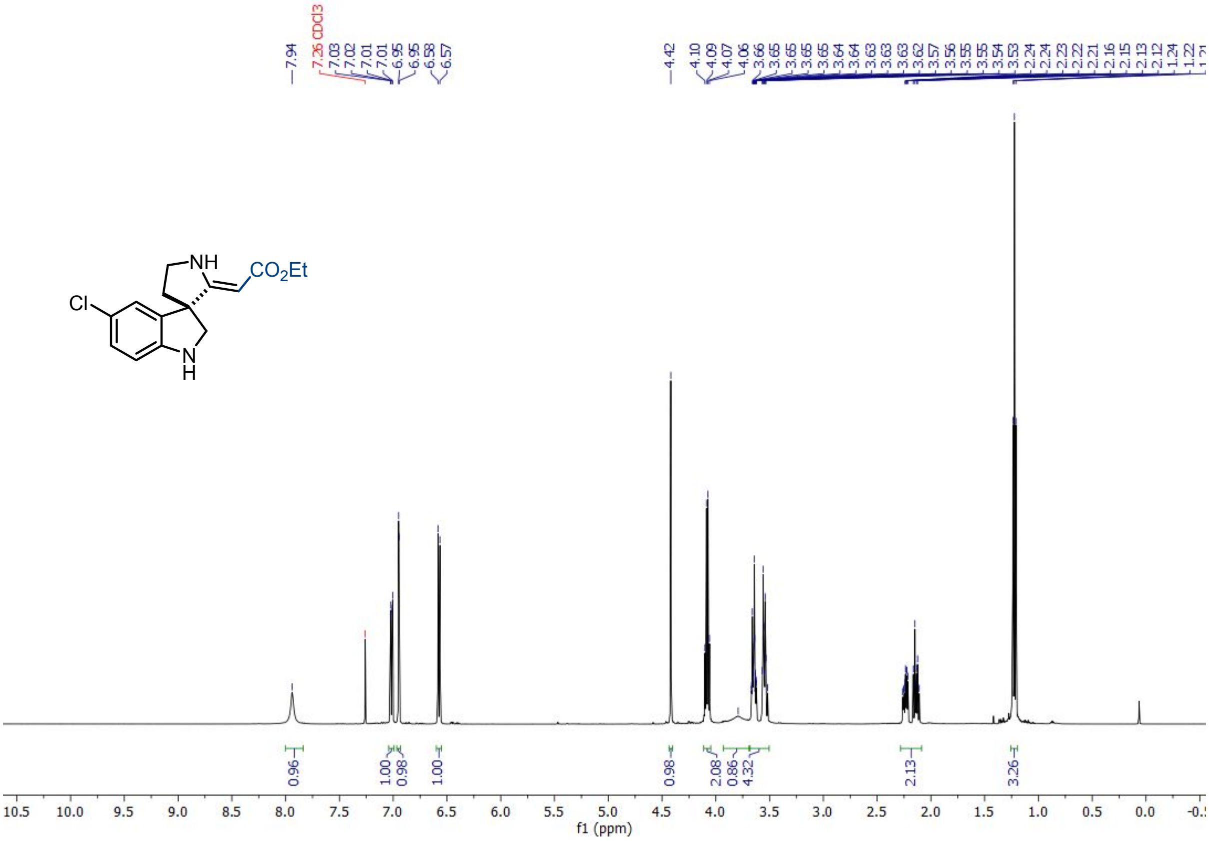

^{13}C{^{1}H} NMR 126 MHz, $CDCl_3$ **25u**

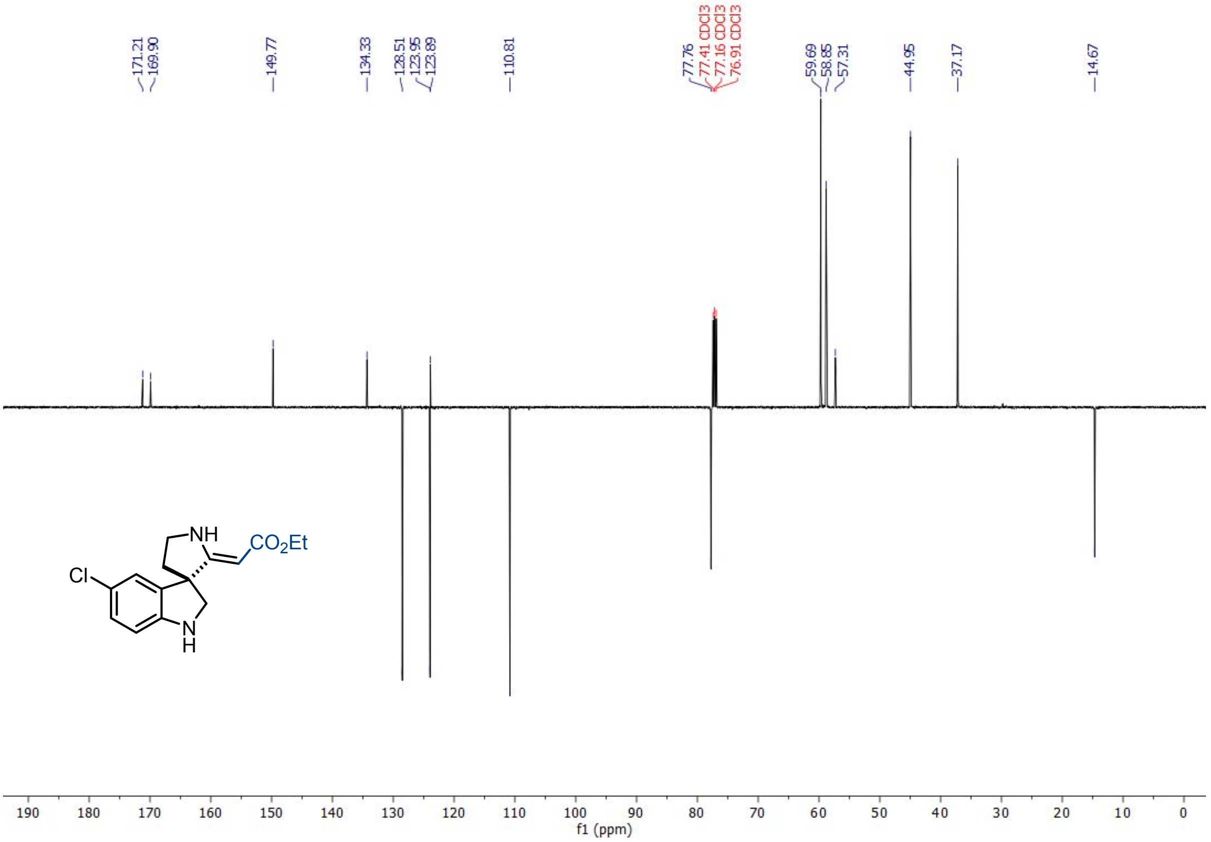

^{1}H NMR 500 MHz, $CDCl_3$ **25v**

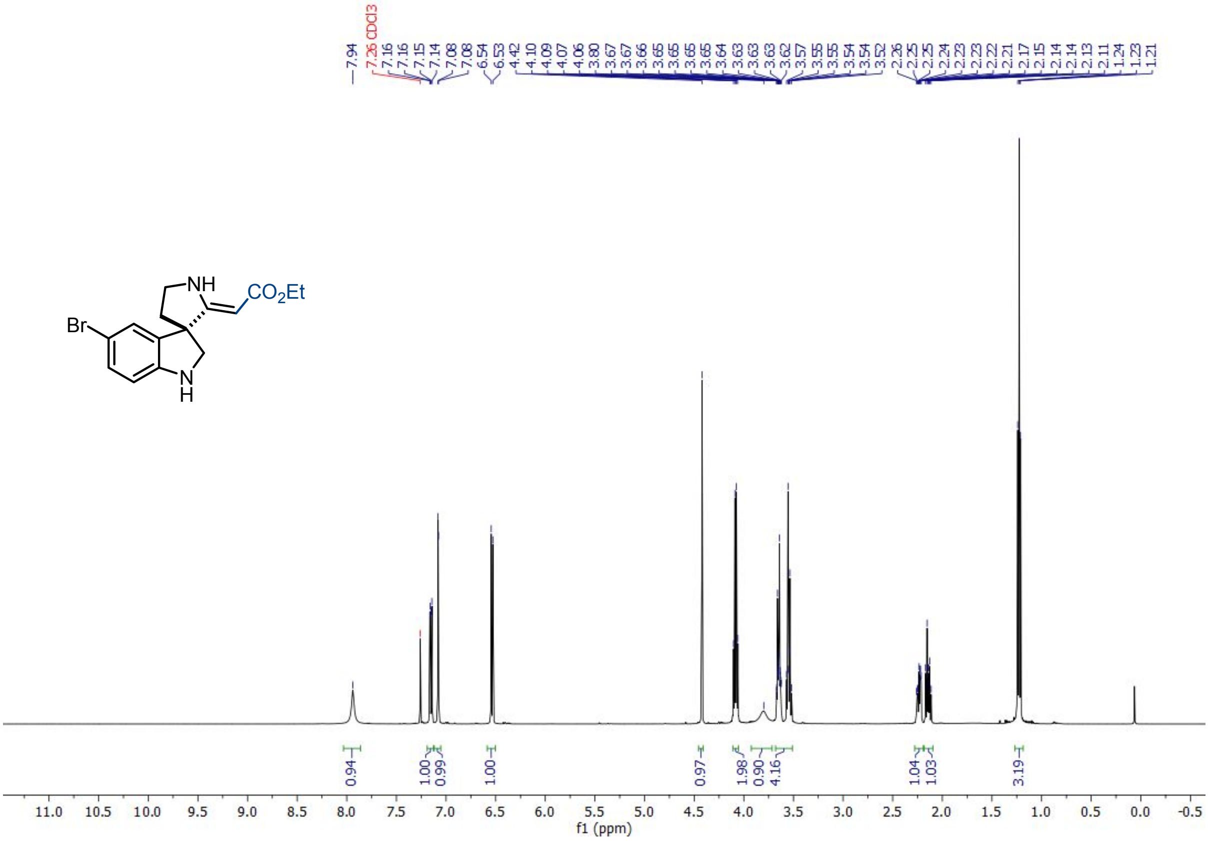

^{13}C{^{1}H} NMR 126 MHz, $CDCl_3$ **25v**

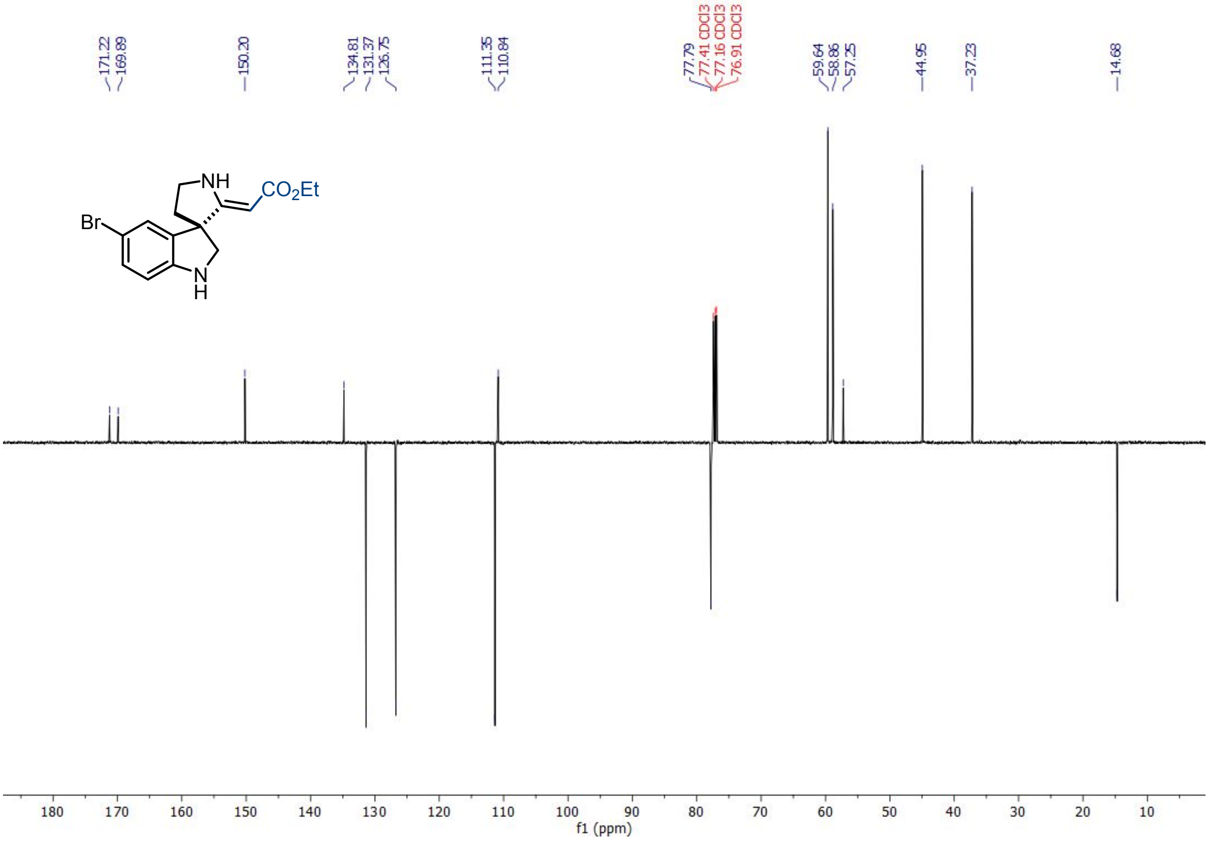

^{1}H NMR 500 MHz, $CDCl_3$ **25w D1**

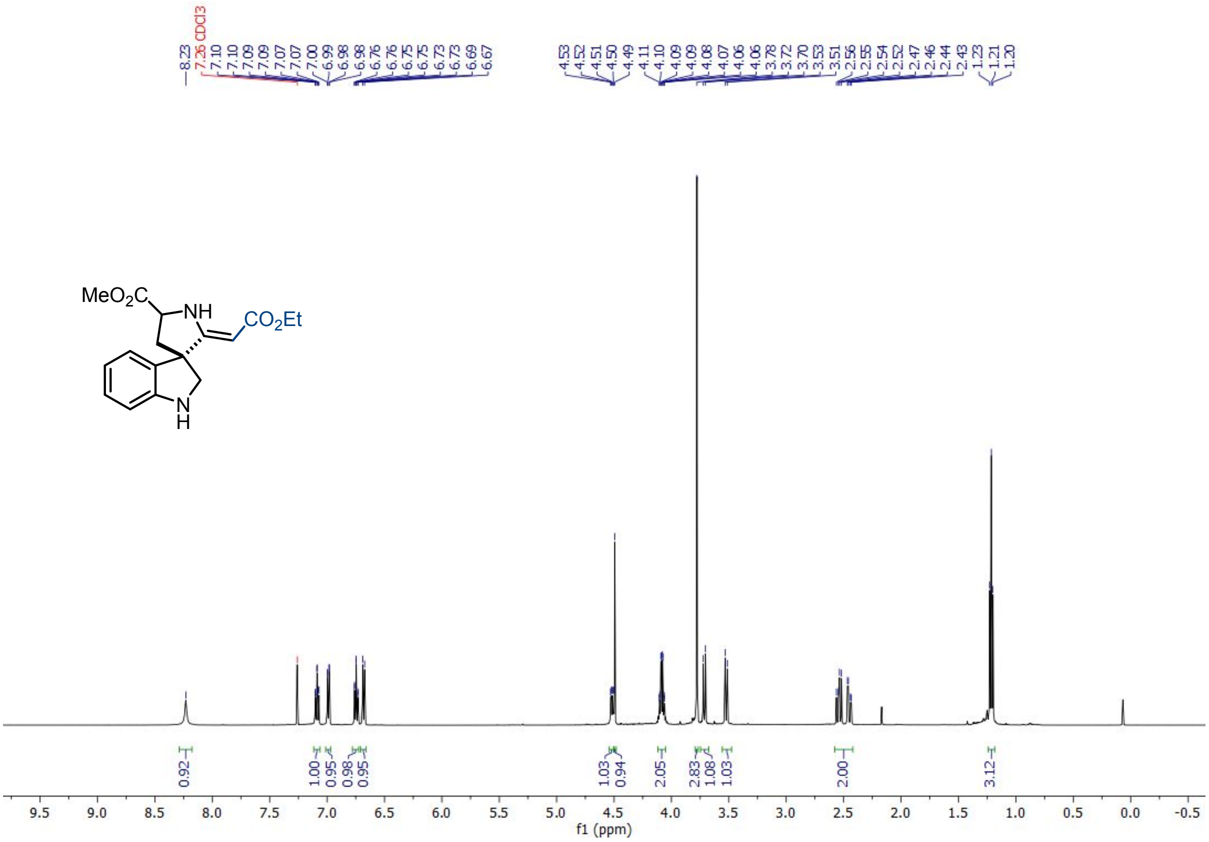

$^{13}C\{^{1}H\}$ NMR 125.8 MHz, $CDCl_3$ **25w D1**

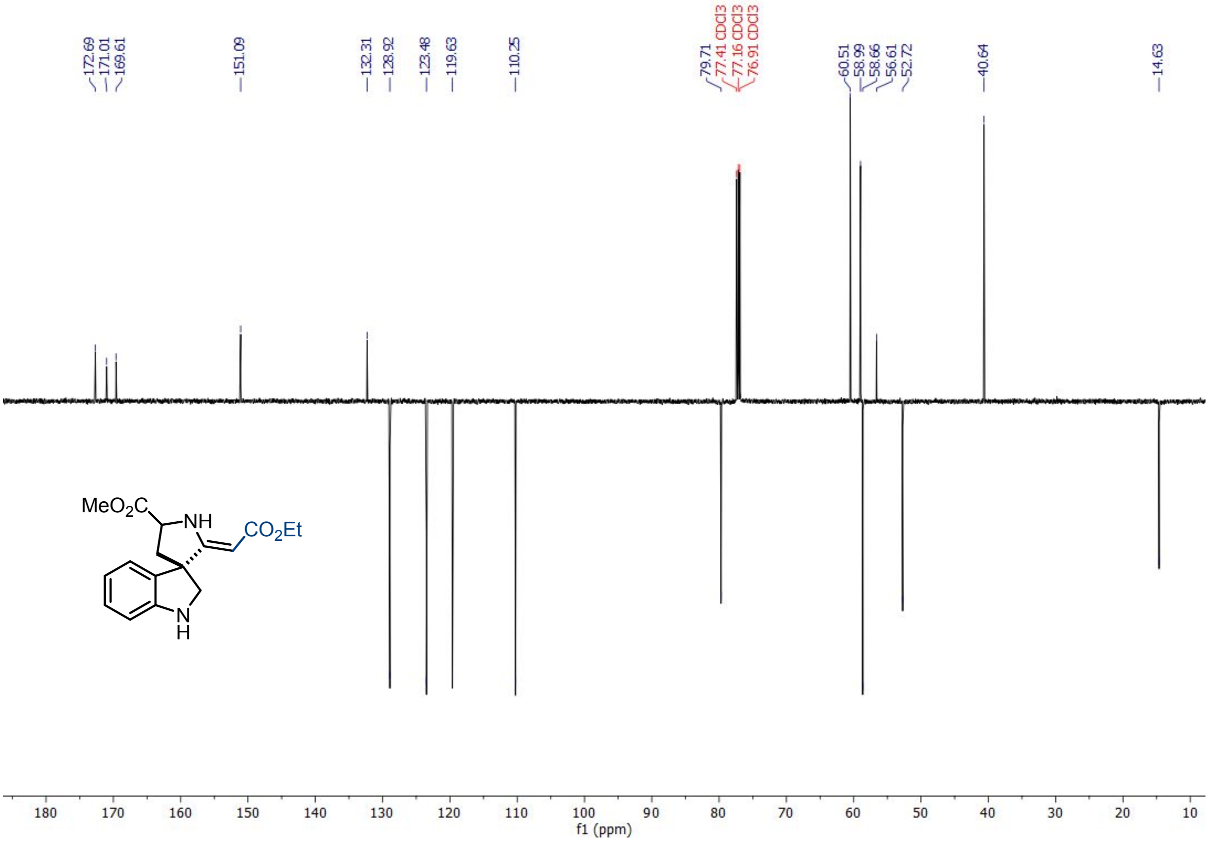

^{1}H NMR 500 MHz, $CDCl_3$ **25w D2**

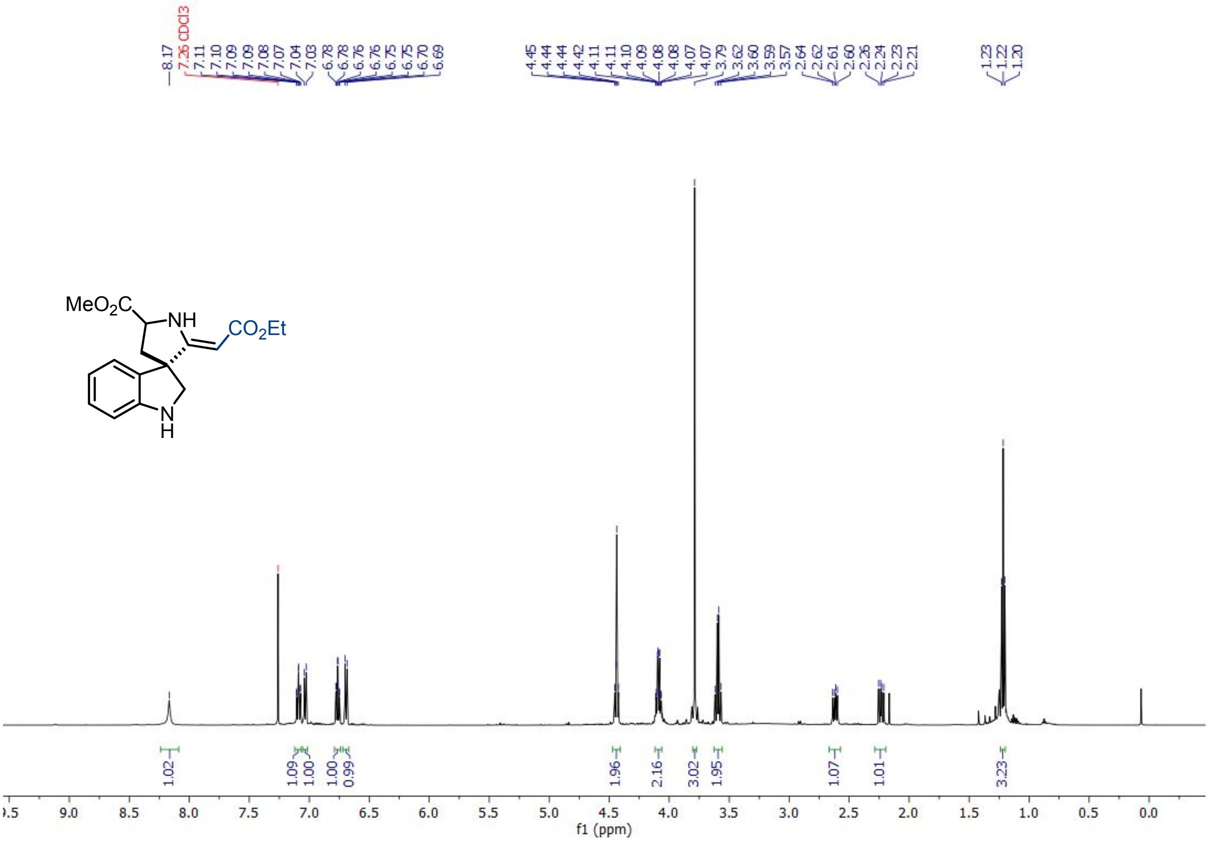

$^{13}C\{^{1}H\}$ NMR 125.8 MHz, $CDCl_3$ **25w D2**

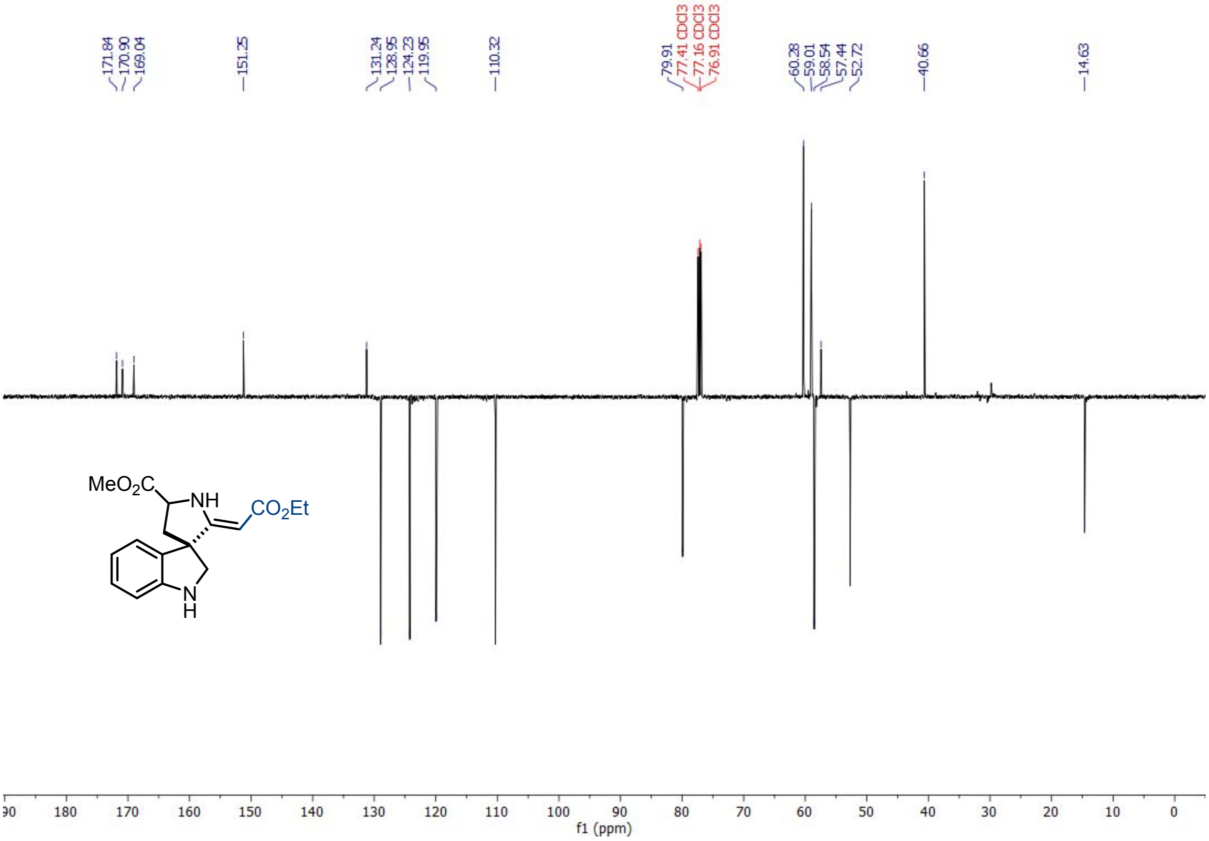

^{1}H NMR 500 MHz, $CDCl_3$ **25x D1**

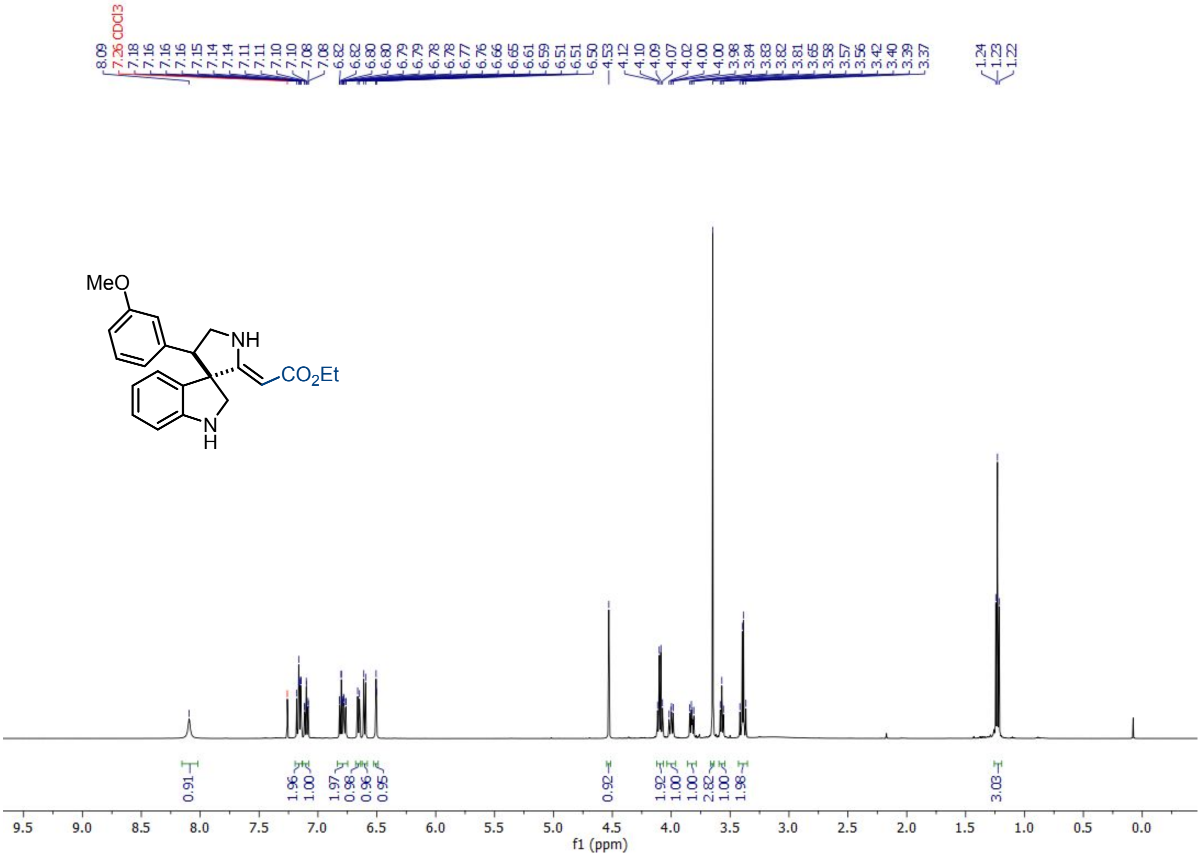

$^{13}C\{^1H\}$ NMR 126 MHz, $CDCl_3$ **25x D1**

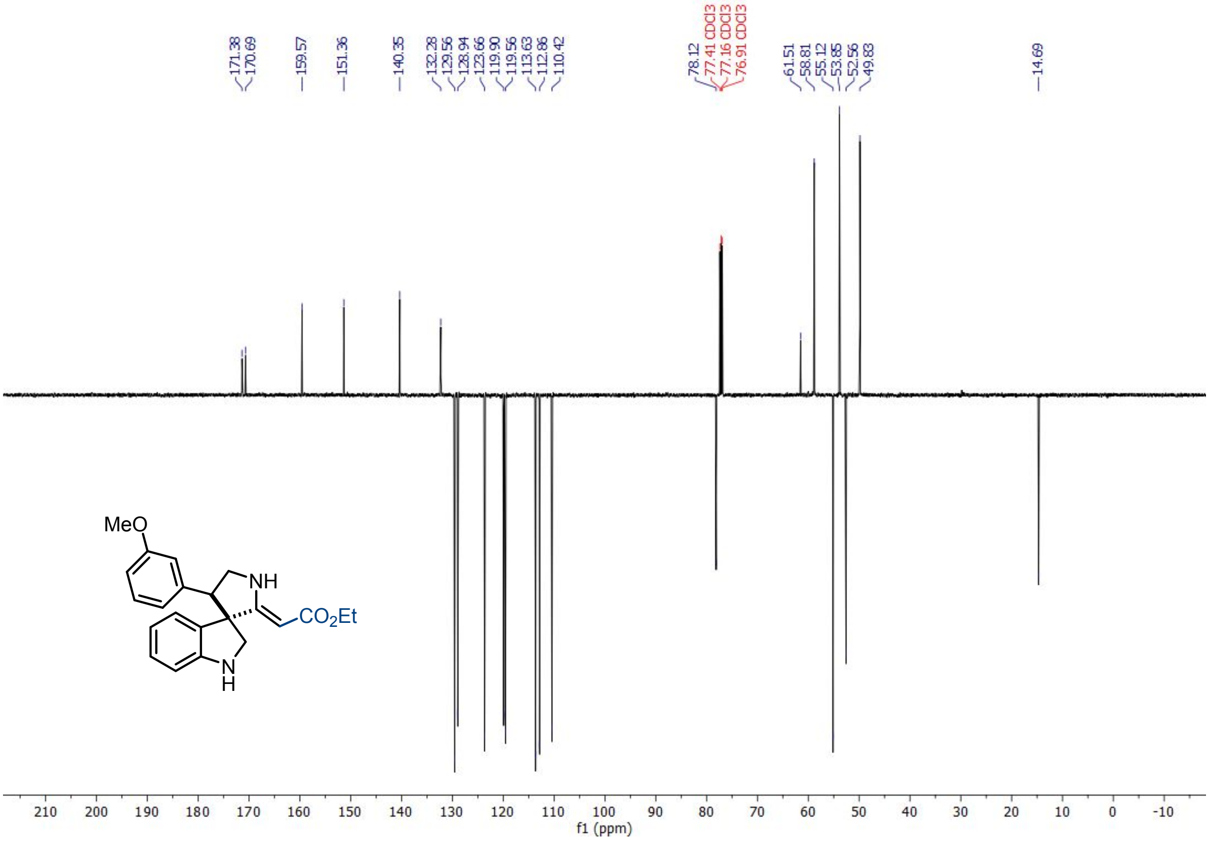

^{1}H NMR 500 MHz, $CDCl_3$ **25x D2**

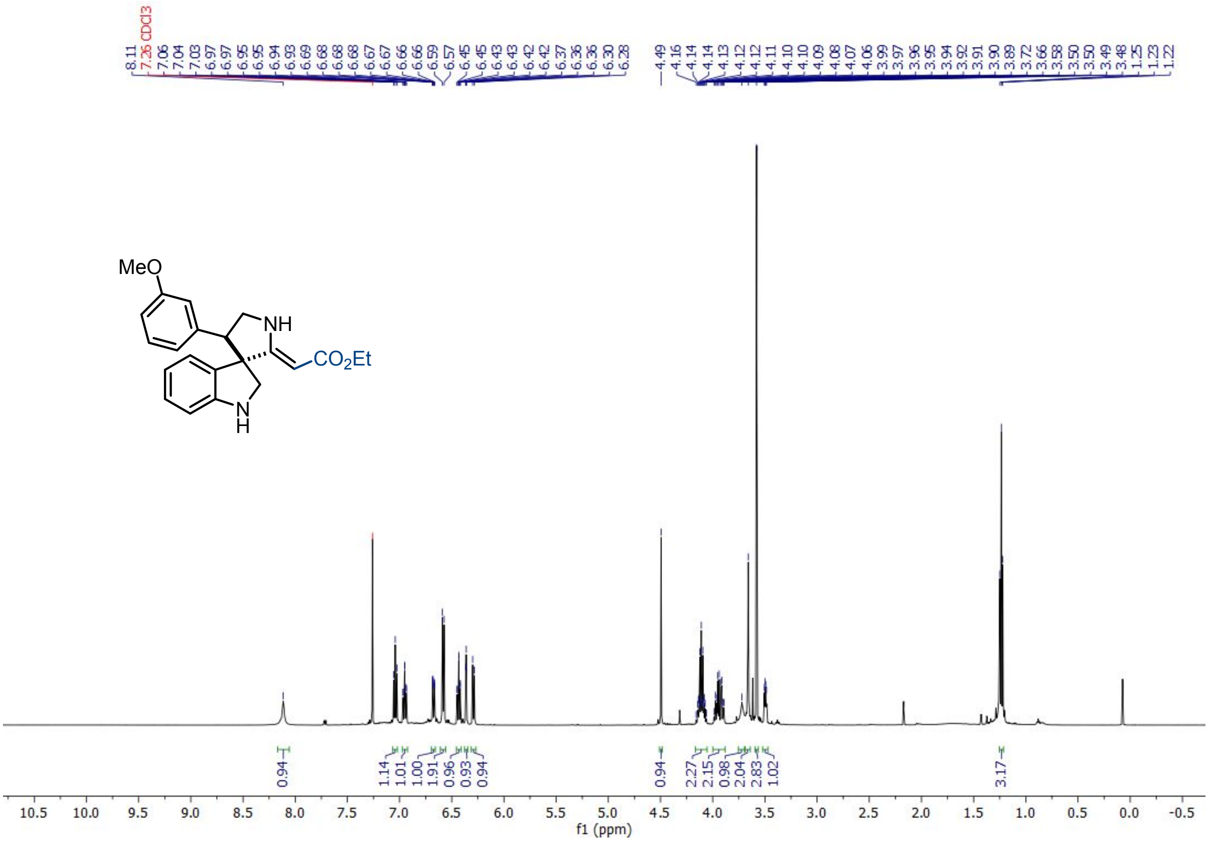

^{13}C{^{1}H} NMR 125.8 MHz, $CDCl_3$ **25x D2**

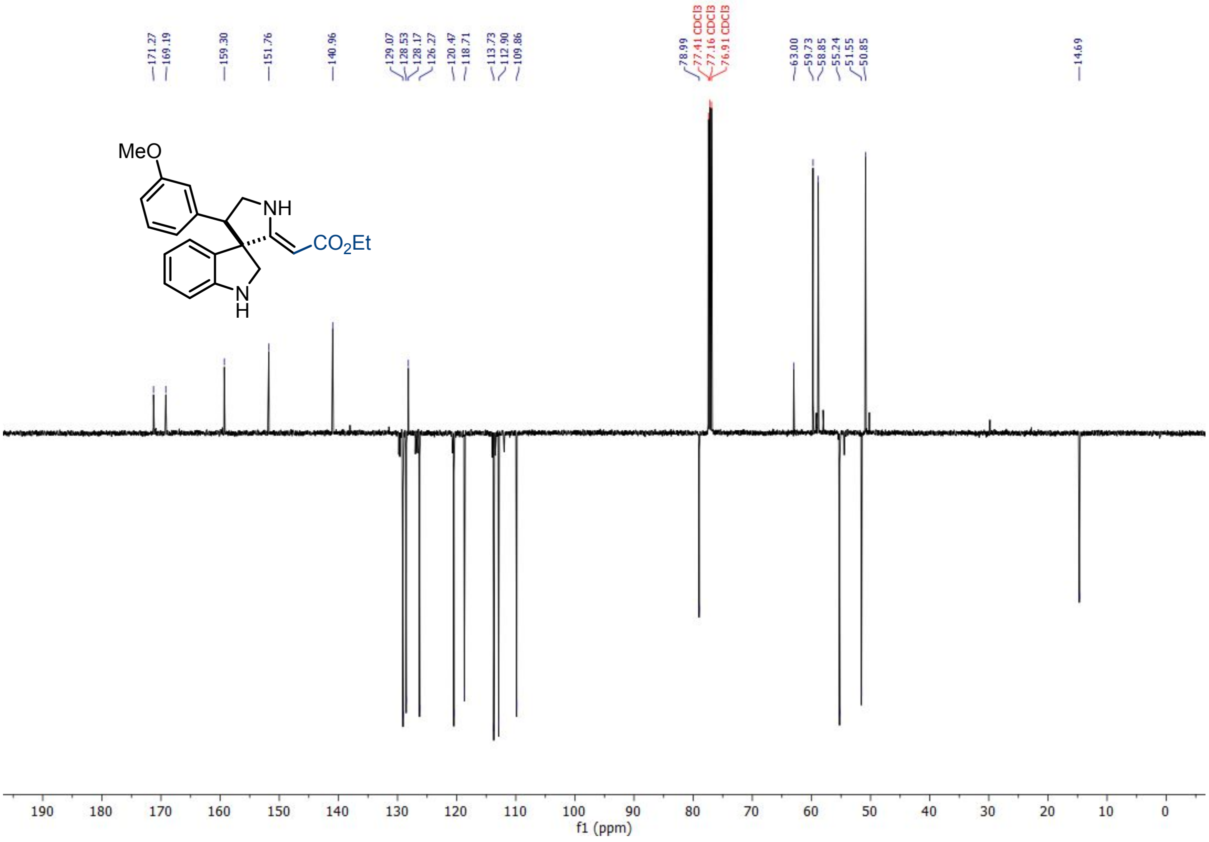

^{1}H NMR 600 MHz, $CDCl_3$ **23ab**

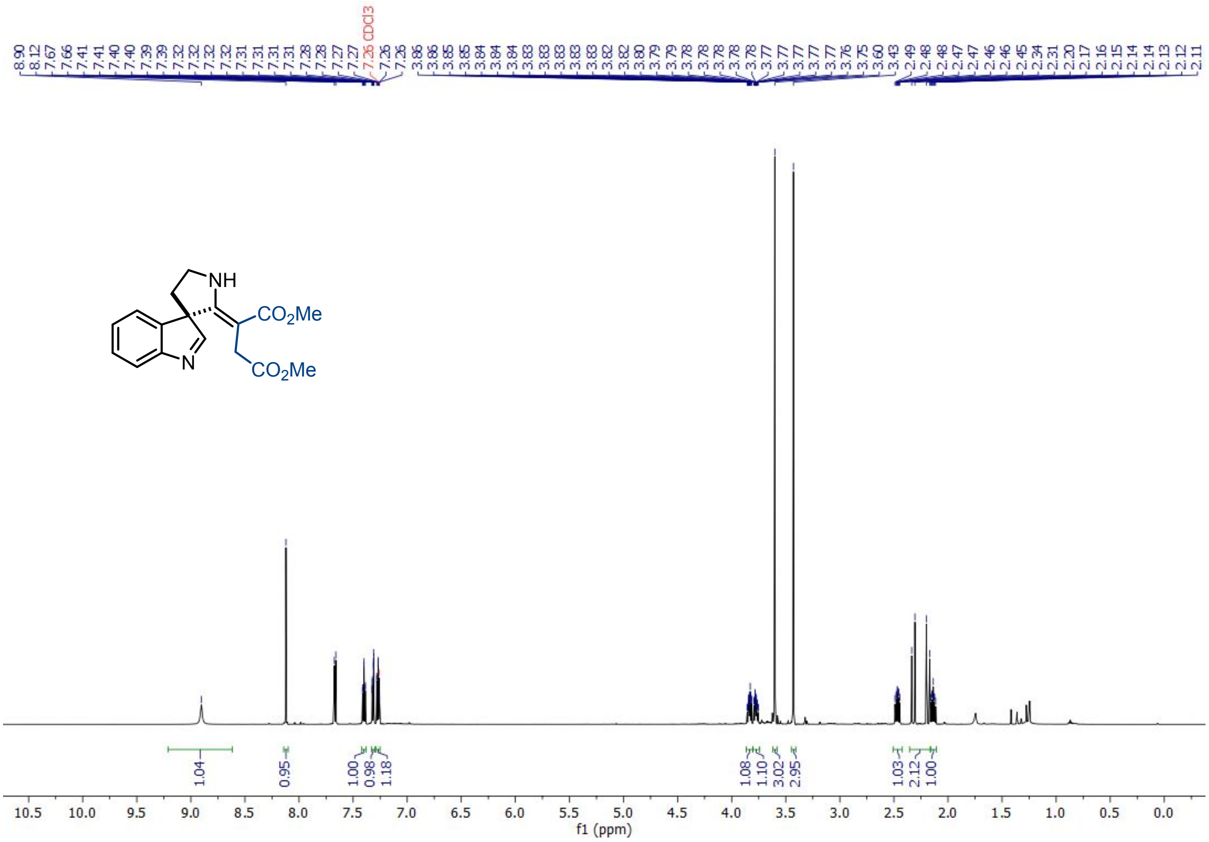

$^{13}C\{^{1}H\}$ NMR 150 MHz, $CDCl_3$ **23ab**

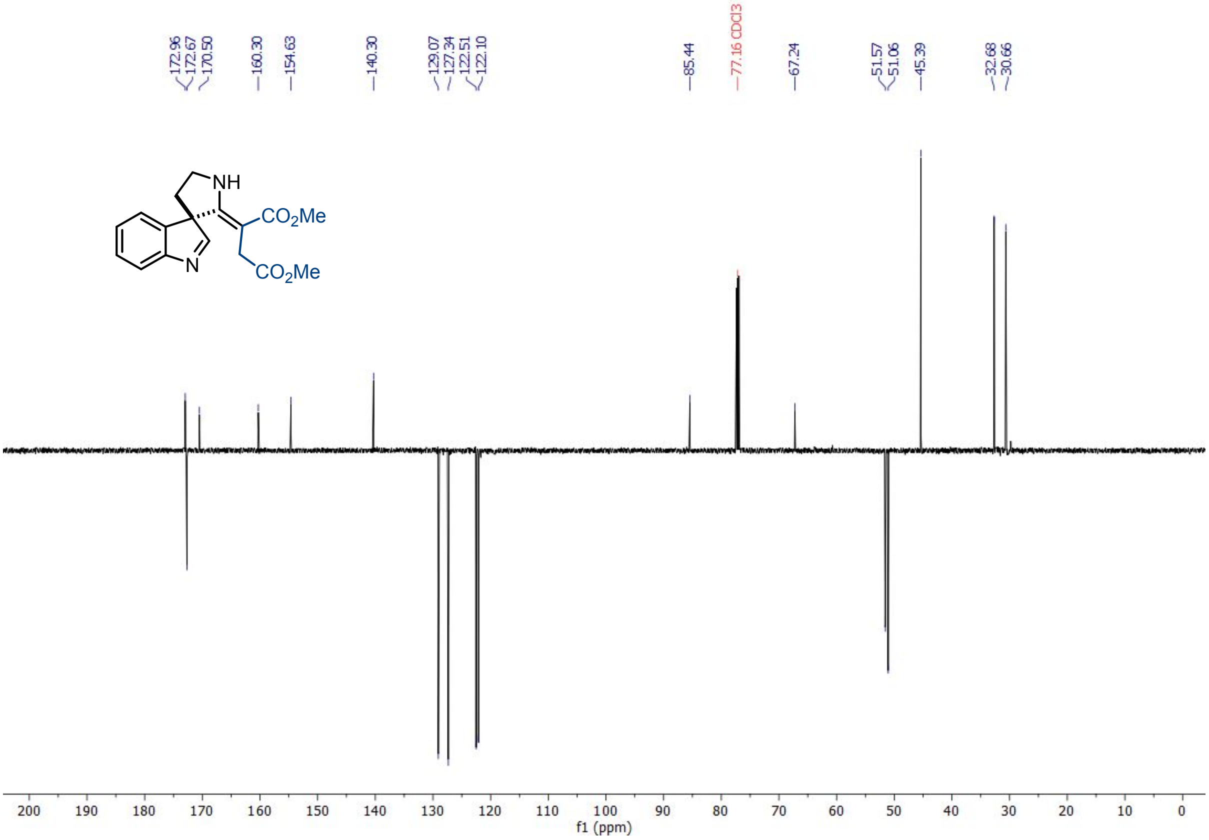

^1H NMR 600 MHz, $CDCl_3$ **23bb**

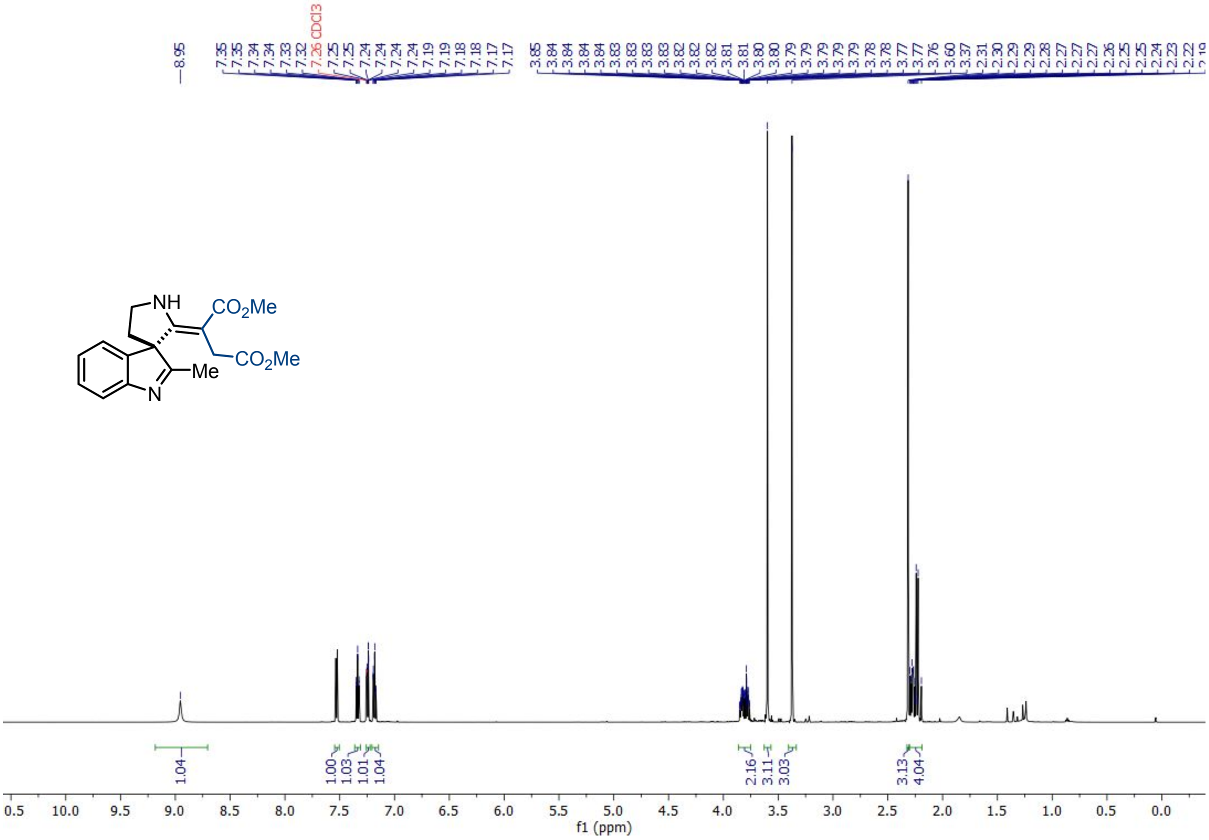

$^{13}C\{^1H\}$ NMR 150 MHz, $CDCl_3$ **23bb**

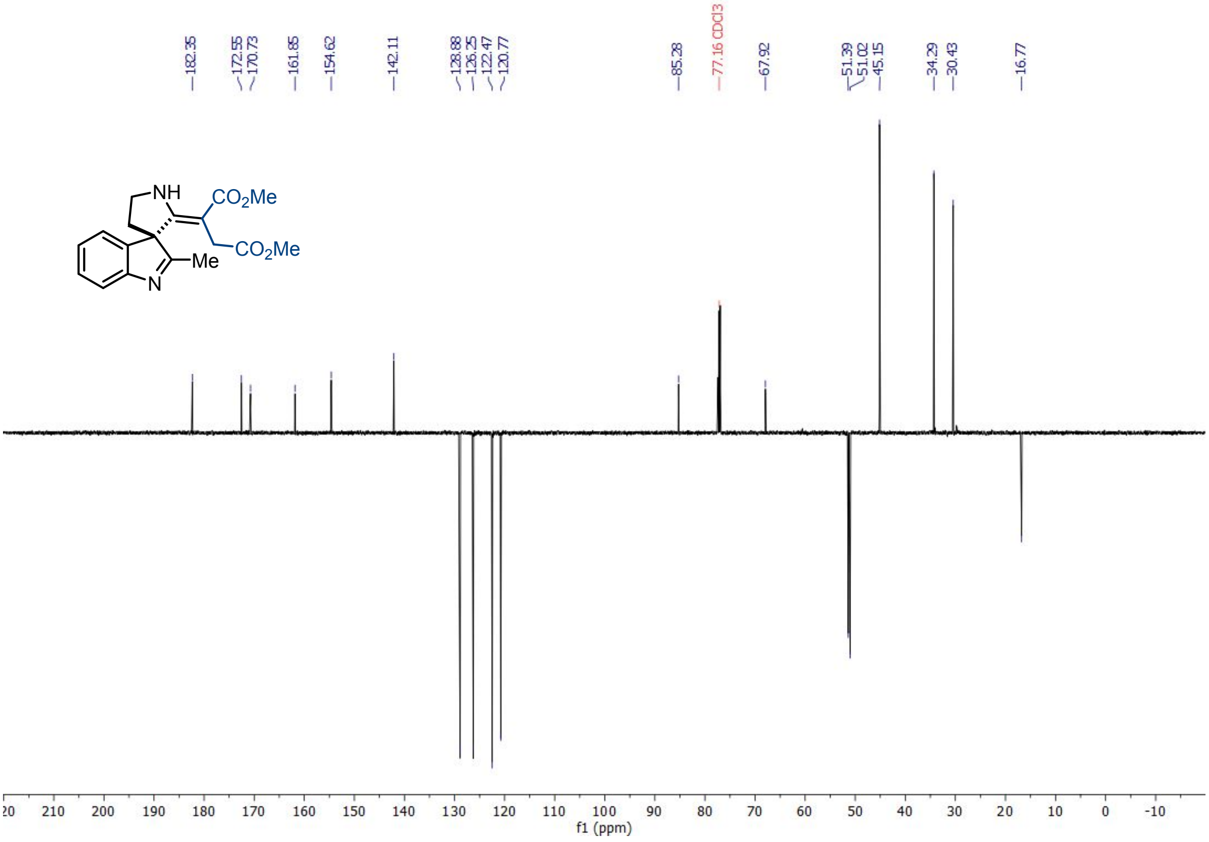

^{1}H NMR 500 MHz, $CDCl_3$ **23be**

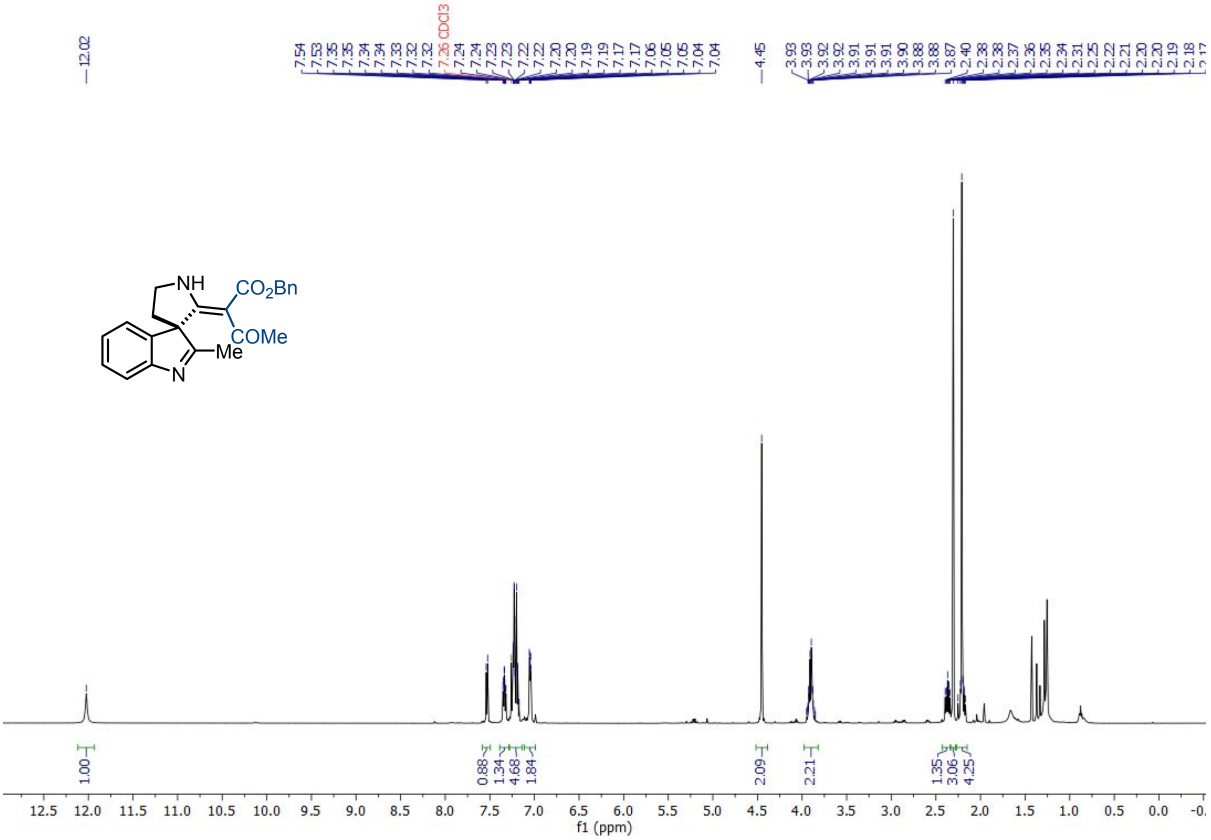

$^{13}C\{^{1}H\}$ NMR 126 MHz, $CDCl_3$ **23be**

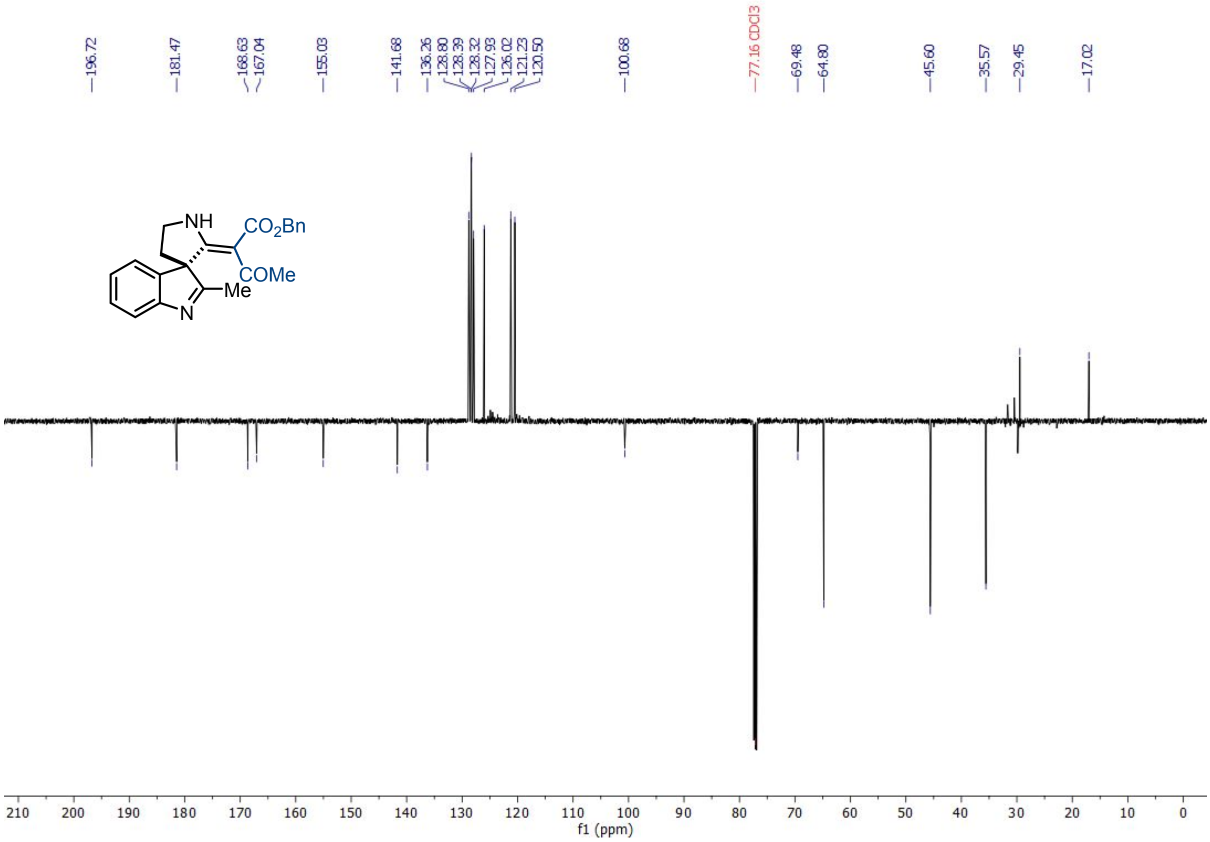

^{1}H NMR 500 MHz, $CDCl_3$ **acetate of 1y**

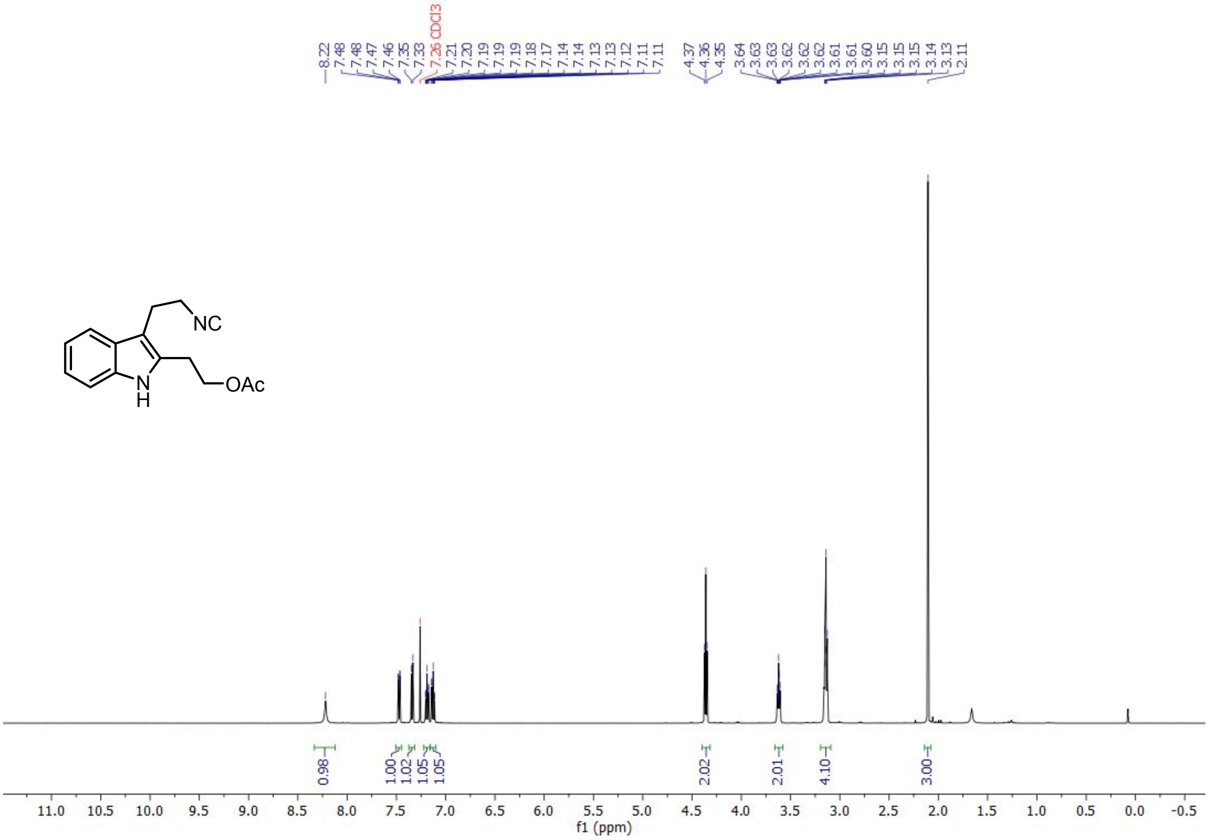

^{13}C{^{1}H} NMR 126 MHz, $CDCl_3$ **acetate of 1y**

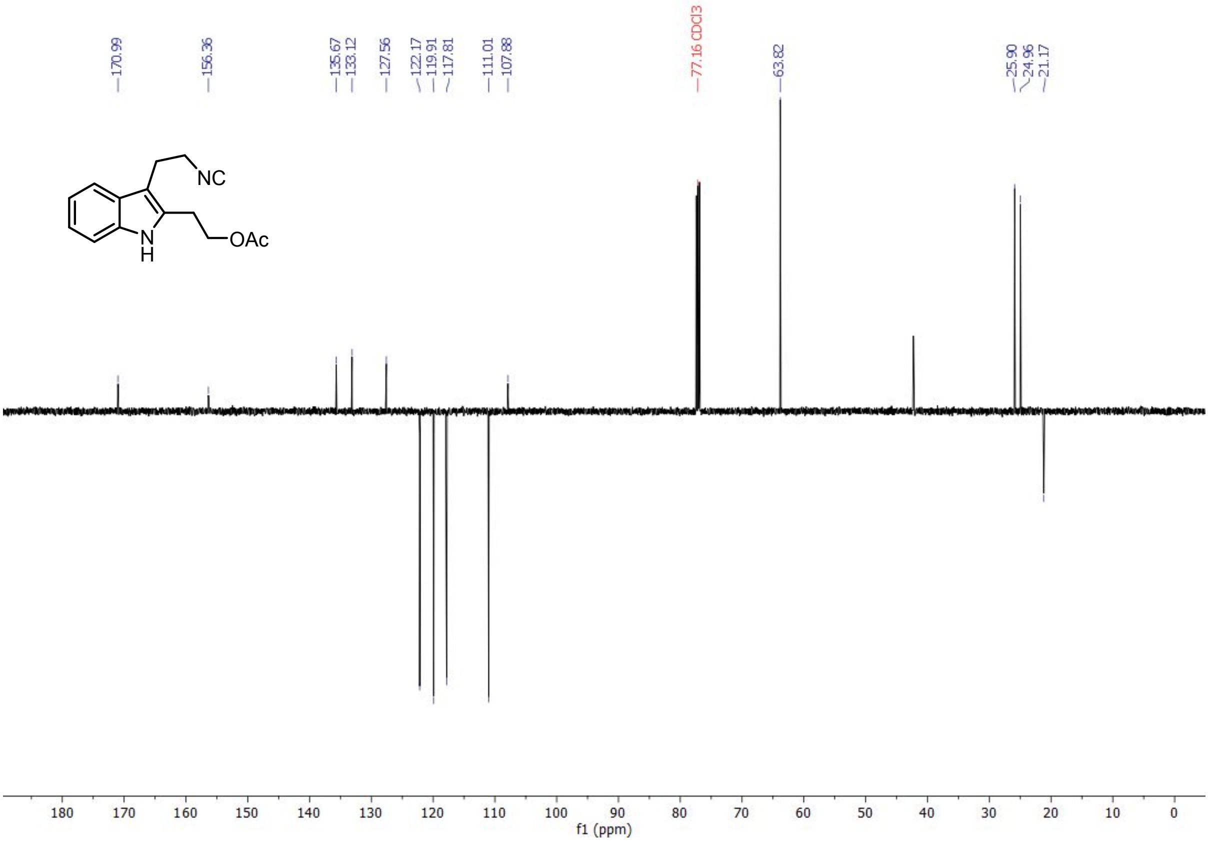

^{1}H NMR 500 MHz, $CDCl_3$ **1y**

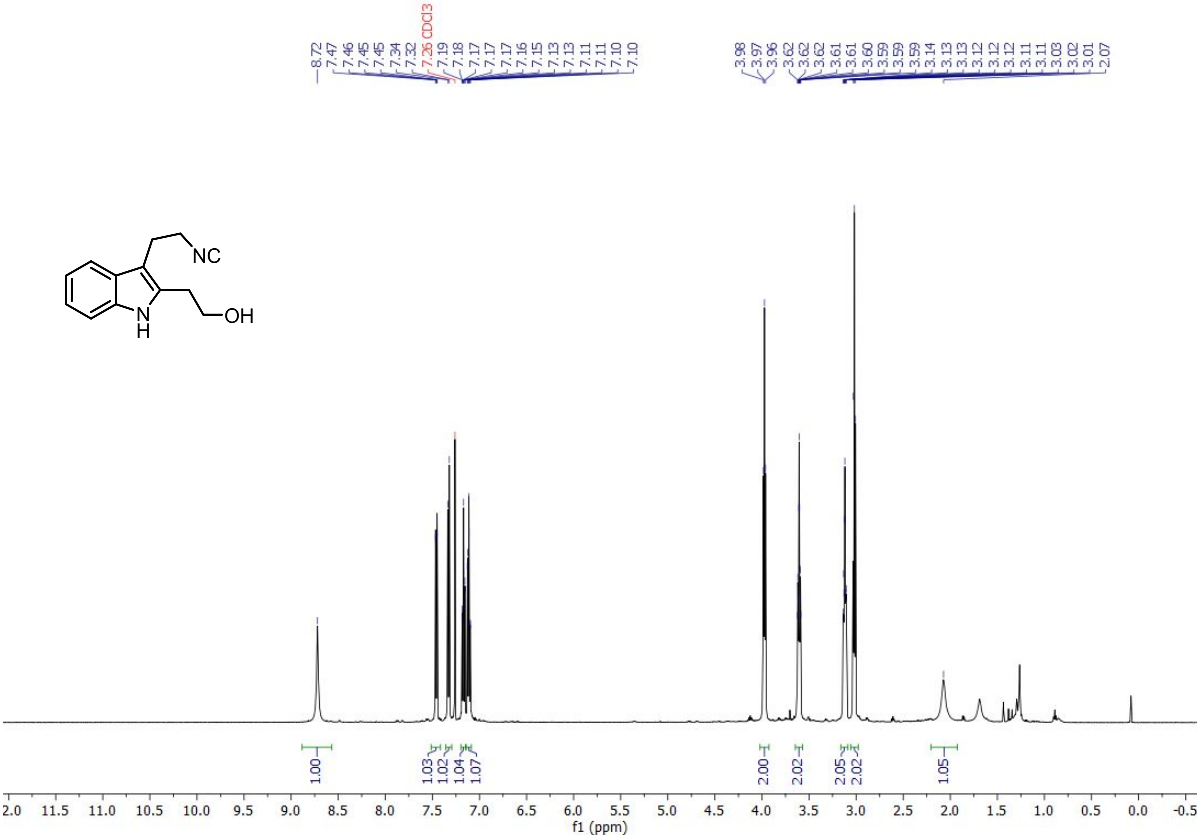

$^{13}C\{^{1}H\}$ NMR 126 MHz, $CDCl_3$ **1y**

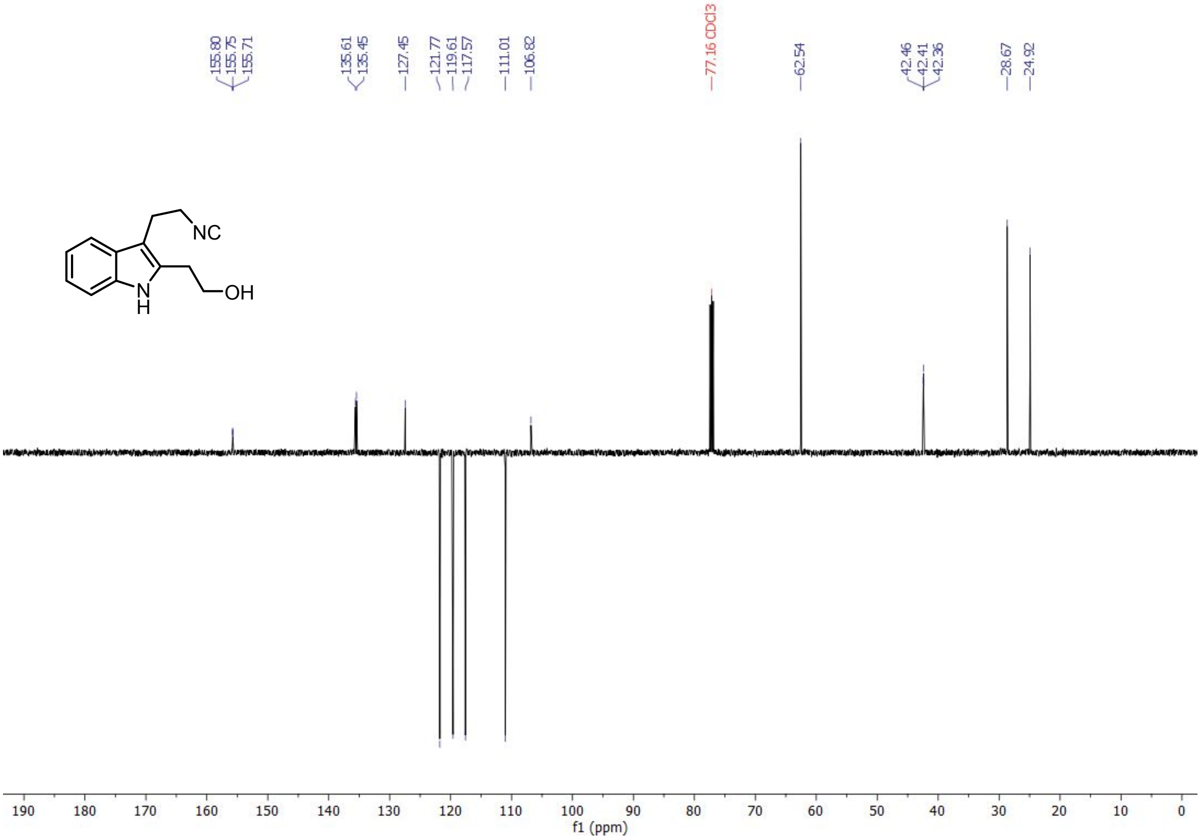

^{1}H NMR 500 MHz, $CDCl_3$ **25y**

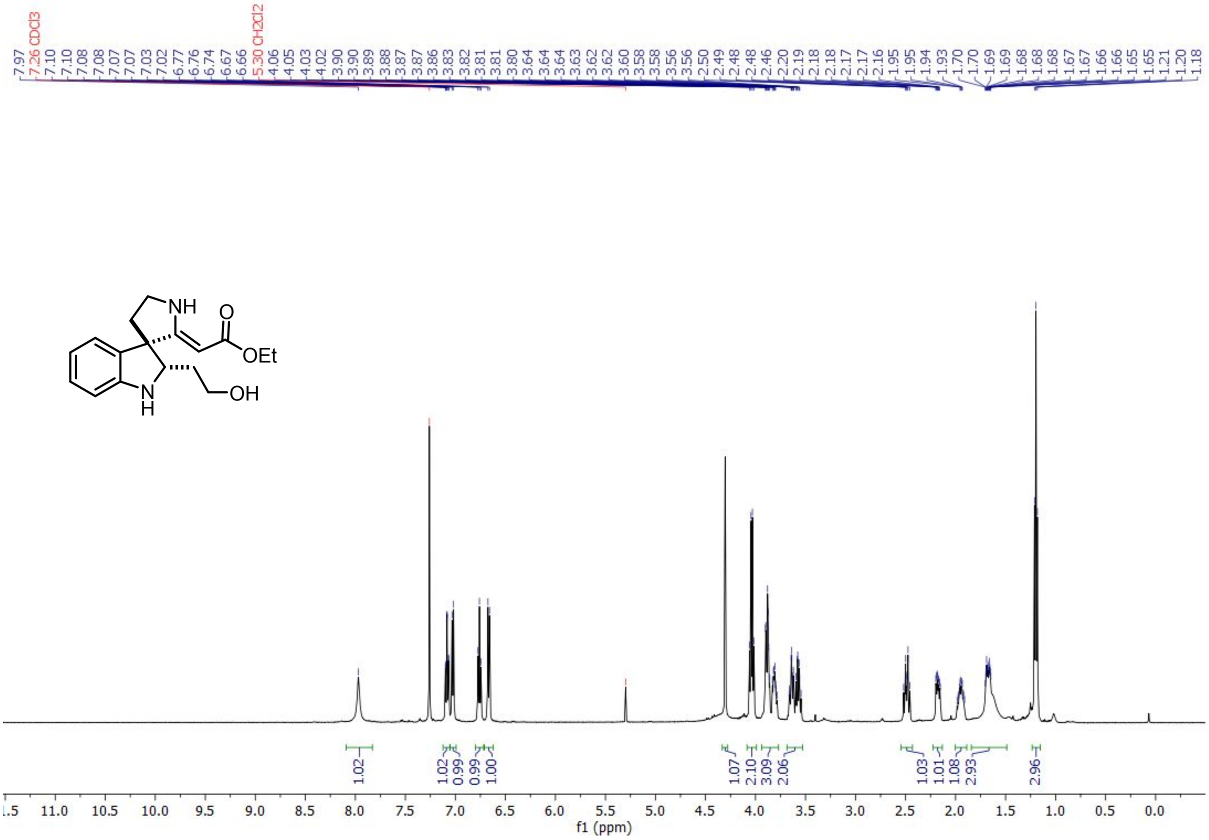

^{13}C{^{1}H} NMR 126 MHz, $CDCl_3$ **25y**

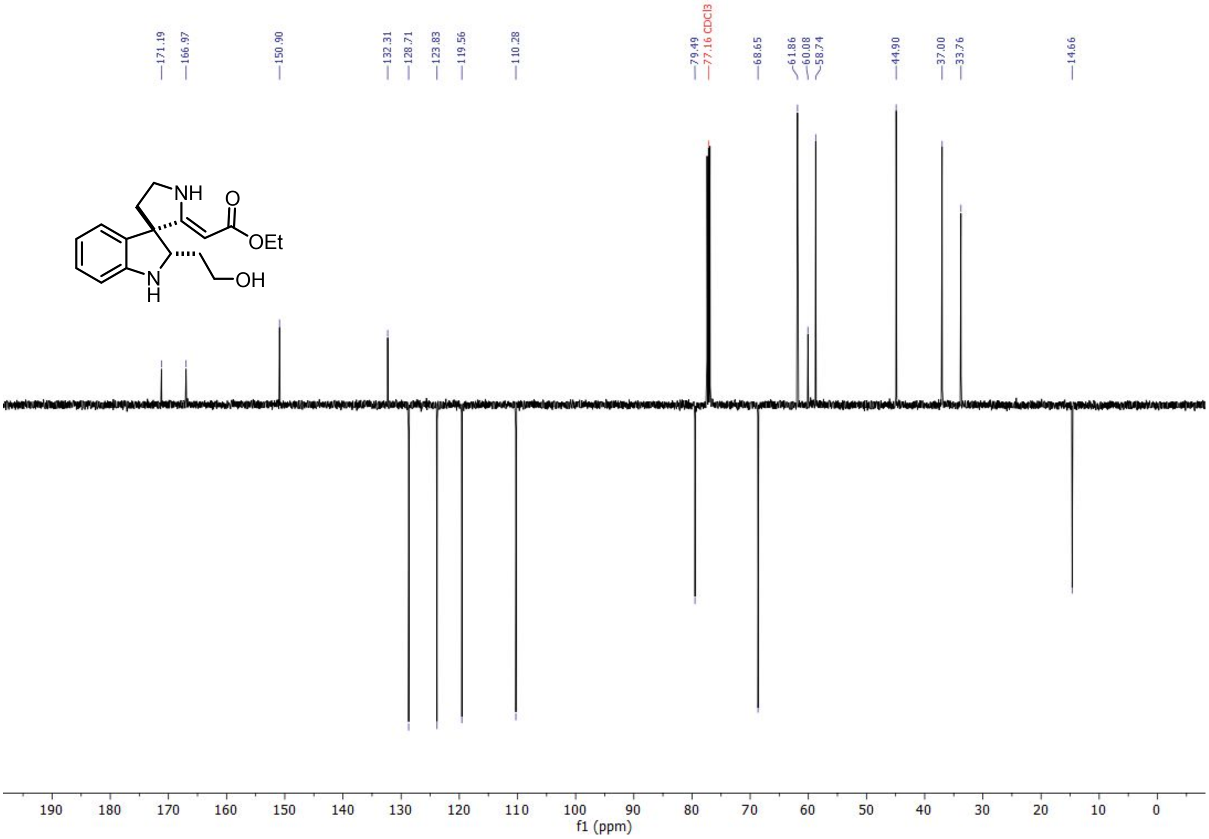

^{1}H NMR 500 MHz, $CDCl_3$ **26**

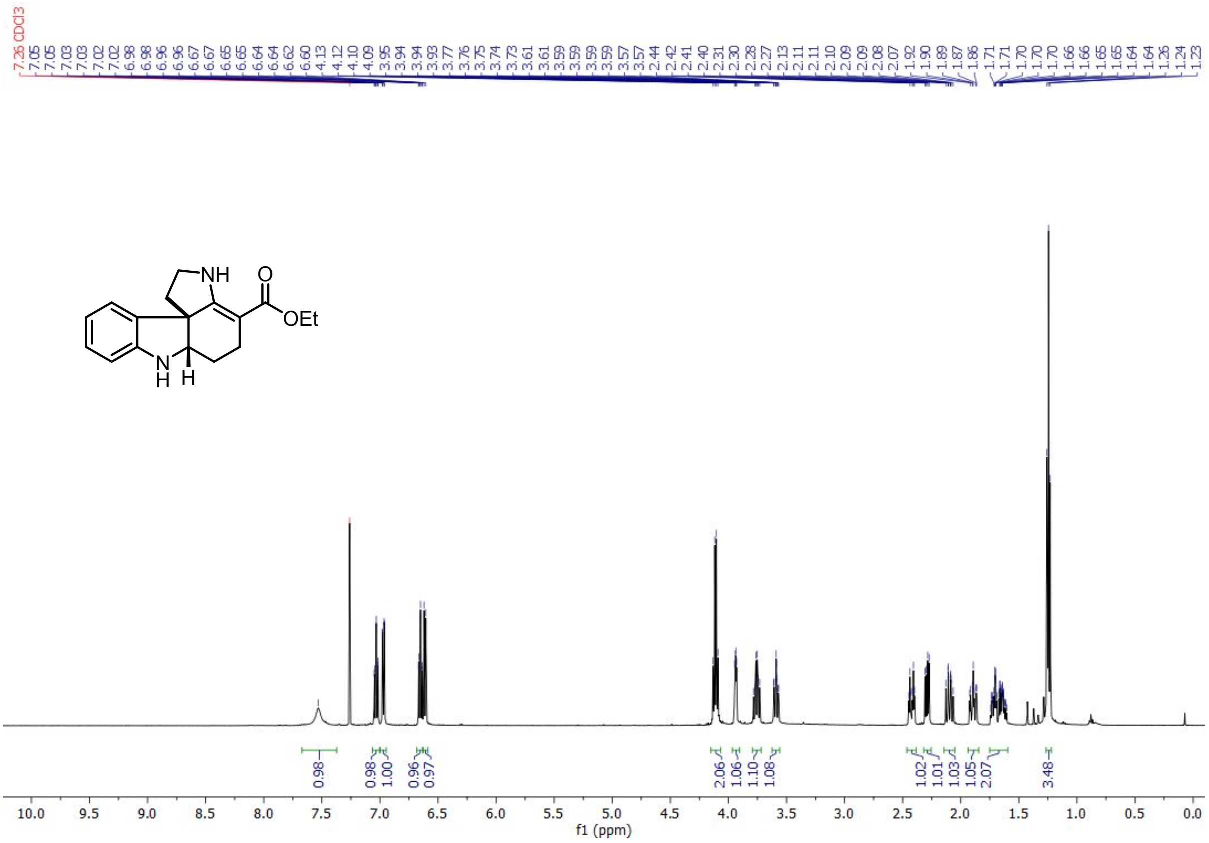

$^{13}C\{^{1}H\}$ NMR 126 MHz, $CDCl_3$ **26**

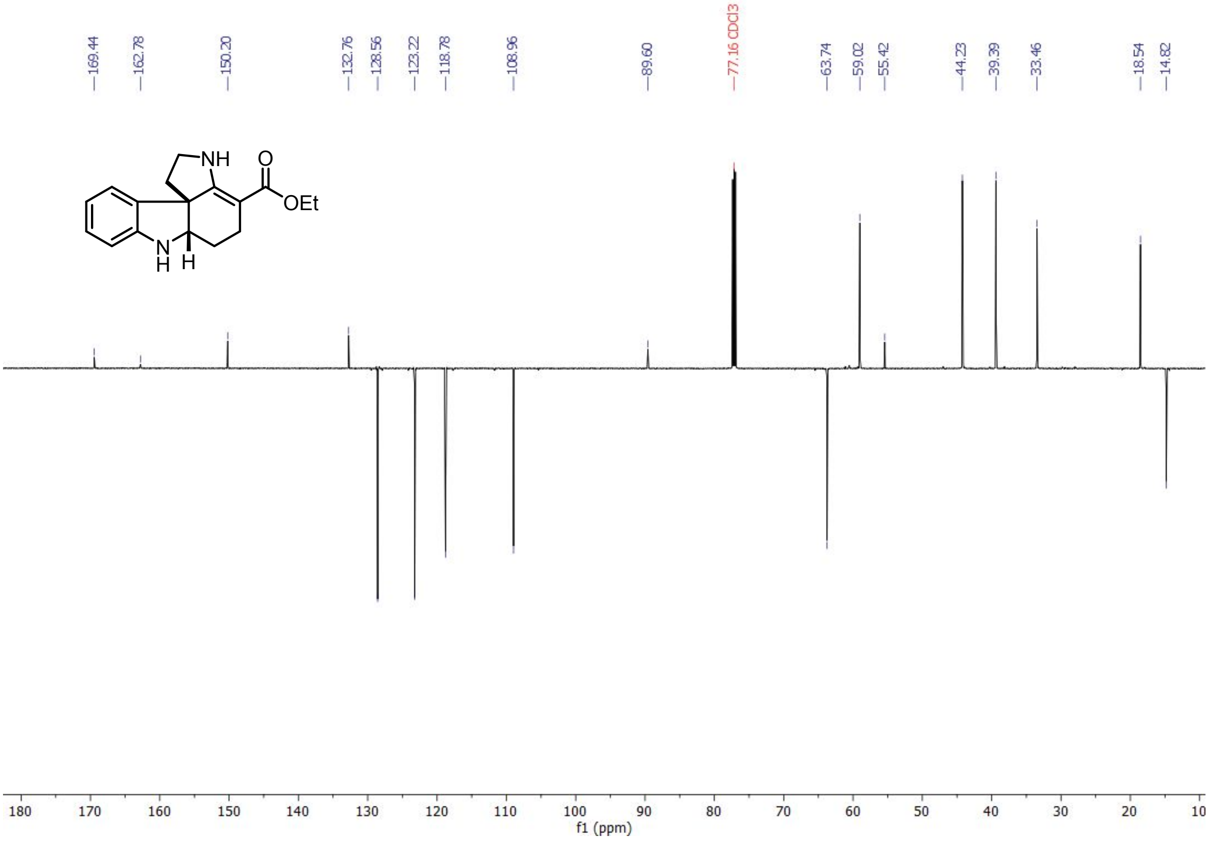

^1H NMR 600 MHz, $CDCl_3$ **20**

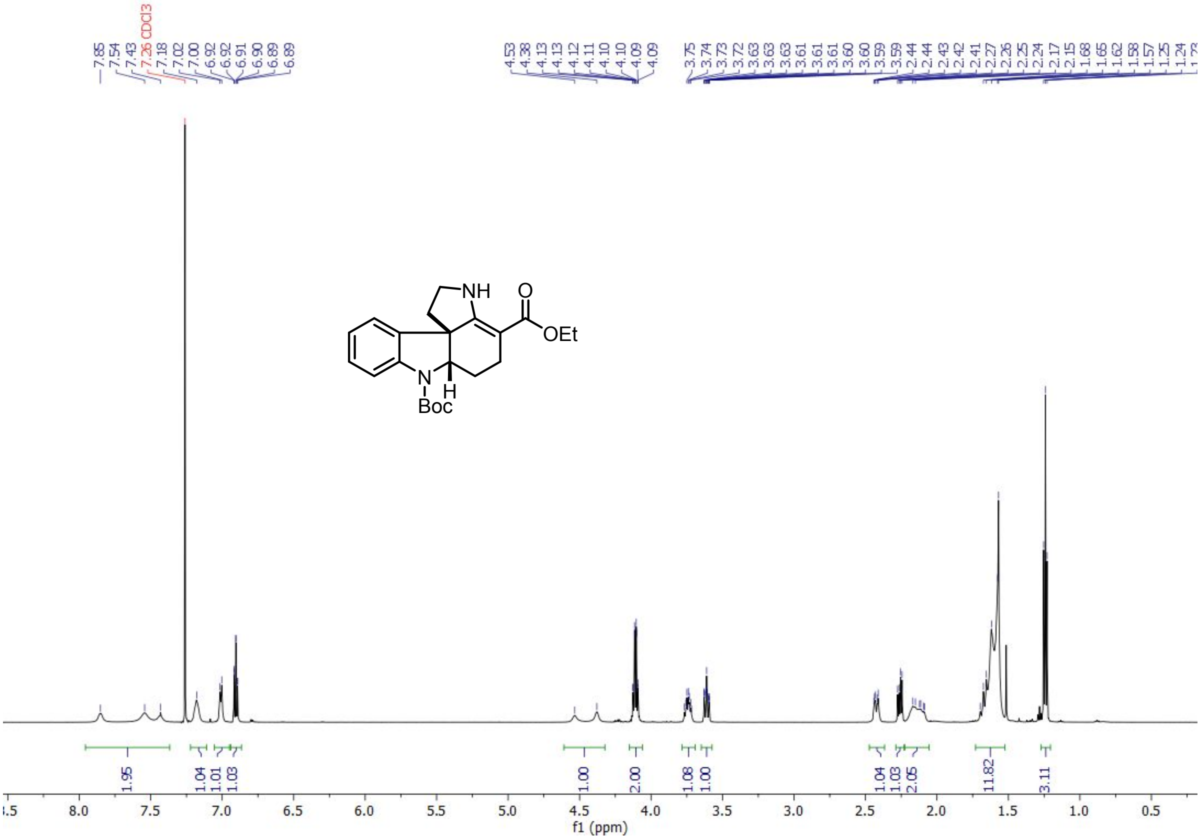

$^{13}C\{^1H\}$ NMR 151 MHz, $CDCl_3$ **20**

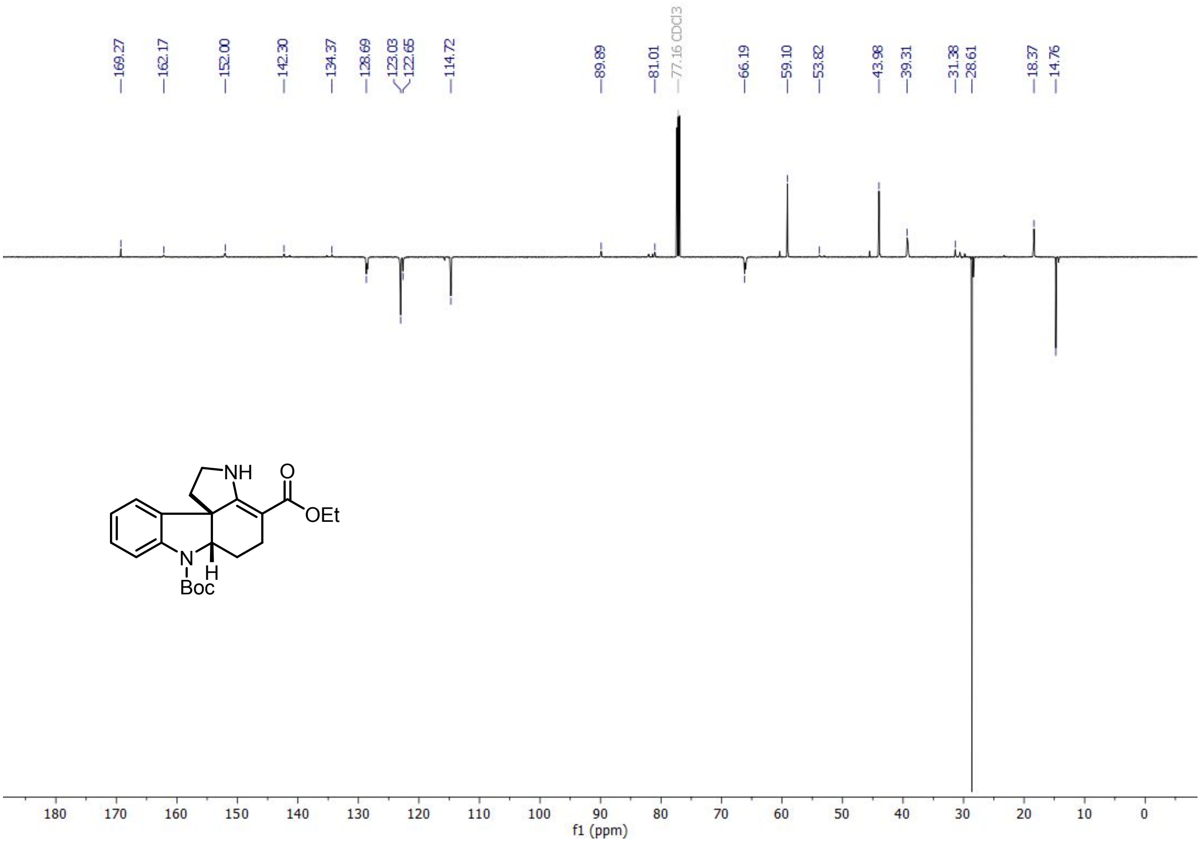

Supplement: Supplementary file 2 — jo3c02160_si_002.pdf [file jo3c02160_si_002.pdf]
