# Supplementary material for: Dearomative Spirocyclization of Tryptamine-Derived Isocyanides via Iron-Catalyzed Carbene Transfer
Source: J Org Chem. 2023 Dec 4;88(24):17345–55. doi: 10.1021/acs.joc.3c02160 (PMC10729054; doi:10.1021/acs.joc.3c02160)
Supplement: Supplementary file 1 — jo3c02160_si_001.zip [file jo3c02160_si_001.zip › FIDs_Fe_Carbene_Transfer_Tryptamine_Isocyanides_Roose_JOC/FID/23f/13C/pdata/1/email_BBN_034_2_1.pdf]

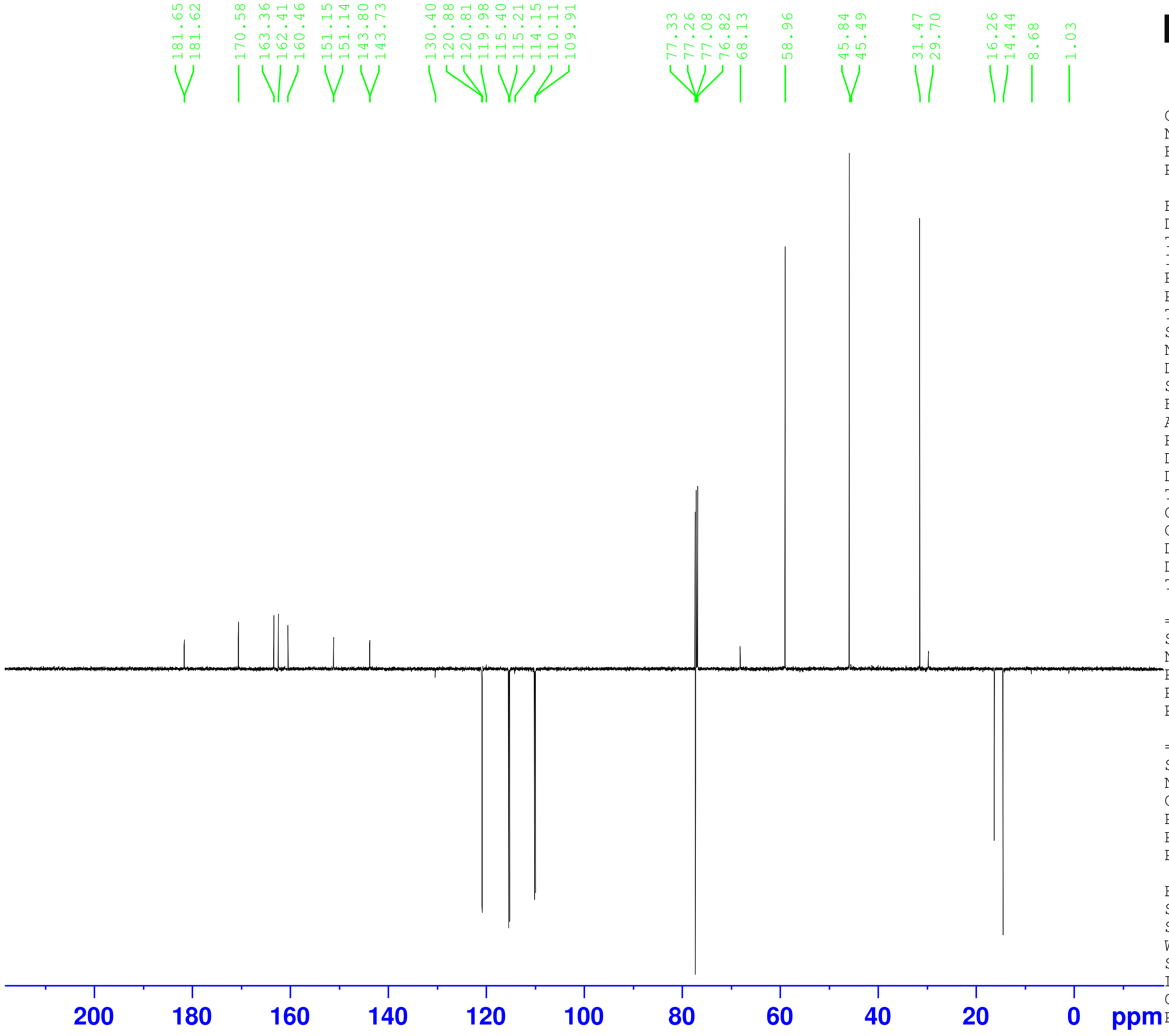

BRUKER

Current Data Parameters

NAME	BBN_034
EXPNO	2
PROCNO	1

F2 - Acquisition Parameters

Date_	20220218
Time	20.02
INSTRUM	spect
PROBHD	5 mm PABBO BB/
PULPROG	jmod
TD	65536
SOLVENT	CDCl3
NS	1024
DS	4
SWH	29761.904 Hz
FIDRES	0.454131 Hz
AQ	1.1010048 sec
RG	2050
DW	16.800 usec
DE	6.50 usec
TE	295.6 K
CNST2	145.0000000
CNST11	1.0000000
D1	2.00000000 sec
D20	0.00689655 sec
TD0	1

======== CHANNEL f1 ========

SFO1	125.7879676 MHz
NUC1	13C
P1	12.00 usec
P2	24.00 usec
PLW1	95.00000000 W

======== CHANNEL f2 ========

SFO2	500.2020008 MHz
NUC2	1H
CPDPRG[2	waltz16
PCPD2	80.00 usec
PLW2	22.00000000 W
PLW12	0.52855003 W

F2 - Processing parameters

SI	32768
SF	125.7753900 MHz
WDW	EM
SSB	0
LB	1.00 Hz
GB	0
PC	1.40
