# Supplementary material for: Dearomative Spirocyclization of Tryptamine-Derived Isocyanides via Iron-Catalyzed Carbene Transfer
Source: J Org Chem. 2023 Dec 4;88(24):17345–55. doi: 10.1021/acs.joc.3c02160 (PMC10729054; doi:10.1021/acs.joc.3c02160)
Supplement: Supplementary file 1 — jo3c02160_si_001.zip [file jo3c02160_si_001.zip › FIDs_Fe_Carbene_Transfer_Tryptamine_Isocyanides_Roose_JOC/FID/23h/13C/pdata/1/email_BBN_033_B_1_1.pdf]

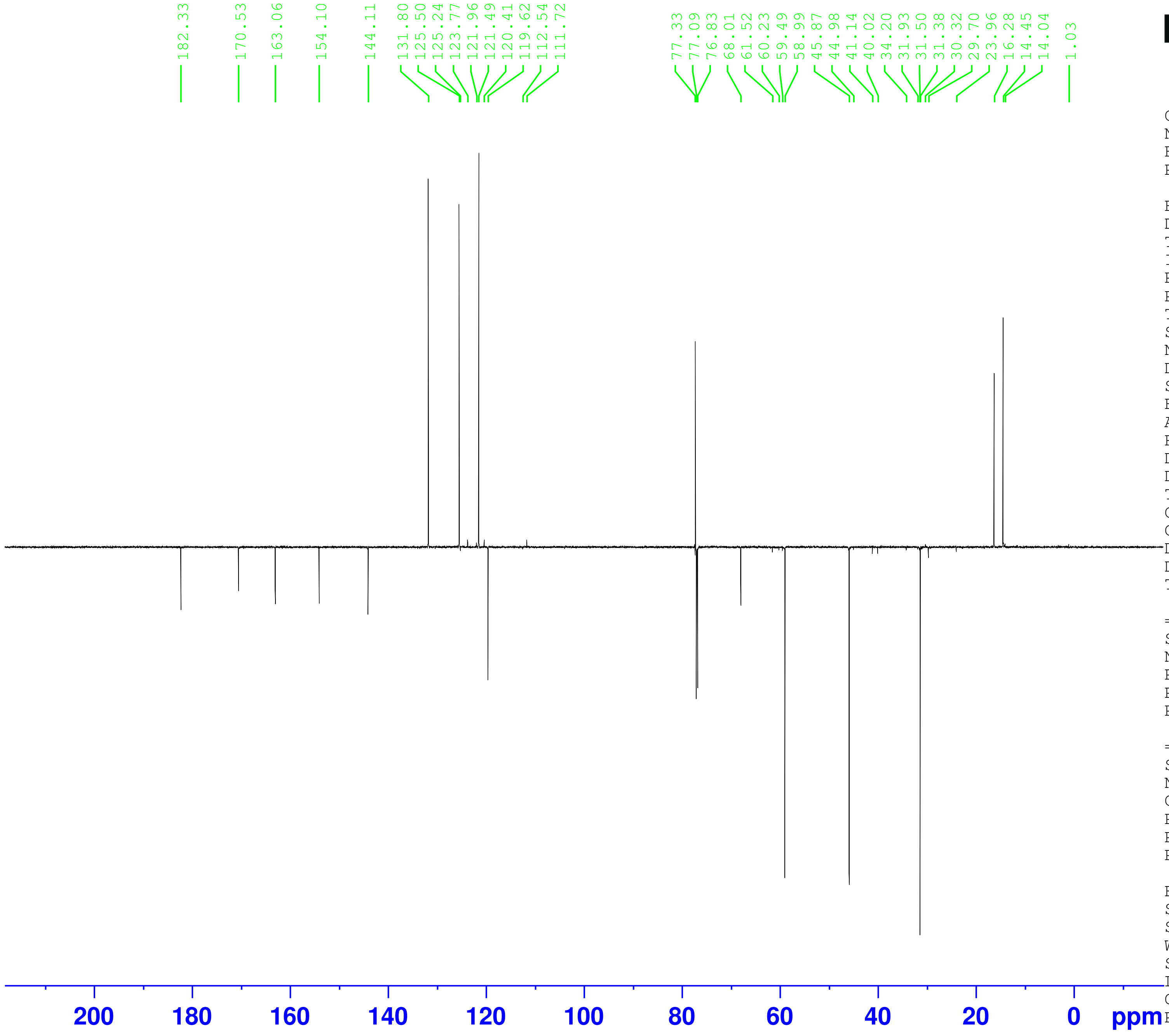


Current Data Parameters
NAME BBN_033_B
EXPNO 1
PROCNO 1

F2 - Acquisition Parameters
Date_ 20220219
Time 1.04
INSTRUM spect
PROBHD 5 mm PABBO BB/
PULPROG jmod
TD 65536
SOLVENT CDCl3
NS 2048
DS 4
SWH 29761.904 Hz
FIDRES 0.454131 Hz
AQ 1.1010048 sec
RG 2050
DW 16.800 usec
DE 6.50 usec
TE 295.5 K
CNST2 145.0000000
CNST11 1.0000000
D1 2.00000000 sec
D20 0.00689655 sec
TD0 1

======== CHANNEL f1 ========
SFO1 125.7879676 MHz
NUC1 13C
P1 12.00 usec
P2 24.00 usec
PLW1 95.00000000 W

======== CHANNEL f2 ========
SFO2 500.2020008 MHz
NUC2 1H
CPDPRG[2 waltz16
PCPD2 80.00 usec
PLW2 22.00000000 W
PLW12 0.52855003 W

F2 - Processing parameters
SI 32768
SF 125.7753900 MHz
WDW EM
SSB 0
LB 1.00 Hz
GB 0
PC 1.40
