# Supplementary material for: Dearomative Spirocyclization of Tryptamine-Derived Isocyanides via Iron-Catalyzed Carbene Transfer
Source: J Org Chem. 2023 Dec 4;88(24):17345–55. doi: 10.1021/acs.joc.3c02160 (PMC10729054; doi:10.1021/acs.joc.3c02160)
Supplement: Supplementary file 1 — jo3c02160_si_001.zip [file jo3c02160_si_001.zip › FIDs_Fe_Carbene_Transfer_Tryptamine_Isocyanides_Roose_JOC/FID/23i/1H/pdata/1/email_BBN_035_1_1.pdf]

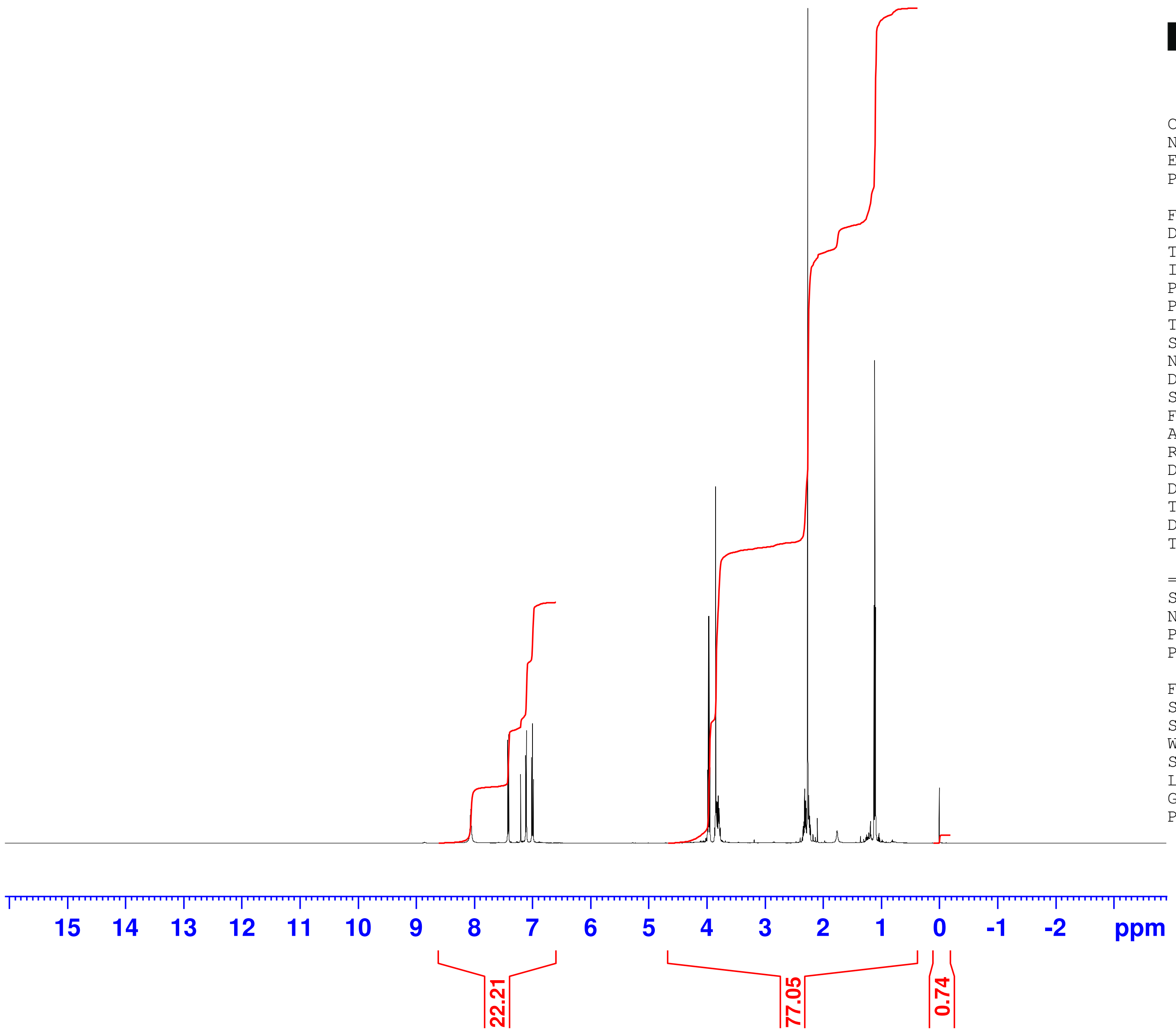

BRUKER

Current Data Parameters
NAME BBN_035
EXPNO 1
PROCNO 1

F2 - Acquisition Parameters
Date_ 20220218
Time 20.09
INSTRUM spect
PROBHD 5 mm PABBO BB/
PULPROG zg30
TD 65536
SOLVENT CDCl3
NS 16
DS 2
SWH 10000.000 Hz
FIDRES 0.152588 Hz
AQ 3.2767999 sec
RG 101
DW 50.000 usec
DE 6.50 usec
TE 295.2 K
D1 1.00000000 sec
TD0 1

======== CHANNEL f1 ========
SFO1 500.2030889 MHz
NUC1 1H
P1 12.40 usec
PLW1 22.00000000 W

F2 - Processing parameters
SI 65536
SF 500.2000456 MHz
WDW EM
SSB 0
LB 0.30 Hz
GB 0
PC 1.00
